# Genetic and epigenetic background and protein expression profiles in relation to telomerase activation in medullary thyroid carcinoma

**DOI:** 10.18632/oncotarget.7237

**Published:** 2016-02-08

**Authors:** Na Wang, Hanna Kjellin, Anastasios Sofiadis, Omid Fotouhi, C. Christofer Juhlin, Martin Bäckdahl, Jan Zedenius, Dawei Xu, Janne Lehtiö, Catharina Larsson

**Affiliations:** ^1^ Department of Oncology-Pathology, Karolinska Institutet, SE-171 76 Stockholm, Sweden; ^2^ Cancer Center Karolinska, Karolinska University Hospital R8:04, SE-171 76 Stockholm, Sweden; ^3^ Department of Molecular Medicine and Surgery, Karolinska Institutet, SE-171 76 Stockholm, Sweden; ^4^ Science for Life Laboratory, SE-171 21 Solna, Sweden; ^5^ Department of Medicine-Solna, Division of Hematology and Center for Molecular Medicine, Karolinska Institutet and Karolinska University Hospital Solna, Stockholm, Sweden

**Keywords:** medullary thyroid carcinoma, methylation, proteomics, telomerase

## Abstract

Medullary thyroid carcinomas (MTCs) exhibit telomerase activation in strong association with shorter patient survival. To understand the background of telomerase activation we quantified *TERT* copy numbers and *TERT* promoter methylation in 42 MTCs and normal thyroid references. Gain of *TERT* was demonstrated by quantitative PCR in 5/39 sporadic MTC. Increased methylation index (MetI) for CpG methylation at the *TERT* promoter was found in sporadic MTCs (*P* < 0.0001) and in MEN 2 associated MTCs (*P* = 0.011) vs. normal thyroid tissues. MetI correlated positively with *TERT* gene expression (*r* = 0.432, *P* = 0.006) and negatively with telomere length (*r* = −0.343, *P* = 0.032). MTC cases with MetI above the median of 52% had shorter survival as compared to cases with lower MetI (*P* = 0.005 for overall survival and *P* = 0.007 for disease-related survival). Protein expression profiles obtained by mass spectrometry were then studied in relation to telomerase activation in MTCs. Comparing protein levels between tumors defined by telomerase activity status, 240 proteins were associated with telomerase activity. Among telomerase activation positive cases a set of proteins was found to discriminate between MTCs with high and low *TERT* gene expression with enrichment for proteins involved in telomerase regulation. *XRCC5* mRNA expression was found increased in MTCs vs. normal thyroid (*P* = 0.007). In conclusion the findings suggest a role for *TERT* copy number gain, *TERT* promoter methylation and *XRCC5* expression in telomerase activation and telomere maintenance of MTC.

## INTRODUCTION

**Figure 1:**
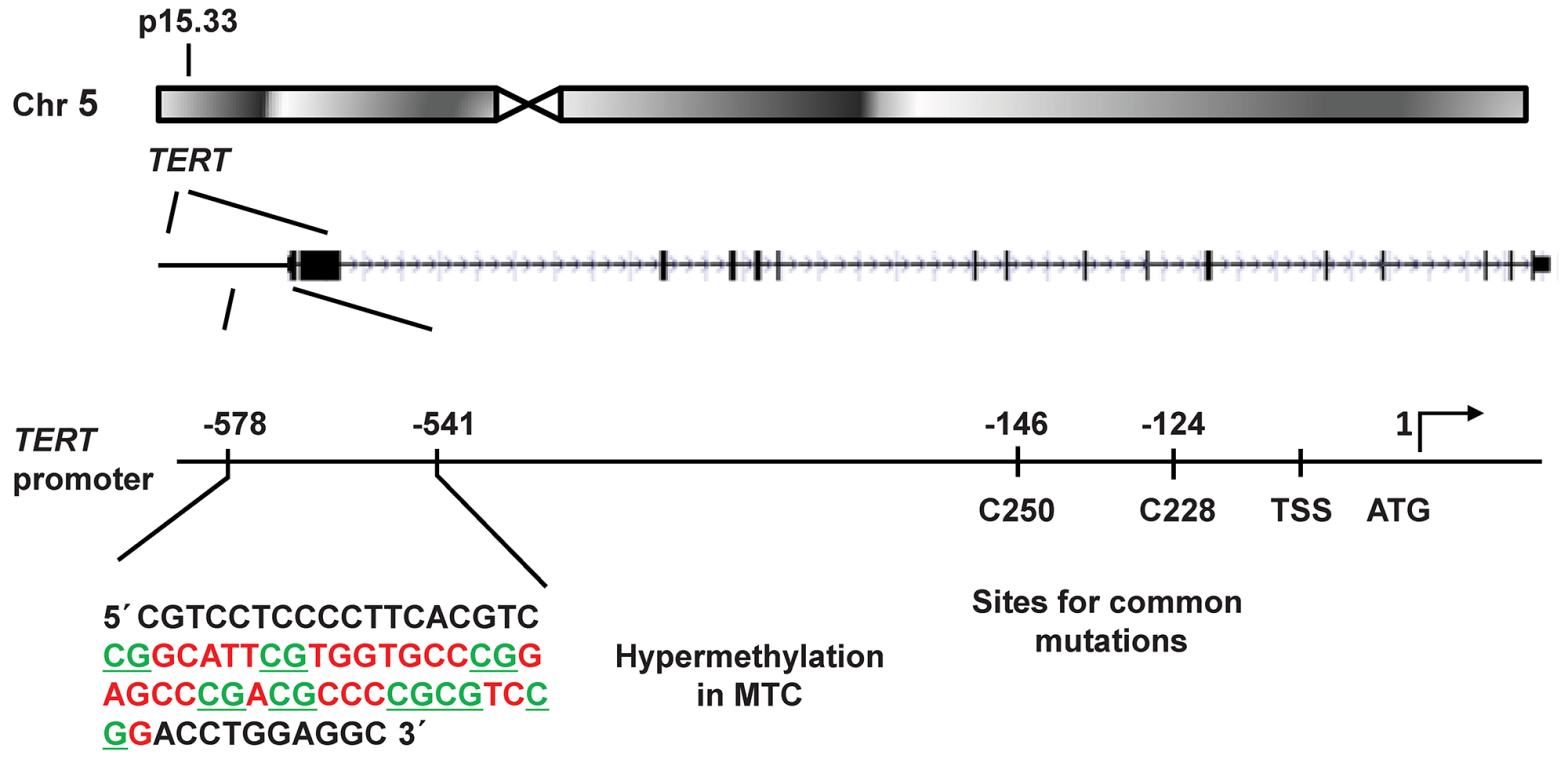
Schematic illustration of the *TERT* gene locus in chromosomal region 5p15.33 and its promoter region The investigated promoter sequence showing CpG hypermethylation in MTCs is shown in an enlargement to the left. The location of the common mutations C250T and C228T are indicated to the right together with the transcription start site (TSS) and the translation start site (ATG). The eight CpG sites analysed here are underlined.

In order to survive, cancer cells must activate a cellular mechanism for telomere maintenance. This is commonly achieved by activation of the ribonucleoprotein telomerase through the telomerase reverse transcriptase component encoded by the *TERT* gene [1]. Telomerase may then elongate telomeric DNA allowing for continued cancer cell proliferation. Telomerase activation is observed in the majority of human cancers, and *TERT*, which is normally suppressed, is frequently over-expressed in cancer cells. This may be due to e.g. specific mutations in the *TERT* promoter (Figure 1) that are common in certain cancer types, as initially described in melanoma [2, 3]. In follicular-cell derived thyroid carcinomas *TERT* promoter mutations were found to be associated with telomerase activation, altered telomere length and poor survival [4, 5]. In medullary thyroid carcinoma (MTC) *TERT* mutations were not observed [4, 6]. However, telomerase activation was detected in approximately 50% of MTCs, together with expression of one or more of three different splicing forms of *TERT* [7]. We found that the activation of telomerase and expression of the full-length *TERT* transcript had strong prognostic influence on patient survival [7]. In addition, a subset of telomerase negative MTCs were found to exhibit the alternative lengthening of telomere (ALT) phenotype [7]. However, the mechanism for activation of telomerase in MTC is not fully understood.

MTC arises from calcitonin-producing C-cells and is the main component of the hereditary multiple endocrine neoplasia type 2 (MEN 2) syndromes also associated with pheochromocytoma and primary hyperparathyroidism [8]. The genetic background of sporadic and familial MTC includes frequent activating mutations of the *RET* (REarranged during Transfection) proto-oncogene [9]. In *RET* negative tumors, mutations of *HRAS* or *KRAS* are recurrently observed [10], and exome-sequencing of MTCs support that the *RET* and *RAS* pathways are prominent drivers in this tumor type [11].

Aberrant gene expression in human cancer may result from different types of molecular genetic alterations. In addition to mutations, numerical DNA alterations and chromosomal rearrangements, the importance of epigenetic modifications is increasingly recognized. This may include e.g. altered DNA methylation affecting gene expressions. The possible involvement of DNA copy number alterations and DNA methylation in telomerase activation has not been explored in MTC. Indeed, CpG hypermethylation of the *TERT* promoter has been demonstrated in some other cancer types and associated with increased *TERT* expression and patient outcome [12].

Here we aimed to further elucidate mechanisms of telomerase activation and the effects on protein expression profiles in MTC. For this purpose we quantified DNA copy numbers and promoter methylation in a series of 42 MTCs and evaluated the findings in relation to telomerase activity, telomere length and clinical outcome. Furthermore, protein expression profiles were established using a proteomics approach.

## RESULTS

### *TERT* copy number gain in a subset of telomerase positive MTCs

**Figure 2:**
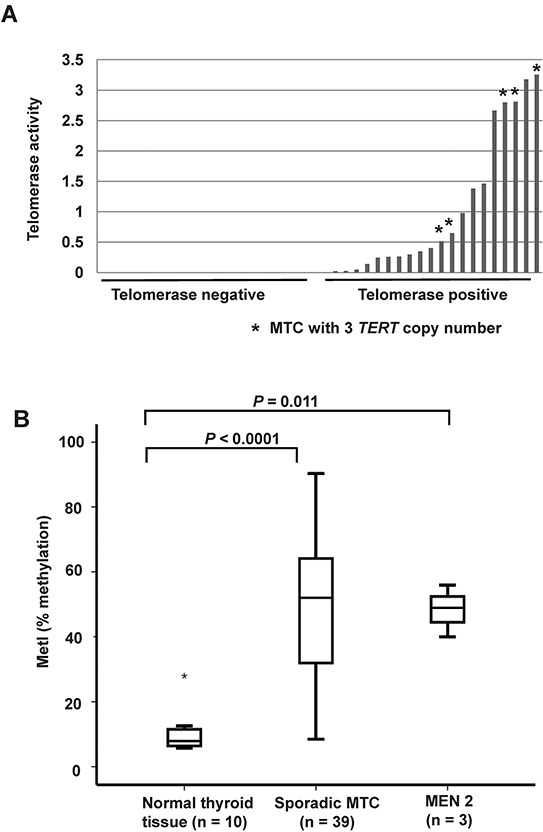
*TERT* copy number gain and promoter hypermethylation in sporadic MTCs **A.** Comparison of DNA copy numbers of *TERT* and telomerase activity in the 39 sporadic MTCs. The same calibrator was used for both experiments. Each bar represents one MTC, and the height of the bar indicates the level of telomerase activity. The five MTCs with three *TERT* copies are indicated by an asterisk (*). All other samples have two copies of *TERT*. **B.** Box plots showing increased *TERT* promoter methylation in 39 sporadic MTCs and in the three MEN 2 cases as compared to 10 normal thyroid tissues. The outlier is indicated by an asterisk (*).

DNA copy numbers of the *TERT* locus were determined in the 42 MTC samples and 10 normal thyroid tissues using a Taqman based assay. Five of the 39 (13%) sporadic MTCs displayed three copies of *TERT* while 34 tumors showed two copies (Figure 2A). The three MEN 2 cases and all normal thyroid tissues revealed two copies of the *TERT* gene locus. All five cases with copy number gain, i.e. three *TERT* copies, exhibited *TERT* expression and telomerase activity (Figure 2A). Furthermore, they presented with late stage disease at the diagnosis (Supplementary Table S1). At the end of follow-up, two patients had died from MTC, two were alive with persistent disease and one had died from other disease.

### Increased *TERT* promoter methylation in MTCs correlate with telomerase activity, telomere length and *RAS* mutations

Specific *TERT* promoter methylation density was quantified at eight CpG sites by Pyrosequencing in MTCs and normal thyroid tissue samples (Supplementary Figure S1A), as well as MTC cell lines. A methylation index (MetI) was calculated for each sample analyzed as a mean of the eight CpGs assessed. Normal thyroid references exhibited low MetIs (mean 10.2%; range 5.7%-28%, Figure 2B). The MTC cell lines had very high levels of methylation with MetIs of 96% in MTC-TT and 96.5% in MZ-CRC-1. In the MTCs MetIs above 10% were observed in 37/39 sporadic MTCs and in 3/3 MEN2 related MTCs. Both the sporadic MTC group (*P* < 0.0001) and the MEN 2 group (*P* = 0.011) had increased *TERT* MetI as compared to normal thyroid (Figure 2B). In MTCs with increased methylation levels MetIs ranged from 12% to 90.3% (Supplementary Table S2). At the individual CpGs 1-8 methylation levels between 5% and 98% were observed without obvious difference between individual CpGs (Supplementary Figure S1B).

**Figure 3:**
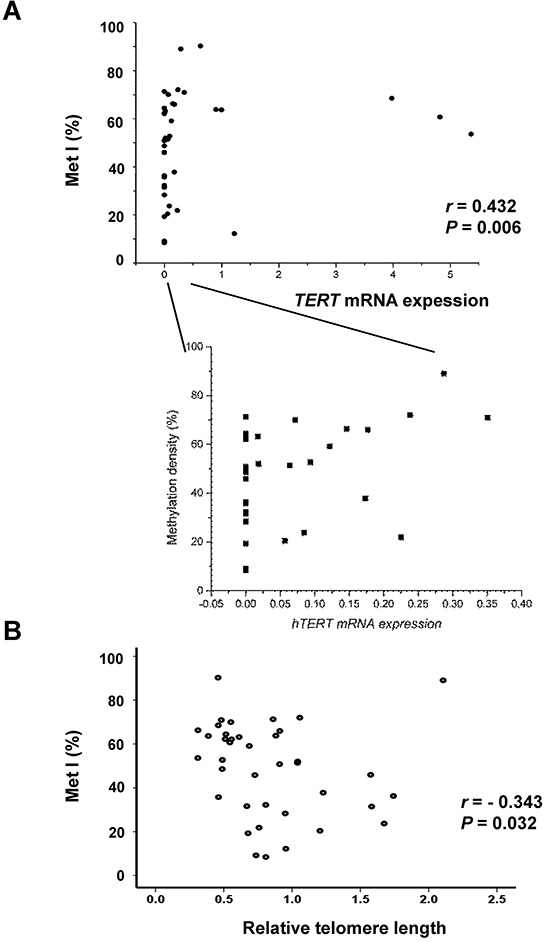
Comparisons of *TERT* promoter methylation index (Met I) with *TERT* gene expression and telomere length in the 39 sporadic MTCs **A.** The scatter diagram shows a positive correlation between MetI and *TERT* mRNA expression in sporadic MTCs. An enlargement to the right illustrates all cases with *TERT* mRNA expression within the lower range. **B.** Negative correlation between MetI and relative telomere length. Correlations were determined using Spearman rank order correlation. The values on the X-axes refer to *TERT* expression and telomere relative length values given in arbitrary units, respectively.

**Figure 4:**
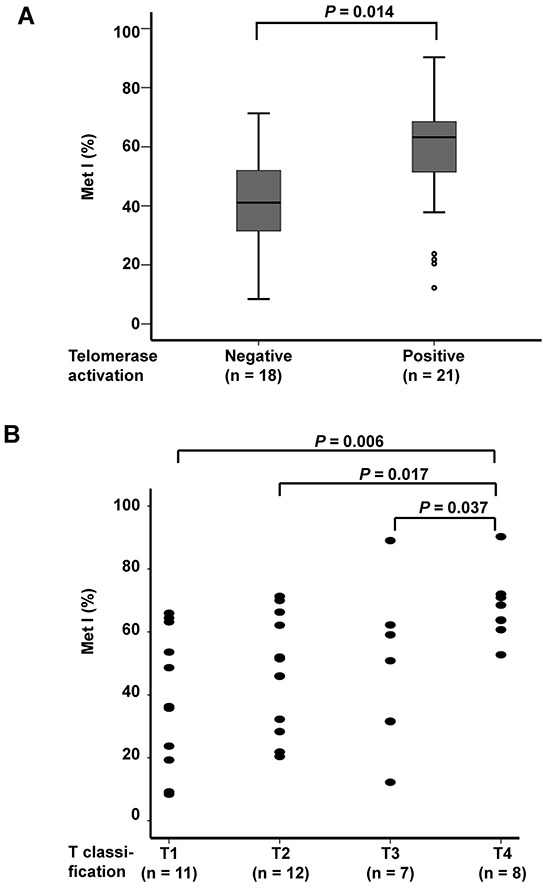
Comparison of *TERT* promoter MetI with telomerase activation status and tumor size of the 38 sporadic MTCs **A.** Box plots show higher *TERT* promoter MetIs in MTCs with telomerase activation as compared to telomerase negative MTCs. **B.** Scatter plot illustrating higher *TERT* promoter MetIs in MTCs with larger tumor size. T1 = ( < 20 mm), T2 = (20 mm-40 mm), T3 = ( > 40 mm but limited to thyroid OR with minimal extra-thyroidal extension), T4 = (tumors with extensive extra-thyroidal growth). Differences between groups were evaluated by Mann-Whitney U test. *P*-values are indicated for comparisons with statistically significant differences.

MetIs were compared with *TERT* expression, telomerase activation and telomere length previously determined for the same cases (7) (Supplementary Table S2). This revealed a positive correlation between MetI and *TERT* mRNA expression (*r* = 0.432, *P* = 0.006; Figure 3A), and a negative correlation was revealed between MetI and telomere length (*r* = −0.343, *P* = 0.032) (Figure 3B). MetIs were also significantly higher in MTCs with telomerase activation than in telomerase negative cases (*P* = 0.014) (Figure 4A; Table 1).

**Table 1:** Comparison of *TERT* promoter methylation density with clinical and molecular parameters in 39 sporadic MTC

Parameter informative cases (n = )	*TERT* methylation Met I (min.-max.) %	*P*-value
T classification (n = 38)		**0.026**
T1 (n = 11)	38 (8–66)	
T2 (n = 12)	47 (20–71)	
T3 (n = 7)	48 (12–89)	
T4 (n = 8)	68 (53–90)	
Stage (n = 39)		0.407
Stage I (n = 3)	36 (8–64)	
Stage II (n = 6)	46 (32–71)	
Stage III (n = 6)	40 (9–70)	
Stage IV (n = 24)	54 (12–90)	
Stage (n = 39)		0.100
Early-Stage (n = 15)	42 (8–71)	
Late-Stage (n = 24)	54 (12–90)	
*RET* mutation (n = 39)		0.167
Positive (n = 21)	54 (20–90)	
Negative (n = 18)	44 (8–89)	
*RAS* mutation (n = 39)		**0.031**
Positive (n = 7)	66 (51–89)	
Negative (n = 32)	45 (8–90)	
*RET* and /or *RAS* mutation (n = 39)		**0.002**
Positive (n = 27)	57 (20–90)	
Negative (n = 12)	32 (8–72)	
Telomerase activation (n = 39)		**0.014**
Positive (n = 21)	56 (12–90)	
Negative (n = 18)	41 (8–71)	
Tumor type (n = 38)		0.215
Primary tumor (n = 27)	52 (8–90)	
Metastasis (n = 11)	41 (9–66)	
Gender (n = 39)		0.862
Female (n = 24)	49 (8–89)	
Male (n = 15)	49 (9–90)	
Overall survival (n = 39)		0.039
Alive (n = 22)	44 (9–71)	
Dead (n = 17)	56 (8–90)	
Disease-related survival (n = 39)		0.053
Alive or dead from other diseases (n = 22 + 4)	45 (8–71)	
Dead of MTC (n = 13)	58 (12–90)	
Outcome (n = 39)		**0.144**
Free of disease (n = 12)	42 (9-71)	
Persistent disease (n = 27)	52 (8-90)	

T1 = (<20 mm); T2 = (20 mm-40 mm)

T3 = ( >40 mm and limited to thyroid OR with minimal extra-thyroidal extension)

T4 = (tumors with extensive extra-thyroidal growth)

Early-Stage = Stage I - Stage III ; Late-Stage = Stage IV

Significant and close to significant *P*-values are indicated in bold

Evaluation of MetIs in relation to *RAS* and *RET* mutation status revealed that MTCs with a *RAS* mutation displayed higher MetIs than *RAS* wild-type cases (*P* = 0.031) (Table 1). Similarly, MTCs with *RAS* and/or *RET* mutation had higher MetI than wild-type cases (*P* = 0.002). However, no statistically significant association was found between MetI and *RET* mutations.

### Increased *TERT* promoter methylation is associated with poor survival in MTC

Evaluation of MetIs in relation to clinical parameters revealed some significant associations (Table 1). MetIs were found to be higher in MTC cases with T4 tumors (tumors with extensive extra-thyroidal growth) as compared to T1 (*P* = 0.006), T2 (*P* = 0.017) and T3 (*P* = 0.037) tumors (Figure 4B). No significant differences were observed between groups with regard to gender or tumor stage (Table 1). Furthermore, no correlation was revealed between patient age and MetI values (*r* = 0.048; *P* = 0.772).

**Figure 5:**
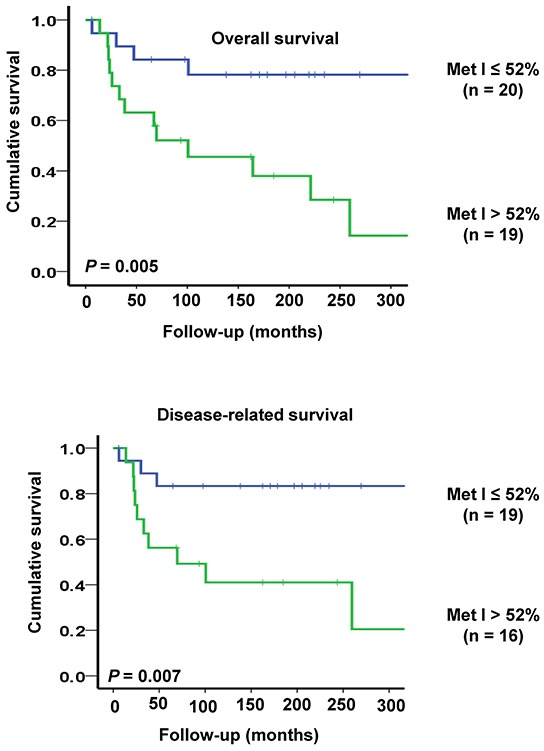
Kaplan-Meier plots illustrating survival of sporadic MTC cases in relation to *TERT* promoter methylation Patients with high MetI > 52% had shorter overall survival (top) and disease-related survival (below) as compared to cases with MetI ≤ 52%. The statistical analysis was performed using log-rank test.

For survival analyses sporadic MTC cases were divided into two groups according to the median MetI (52%). This cut-off gave an optimal combination of sensitivity and specificity for both overall survival (sensitivity: 0.76; specificity: 0.73) and disease-related survival (sensitivity: 0.77; specificity: 0.68). Among the 19 sporadic MTC cases with MetI > 52%, 10 died from MTC, three died from intercurrent disease, three were alive with disease and only three were alive without disease at the end of follow-up. In the group with MetI ≤ 52%, only three patients died from MTC, one died from intercurrent disease (lymphoma), seven were alive with disease and 9 patients were alive without disease. Using log rank test and Kaplan-Meier plots MetI > 52% was found to be associated with poor outcome. Patients with MetI > 52% showed shorter survival compared to patients with MetI ≤ 52% (*P* = 0.005 for overall survival and *P* = 0.007 for disease-related survival) (Figure 5).

### Protein expression profiles and pathway analysis in MTCs with and without telomerase activation

Fourteen fresh-frozen MTC samples were profiled by HiRIEF-LC-MS/MS including eight telomerase positive with different levels of *TERT* mRNA expression and six telomerase negative cases without detectable *TERT* mRNA expression. In total, 4,837 proteins were identified (1% FDR) in all samples, and the further data analyses were performed based on 4,321 proteins with quantitative data cross all tumors (Supplementary Table S3). Using PCA for visualization of the data no obvious grouping of MTCs could be observed (Supplementary Figure S2). Two samples were close to the 2-standard deviation line, which could be due to biological variation between cases (no. 26 and 19; Supplementary Table S1).

Different analytical approaches were taken to decipher the data, with the goal of finding proteins or groups of proteins that could distinguish the samples based on telomerase activation. Based on student's t-test 240 proteins were found to de differentially expressed between telomerase positive and negative MTCs (*P* < 0.05) (Supplementary Table S4). Among these 240 proteins, 101 showed higher expression in telomerase positive MTCs as compared to telomerase negative cases. Another 139 proteins had lower expression levels in telomerase positive cases, including e.g. CDKN1B (p27) which has been suggested to be a telomerase regulator [13].

**Figure 6:**
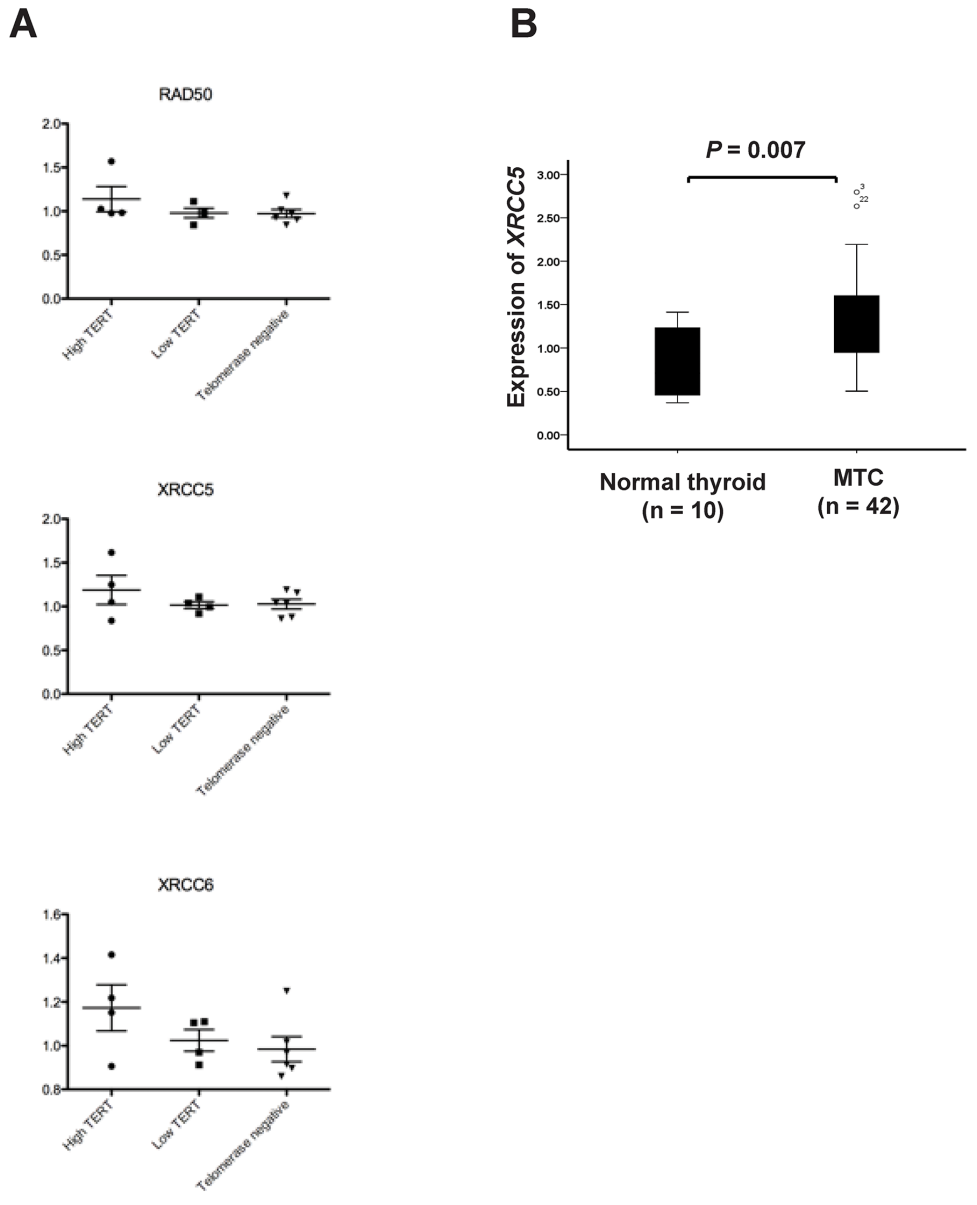
Expression of XRCC5 (Ku80), XRCC6 (Ku70) and RAD50 in sporadic MTCs **A.** Scatter plots showing relative protein expression levels of RAD50, XRCC5 and XRCC6 in 14 MTCs based on HiRIEF-LC-MS/MS data. Separate plots are shown for telomerase positive MTCs with high or low *TERT* mRNA expression as well as for telomerase negative MTCs. **B.** Relative mRNA expression of the *XRCC5* gene in 39 sporadic MTCs determined by qRT-PCR.

The OPLS analysis generated a model that could separate the eight MTCs with high versus low *TERT* expression levels (*P* = 0.002). The model was refined by ranking to define the most important proteins for the separation (Supplementary Table S5). These 93 proteins were then used to find enriched pathways employing the IPA tool (Table 2). The two top enriched canonical pathways identified were ‘DNA double-strand break repair by non-homologous end joining’ (*P* = 7×10^−5^) and ‘telomere extension by telomerase’ (*P* = 8×10^−5^). The same three proteins from our data were part of these two pathways, i.e. XRCC5 (Ku80) (gene), XRCC6 (Ku70) (gene) and RAD50. None of the three proteins were significantly differentially expressed on their own but showed high similarities in expression levels (Figure 6A).

**Table 2:** Canonical pathways identified according to *TERT* mRNA expression levels in 8 telomerase positive MTC

Comparison / Pathways identified	−log (*P*-value)	Ratio	Symbol
DNA double-strand break repair by non-homologous end joining	4.15E00	1.88E-01	XRCC6, XRCC5, RAD50
Telomere extension by telomerase	4.06E00	1.88E-01	XRCC6, XRCC5, RAD50
Mismatch repair in eukaryotes	3.97E00	1.5E-01	MSH2, MSH6, POLD1
Signaling by Rho family GTPases	3.54E00	2.85E-02	RELA, STMN1, WASL, CFL1, PIP4K2B, SLC9A1, MSN
Protein ubiquitination	2.78E00	2.28E-02	PSMA6, PSMA7, PSMA5, PSMA4, PSMA1, PSMA2
Mitochondrial L-carnitine shuttle	2.35E00	1.11E-01	ACSL3, CPT1A
Role of BRCA1 in DNA damage response	2.27E00	4.76E-02	MSH2, MSH6, RAD50
Actin cytoskeleton signaling	2.15E00	2.17E-02	WASL, CFL1, PIP4K2B, SLC9A1, MSN
Regulation of actin-based motility by Rho	1.98E00	3.49E-02	WASL, CFL1, PIP4K2B
RhoGDI signaling	1.82E00	2.13E-02	WASL, CFL1, PIP4K2B, MSN
Rac signaling	1.67E00	2.56E-02	RELA, CFL1, PIP4K2B
RhoA signaling	1.67E00	2.75E-02	CFL1,P IP4K2B, MSN
Pentose phosphate (oxidative branch)	1.63E00	2.5E-01	PGD
Hereditary breast cancer signaling	1.55E00	2.52E-02	MSH2, MSH6, RAD50
Glycine cleavage complex	1.45E00	1.67E-01	GCSH
LPS/IL-1 mediated inhibition of RXR function	1.44E00	1.8E-02	HS6ST1, ACSL3, CPT1A, GSTP1
Semaphorin signaling in neurons	1.42E00	3.85E-02	CFL1, DPYSL4
Acetyl-CoA biosynthesis I (pyruvate dehydrogenase complex)	1.39E00	1.43E-01	DLAT
ATM signaling	1.32E00	3.28E-02	TRIM28, RAD50

Pathways are based on analyses of 93 proteins from the OPLS model /Supplementary Table S5

The IPA tool was subsequently used to identify upstream regulators of the proteins in the data that have altered expression levels. For this purpose the 240 proteins identified as significantly changed by student's t-test (Supplementary Table S5) were analyzed regarding upstream regulators. This showed that FOXO1 was predicted as inhibited in the telomerase positive samples, based on the observed inhibition of a set of molecules such as STAT5B, NDUFAF1, CMC4, CDKN1B(p27) and BCL2.

### Increased expression of *XRCC5* in MTCs

*XRCC5,* which encodes the Ku80 subunit of the Ku heterodimer involved in DNA double strand break repair and has been associated with cancer development, was further analyzed in the MTC cohort. The mRNA expression of *XRCC5* was found to be increased in MTCs as compared to normal thyroid tissue (*P* = 0.007; Figure 6B).

## DISCUSSION

We have previously observed frequent telomerase activation and *TERT* expression in MTC tumors and identified *TERT*/telomerase as a prognostic factor in MTC patients [7]. In the present study, we further explored the mechanisms underlying these findings. To this end, we analyzed the *TERT* promoter methylation status and proteomic profiles, their relationship with *TERT* expression and clinical implications in MTCs.

The *TERT* promoter is highly GC-rich and in general unmethylated in normal human somatic cells irrespective of *TERT* expression status [12, 14]. In sharp contrast, *TERT*-expressing malignant cells exhibit promoter hypermethylation in certain regions [12, 13, 15]. It is believed that the acquisition of methylated CpGs in those specific regions leads to dissociation of *TERT* repressors from the *TERT* promoter, thereby contributing to the trans-activation of the *TERT* gene and subsequent telomerase activation in oncogenesis [12, 13, 15]. Indeed, we found significantly higher MetIs in MTC tumors than in normal thyroid tissues; and moreover, the score of MetIs was highly correlated with *TERT* mRNA and telomerase activity levels in MTCs. These results thus suggest that the hypermethylation of the *TERT* promoter plays an important part in the induction of *TERT* transcription and expression during MTC development, as seen in other human malignancies [12, 13, 15]. It may be argued that C-cells from which MTC is derived only constitute a minority of the normal thyroid cells and therefore a firm conclusion about increased MetI in MTC compared to its normal counterpart cannot be made. On the other hand, the observed low level of *TERT* promoter methylation in normal thyroids is in agreement with reports of several different types of non-cancer cells [12, 14].

The cancer-specific *TERT* promoter methylation has previously been tested for clinical significance [12, 13, 16]. However, most of these studies evaluated it as a cancer diagnostic biomarker. Only recently did Castelo-Branco and co-workers report that the *TERT* promoter methylation increased with cancer progression and could serve as a useful prognostic factor in posterior fossa ependymomas [12]. To see if this is also the case in MTCs, we divided the MTC patients into low and high MetI categories using the median score as a cut-off, and the result showed that a higher MetI was significantly associated with both shorter overall and disease-free survivals in MTC patients. Conceivably, higher MetIs might stimulate *TERT* expression and telomerase activation more potently, thereby contributing to more aggressive disease and shorter patient survival. It is currently unclear whether MetIs can act as a prognostic factor independently of *TERT* or telomerase activity, which calls for further clinical investigations in additional cohorts of MTC or other cancer patients. Furthermore, the correlation between MetI and *TERT* expression was not absolute, suggesting that additional mechanisms could also contribute to *TERT* expression such as transcriptional regulators with other binding sites or other epigenetic modifications.

As described above, the *TERT* promoter is not universally methylated in malignant cells. For instance, the CpG methylation is undetectable in the proximal *TERT* promoter (from −150 to +150 upstream of ATG) [17]. Furthermore, the methylated regions of the *TERT* promoter may differ among different types of cancer. The *TERT* promoter region that we chose for the present study was based on the result obtained from cancer cell lines [17]. Specifically, the interval at −550 bp from the ATG investigated here (Figure 1) is the same as the highly methylated region (included in BS-1) reported by Zinn *et al*. [17]. The two MTC cell lines studied here both showed very high MetIs at the same CpGs as investigated in the MTC tumors, supporting their relevance in cancers of different origins including thyroid C-cells. In addition, a region located down-stream in the *TERT* promoter was investigated in paediatric brain tumors and found to be hypermethylated [12]. Furthermore, in a region around the ATG of *TERT* low levels of methylation were shown to contribute to *TERT* expression [18].

Little is known about the mechanism underlying the *TERT* promoter hypermethylation in cancer. In the present study of MTCs, we noticed that MetIs were highly correlated with (i) the presence of *RAS* mutations, and (ii) shorter telomere length. Likely, oncogenic drivers promote target cells to over-proliferate; thereby leading to telomere over-erosion or crisis, and telomere crisis subsequently triggers both genomic catastrophic changes and telomerase activation. The *TERT* promoter hypermethylation may be mediated by genomic instability, and result in the dissociation of the specific repressors from the promoter, thereby derepressing *TERT* gene transcription and then activating telomerase.

In addition to the aberrant *TERT* promoter methylation, the *TERT* gene amplification has also been shown to frequently occur in many human malignancies and contribute to the up-regulation of *TERT* expression and telomerase activation [19, 20]. We thus determined whether the *TERT* copy number is altered in MTC tumors. Our analysis revealed that 5 of 39 (13%) MTC tumors carried 3 *TERT* copies, and all of them exhibited *TERT* expression and telomerase activation. It is not possible to evaluate their clinical significance due to too few patients. However, it may be noted that all 5 patients with increased *TERT* copy number presented with late stages of the disease, two of whom died of MTC and the remaining three had persistent disease. Taken together, the *TERT* copy number increase occurs in a subset of MTCs, and may be associated with *TERT* up-regulation and progressive disease.

As the *TERT* and telomerase regulation occurs at multiple levels, we further sought to characterize proteins related to and thus potentially responsible for *TERT* expression in MTCs. A total of 240 differentially expressed proteins were identified in group wide comparison of telomerase positive and negative MTCs tumors using a proteomic approach, among which 101 showed higher expression and 139 proteins had lower expression in telomerase positive tumors compared to those in the negative ones. Comparison with the protein expression profiles associated with *RET* and *RAS* mutation status, respectively, suggested that these are generally different from the proteins associated with telomerase activation (unpublished data). This would support that the 240 proteins identified were related to telomerase activation, and not merely a consequence of the underlying mutated drivers. On the other hand, *RAS* mutations could promote cellular over-proliferation at early stages when the *TERT* gene is still silent, leading to over-erosion of telomere length and subsequent telomere dysfunction/genomic instability. In *in vitro* immortalization models, it is well established that telomere dysfunction/genomic instability or catastrophe drives *TERT* transcription and telomerase activation, which is similar to that seen in *in vivo* settings. However, it remains unclear exactly how hypermethylation of the *TERT* promoter occur during *in vitro* cellular immortalization and *in vivo* malignant development, as well as how genomic catastrophe is linked to activation of *TERT* transcription. Among the proteins identified by upstream or pathway analyses, some such as CDKN1B (p27) and BCL2 are reported to regulate *TERT* transcription and telomerase activity [13]. While p27 inhibits telomerase activity and *TERT* expression [21, 22], BCL2 up-regulates telomerase activity [23]. It is currently unclear whether their effects on *TERT* expression are due to alterations in the methylation status of the *TERT* promoter, which calls for further detailed investigations.

Furthermore, FOXO1 was predicted as inhibited, and one of the functions of the FOXO1 transcription factor is to control expression of CDKN1B (p27), which in turn may inhibit *TERT* gene transcription.

The three proteins XRCC5 (Ku80), XRCC6 (Ku70) and RAD50 in the top enriched pathway “telomere extension by telomerase” have a role in telomere maintenance. However, the relationship between *TERT* regulation and most of these proteins remains to be defined, and further experimental studies are needed to elucidate this issue. Interestingly, XRCC5 (Ku80), XRCC6 (Ku70) and RAD50 are members of the non-homologous end joining (NHEJ) DNA repair machinery and are recurrently mutated genes in thyroid cancer according to the Catalogue of Somatic Mutations in Cancer (COSMIC) database. Moreover, another member of the NHEJ pathway, the mediator of DNA damage check point protein 1 gene (*MDC1)*, has been similarly found to be recurrently mutated in MTCs [11]. The observed association between telomerase activity and this pathway through XRCC5/6 and RAD50 thus further supports a role for deregulation of the NHEJ pathway in subsets of MTCs. Given that telomere dysfunction/genomic instability or catastrophe is a strong driving-force for *TERT* transcription and telomerase it is conceivable that NHEJ proteins may be directly or indirectly involved in genetic and epigenetic alterations of the *TERT* gene in MTC. The present study does not provide direct evidence for such a link. However, a recent publication did show that telomere dysfunction or DNA damage significantly alters DNA repair, chromatin remodeling, histone modification and other epigenetic landscapes in telomerase-knockout mice [24].

It should be pointed out that *TERT*, as the catalytic component of telomerase, not only catalyzes telomeric DNA synthesis, but also exhibits multiple telomere lengthening-independent activities [25, 26]. One of such *TERT* functions is to serve as a co-factor, promoting beta-catenin target gene transcription [25, 27]. Therefore, it is most likely that subsets of those 240 differentially expressed proteins observed between *TERT*-positive and negative tumors are due to the presence and absence of *TERT* expression. Nevertheless, the telomere lengthening-independent functions of *TERT* may also play important roles in cancer progression, which eventually leads to poor outcomes as seen in MTC and other cancer patients [25].

In summary, we show here that increased *TERT* copy number and *TERT* promoter hypermethylation are highly correlated with *TERT* expression in MTCs, indicating their contribution to telomerase activation; higher MetIs predict shorter overall and disease-free survival in MTC patients. Moreover, we identified differences in protein expression profiles between telomerase positive and negative MTC tumors, and their relationship with *TERT* expression remains to be defined. Collectively, the present findings not only provide insights into the mechanism underlying telomerase activation in MTCs, but also carry important clinical significance.

## MATERIALS AND METHODS

### MTC cell lines

MTC-TT was obtained from ATCC (LGC Standards GmhH, Germany), and MZ-CRC-1 was kindly provided by Professor Bruce Robinson and Professor Stan Sidhu, University of Sydney, Australia. Both cell lines were grown in DMEM with 10% fetal bovine serum (FBS), and verified by sequencing to carry *RET* mutations.

### Patients and clinical samples

The study includes 42 patients (39 sporadic and three MEN2) who were operated for MTC between 1986 and 2010 at the Karolinska University Hospital, Stockholm. The clinical information is provided in detail for each case in Supplementary Table S1. The diagnosis was established after routine histopathological examination following criteria of the WHO guidelines [8]. Ten histopathologically verified non-cancerous thyroid tissue samples from patients operated for a benign thyroid tumor were included as normal references. All tissue samples were retrieved from the Karolinska University Hospital Biobank. Informed consent was obtained from all patients and the study of the tissue samples was approved by the local ethics committee.

All 42 MTCs have previously been studied for C228T and C250T *TERT* promoter mutation, *TERT* expression, telomerase activity and telomere length, and a subset for the ALT phenotype (Supplementary Table S2) [4, 7]. In addition the mutation status for *RET* hot spot exons and *HRAS*, *NRAS* and *KRAS* hot spot codons have been performed (Wang et al. manuscript in preparation).

### DNA extraction

Genomic DNA was extracted using the DNeasy® Blood & Tissue kit (QIAGEN, Germany). Samples were quantified with a NanoDrop ND-100 spectrophotometer (Nano Drop Technologies, Wilmington, DE, USA) and stored at −80°C until use.

### DNA copy number assay

Copy numbers of the *TERT* gene locus were assessed in MTCs and normal thyroid tissues by a TaqMan-based assay. All samples were run in quadruplicate in a 96-well plate using commercially available assays for *TERT* (Hs 01237576_CN) and the endogenous control *RNase P* (4403326_CN). The results were normalized to the endogenous control followed by calibration to normal human reference DNA (Promega). *TERT* copy numbers were predicted using the CopyCaller software version 2.0 (Applied Biosystems, Foster City, CA, USA).

### Bisulfite pyrosequencing

Methylation density at the *TERT* promoter was quantified in MTCs, normal thyroid samples and MTC cell lines by Pyrosequencing. Genomic DNA (150 ng) was subjected to sodium bisulfate modification using the EpiTect Bisulfite Kit (Qiagen AB, Sweden) according to the recommendations of the manufacturer. Converted DNA was then used as template for PCR amplification at 58°C annealing temperature. A Pyrosequencing assay targeting eight CpGs in the *TERT* promoter (Figure 1) was designed using PyroMark Assay Design software version 2.0 (Qiagen). The assay included the following primers: 5′-GGGTTTGTGTTAAGGAGTTTAAGT-3′ (forward biotinylated); 5′-AAACCCAAAACTACCTCCA-3′ (reverse); and 5′-CCAAAACTACCTCCAAAT-3′ (sequencing). The PCR products were immobilized to streptavidin-coated Sepharose beads and were captured using a pump with filter. Pyrosequencing reactions were run in a PyroMark Q24 and the data were analysed with the PyroMark Q24 software (Qiagen AB, Sweden). For each sample, a methylation index (Met I) was calculated as the mean methylation level of all CpG dinucleotides covered [28]. The methylation status in MTCs was defined based on the comparison to the normal thyroid samples.

### Protein extraction and iTRAQ labeling for HiRIEF-LC-MS/MS

Fourteen MTCs (8 with telomerase activation and 6 without telomerase activation) were included in the HiRIEF-LC-MS/MS analysis essentially following previously published procedures [29]. Tumor samples were treated in sample buffer consisting of 4% SDS, 25 mM HEPES pH 7.6, 1 mM DTT followed by homogenizing with pestle. Protein extraction was performed by cell lysis at 90°C for 15 min, and sonication at room temperature to shear DNA and reduce viscosity. The lysates were cleared by centrifugation (10,000g for 20 min), and the supernatant containing the soluble protein fraction was transferred to a new tube. Protein concentrations were determined using Bio-Rad DCC. Extracted proteins were precipitated in four volumes of ice cold acetone to remove lysis buffer. Samples were kept on ice for one hour and centrifuged for 10 minutes at 4°C and 12,000g. Protein pellets were allowed to air dry, dissolved in 0.2% SDS and protein concentrations were determined again.

A protein aliquot from each sample was reduced and alkylated with iodoacetamide, and digested with trypsin. Peptides were then labeled with 8-plex iTRAQ (isobaric tags for relative and absolute quantitation). For standardization between the two sets, an internal standard consisting of a mixture of all 14 samples was labeled and included in both iTRAQ sets. Excess iTRAQ reagent and detergents were removed by strong cation exchange solid phase extraction (Strata X-C 33 μm polymeric SCX, Phenomenex). Purified samples were freeze dried in a SpeedVac system and placed in −80°C overnight.

### HiRIEF-LC-MS/MS analysis and protein identification

Protein samples were iTRAQ labeled and pooled samples were separated on both narrow range IPG strips (pH 3.7 – 4.9) and on ultra-narrow range IPG strips (pH 4.0 – 4.2). After separation, peptides were eluted from the gel strips into 72 fractions for each strip. These fractions were one by one subjected to reversed phase LC-MS/MS, where the peptides were fragmented to obtain the amino acid sequences. LC-MS was performed on a hybrid LTQ-Orbitrap Velos mass spectrometer (Thermo Fischer Scientific, San Jose, CA, USA). An Agilent HPLC 1200 system (Agilent Technologies, Santa Clara, CA, USA) was used for online reversed-phase nano-LC at a flow of 0.4 μl/min. Solvent A included 97 % water, 3 % ACN, and 0.1 % formic acid; and solvent B had 5 % water, 95 % ACN, and 0.1 % formic acid. The curved gradient went from 2 % B up to 40 % B in 45 min, followed by a steep increase to 100 % B in 5 min. Samples (3 (of 8) μl from each IPG fraction) were trapped on Zorbax 300SB-C18, 5 μm, 5 × 0.3 mm (Agilent Technologies, Santa Clara, CA, USA) and separated on a NTCC-360/100-5-153 C18 column (Nikkyo Technos Co., Tokyo, Japan) installed on to the nano electrospray ionisation (NSI) source of the Orbitrap Velos instrument. Acquisition proceeded in ∼3.5 s scan cycles, starting by a single full scan MS at 30000 resolution (profile mode), followed by two stages of data-dependent tandem MS (centroid mode): the top 5 ions from the full scan MS were selected firstly for collision induced dissociation (CID, at 35 % energy) with MS/MS detection in the ion trap, and finally for high energy collision dissociation (HCD, at 37.5 % energy) with MS/MS detection in the Orbitrap. Precursors were isolated with a 2 m/z width and dynamic exclusion was used with 60 s duration. The fragment spectra from the mass spectrometer were then matched to a database consisting of theoretical fragment spectra from all human proteins; this way the protein identities can be obtained. Quantitative information was acquired from the fragment spectra by using the iTRAQ reporter ion intensities.

### Proteomics data analysis

In total, 4,837 proteins (1% FDR on peptide level) were identified across the samples, with quantitative information retrieved for 4,321 proteins These 4,321 proteins were first assessed by principal component analysis (PCA) [30]. This method allows for detection of outliers and groupings among the samples. Protein levels were compared between telomerase positive and negative MTCs using student's t-test.

Further, multivariate data analyses were used to find groups of proteins that separate telomerase positive MTCs with different levels of *TERT* mRNA expression. These proteins will not necessarily be significant in univariate analysis comparing the two groups, but together they can form a panel of proteins that can classify tumors according to telomere activity status based on quantitative proteome data. This set of proteins can be used in downstream analysis to elucidate involved pathways or biological processes. For multivariate analysis orthogonal projections to latent structures (OPLS) [31] analysis was used as well as the commercial software package Ingenuity Pathway Analysis (IPA). For OPLS analysis, only the eight telomerase positive samples with different *TERT* mRNA levels were included to allow distinction of proteins important for the level of *TERT* mRNA expression. The proteins that distinguished MTCs with high versus low *TERT* expression in the OPLS analysis were analyzed with software in the Ingenuity Pathway Analysis (IPA) program to reveal connections to canonical pathways and find enriched pathways among the defined protein set.

### RNA extraction and reverse transcription-quantitative real-time PCR (qRT-PCR)

RNA was extracted from frozen tissue samples of MTCs and normal thyroids using the mirVana miRNA Isolation kit (Appied Biosystems), and cDNA was synthesized from RNA using High-Capacity cDNA Reverse Transcription kit (Invitrogen). qRT-PCR was carried out in an ABI 7900HT Real time PCR System (Applied Biosystems) using SYBR® Green PCR master mix (Invitrogen) for *XRCC5* (Hs00162669_m1) and *β-actin* as endogenous control. Expression levels of *XRCC5* mRNA were calculated based on the threshold cycle (Ct) values and normalization to human *β-actin* mRNA.

### Statistical analysis

The statistical analyses were performed with the SPSS software version 18.0 for windows. Differences between groups were evaluated by chi-square test (χ^2^) or Fisher's exact test (where appropriate) and Mann-Whitney U test. Spearman rank order correlation was performed to analyze correlation between *TERT* methylation, age, *TERT* expression, and telomere length. A cut-off of MetI 52% was selected based on the median value for the 39 sporadic MTCs and based on the optimal sensitivity and specificity. Survival curves were illustrated by Kaplan-Meier plots, and significance was calculated by log-rank test. *P*-values < 0.05 were regarded as statistically significant.

## SUPPLEMENTARY FIGURES AND TABLES












