# Supplementary material for: Genetic and epigenetic background and protein expression profiles in relation to telomerase activation in medullary thyroid carcinoma
Source: Oncotarget. 2016 Feb 8;7(16):21332–46. doi: 10.18632/oncotarget.7237 (PMC5008288; doi:10.18632/oncotarget.7237)
Supplement: Supplementary file 1 [file oncotarget-07-21332-s001.pdf]

SUPPLEMENTARY FIGURES AND TABLES

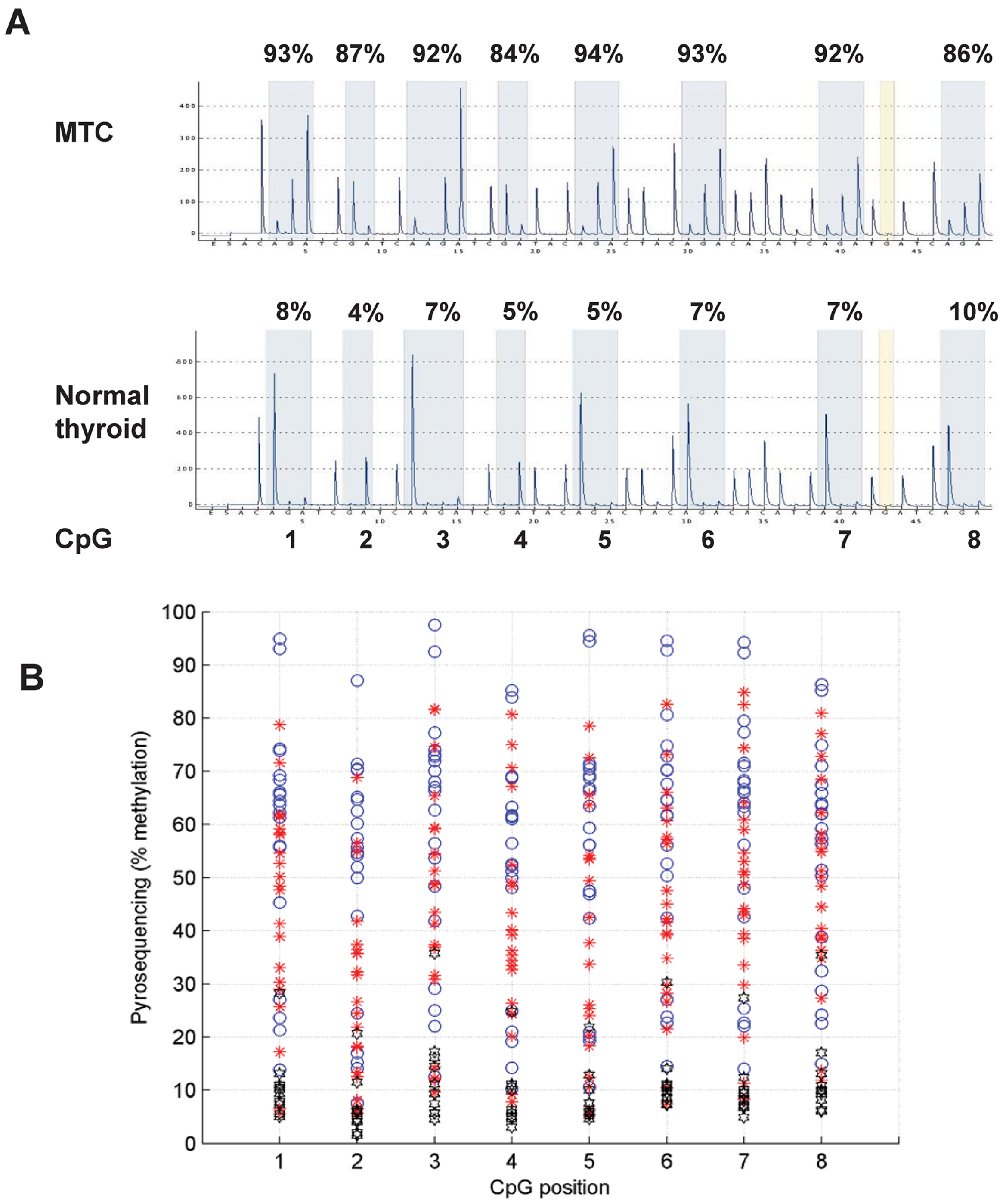

Supplementary Figure S1: Quantification of *TERT* promoter methylation at eight CpG sites by Pyrosequencing. A. Representative examples of Pyrograms showing increased methylation of the *TERT* promoter in an MTC as compared to a normal thyroid tissue sample. Methylation was quantified by Pyrosequencing over eight CpG sites and the degree of methylation is indicated on the top of each graph for each CpG site assessed. **B.** Scatter plot illustrating methylation density at CpG1–8 in telomerase positive MTCs (blue closed circles), telomerase negative MTCs (red stars) and normal thyroid (black hexagons).

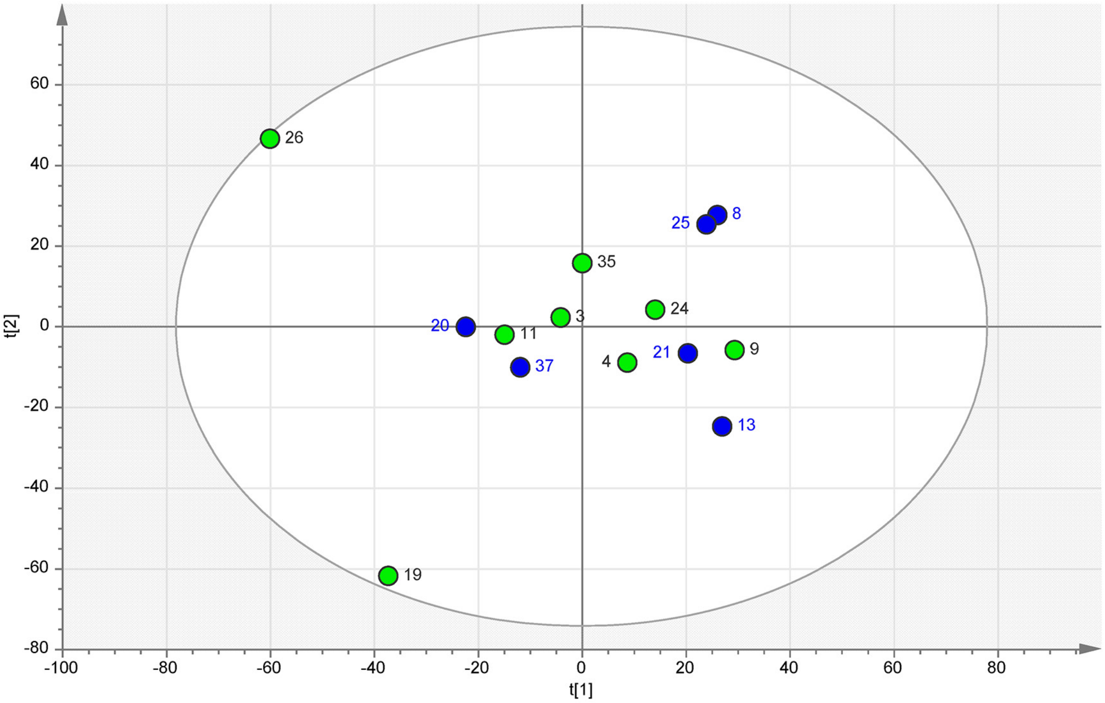

Supplementary Figure S2: Principal component analysis (PCA) of HiRIEF-LC-MS/MS data for 16 MTCs. Blue = telomerase negative, green = telomerase positive.

Supplementary Table S1: Clinical details of the 42 MTC cases
See Supplementary File 1

Supplementary Table S2: Details of *TERT* gene analysis, telomerase activation and telomere length in the 42 MTC
See Supplementary File 2

Supplementary Table S3: All 4,321 proteins identified and quantified in 14 MTCs by HiRIEF-LC-MS/MS.
See Supplementary File 3

Supplementary Table S4: Differentially expressed proteins by HiRIEF-LC-MS/MS between telomerase positive (n = 8) and negative (n = 6) MTCs
See Supplementary File 4

Supplementary Table S5: The 93 proteins identified in the OPLS model for telomerase positive MTCs (n =8) and used for pathway analysis.
See Supplementary File 5
