# Supplementary material for: Genetic and epigenetic background and protein expression profiles in relation to telomerase activation in medullary thyroid carcinoma
Source: Oncotarget. 2016 Feb 8;7(16):21332–46. doi: 10.18632/oncotarget.7237 (PMC5008288; doi:10.18632/oncotarget.7237)
Supplement: Supplementary file 2 [file oncotarget-07-21332-s002.doc]

| **Supplementary Table S1. Clinical details of the 42 MTC cases in the study.** | | | | | |  |  |  |
| --- | --- | --- | --- | --- | --- | --- | --- | --- |
| **Case** | **Gender** | **Age** |  |  | **Sample** | **Follow-up** | |  |
| **no.** | **M / F** | **years** | **TNM** | **Stage** | **analysed** | **years** | **outcome** |  |
| **Sporadic MTCs** | |  |  |  |  |  |  |  |
| 1 | F | 75 | T1bNxM0 | I | P | 8 | Dead | from other disease |
| 2 | M | 40 | T4N1M1 | IVC | P | 2 | Dead | DOD |
| 3 * | F | 65 | T4N1M0 | IVA | P | 6 | Dead | DOD |
| 4 * | F | 51 | T2N1MX | III | P | 22 | Dead | DOD |
| 5 | F | 46 | T1N1MX | III | M | 38 | Alive | AWD |
| 6 | M | 65 | T1N1MX | III | M | 27 | Alive | without disease |
| 7 | M | 35 | T2N1bM1 | IVC | P | 4 | Dead | DOD |
| 8 * | M | 61 | T1N1bMx | IVA | P | 22 | Alive | AWD |
| 9 * | F | 58 | T3N1bMx | IVA | P | 8 | Dead | DOD |
| 10 | M | 39 | T1N1bMX | IVA | M | 31 | Alive | AWD |
| 11 * | M | 76 | T4NxMx | IVA | P | 6 | Dead | from other disease |
| 12 | F | 73 | T2NxMx | II | P | 20 | Alive | without disease |
| 13 * | F | 69 | T2NxMx | II | P | 20 | Alive | without disease |
| 14 | M | 45 | T2N1bM0 | IVA | M | 30 | Alive | AWD |
| 15 | F | 61 | T3N0M0 | II | P | 19 | Alive | without disease |
| 16 | F | 58 | T3N1bMx | IVA | P | 18 | Alive | without disease |
| 17 | F | 37 | T2NxMx | II | P | 17 | Alive | without disease |
| 18 | F | 67 | T2N0M0 | II | ? | 16 | Alive | without disease |
| 19 * | F | 79 | T3N1M1 | IVC | M | 1 | Dead | DOD |
| 20 * | F | 54 | T1N0MX | I | P | 15 | Alive | without disease |
| 21 * | F | 72 | T3N1aM0 | III | P | 14 | Dead | from other disease |
| 22 | F | 87 | T3N1aM1 | IVC | P | 2 | Dead | DOD |
| 23 | F | 59 | T1N0M0 | I | P | 15 | Alive | without disease |
| 24 * | M | 63 | T1N1bM0 | IVA | P | 3 | Dead | DOD |
| 25 * | F | 41 | T1N1bM0 | IVA | P | 14 | Alive | AWD |
| 26 * | F | 13 | T4aN1bMx | IVA | P | 2 | Dead | DOD |
| 27 | M | 31 | T1N1bMX | IVA | M | 12 | Alive | AWD |
| 28 | M | 40 | T2N1bMX | IVA | P | 3 | Dead | DOD |
| 29 | F | 59 | T4N1bM0 | IVA | M | 14 | Alive | AWD |
| 30 | M | 51 | T2N1bMx | IVA | M | 18 | Dead | from other disease |
| 31 | F | 49 | T2N1aM0 | III | M | 14 | Alive | AWD |
| 32 | M | 57 | T4N1bM1 | IVC | P | 2 | Dead | DOD |
| 33 | F | 21 | T3N0M0 | II | P | 8 | Alive | without disease |
| 34 | M | 67 | T1N1bM0 | IVA | M | 8 | Alive | AWD |
| 35 * | F | 75 | T4aN1bMx | IVA | P | 1 | Dead | DOD |
| 36 | F | 46 | T4aN1bM1 | IVC | P | 3 | Dead | DOD |
| 37 * | M | 66 | T2N1bM0 | IVA | P | 6 | Alive | without disease |
| 38 | F | 73 | T2N1aM0 | III | P | 5 | Alive | without disease |
| 39 | M | 38 | T?N1bMx | IVA | M | 5 | Alive | AWD |
| ***MEN2 related MTCs*** | | |  |  |  |  |  |  |
| 40 | F | 28 | T3N1Mx | IVA | P | 25 | Alive | AWD |
| 41 | M | 14 | T?N0M0 |  | M | 37 | Alive | - |
| 42 | F | 54 | T1NxMx | I | P | 12 | Alive | without disease |
| M = Male; F = Female; DOD = Died of disease; AWD = Alive with disease | | | | | | |  |  |
| * Included in the HiRIEF-LC-MS/MS | | | |  |  |  |  |  |
|  |  |  |  |  |  |  |  |  |
