# Supplementary material for: Genetic and epigenetic background and protein expression profiles in relation to telomerase activation in medullary thyroid carcinoma
Source: Oncotarget. 2016 Feb 8;7(16):21332–46. doi: 10.18632/oncotarget.7237 (PMC5008288; doi:10.18632/oncotarget.7237)
Supplement: Supplementary file 3 [file oncotarget-07-21332-s003.doc]

| **Supplementary Table S2. Details of *TERT* gene analysis, telomerase activation and telomere length in the 42 MTC.** | | | | | | | |  |
| --- | --- | --- | --- | --- | --- | --- | --- | --- |
| **Case** | ***TERT* copy** | ***TERT* promoter methylation (%)** | | | ***TERT*** | **Telomerase** | **Telomere** |  |
| **no.*** | **number** | **Met I** | **Min.** | **Max.** | **expression *** | **activity *** | **length *** |  |
| ***Sporadic MTCs*** |  |  |  |  |  |  |  |  |
| 1 | 2 | 8.4 | 6 | 14 | 0 | 0 | 0.81 |  |
| 2 | 3 | 90.3 | 86 | 94 | 0.63 | 0.26 | 0.46 |  |
| 3 | 2 | 68.5 | 63 | 73 | 3.98 | 2.81 | 0.46 |  |
| 4 | 2 | 70.0 | 54 | 81 | 0.07 | 0.14 | 0.55 |  |
| 5^#^ | 2 | 19.3 | 6 | 26 | 0 | 0 | 0.68 |  |
| 6 | 2 | 9.2 | 5 | 12 | 0 | 0 | 0.74 |  |
| 7 | 2 | 20.4 | 14 | 24 | 0.06 | 0.24 | 1.20 |  |
| 8 | 2 | 36.3 | 18 | 45 | 0 | 0 | 1.74 |  |
| 9 | 2 | 59.1 | 48 | 70 | 0.12 | 0.05 | 0.69 |  |
| 10 | 3 | 65.9 | 62 | 72 | 0.18 | 0.26 | 0.91 |  |
| 11 | 3 | 63.8 | 51 | 77 | 0.90 | 3.25 | 0.88 |  |
| 12 | 2 | 71.3 | 67 | 75 | 0 | 0 | 0.86 |  |
| 13 | 2 | 32.2 | 14 | 57 | 0 | 0 | 0.81 |  |
| 14 | 2 | 51.5 | 47 | 56 | 0.06 | 0.001 | 1.04 |  |
| 15 | 2 | 31.6 | 12 | 55 | 0 | 0 | 0.67 |  |
| 16 | 2 | 31.5 | 13 | 39 | 0 | 0 | 1.58 |  |
| 17 | 2 | 46.0 | 22 | 66 | 0 | 0 | 1.58 |  |
| 18 | 2 | 45.9 | 32 | 59 | 0 | 0 | 0.73 |  |
| 19 | 2 | 12.2 | 7 | 15 | 1.22 | 3.18 | 0.95 |  |
| 20^#^ | 2 | 64.4 | 18 | 82 | 0 | 0 | 0.52 |  |
| 21^#^ | 2 | 62.2 | 42 | 85 | 0 | 0 | 0.51 |  |
| 22 | 2 | 89.0 | 65 | 98 | 0.29 | 0.35 | 2.11 |  |
| 23 | 2 | 35.8 | 8 | 49 | 0 | 0 | 0.46 |  |
| 24 | 2 | 63.2 | 43 | 69 | 0.02 | 0.02 | 0.61 |  |
| 25 | 2 | 48.6 | 36 | 58 | 0 | 0 | 0.49 |  |
| 26 | 2 | 52.7 | 32 | 66 | 0.09 | 0.30 | 0.49 |  |
| 27 | 2 | 23.7 | 14 | 29 | 0.08 | 0.65 | 1.67 |  |
| 28 | 2 | 21.8 | 17 | 27 | 0.23 | 0.51 | 0.76 |  |
| 29 | 3 | 63.6 | 57 | 68 | 1.00 | 2.80 | 0.39 |  |
| 30 | 2 | 66.3 | 53 | 74 | 0.15 | 0.41 | 0.31 |  |
| 31 | 2 | 52.0 | 67 | 74 | 0.02 | 0 | 1.04 |  |
| 32 | 2 | 70.9 | 36 | 66 | 0.35 | 1.46 | 0.48 |  |
| 33^#^ | 2 | 50.8 | 48 | 56 | 0 | 0 | 0.91 |  |
| 34 | 2 | 53.5 | 21 | 77 | 5.36 | 1.38 | 0.31 |  |
| 35 | 2 | 60.7 | 56 | 66 | 4.82 | 0.98 | 0.54 |  |
| 36 | 3 | 72.0 | 69 | 75 | 0.24 | 2.67 | 1.06 |  |
| 37^#^ | 2 | 62.1 | 26 | 82 | 0 | 0 | 0.56 |  |
| 38^#^ | 2 | 28.3 | 13 | 36 | 0 | 0 | 0.95 |  |
| 39 | 2 | 37.8 | 24 | 45 | 0.17 | 0.02 | 1.23 |  |
| ***MEN2 related MTCs*** | |  |  |  |  |  |  |  |
| 40 | 2 | 40.4 | 32 | 50 | 0 | 0 | 0.79 |  |
| 41 | 2 | 48.9 | 20 | 63 | 0 | 0 | 1.14 |  |
| 42 | 2 | 55.9 | 52 | 62 | 0 | 0 | 0.6 |  |
| ***Normal thyroid (n = 10)*** | |  |  |  |  |  |  |  |
|  | 2 | 10.2 | 1.5 | 35.6 | 0 | 0 | 1.36 |  |
| * Based on data in Wang et al. 2014; Met I = Mean methylation for CpG 1-8. | | | | |  |  |  |  |
| Min. = Lowest methylation density at an individual CpG; Max. = Highest methylation density at an individual CpG | | | | | | |  |  |
| # = case presenting the ALT phenotype | | | | | | |  |  |
|  |  |  |  |  |  |  |  |  |
