# Supplementary material for: Genetic and epigenetic background and protein expression profiles in relation to telomerase activation in medullary thyroid carcinoma
Source: Oncotarget. 2016 Feb 8;7(16):21332–46. doi: 10.18632/oncotarget.7237 (PMC5008288; doi:10.18632/oncotarget.7237)
Supplement: Supplementary file 4 [file oncotarget-07-21332-s004.doc]

| **Supplementary Table S3. All 4,321 proteins identified and quantified in 14 MTCs by HiRIEF-LC-MS/MS.** | | |  |
| --- | --- | --- | --- |
| **Accession** |  |  | **Fold change** |
| **no.** | **Symbol** | **Name** | **(telomerase pos/neg)** |
|  |  |  |  |
| P51692 | *STAT5B* | Signal transducer and activator of transcription 5B OS=Homo sapiens GN=STAT5B PE=1 SV=2 - [STA5B_HUMAN] | 0.694 |
| P55268 | *LAMB2* | Laminin subunit beta-2 OS=Homo sapiens GN=LAMB2 PE=1 SV=2 - [LAMB2_HUMAN] | 0.327 |
| P13987 | *CD59* | CD59 glycoprotein OS=Homo sapiens GN=CD59 PE=1 SV=1 - [CD59_HUMAN] | 0.386 |
| P48163 | *ME1* | NADP-dependent malic enzyme OS=Homo sapiens GN=ME1 PE=1 SV=1 - [MAOX_HUMAN] | 0.480 |
| P50579 | *METAP2* | Methionine aminopeptidase 2 OS=Homo sapiens GN=METAP2 PE=1 SV=1 - [AMPM2_HUMAN] | 0.805 |
| Q04837 | *SSBP1* | Single-stranded DNA-binding protein, mitochondrial OS=Homo sapiens GN=SSBP1 PE=1 SV=1 - [SSBP_HUMAN] | 0.750 |
| Q9Y2S2 | *CRYL1* | Isoform 2 of Lambda-crystallin homolog OS=Homo sapiens GN=CRYL1 - [CRYL1_HUMAN] | 0.562 |
| O95622 | *ADCY5* | Adenylate cyclase type 5 OS=Homo sapiens GN=ADCY5 PE=1 SV=3 - [ADCY5_HUMAN] | 0.525 |
| Q56VL3 | *OCIAD2* | Isoform 2 of OCIA domain-containing protein 2 OS=Homo sapiens GN=OCIAD2 - [OCAD2_HUMAN] | 1.584 |
| P49908 | *SEPP1* | Selenoprotein P OS=Homo sapiens GN=SEPP1 PE=1 SV=3 - [SEPP1_HUMAN] | 0.301 |
| P51178 | *PLCD1* | 1-phosphatidylinositol-4,5-bisphosphate phosphodiesterase delta-1 OS=Homo sapiens GN=PLCD1 PE=1 SV=2 - [PLCD1_HUMAN] | 0.564 |
| Q9NRW1 | *RAB6B* | Ras-related protein Rab-6B OS=Homo sapiens GN=RAB6B PE=1 SV=1 - [RAB6B_HUMAN] | 0.526 |
| P53396 | *ACLY* | Isoform 2 of ATP-citrate synthase OS=Homo sapiens GN=ACLY - [ACLY_HUMAN] | 0.558 |
| Q9NUJ1 | *ABHD10* | Abhydrolase domain-containing protein 10, mitochondrial OS=Homo sapiens GN=ABHD10 PE=1 SV=1 - [ABHDA_HUMAN] | 0.654 |
| Q96RL7 | *VPS13A* | Isoform 4 of Vacuolar protein sorting-associated protein 13A OS=Homo sapiens GN=VPS13A - [VP13A_HUMAN] | 0.768 |
| P00966 | *ASS1* | Argininosuccinate synthase OS=Homo sapiens GN=ASS1 PE=1 SV=2 - [ASSY_HUMAN] | 0.315 |
| P08571 | *CD14* | Monocyte differentiation antigen CD14 OS=Homo sapiens GN=CD14 PE=1 SV=2 - [CD14_HUMAN] | 0.631 |
| Q9H3G5 | *CPVL* | Probable serine carboxypeptidase CPVL OS=Homo sapiens GN=CPVL PE=1 SV=2 - [CPVL_HUMAN] | 0.568 |
| O00629 | *KPNA4* | Importin subunit alpha-4 OS=Homo sapiens GN=KPNA4 PE=1 SV=1 - [IMA4_HUMAN] | 0.809 |
| P46527 | *CDKN1B* | Cyclin-dependent kinase inhibitor 1B OS=Homo sapiens GN=CDKN1B PE=1 SV=1 - [CDN1B_HUMAN] | 0.734 |
| O15230 | *LAMA5* | Laminin subunit alpha-5 OS=Homo sapiens GN=LAMA5 PE=1 SV=8 - [LAMA5_HUMAN] | 0.330 |
| Q8N9F7 | *GDPD1* | Isoform 3 of Glycerophosphodiester phosphodiesterase domain-containing protein 1 OS=Homo sapiens GN=GDPD1 - [GDPD1_HUMAN] | 1.634 |
| O43264 | *ZW10* | Centromere/kinetochore protein zw10 homolog OS=Homo sapiens GN=ZW10 PE=1 SV=3 - [ZW10_HUMAN] | 1.357 |
| P28289 | *TMOD1* | Tropomodulin-1 OS=Homo sapiens GN=TMOD1 PE=1 SV=1 - [TMOD1_HUMAN] | 0.647 |
| Q9BX67 | *JAM3* | Junctional adhesion molecule C OS=Homo sapiens GN=JAM3 PE=1 SV=1 - [JAM3_HUMAN] | 0.519 |
| P21926 | *CD9* | CD9 antigen OS=Homo sapiens GN=CD9 PE=1 SV=4 - [CD9_HUMAN] | 0.427 |
| Q9NRL3 | *STRN4* | Striatin-4 OS=Homo sapiens GN=STRN4 PE=1 SV=2 - [STRN4_HUMAN] | 1.289 |
| P49748 | *ACADVL* | Isoform 2 of Very long-chain specific acyl-CoA dehydrogenase, mitochondrial OS=Homo sapiens GN=ACADVL - [ACADV_HUMAN] | 0.649 |
| Q6ZMZ3 | *SYNE3* | Isoform 2 of Nesprin-3 OS=Homo sapiens GN=C14orf49 - [SYNE3_HUMAN] | 0.558 |
| Q9H074 | *PAIP1* | Isoform 2 of Polyadenylate-binding protein-interacting protein 1 OS=Homo sapiens GN=PAIP1 - [PAIP1_HUMAN] | 1.407 |
| P42574 | *CASP3* | Caspase-3 OS=Homo sapiens GN=CASP3 PE=1 SV=2 - [CASP3_HUMAN] | 1.384 |
| Q6IPR1 | *LYRM5* | LYR motif-containing protein 5 OS=Homo sapiens GN=LYRM5 PE=2 SV=1 - [LYRM5_HUMAN] | 0.623 |
| Q96KA5 | *CLPTM1L* | Isoform 2 of Cleft lip and palate transmembrane protein 1-like protein OS=Homo sapiens GN=CLPTM1L - [CLP1L_HUMAN] | 1.903 |
| O95470 | *SGPL1* | Sphingosine-1-phosphate lyase 1 OS=Homo sapiens GN=SGPL1 PE=1 SV=3 - [SGPL1_HUMAN] | 2.599 |
| Q9H223 | *EHD4* | EH domain-containing protein 4 OS=Homo sapiens GN=EHD4 PE=1 SV=1 - [EHD4_HUMAN] | 0.679 |
| P10909 | *CLU* | Isoform 4 of Clusterin OS=Homo sapiens GN=CLU - [CLUS_HUMAN] | 0.279 |
| P30626 | *SRI* | Sorcin OS=Homo sapiens GN=SRI PE=1 SV=1 - [SORCN_HUMAN] | 0.712 |
| P63313 | *TMSB10* | Thymosin beta-10 OS=Homo sapiens GN=TMSB10 PE=1 SV=2 - [TYB10_HUMAN] | 1.603 |
| Q5VYK3 | *KIAA0368* | Proteasome-associated protein ECM29 homolog OS=Homo sapiens GN=ECM29 PE=1 SV=2 - [ECM29_HUMAN] | 1.225 |
| O75976 | *CPD* | Carboxypeptidase D OS=Homo sapiens GN=CPD PE=1 SV=2 - [CBPD_HUMAN] | 1.812 |
| Q9H845 | *ACAD9* | Acyl-CoA dehydrogenase family member 9, mitochondrial OS=Homo sapiens GN=ACAD9 PE=1 SV=1 - [ACAD9_HUMAN] | 0.658 |
| O00186 | *STXBP3* | Syntaxin-binding protein 3 OS=Homo sapiens GN=STXBP3 PE=1 SV=2 - [STXB3_HUMAN] | 0.759 |
| P51149 | *RAB7A* | Ras-related protein Rab-7a OS=Homo sapiens GN=RAB7A PE=1 SV=1 - [RAB7A_HUMAN] | 0.797 |
| Q15047 | *SETDB1* | Isoform 2 of Histone-lysine N-methyltransferase SETDB1 OS=Homo sapiens GN=SETDB1 - [SETB1_HUMAN] | 1.267 |
| Q9NY15 | *STAB1* | Stabilin-1 OS=Homo sapiens GN=STAB1 PE=1 SV=3 - [STAB1_HUMAN] | 0.497 |
| P39019 | *RPS19* | 40S ribosomal protein S19 OS=Homo sapiens GN=RPS19 PE=1 SV=2 - [RS19_HUMAN] | 1.178 |
| Q6VY07 | *PACS1* | Phosphofurin acidic cluster sorting protein 1 OS=Homo sapiens GN=PACS1 PE=1 SV=2 - [PACS1_HUMAN] | 1.342 |
| Q2NL82 | *TSR1* | Pre-rRNA-processing protein TSR1 homolog OS=Homo sapiens GN=TSR1 PE=1 SV=1 - [TSR1_HUMAN] | 1.191 |
| Q16822 | *PCK2* | Phosphoenolpyruvate carboxykinase [GTP], mitochondrial OS=Homo sapiens GN=PCK2 PE=1 SV=3 - [PCKGM_HUMAN] | 0.777 |
| Q9H3H9 | *TCEAL2* | Transcription elongation factor A protein-like 2 OS=Homo sapiens GN=TCEAL2 PE=2 SV=1 - [TCAL2_HUMAN] | 0.357 |
| P15153 | *RAC2* | Ras-related C3 botulinum toxin substrate 2 OS=Homo sapiens GN=RAC2 PE=1 SV=1 - [RAC2_HUMAN] | 0.871 |
| Q6ZN30 | *BNC2* | Isoform 2 of Zinc finger protein basonuclin-2 OS=Homo sapiens GN=BNC2 - [BNC2_HUMAN] | 0.387 |
| Q9UBS3 | *DNAJB9* | DnaJ homolog subfamily B member 9 OS=Homo sapiens GN=DNAJB9 PE=1 SV=1 - [DNJB9_HUMAN] | 0.267 |
| Q5T3F8 | *TMEM63B* | Transmembrane protein 63B OS=Homo sapiens GN=TMEM63B PE=1 SV=1 - [TM63B_HUMAN] | 1.545 |
| Q9UBW8 | *COPS7A* | COP9 signalosome complex subunit 7a OS=Homo sapiens GN=COPS7A PE=1 SV=1 - [CSN7A_HUMAN] | 0.780 |
| P22830 | *FECH* | Ferrochelatase, mitochondrial OS=Homo sapiens GN=FECH PE=1 SV=2 - [HEMH_HUMAN] | 0.651 |
| P41208 | *CETN2* | Centrin-2 OS=Homo sapiens GN=CETN2 PE=1 SV=1 - [CETN2_HUMAN] | 0.791 |
| P10768 | *ESD* | S-formylglutathione hydrolase OS=Homo sapiens GN=ESD PE=1 SV=2 - [ESTD_HUMAN] | 0.756 |
| P23634 | *ATP2B4* | Isoform ZB of Plasma membrane calcium-transporting ATPase 4 OS=Homo sapiens GN=ATP2B4 - [AT2B4_HUMAN] | 0.728 |
| P30084 | *ECHS1* | Enoyl-CoA hydratase, mitochondrial OS=Homo sapiens GN=ECHS1 PE=1 SV=4 - [ECHM_HUMAN] | 0.714 |
| P04899 | *GNAI2* | Isoform 2 of Guanine nucleotide-binding protein G(i) subunit alpha-2 OS=Homo sapiens GN=GNAI2 - [GNAI2_HUMAN] | 0.716 |
| P09936 | *UCHL1* | Ubiquitin carboxyl-terminal hydrolase isozyme L1 OS=Homo sapiens GN=UCHL1 PE=1 SV=2 - [UCHL1_HUMAN] | 0.528 |
| Q02083 | *NAAA* | Isoform 2 of N-acylethanolamine-hydrolyzing acid amidase OS=Homo sapiens GN=NAAA - [NAAA_HUMAN] | 0.700 |
| P26440 | *IVD* | Isovaleryl-CoA dehydrogenase, mitochondrial OS=Homo sapiens GN=IVD PE=1 SV=1 - [IVD_HUMAN] | 0.703 |
| O00178 | *GTPBP1* | GTP-binding protein 1 OS=Homo sapiens GN=GTPBP1 PE=1 SV=3 - [GTPB1_HUMAN] | 1.240 |
| Q8NFW8 | *CMAS* | N-acylneuraminate cytidylyltransferase OS=Homo sapiens GN=CMAS PE=1 SV=2 - [NEUA_HUMAN] | 0.656 |
| P20337 | *RAB3B* | Ras-related protein Rab-3B OS=Homo sapiens GN=RAB3B PE=1 SV=2 - [RAB3B_HUMAN] | 4.044 |
| Q9UF11 | *PLEKHB1* | Isoform 4 of Pleckstrin homology domain-containing family B member 1 OS=Homo sapiens GN=PLEKHB1 - [PKHB1_HUMAN] | 0.559 |
| Q96RQ3 | *MCCC1* | Methylcrotonoyl-CoA carboxylase subunit alpha, mitochondrial OS=Homo sapiens GN=MCCC1 PE=1 SV=3 - [MCCA_HUMAN] | 0.642 |
| Q15819 | *UBE2V2* | Ubiquitin-conjugating enzyme E2 variant 2 OS=Homo sapiens GN=UBE2V2 PE=1 SV=4 - [UB2V2_HUMAN] | 1.195 |
| Q9BQE5 | *APOL2* | Apolipoprotein L2 OS=Homo sapiens GN=APOL2 PE=1 SV=1 - [APOL2_HUMAN] | 1.355 |
| Q8NI22 | *MCFD2* | Multiple coagulation factor deficiency protein 2 OS=Homo sapiens GN=MCFD2 PE=1 SV=1 - [MCFD2_HUMAN] | 0.773 |
| Q9UFG5 | *C19orf25* | UPF0449 protein C19orf25 OS=Homo sapiens GN=C19orf25 PE=1 SV=2 - [CS025_HUMAN] | 1.330 |
| Q8TEW0 | *PARD3* | Isoform 9 of Partitioning defective 3 homolog OS=Homo sapiens GN=PARD3 - [PARD3_HUMAN] | 1.343 |
| Q96F85 | *CNRIP1* | Isoform 2 of CB1 cannabinoid receptor-interacting protein 1 OS=Homo sapiens GN=CNRIP1 - [CNRP1_HUMAN] | 0.439 |
| Q86V97 | *KBTBD6* | Kelch repeat and BTB domain-containing protein 6 OS=Homo sapiens GN=KBTBD6 PE=1 SV=1 - [KBTB6_HUMAN] | 0.588 |
| Q9Y496 | *KIF3A* | Kinesin-like protein KIF3A OS=Homo sapiens GN=KIF3A PE=1 SV=4 - [KIF3A_HUMAN] | 1.256 |
| Q9BVL2 | *NUPL1* | Isoform 2 of Nucleoporin p58/p45 OS=Homo sapiens GN=NUPL1 - [NUPL1_HUMAN] | 1.192 |
| O14974 | *PPP1R12A* | Isoform 4 of Protein phosphatase 1 regulatory subunit 12A OS=Homo sapiens GN=PPP1R12A - [MYPT1_HUMAN] | 0.816 |
| Q9Y5K5 | *UCHL5* | Isoform 2 of Ubiquitin carboxyl-terminal hydrolase isozyme L5 OS=Homo sapiens GN=UCHL5 - [UCHL5_HUMAN] | 1.376 |
| P29401 | *TKT* | Transketolase OS=Homo sapiens GN=TKT PE=1 SV=3 - [TKT_HUMAN] | 0.713 |
| P29966 | *MARCKS* | Myristoylated alanine-rich C-kinase substrate OS=Homo sapiens GN=MARCKS PE=1 SV=4 - [MARCS_HUMAN] | 1.399 |
| P11047 | *LAMC1* | Laminin subunit gamma-1 OS=Homo sapiens GN=LAMC1 PE=1 SV=3 - [LAMC1_HUMAN] | 0.487 |
| Q9BUF7 | *CRB3* | Crumbs protein homolog 3 OS=Homo sapiens GN=CRB3 PE=1 SV=3 - [CRUM3_HUMAN] | 0.541 |
| P34949 | *MPI* | Isoform 2 of Mannose-6-phosphate isomerase OS=Homo sapiens GN=MPI - [MPI_HUMAN] | 1.269 |
| P20674 | *COX5A* | Cytochrome c oxidase subunit 5A, mitochondrial OS=Homo sapiens GN=COX5A PE=1 SV=2 - [COX5A_HUMAN] | 0.815 |
| Q8N475 | *FSTL5* | Follistatin-related protein 5 OS=Homo sapiens GN=FSTL5 PE=2 SV=2 - [FSTL5_HUMAN] | 0.418 |
| Q3L8U1 | *CHD9* | Isoform 2 of Chromodomain-helicase-DNA-binding protein 9 OS=Homo sapiens GN=CHD9 - [CHD9_HUMAN] | 1.403 |
| Q8NEY1 | *NAV1* | Isoform 5 of Neuron navigator 1 OS=Homo sapiens GN=NAV1 - [NAV1_HUMAN] | 1.303 |
| Q86VM9 | *ZC3H18* | Zinc finger CCCH domain-containing protein 18 OS=Homo sapiens GN=ZC3H18 PE=1 SV=2 - [ZCH18_HUMAN] | 1.266 |
| O75506 | *HSBP1* | Heat shock factor-binding protein 1 OS=Homo sapiens GN=HSBP1 PE=1 SV=1 - [HSBP1_HUMAN] | 1.470 |
| Q7Z422 | *SZRD1* | Isoform 2 of UPF0485 protein C1orf144 OS=Homo sapiens GN=C1orf144 - [CA144_HUMAN] | 1.210 |
| Q32P28 | *P3H1* | Prolyl 3-hydroxylase 1 OS=Homo sapiens GN=LEPRE1 PE=1 SV=2 - [P3H1_HUMAN] | 1.600 |
| P53582 | *METAP1* | Methionine aminopeptidase 1 OS=Homo sapiens GN=METAP1 PE=1 SV=2 - [AMPM1_HUMAN] | 1.274 |
| A0AV96 | *RBM47* | Isoform 2 of RNA-binding protein 47 OS=Homo sapiens GN=RBM47 - [RBM47_HUMAN] | 0.768 |
| Q9Y6X9 | *MORC2* | Isoform 2 of MORC family CW-type zinc finger protein 2 OS=Homo sapiens GN=MORC2 - [MORC2_HUMAN] | 1.377 |
| Q9UBL3 | *ASH2L* | Isoform 2 of Set1/Ash2 histone methyltransferase complex subunit ASH2 OS=Homo sapiens GN=ASH2L - [ASH2L_HUMAN] | 1.305 |
| P05067 | *APP* | Isoform L-APP733 of Amyloid beta A4 protein OS=Homo sapiens GN=APP - [A4_HUMAN] | 1.771 |
| O43583 | *DENR* | Density-regulated protein OS=Homo sapiens GN=DENR PE=1 SV=2 - [DENR_HUMAN] | 1.277 |
| Q9UL12 | *SARDH* | Sarcosine dehydrogenase, mitochondrial OS=Homo sapiens GN=SARDH PE=1 SV=1 - [SARDH_HUMAN] | 0.546 |
| P20042 | *EIF2S2* | Eukaryotic translation initiation factor 2 subunit 2 OS=Homo sapiens GN=EIF2S2 PE=1 SV=2 - [IF2B_HUMAN] | 1.208 |
| O95630 | *STAMBP* | STAM-binding protein OS=Homo sapiens GN=STAMBP PE=1 SV=1 - [STABP_HUMAN] | 1.128 |
| P46926 | *GNPDA1* | Glucosamine-6-phosphate isomerase 1 OS=Homo sapiens GN=GNPDA1 PE=1 SV=1 - [GNPI1_HUMAN] | 0.810 |
| Q9Y281 | *CFL2* | Cofilin-2 OS=Homo sapiens GN=CFL2 PE=1 SV=1 - [COF2_HUMAN] | 0.684 |
| Q9P2X0 | *DPM3* | Dolichol-phosphate mannosyltransferase subunit 3 OS=Homo sapiens GN=DPM3 PE=1 SV=2 - [DPM3_HUMAN] | 1.281 |
| Q96NT1 | *NAP1L5* | Nucleosome assembly protein 1-like 5 OS=Homo sapiens GN=NAP1L5 PE=2 SV=1 - [NP1L5_HUMAN] | 0.619 |
| P24752 | *ACAT1* | Acetyl-CoA acetyltransferase, mitochondrial OS=Homo sapiens GN=ACAT1 PE=1 SV=1 - [THIL_HUMAN] | 0.765 |
| Q8WW59 | *SPRYD4* | SPRY domain-containing protein 4 OS=Homo sapiens GN=SPRYD4 PE=1 SV=2 - [SPRY4_HUMAN] | 0.745 |
| O43719 | *HTATSF1* | HIV Tat-specific factor 1 OS=Homo sapiens GN=HTATSF1 PE=1 SV=1 - [HTSF1_HUMAN] | 0.658 |
| P42765 | *ACAA2* | 3-ketoacyl-CoA thiolase, mitochondrial OS=Homo sapiens GN=ACAA2 PE=1 SV=2 - [THIM_HUMAN] | 0.685 |
| Q96RS6 | *NUDCD1* | Isoform 3 of NudC domain-containing protein 1 OS=Homo sapiens GN=NUDCD1 - [NUDC1_HUMAN] | 1.199 |
| Q9H8H3 | *METTL7A* | Methyltransferase-like protein 7A OS=Homo sapiens GN=METTL7A PE=1 SV=1 - [MET7A_HUMAN] | 0.604 |
| Q8N9T8 | *KRI1* | Isoform 2 of Protein KRI1 homolog OS=Homo sapiens GN=KRI1 - [KRI1_HUMAN] | 0.766 |
| Q8NFP9 | *NBEA* | Neurobeachin OS=Homo sapiens GN=NBEA PE=1 SV=3 - [NBEA_HUMAN] | 0.707 |
| Q9UDR5 | *AASS* | Alpha-aminoadipic semialdehyde synthase, mitochondrial OS=Homo sapiens GN=AASS PE=1 SV=1 - [AASS_HUMAN] | 0.398 |
| Q8NEJ9 | *NGDN* | Isoform 2 of Neuroguidin OS=Homo sapiens GN=NGDN - [NGDN_HUMAN] | 1.421 |
| P09960 | *LTA4H* | Leukotriene A-4 hydrolase OS=Homo sapiens GN=LTA4H PE=1 SV=2 - [LKHA4_HUMAN] | 0.744 |
| Q15147 | *PLCB4* | Isoform 3 of 1-phosphatidylinositol-4,5-bisphosphate phosphodiesterase beta-4 OS=Homo sapiens GN=PLCB4 - [PLCB4_HUMAN] | 1.727 |
| P14543 | *NID1* | Nidogen-1 OS=Homo sapiens GN=NID1 PE=1 SV=3 - [NID1_HUMAN] | 0.555 |
| A8MW06 | *TMSL3* | Thymosin beta-4-like protein 3 OS=Homo sapiens GN=TMSL3 PE=2 SV=1 - [TMSL3_HUMAN] | 1.946 |
| Q14344 | *GNA13* | Guanine nucleotide-binding protein subunit alpha-13 OS=Homo sapiens GN=GNA13 PE=1 SV=2 - [GNA13_HUMAN] | 0.751 |
| A6NIH7 | *UNC119B* | Protein unc-119 homolog B OS=Homo sapiens GN=UNC119B PE=1 SV=1 - [U119B_HUMAN] | 0.814 |
| Q9NRV9 | *HEBP1* | Heme-binding protein 1 OS=Homo sapiens GN=HEBP1 PE=1 SV=1 - [HEBP1_HUMAN] | 0.532 |
| Q96EY8 | *MMAB* | Cob(I)yrinic acid a,c-diamide adenosyltransferase, mitochondrial OS=Homo sapiens GN=MMAB PE=1 SV=1 - [MMAB_HUMAN] | 0.831 |
| Q9UBI6 | *GNG12* | Guanine nucleotide-binding protein G(I)/G(S)/G(O) subunit gamma-12 OS=Homo sapiens GN=GNG12 PE=1 SV=3 - [GBG12_HUMAN] | 0.726 |
| P78356 | *PIP4K2B* | Phosphatidylinositol-5-phosphate 4-kinase type-2 beta OS=Homo sapiens GN=PIP4K2B PE=1 SV=1 - [PI42B_HUMAN] | 0.806 |
| Q9NQR4 | *NIT2* | Omega-amidase NIT2 OS=Homo sapiens GN=NIT2 PE=1 SV=1 - [NIT2_HUMAN] | 0.793 |
| P35858 | *IGFALS* | Insulin-like growth factor-binding protein complex acid labile subunit OS=Homo sapiens GN=IGFALS PE=1 SV=1 - [ALS_HUMAN] | 0.495 |
| Q9UBF2 | *COPG2* | Coatomer subunit gamma-2 OS=Homo sapiens GN=COPG2 PE=1 SV=1 - [COPG2_HUMAN] | 1.408 |
| Q8WU76 | *SCFD2* | Isoform 2 of Sec1 family domain-containing protein 2 OS=Homo sapiens GN=SCFD2 - [SCFD2_HUMAN] | 1.365 |
| P23528 | *CFL1* | Cofilin-1 OS=Homo sapiens GN=CFL1 PE=1 SV=3 - [COF1_HUMAN] | 1.354 |
| P14543 | *NID1* | Isoform 2 of Nidogen-1 OS=Homo sapiens GN=NID1 - [NID1_HUMAN] | 0.496 |
| P09486 | *SPARC* | SPARC OS=Homo sapiens GN=SPARC PE=1 SV=1 - [SPRC_HUMAN] | 1.740 |
| Q86YR5 | *GPSM1* | Isoform 4 of G-protein-signaling modulator 1 OS=Homo sapiens GN=GPSM1 - [GPSM1_HUMAN] | 1.404 |
| P53041 | *PPP5C* | Serine/threonine-protein phosphatase 5 OS=Homo sapiens GN=PPP5C PE=1 SV=1 - [PPP5_HUMAN] | 1.294 |
| P17600 | *SYN1* | Isoform IB of Synapsin-1 OS=Homo sapiens GN=SYN1 - [SYN1_HUMAN] | 0.525 |
| O15355 | *PPM1G* | Protein phosphatase 1G OS=Homo sapiens GN=PPM1G PE=1 SV=1 - [PPM1G_HUMAN] | 1.323 |
| Q9P2W9 | *STX18* | Syntaxin-18 OS=Homo sapiens GN=STX18 PE=1 SV=1 - [STX18_HUMAN] | 1.392 |
| Q9NR46 | *SH3GLB2* | Isoform 2 of Endophilin-B2 OS=Homo sapiens GN=SH3GLB2 - [SHLB2_HUMAN] | 1.380 |
| O43933 | *PEX1* | Peroxisome biogenesis factor 1 OS=Homo sapiens GN=PEX1 PE=1 SV=1 - [PEX1_HUMAN] | 1.564 |
| Q01831 | *XPC* | DNA repair protein complementing XP-C cells OS=Homo sapiens GN=XPC PE=1 SV=4 - [XPC_HUMAN] | 0.713 |
| P16333 | *NCK1* | Cytoplasmic protein NCK1 OS=Homo sapiens GN=NCK1 PE=1 SV=1 - [NCK1_HUMAN] | 0.757 |
| Q96SB8 | *SMC6* | Structural maintenance of chromosomes protein 6 OS=Homo sapiens GN=SMC6 PE=1 SV=2 - [SMC6_HUMAN] | 1.644 |
| Q9Y385 | *UBE2J1* | Ubiquitin-conjugating enzyme E2 J1 OS=Homo sapiens GN=UBE2J1 PE=1 SV=2 - [UB2J1_HUMAN] | 1.471 |
| Q5H9L2 | *TCEAL5* | Transcription elongation factor A protein-like 5 OS=Homo sapiens GN=TCEAL5 PE=1 SV=1 - [TCAL5_HUMAN] | 0.351 |
| Q9UQ26 | *RIMS2* | Isoform 4 of Regulating synaptic membrane exocytosis protein 2 OS=Homo sapiens GN=RIMS2 - [RIMS2_HUMAN] | 0.764 |
| A5YKK6 | *CNOT1* | Isoform 2 of CCR4-NOT transcription complex subunit 1 OS=Homo sapiens GN=CNOT1 - [CNOT1_HUMAN] | 1.169 |
| Q9H0S4 | *DDX47* | Probable ATP-dependent RNA helicase DDX47 OS=Homo sapiens GN=DDX47 PE=1 SV=1 - [DDX47_HUMAN] | 1.370 |
| Q9HCU9 | *BRMS1* | Breast cancer metastasis-suppressor 1 OS=Homo sapiens GN=BRMS1 PE=1 SV=1 - [BRMS1_HUMAN] | 1.556 |
| Q13642 | *FHL1* | Isoform 3 of Four and a half LIM domains protein 1 OS=Homo sapiens GN=FHL1 - [FHL1_HUMAN] | 0.330 |
| P07602 | *PSAP* | Proactivator polypeptide OS=Homo sapiens GN=PSAP PE=1 SV=2 - [SAP_HUMAN] | 0.626 |
| Q9BVJ7 | *DUSP23* | Dual specificity protein phosphatase 23 OS=Homo sapiens GN=DUSP23 PE=1 SV=1 - [DUS23_HUMAN] | 1.285 |
| Q4G0J3 | *LARP7* | La-related protein 7 OS=Homo sapiens GN=LARP7 PE=1 SV=1 - [LARP7_HUMAN] | 1.119 |
| O15247 | *CLIC2* | Chloride intracellular channel protein 2 OS=Homo sapiens GN=CLIC2 PE=1 SV=3 - [CLIC2_HUMAN] | 0.339 |
| P50897 | *PPT1* | Isoform 2 of Palmitoyl-protein thioesterase 1 OS=Homo sapiens GN=PPT1 - [PPT1_HUMAN] | 0.636 |
| P56277 | *CMC4* | Mature T-cell proliferation 1 neighbor protein OS=Homo sapiens GN=MTCP1NB PE=1 SV=1 - [MTCNB_HUMAN] | 0.846 |
| Q8ND24 | *RNF214* | RING finger protein 214 OS=Homo sapiens GN=RNF214 PE=1 SV=2 - [RN214_HUMAN] | 1.279 |
| P07225 | *PROS1* | Vitamin K-dependent protein S OS=Homo sapiens GN=PROS1 PE=1 SV=1 - [PROS_HUMAN] | 0.523 |
| Q9Y3D0 | *FAM96B* | Mitotic spindle-associated MMXD complex subunit MIP18 OS=Homo sapiens GN=FAM96B PE=1 SV=1 - [MIP18_HUMAN] | 1.207 |
| P41252 | *IARS* | Isoleucine--tRNA ligase, cytoplasmic OS=Homo sapiens GN=IARS PE=1 SV=2 - [SYIC_HUMAN] | 1.114 |
| Q14653 | *IRF3* | Interferon regulatory factor 3 OS=Homo sapiens GN=IRF3 PE=1 SV=1 - [IRF3_HUMAN] | 1.273 |
| P21757 | *MSR1* | Isoform II of Macrophage scavenger receptor types I and II OS=Homo sapiens GN=MSR1 - [MSRE_HUMAN] | 0.725 |
| Q9Y2G0 | *EFR3B* | Isoform 3 of Protein EFR3 homolog B OS=Homo sapiens GN=EFR3B - [EFR3B_HUMAN] | 0.716 |
| Q8WY22 | *BRI3BP* | BRI3-binding protein OS=Homo sapiens GN=BRI3BP PE=1 SV=1 - [BRI3B_HUMAN] | 1.668 |
| Q86TU7 | *SETD3* | Histone-lysine N-methyltransferase setd3 OS=Homo sapiens GN=SETD3 PE=1 SV=1 - [SETD3_HUMAN] | 1.295 |
| Q86SR1 | *GALNT10* | Isoform 3 of Polypeptide N-acetylgalactosaminyltransferase 10 OS=Homo sapiens GN=GALNT10 - [GLT10_HUMAN] | 1.643 |
| O75718 | *CRTAP* | Cartilage-associated protein OS=Homo sapiens GN=CRTAP PE=1 SV=1 - [CRTAP_HUMAN] | 1.500 |
| P57735 | *RAB25* | Ras-related protein Rab-25 OS=Homo sapiens GN=RAB25 PE=1 SV=2 - [RAB25_HUMAN] | 1.272 |
| Q9UI15 | *TAGLN3* | Transgelin-3 OS=Homo sapiens GN=TAGLN3 PE=1 SV=2 - [TAGL3_HUMAN] | 0.438 |
| P48735 | *IDH2* | Isocitrate dehydrogenase [NADP], mitochondrial OS=Homo sapiens GN=IDH2 PE=1 SV=2 - [IDHP_HUMAN] | 2.080 |
| Q9H1C7 | *CYSTM1* | UPF0467 protein C5orf32 OS=Homo sapiens GN=C5orf32 PE=2 SV=1 - [CE032_HUMAN] | 0.560 |
| Q658P3 | *STEAP3* | Isoform 4 of Metalloreductase STEAP3 OS=Homo sapiens GN=STEAP3 - [STEA3_HUMAN] | 0.627 |
| P49585 | *PCYT1A* | Choline-phosphate cytidylyltransferase A OS=Homo sapiens GN=PCYT1A PE=1 SV=2 - [PCY1A_HUMAN] | 0.773 |
| Q9Y4E6 | *WDR7* | Isoform 2 of WD repeat-containing protein 7 OS=Homo sapiens GN=WDR7 - [WDR7_HUMAN] | 0.828 |
| O00159 | *MYO1C* | Isoform 2 of Myosin-Ic OS=Homo sapiens GN=MYO1C - [MYO1C_HUMAN] | 0.680 |
| Q02750 | *MAP2K1* | Isoform 2 of Dual specificity mitogen-activated protein kinase kinase 1 OS=Homo sapiens GN=MAP2K1 - [MP2K1_HUMAN] | 0.790 |
| Q12904 | *AIMP1* | Aminoacyl tRNA synthase complex-interacting multifunctional protein 1 OS=Homo sapiens GN=AIMP1 PE=1 SV=2 - [AIMP1_HUMAN] | 1.186 |
| Q96C90 | *PPP1R14B* | Protein phosphatase 1 regulatory subunit 14B OS=Homo sapiens GN=PPP1R14B PE=1 SV=3 - [PP14B_HUMAN] | 2.031 |
| Q8N2E2 | *VWDE* | von Willebrand factor D and EGF domain-containing protein OS=Homo sapiens GN=VWDE PE=2 SV=4 - [VWDE_HUMAN] | 0.557 |
| Q04760 | *GLO1* | Isoform 2 of Lactoylglutathione lyase OS=Homo sapiens GN=GLO1 - [LGUL_HUMAN] | 0.729 |
| Q9NRX5 | *SERINC1* | Serine incorporator 1 OS=Homo sapiens GN=SERINC1 PE=1 SV=1 - [SERC1_HUMAN] | 0.710 |
| O75962 | *TRIO* | Isoform 5 of Triple functional domain protein OS=Homo sapiens GN=TRIO - [TRIO_HUMAN] | 1.428 |
| O43633 | *CHMP2A* | Charged multivesicular body protein 2a OS=Homo sapiens GN=CHMP2A PE=1 SV=1 - [CHM2A_HUMAN] | 0.867 |
| Q6NUQ1 | *RINT1* | RAD50-interacting protein 1 OS=Homo sapiens GN=RINT1 PE=1 SV=1 - [RINT1_HUMAN] | 1.325 |
| Q9Y5K6 | *CD2AP* | CD2-associated protein OS=Homo sapiens GN=CD2AP PE=1 SV=1 - [CD2AP_HUMAN] | 1.212 |
| O75525 | *KHDRBS3* | Isoform 2 of KH domain-containing, RNA-binding, signal transduction-associated protein 3 OS=Homo sapiens GN=KHDRBS3 - [KHDR3_HUMAN] | 1.514 |
| P05091 | *ALDH2* | Aldehyde dehydrogenase, mitochondrial OS=Homo sapiens GN=ALDH2 PE=1 SV=2 - [ALDH2_HUMAN] | 0.618 |
| P55072 | *VCP* | Transitional endoplasmic reticulum ATPase OS=Homo sapiens GN=VCP PE=1 SV=4 - [TERA_HUMAN] | 1.165 |
| P10415 | *BCL2* | Isoform Beta of Apoptosis regulator Bcl-2 OS=Homo sapiens GN=BCL2 - [BCL2_HUMAN] | 0.542 |
| P32322 | *PYCR1* | Pyrroline-5-carboxylate reductase 1, mitochondrial OS=Homo sapiens GN=PYCR1 PE=1 SV=2 - [P5CR1_HUMAN] | 1.831 |
| Q96DB2 | *HDAC11* | Histone deacetylase 11 OS=Homo sapiens GN=HDAC11 PE=1 SV=1 - [HDA11_HUMAN] | 0.638 |
| Q96DC8 | *ECHDC3* | Enoyl-CoA hydratase domain-containing protein 3, mitochondrial OS=Homo sapiens GN=ECHDC3 PE=1 SV=2 - [ECHD3_HUMAN] | 0.631 |
| Q96E11 | *MRRF* | Isoform 8 of Ribosome-recycling factor, mitochondrial OS=Homo sapiens GN=MRRF - [RRFM_HUMAN] | 0.805 |
| O95372 | *LYPLA2* | Acyl-protein thioesterase 2 OS=Homo sapiens GN=LYPLA2 PE=1 SV=1 - [LYPA2_HUMAN] | 1.343 |
| Q9UKM9 | *RALY* | RNA-binding protein Raly OS=Homo sapiens GN=RALY PE=1 SV=1 - [RALY_HUMAN] | 1.392 |
| P02794 | *FTH1* | Ferritin heavy chain OS=Homo sapiens GN=FTH1 PE=1 SV=2 - [FRIH_HUMAN] | 0.683 |
| P20700 | *LMNB1* | Lamin-B1 OS=Homo sapiens GN=LMNB1 PE=1 SV=2 - [LMNB1_HUMAN] | 1.327 |
| Q9NY33 | *DPP3* | Dipeptidyl peptidase 3 OS=Homo sapiens GN=DPP3 PE=1 SV=2 - [DPP3_HUMAN] | 1.570 |
| Q96CN9 | *GCC1* | GRIP and coiled-coil domain-containing protein 1 OS=Homo sapiens GN=GCC1 PE=1 SV=1 - [GCC1_HUMAN] | 1.190 |
| Q9Y3A5 | *SBDS* | Ribosome maturation protein SBDS OS=Homo sapiens GN=SBDS PE=1 SV=4 - [SBDS_HUMAN] | 0.774 |
| P22102 | *GART* | Trifunctional purine biosynthetic protein adenosine-3 OS=Homo sapiens GN=GART PE=1 SV=1 - [PUR2_HUMAN] | 1.160 |
| Q9UJ68 | *MSRA* | Isoform 2 of Mitochondrial peptide methionine sulfoxide reductase OS=Homo sapiens GN=MSRA - [MSRA_HUMAN] | 0.569 |
| O43505 | *B4GAT1* | N-acetyllactosaminide beta-1,3-N-acetylglucosaminyltransferase OS=Homo sapiens GN=B3GNT1 PE=1 SV=1 - [B3GN1_HUMAN] | 0.514 |
| O60243 | *HS6ST1* | Isoform 2 of Heparan-sulfate 6-O-sulfotransferase 1 OS=Homo sapiens GN=HS6ST1 - [H6ST1_HUMAN] | 0.621 |
| Q14012 | *CAMK1* | Calcium/calmodulin-dependent protein kinase type 1 OS=Homo sapiens GN=CAMK1 PE=1 SV=1 - [KCC1A_HUMAN] | 0.747 |
| Q96RP9 | *GFM1* | Elongation factor G, mitochondrial OS=Homo sapiens GN=GFM1 PE=1 SV=2 - [EFGM_HUMAN] | 0.770 |
| Q92563 | *SPOCK2* | Testican-2 OS=Homo sapiens GN=SPOCK2 PE=1 SV=1 - [TICN2_HUMAN] | 0.182 |
| Q9Y375 | *NDUFAF1* | Complex I intermediate-associated protein 30, mitochondrial OS=Homo sapiens GN=NDUFAF1 PE=1 SV=2 - [CIA30_HUMAN] | 0.787 |
| Q9BZ67 | *FRMD8* | Isoform 2 of FERM domain-containing protein 8 OS=Homo sapiens GN=FRMD8 - [FRMD8_HUMAN] | 0.879 |
| P54105 | *CLNS1A* | Methylosome subunit pICln OS=Homo sapiens GN=CLNS1A PE=1 SV=1 - [ICLN_HUMAN] | 1.310 |
| Q15528 | *MED22* | Isoform Surf5A of Mediator of RNA polymerase II transcription subunit 22 OS=Homo sapiens GN=MED22 - [MED22_HUMAN] | 1.155 |
| Q9BUK0 | *CHCHD7* | Coiled-coil-helix-coiled-coil-helix domain-containing protein 7 OS=Homo sapiens GN=CHCHD7 PE=2 SV=1 - [CHCH7_HUMAN] | 0.586 |
| P43652 | *AFM* | Afamin OS=Homo sapiens GN=AFM PE=1 SV=1 - [AFAM_HUMAN] | 0.628 |
| Q8IWJ2 | *GCC2* | GRIP and coiled-coil domain-containing protein 2 OS=Homo sapiens GN=GCC2 PE=1 SV=4 - [GCC2_HUMAN] | 1.209 |
| Q00796 | *SORD* | Sorbitol dehydrogenase OS=Homo sapiens GN=SORD PE=1 SV=4 - [DHSO_HUMAN] | 0.266 |
| P42858 | *HTT* | Huntingtin OS=Homo sapiens GN=HTT PE=1 SV=2 - [HD_HUMAN] | 1.350 |
| P20340 | *RAB6A* | Ras-related protein Rab-6A OS=Homo sapiens GN=RAB6A PE=1 SV=3 - [RAB6A_HUMAN] | 1.144 |
| Q96CN7 | *ISOC1* | Isochorismatase domain-containing protein 1 OS=Homo sapiens GN=ISOC1 PE=1 SV=3 - [ISOC1_HUMAN] | 0.703 |
| Q6FI81 | *CIAPIN1* | Isoform 3 of Anamorsin OS=Homo sapiens GN=CIAPIN1 - [CPIN1_HUMAN] | 1.637 |
| O75369 | *FLNB* | Isoform 5 of Filamin-B OS=Homo sapiens GN=FLNB - [FLNB_HUMAN] | 0.657 |
| P16615 | *ATP2A2* | Isoform 2 of Sarcoplasmic/endoplasmic reticulum calcium ATPase 2 OS=Homo sapiens GN=ATP2A2 - [AT2A2_HUMAN] | 1.206 |
| O75663 | *TIPRL* | TIP41-like protein OS=Homo sapiens GN=TIPRL PE=1 SV=2 - [TIPRL_HUMAN] | 1.327 |
| Q5W0U4 | *BSPRY* | Isoform 2 of B box and SPRY domain-containing protein OS=Homo sapiens GN=BSPRY - [BSPRY_HUMAN] | 0.759 |
| P21397 | *MAOA* | Amine oxidase [flavin-containing] A OS=Homo sapiens GN=MAOA PE=1 SV=1 - [AOFA_HUMAN] | 0.545 |
| O14880 | *MGST3* | Microsomal glutathione S-transferase 3 OS=Homo sapiens GN=MGST3 PE=1 SV=1 - [MGST3_HUMAN] | 0.683 |
| Q14247 | *CTTN* | Src substrate cortactin OS=Homo sapiens GN=CTTN PE=1 SV=2 - [SRC8_HUMAN] | 1.297 |
| P10909 | *CLU* | Isoform 3 of Clusterin OS=Homo sapiens GN=CLU - [CLUS_HUMAN] | 0.375 |
| O60547 | *GMDS* | GDP-mannose 4,6 dehydratase OS=Homo sapiens GN=GMDS PE=1 SV=1 - [GMDS_HUMAN] | 1.999 |
| Q9UI12 | *ATP6V1H* | Isoform 2 of V-type proton ATPase subunit H OS=Homo sapiens GN=ATP6V1H - [VATH_HUMAN] | 0.850 |
| Q6GMV2 | *SMYD5* | SET and MYND domain-containing protein 5 OS=Homo sapiens GN=SMYD5 PE=1 SV=2 - [SMYD5_HUMAN] | 0.879 |
| O76062 | *TM7SF2* | Isoform 2 of Delta(14)-sterol reductase OS=Homo sapiens GN=TM7SF2 - [ERG24_HUMAN] | 1.722 |
| Q5T8D3 | *ACBD5* | Isoform 4 of Acyl-CoA-binding domain-containing protein 5 OS=Homo sapiens GN=ACBD5 - [ACBD5_HUMAN] | 1.352 |
| Q03001 | *DST* | Isoform 2 of Dystonin OS=Homo sapiens GN=DST - [DYST_HUMAN] | 1.411 |
| P18065 | *IGFBP2* | Insulin-like growth factor-binding protein 2 OS=Homo sapiens GN=IGFBP2 PE=1 SV=2 - [IBP2_HUMAN] | 3.554 |
| Q9UBR2 | *CTSZ* | Cathepsin Z OS=Homo sapiens GN=CTSZ PE=1 SV=1 - [CATZ_HUMAN] | 0.599 |
| O60518 | *RANBP6* | Ran-binding protein 6 OS=Homo sapiens GN=RANBP6 PE=1 SV=2 - [RNBP6_HUMAN] | 0.672 |
| P26038 | *MSN* | Moesin OS=Homo sapiens GN=MSN PE=1 SV=3 - [MOES_HUMAN] | 0.751 |
| Q29RF7 | *PDS5A* | Sister chromatid cohesion protein PDS5 homolog A OS=Homo sapiens GN=PDS5A PE=1 SV=1 - [PDS5A_HUMAN] | 1.127 |
| Q9H147 | *DNTTIP1* | Deoxynucleotidyltransferase terminal-interacting protein 1 OS=Homo sapiens GN=DNTTIP1 PE=1 SV=2 - [TDIF1_HUMAN] | 0.705 |
| Q8WWX9 | *#N/A* | Selenoprotein M OS=Homo sapiens GN=SELM PE=1 SV=3 - [SELM_HUMAN] | 0.642 |
| Q13310 | *PABPC4* | Isoform 2 of Polyadenylate-binding protein 4 OS=Homo sapiens GN=PABPC4 - [PABP4_HUMAN] | 1.162 |
| P61586 | *RHOA* | Transforming protein RhoA OS=Homo sapiens GN=RHOA PE=1 SV=1 - [RHOA_HUMAN] | 0.909 |
| Q9H7C9 | *AAMDC* | Isoform 3 of UPF0366 protein C11orf67 OS=Homo sapiens GN=C11orf67 - [CK067_HUMAN] | 1.355 |
| Q14642 | *INPP5A* | Type I inositol-1,4,5-trisphosphate 5-phosphatase OS=Homo sapiens GN=INPP5A PE=2 SV=1 - [I5P1_HUMAN] | 0.738 |
| Q9UJY5 | *GGA1* | Isoform 2 of ADP-ribosylation factor-binding protein GGA1 OS=Homo sapiens GN=GGA1 - [GGA1_HUMAN] | 1.354 |
| P17936 | *IGFBP3* | Insulin-like growth factor-binding protein 3 OS=Homo sapiens GN=IGFBP3 PE=1 SV=2 - [IBP3_HUMAN] | 0.222 |
| P63092 | *GNAS* | Guanine nucleotide-binding protein G(s) subunit alpha isoforms short OS=Homo sapiens GN=GNAS PE=1 SV=1 - [GNAS2_HUMAN] | 0.626 |
| Q8NE62 | *CHDH* | Choline dehydrogenase, mitochondrial OS=Homo sapiens GN=CHDH PE=2 SV=2 - [CHDH_HUMAN] | 0.613 |
| Q99417 | *MYCBP* | C-Myc-binding protein OS=Homo sapiens GN=MYCBP PE=1 SV=3 - [MYCBP_HUMAN] | 1.304 |
| O60476 | *MAN1A2* | Mannosyl-oligosaccharide 1,2-alpha-mannosidase IB OS=Homo sapiens GN=MAN1A2 PE=2 SV=1 - [MA1A2_HUMAN] | 1.400 |
| Q9UBB9 | *TFIP11* | Tuftelin-interacting protein 11 OS=Homo sapiens GN=TFIP11 PE=1 SV=1 - [TFP11_HUMAN] | 1.176 |
| P50454 | *SERPINH1* | Serpin H1 OS=Homo sapiens GN=SERPINH1 PE=1 SV=2 - [SERPH_HUMAN] | 1.806 |
| Q9UPQ0 | *LIMCH1* | Isoform 9 of LIM and calponin homology domains-containing protein 1 OS=Homo sapiens GN=LIMCH1 - [LIMC1_HUMAN] | 0.583 |
| Q14141 | *SEPT6* | Isoform I of Septin-6 OS=Homo sapiens GN=SEPT6 - [SEPT6_HUMAN] | 0.739 |
| Q9NVM4 | *PRMT7* | Isoform 4 of Protein arginine N-methyltransferase 7 OS=Homo sapiens GN=PRMT7 - [ANM7_HUMAN] | 1.156 |
| P02766 | *TTR* | Transthyretin OS=Homo sapiens GN=TTR PE=1 SV=1 - [TTHY_HUMAN] | 0.598 |
| O43447 | *PPIH* | Peptidyl-prolyl cis-trans isomerase H OS=Homo sapiens GN=PPIH PE=1 SV=1 - [PPIH_HUMAN] | 1.145 |
| P33121 | *ACSL1* | Isoform 2 of Long-chain-fatty-acid--CoA ligase 1 OS=Homo sapiens GN=ACSL1 - [ACSL1_HUMAN] | 0.600 |
| P62854 | *RPS26* | 40S ribosomal protein S26 OS=Homo sapiens GN=RPS26 PE=1 SV=3 - [RS26_HUMAN] | 1.292 |
| P25788 | *PSMA3* | Isoform 2 of Proteasome subunit alpha type-3 OS=Homo sapiens GN=PSMA3 - [PSA3_HUMAN] | 1.174 |
| P17540 | *CKMT2* | Creatine kinase S-type, mitochondrial OS=Homo sapiens GN=CKMT2 PE=1 SV=2 - [KCRS_HUMAN] | 0.519 |
| Q8WUM4 | *PDCD6IP* | Programmed cell death 6-interacting protein OS=Homo sapiens GN=PDCD6IP PE=1 SV=1 - [PDC6I_HUMAN] | 0.809 |
| Q9NRR5 | *UBQLN4* | Ubiquilin-4 OS=Homo sapiens GN=UBQLN4 PE=1 SV=2 - [UBQL4_HUMAN] | 1.395 |
| O95571 | *ETHE1* | Protein ETHE1, mitochondrial OS=Homo sapiens GN=ETHE1 PE=1 SV=2 - [ETHE1_HUMAN] | 0.644 |
| O75369 | *FLNB* | Isoform 6 of Filamin-B OS=Homo sapiens GN=FLNB - [FLNB_HUMAN] | 0.720 |
| Q9Y3D6 | *FIS1* | Mitochondrial fission 1 protein OS=Homo sapiens GN=FIS1 PE=1 SV=2 - [FIS1_HUMAN] | 0.803 |
| Q8N131 | *TMEM123* | Isoform 2 of Porimin OS=Homo sapiens GN=TMEM123 - [PORIM_HUMAN] | 1.330 |
| P82094 | *TMF1* | TATA element modulatory factor OS=Homo sapiens GN=TMF1 PE=1 SV=2 - [TMF1_HUMAN] | 0.856 |
| Q9BRQ6 | *CHCHD6* | Coiled-coil-helix-coiled-coil-helix domain-containing protein 6 OS=Homo sapiens GN=CHCHD6 PE=1 SV=1 - [CHCH6_HUMAN] | 0.762 |
| Q8IXS6 | *PALM2* | Paralemmin-2 OS=Homo sapiens GN=PALM2 PE=1 SV=3 - [PALM2_HUMAN] | 1.583 |
| A2RRP1 | *NBAS* | Isoform 2 of Neuroblastoma-amplified sequence OS=Homo sapiens GN=NBAS - [NBAS_HUMAN] | 1.209 |
| Q8TDJ6 | *DMXL2* | DmX-like protein 2 OS=Homo sapiens GN=DMXL2 PE=1 SV=2 - [DMXL2_HUMAN] | 0.764 |
| Q8WX92 | *NELFB* | Negative elongation factor B OS=Homo sapiens GN=COBRA1 PE=1 SV=1 - [NELFB_HUMAN] | 1.285 |
| Q5JRX3 | *PITRM1* | Presequence protease, mitochondrial OS=Homo sapiens GN=PITRM1 PE=1 SV=2 - [PREP_HUMAN] | 1.377 |
| P05156 | *CFI* | Complement factor I OS=Homo sapiens GN=CFI PE=1 SV=2 - [CFAI_HUMAN] | 0.604 |
| O75600 | *GCAT* | 2-amino-3-ketobutyrate coenzyme A ligase, mitochondrial OS=Homo sapiens GN=GCAT PE=2 SV=1 - [KBL_HUMAN] | 0.492 |
| Q9UHQ9 | *CYB5R1* | NADH-cytochrome b5 reductase 1 OS=Homo sapiens GN=CYB5R1 PE=1 SV=1 - [NB5R1_HUMAN] | 0.743 |
| A0MZ66 | *KIAA1598* | Isoform 2 of Shootin-1 OS=Homo sapiens GN=KIAA1598 - [SHOT1_HUMAN] | 1.225 |
| Q9NTK5 | *OLA1* | Obg-like ATPase 1 OS=Homo sapiens GN=OLA1 PE=1 SV=2 - [OLA1_HUMAN] | 1.152 |
| O43795 | *MYO1B* | Isoform 2 of Myosin-Ib OS=Homo sapiens GN=MYO1B - [MYO1B_HUMAN] | 0.681 |
| Q99816 | *TSG101* | Tumor susceptibility gene 101 protein OS=Homo sapiens GN=TSG101 PE=1 SV=2 - [TS101_HUMAN] | 1.341 |
| Q96I45 | *TMEM141* | Transmembrane protein 141 OS=Homo sapiens GN=TMEM141 PE=2 SV=1 - [TM141_HUMAN] | 1.357 |
| O75381 | *PEX14* | Isoform 2 of Peroxisomal membrane protein PEX14 OS=Homo sapiens GN=PEX14 - [PEX14_HUMAN] | 1.537 |
| P00740 | *F9* | Coagulation factor IX OS=Homo sapiens GN=F9 PE=1 SV=2 - [FA9_HUMAN] | 0.434 |
| O60762 | *DPM1* | Dolichol-phosphate mannosyltransferase OS=Homo sapiens GN=DPM1 PE=1 SV=1 - [DPM1_HUMAN] | 1.229 |
| Q6UXG2 | *KIAA1324* | Isoform 2 of UPF0577 protein KIAA1324 OS=Homo sapiens GN=KIAA1324 - [K1324_HUMAN] | 0.571 |
| P09496 | *CLTA* | Isoform Non-brain of Clathrin light chain A OS=Homo sapiens GN=CLTA - [CLCA_HUMAN] | 1.155 |
| P08758 | *ANXA5* | Annexin A5 OS=Homo sapiens GN=ANXA5 PE=1 SV=2 - [ANXA5_HUMAN] | 0.775 |
| Q7L311 | *ARMCX2* | Armadillo repeat-containing X-linked protein 2 OS=Homo sapiens GN=ARMCX2 PE=2 SV=1 - [ARMX2_HUMAN] | 0.796 |
| Q8ND56 | *LSM14A* | Protein LSM14 homolog A OS=Homo sapiens GN=LSM14A PE=1 SV=3 - [LS14A_HUMAN] | 1.230 |
| Q8TF61 | *FBXO41* | F-box only protein 41 OS=Homo sapiens GN=FBXO41 PE=2 SV=4 - [FBX41_HUMAN] | 0.767 |
| O75094 | *SLIT3* | Isoform 2 of Slit homolog 3 protein OS=Homo sapiens GN=SLIT3 - [SLIT3_HUMAN] | 0.680 |
| Q08AM6 | *VAC14* | Protein VAC14 homolog OS=Homo sapiens GN=VAC14 PE=1 SV=1 - [VAC14_HUMAN] | 1.197 |
| Q9UKK9 | *NUDT5* | ADP-sugar pyrophosphatase OS=Homo sapiens GN=NUDT5 PE=1 SV=1 - [NUDT5_HUMAN] | 0.837 |
| O75122 | *CLASP2* | CLIP-associating protein 2 OS=Homo sapiens GN=CLASP2 PE=1 SV=2 - [CLAP2_HUMAN] | 0.775 |
| Q9BZQ6 | *EDEM3* | ER degradation-enhancing alpha-mannosidase-like 3 OS=Homo sapiens GN=EDEM3 PE=1 SV=2 - [EDEM3_HUMAN] | 1.244 |
| Q12884 | *FAP* | Seprase OS=Homo sapiens GN=FAP PE=1 SV=5 - [SEPR_HUMAN] | 1.850 |
| P01714 | *#N/A* | Ig lambda chain V-III region SH OS=Homo sapiens PE=1 SV=1 - [LV301_HUMAN] | 0.718 |
| Q9BV38 | *WDR18* | WD repeat-containing protein 18 OS=Homo sapiens GN=WDR18 PE=1 SV=2 - [WDR18_HUMAN] | 1.207 |
| O75695 | *RP2* | Protein XRP2 OS=Homo sapiens GN=RP2 PE=1 SV=4 - [XRP2_HUMAN] | 0.797 |
| Q92541 | *RTF1* | RNA polymerase-associated protein RTF1 homolog OS=Homo sapiens GN=RTF1 PE=1 SV=4 - [RTF1_HUMAN] | 1.234 |
| P08123 | *COL1A2* | Collagen alpha-2(I) chain OS=Homo sapiens GN=COL1A2 PE=1 SV=7 - [CO1A2_HUMAN] | 2.361 |
| P36969 | *GPX4* | Isoform Cytoplasmic of Phospholipid hydroperoxide glutathione peroxidase, mitochondrial OS=Homo sapiens GN=GPX4 - [GPX4_HUMAN] | 0.831 |
| P22234 | *PAICS* | Multifunctional protein ADE2 OS=Homo sapiens GN=PAICS PE=1 SV=3 - [PUR6_HUMAN] | 1.374 |
| Q07075 | *ENPEP* | Glutamyl aminopeptidase OS=Homo sapiens GN=ENPEP PE=1 SV=3 - [AMPE_HUMAN] | 1.602 |
| P30153 | *PPP2R1A* | Serine/threonine-protein phosphatase 2A 65 kDa regulatory subunit A alpha isoform OS=Homo sapiens GN=PPP2R1A PE=1 SV=4 - [2AAA_HUMAN] | 1.159 |
| Q13740 | *ALCAM* | Isoform 2 of CD166 antigen OS=Homo sapiens GN=ALCAM - [CD166_HUMAN] | 0.493 |
| Q86TH1 | *ADAMTSL2* | ADAMTS-like protein 2 OS=Homo sapiens GN=ADAMTSL2 PE=1 SV=1 - [ATL2_HUMAN] | 0.653 |
| Q8NES3 | *LFNG* | Isoform 3 of Beta-1,3-N-acetylglucosaminyltransferase lunatic fringe OS=Homo sapiens GN=LFNG - [LFNG_HUMAN] | 0.562 |
| Q9GZM7 | *TINAGL1* | Tubulointerstitial nephritis antigen-like OS=Homo sapiens GN=TINAGL1 PE=1 SV=1 - [TINAL_HUMAN] | 0.186 |
| Q16568 | *CARTPT* | Cocaine- and amphetamine-regulated transcript protein OS=Homo sapiens GN=CARTPT PE=1 SV=1 - [CART_HUMAN] | 0.417 |
| Q92581 | *SLC9A6* | Isoform 2 of Sodium/hydrogen exchanger 6 OS=Homo sapiens GN=SLC9A6 - [SL9A6_HUMAN] | 0.862 |
| P05198 | *EIF2S1* | Eukaryotic translation initiation factor 2 subunit 1 OS=Homo sapiens GN=EIF2S1 PE=1 SV=3 - [IF2A_HUMAN] | 1.151 |
| P25205 | *MCM3* | DNA replication licensing factor MCM3 OS=Homo sapiens GN=MCM3 PE=1 SV=3 - [MCM3_HUMAN] | 1.391 |
| Q96DC8 | *ECHDC3* | Isoform 2 of Enoyl-CoA hydratase domain-containing protein 3, mitochondrial OS=Homo sapiens GN=ECHDC3 - [ECHD3_HUMAN] | 0.603 |
| P52788 | *SMS* | Isoform 2 of Spermine synthase OS=Homo sapiens GN=SMS - [SPSY_HUMAN] | 1.277 |
| Q13576 | *IQGAP2* | Ras GTPase-activating-like protein IQGAP2 OS=Homo sapiens GN=IQGAP2 PE=1 SV=4 - [IQGA2_HUMAN] | 0.521 |
| Q14019 | *COTL1* | Coactosin-like protein OS=Homo sapiens GN=COTL1 PE=1 SV=3 - [COTL1_HUMAN] | 1.812 |
| P27694 | *RPA1* | Replication protein A 70 kDa DNA-binding subunit OS=Homo sapiens GN=RPA1 PE=1 SV=2 - [RFA1_HUMAN] | 0.841 |
| Q86WA6 | *BPHL* | Isoform 2 of Valacyclovir hydrolase OS=Homo sapiens GN=BPHL - [BPHL_HUMAN] | 1.387 |
| P46379 | *BAG6* | Isoform 2 of Large proline-rich protein BAG6 OS=Homo sapiens GN=BAG6 - [BAG6_HUMAN] | 1.206 |
| Q14393 | *GAS6* | Isoform 3 of Growth arrest-specific protein 6 OS=Homo sapiens GN=GAS6 - [GAS6_HUMAN] | 0.503 |
| P05186 | *ALPL* | Alkaline phosphatase, tissue-nonspecific isozyme OS=Homo sapiens GN=ALPL PE=1 SV=4 - [PPBT_HUMAN] | 0.584 |
| O75531 | *BANF1* | Barrier-to-autointegration factor OS=Homo sapiens GN=BANF1 PE=1 SV=1 - [BAF_HUMAN] | 1.260 |
| Q8IUH3 | *RBM45* | Isoform 2 of RNA-binding protein 45 OS=Homo sapiens GN=RBM45 - [RBM45_HUMAN] | 1.202 |
| Q7Z7A3 | *CTU1* | Cytoplasmic tRNA 2-thiolation protein 1 OS=Homo sapiens GN=CTU1 PE=1 SV=1 - [CTU1_HUMAN] | 1.348 |
| O76081 | *RGS20* | Isoform 3 of Regulator of G-protein signaling 20 OS=Homo sapiens GN=RGS20 - [RGS20_HUMAN] | 1.433 |
| O94903 | *PROSC* | Proline synthase co-transcribed bacterial homolog protein OS=Homo sapiens GN=PROSC PE=1 SV=1 - [PROSC_HUMAN] | 0.769 |
| Q9BX59 | *TAPBPL* | Tapasin-related protein OS=Homo sapiens GN=TAPBPL PE=1 SV=2 - [TPSNR_HUMAN] | 0.666 |
| O43426 | *SYNJ1* | Isoform 3 of Synaptojanin-1 OS=Homo sapiens GN=SYNJ1 - [SYNJ1_HUMAN] | 0.748 |
| P98160 | *HSPG2* | Basement membrane-specific heparan sulfate proteoglycan core protein OS=Homo sapiens GN=HSPG2 PE=1 SV=4 - [PGBM_HUMAN] | 0.597 |
| Q96SI9 | *STRBP* | Isoform 2 of Spermatid perinuclear RNA-binding protein OS=Homo sapiens GN=STRBP - [STRBP_HUMAN] | 1.452 |
| P05107 | *ITGB2* | Integrin beta-2 OS=Homo sapiens GN=ITGB2 PE=1 SV=2 - [ITB2_HUMAN] | 0.576 |
| P21953 | *BCKDHB* | 2-oxoisovalerate dehydrogenase subunit beta, mitochondrial OS=Homo sapiens GN=BCKDHB PE=1 SV=2 - [ODBB_HUMAN] | 0.753 |
| P83593 | *#N/A* | Ig kappa chain V-IV region STH (Fragment) OS=Homo sapiens PE=1 SV=1 - [KV405_HUMAN] | 0.495 |
| O75608 | *LYPLA1* | Isoform 2 of Acyl-protein thioesterase 1 OS=Homo sapiens GN=LYPLA1 - [LYPA1_HUMAN] | 1.321 |
| Q8IXK0 | *PHC2* | Isoform 3 of Polyhomeotic-like protein 2 OS=Homo sapiens GN=PHC2 - [PHC2_HUMAN] | 1.329 |
| Q9UL25 | *RAB21* | Ras-related protein Rab-21 OS=Homo sapiens GN=RAB21 PE=1 SV=3 - [RAB21_HUMAN] | 0.876 |
| P00747 | *PLG* | Plasminogen OS=Homo sapiens GN=PLG PE=1 SV=2 - [PLMN_HUMAN] | 0.629 |
| Q05639 | *EEF1A2* | Elongation factor 1-alpha 2 OS=Homo sapiens GN=EEF1A2 PE=1 SV=1 - [EF1A2_HUMAN] | 0.612 |
| Q96KP4 | *CNDP2* | Cytosolic non-specific dipeptidase OS=Homo sapiens GN=CNDP2 PE=1 SV=2 - [CNDP2_HUMAN] | 0.763 |
| O15212 | *PFDN6* | Prefoldin subunit 6 OS=Homo sapiens GN=PFDN6 PE=1 SV=1 - [PFD6_HUMAN] | 1.302 |
| O14964 | *HGS* | Hepatocyte growth factor-regulated tyrosine kinase substrate OS=Homo sapiens GN=HGS PE=1 SV=1 - [HGS_HUMAN] | 1.125 |
| Q12802 | *AKAP13* | Isoform 3 of A-kinase anchor protein 13 OS=Homo sapiens GN=AKAP13 - [AKP13_HUMAN] | 0.775 |
| P43007 | *SLC1A4* | Neutral amino acid transporter A OS=Homo sapiens GN=SLC1A4 PE=1 SV=1 - [SATT_HUMAN] | 0.630 |
| P16422 | *EPCAM* | Epithelial cell adhesion molecule OS=Homo sapiens GN=EPCAM PE=1 SV=2 - [EPCAM_HUMAN] | 0.530 |
| P62140 | *PPP1CB* | Serine/threonine-protein phosphatase PP1-beta catalytic subunit OS=Homo sapiens GN=PPP1CB PE=1 SV=3 - [PP1B_HUMAN] | 0.793 |
| Q9HD26 | *GOPC* | Isoform 3 of Golgi-associated PDZ and coiled-coil motif-containing protein OS=Homo sapiens GN=GOPC - [GOPC_HUMAN] | 0.860 |
| Q05823 | *RNASEL* | 2-5A-dependent ribonuclease OS=Homo sapiens GN=RNASEL PE=1 SV=2 - [RN5A_HUMAN] | 1.277 |
| P10451 | *SPP1* | Isoform C of Osteopontin OS=Homo sapiens GN=SPP1 - [OSTP_HUMAN] | 0.513 |
| Q9Y6D9 | *MAD1L1* | Mitotic spindle assembly checkpoint protein MAD1 OS=Homo sapiens GN=MAD1L1 PE=1 SV=2 - [MD1L1_HUMAN] | 0.754 |
| P48723 | *HSPA13* | Heat shock 70 kDa protein 13 OS=Homo sapiens GN=HSPA13 PE=1 SV=1 - [HSP13_HUMAN] | 0.762 |
| P60468 | *SEC61B* | Protein transport protein Sec61 subunit beta OS=Homo sapiens GN=SEC61B PE=1 SV=2 - [SC61B_HUMAN] | 0.675 |
| Q9Y5X3 | *SNX5* | Sorting nexin-5 OS=Homo sapiens GN=SNX5 PE=1 SV=1 - [SNX5_HUMAN] | 0.786 |
| Q96JJ3 | *ELMO2* | Engulfment and cell motility protein 2 OS=Homo sapiens GN=ELMO2 PE=1 SV=2 - [ELMO2_HUMAN] | 1.271 |
| Q9BQ04 | *RBM4B* | RNA-binding protein 4B OS=Homo sapiens GN=RBM4B PE=1 SV=1 - [RBM4B_HUMAN] | 1.139 |
| O75460 | *ERN1* | Serine/threonine-protein kinase/endoribonuclease IRE1 OS=Homo sapiens GN=ERN1 PE=1 SV=2 - [ERN1_HUMAN] | 0.735 |
| O15041 | *SEMA3E* | Semaphorin-3E OS=Homo sapiens GN=SEMA3E PE=2 SV=1 - [SEM3E_HUMAN] | 0.454 |
| O95983 | *MBD3* | Isoform 2 of Methyl-CpG-binding domain protein 3 OS=Homo sapiens GN=MBD3 - [MBD3_HUMAN] | 1.249 |
| A6NKF9 | *GPR89C* | Putative Golgi pH regulator C OS=Homo sapiens GN=GPR89C PE=5 SV=2 - [GPHRC_HUMAN] | 1.294 |
| Q01974 | *ROR2* | Tyrosine-protein kinase transmembrane receptor ROR2 OS=Homo sapiens GN=ROR2 PE=1 SV=2 - [ROR2_HUMAN] | 0.356 |
| O15079 | *SNPH* | Syntaphilin OS=Homo sapiens GN=SNPH PE=1 SV=2 - [SNPH_HUMAN] | 0.749 |
| Q08209 | *PPP3CA* | Isoform 2 of Serine/threonine-protein phosphatase 2B catalytic subunit alpha isoform OS=Homo sapiens GN=PPP3CA - [PP2BA_HUMAN] | 1.204 |
| P30793 | *GCH1* | GTP cyclohydrolase 1 OS=Homo sapiens GN=GCH1 PE=1 SV=1 - [GCH1_HUMAN] | 1.378 |
| Q14151 | *SAFB2* | Scaffold attachment factor B2 OS=Homo sapiens GN=SAFB2 PE=1 SV=1 - [SAFB2_HUMAN] | 0.841 |
| Q9UPY3 | *DICER1* | Endoribonuclease Dicer OS=Homo sapiens GN=DICER1 PE=1 SV=3 - [DICER_HUMAN] | 1.141 |
| P62277 | *RPS13* | 40S ribosomal protein S13 OS=Homo sapiens GN=RPS13 PE=1 SV=2 - [RS13_HUMAN] | 1.119 |
| Q10567 | *AP1B1* | Isoform C of AP-1 complex subunit beta-1 OS=Homo sapiens GN=AP1B1 - [AP1B1_HUMAN] | 1.207 |
| Q92805 | *GOLGA1* | Golgin subfamily A member 1 OS=Homo sapiens GN=GOLGA1 PE=1 SV=3 - [GOGA1_HUMAN] | 1.190 |
| O95816 | *BAG2* | BAG family molecular chaperone regulator 2 OS=Homo sapiens GN=BAG2 PE=1 SV=1 - [BAG2_HUMAN] | 1.490 |
| P05413 | *FABP3* | Fatty acid-binding protein, heart OS=Homo sapiens GN=FABP3 PE=1 SV=4 - [FABPH_HUMAN] | 0.512 |
| Q6ZUT6 | *C15orf52* | Isoform 3 of Uncharacterized protein C15orf52 OS=Homo sapiens GN=C15orf52 - [CO052_HUMAN] | 0.629 |
| P06454 | *PTMA* | Isoform 2 of Prothymosin alpha OS=Homo sapiens GN=PTMA - [PTMA_HUMAN] | 1.344 |
| Q9UPQ0 | *LIMCH1* | Isoform 8 of LIM and calponin homology domains-containing protein 1 OS=Homo sapiens GN=LIMCH1 - [LIMC1_HUMAN] | 0.554 |
| Q99536 | *VAT1* | Synaptic vesicle membrane protein VAT-1 homolog OS=Homo sapiens GN=VAT1 PE=1 SV=2 - [VAT1_HUMAN] | 0.702 |
| Q96S52 | *PIGS* | Isoform 2 of GPI transamidase component PIG-S OS=Homo sapiens GN=PIGS - [PIGS_HUMAN] | 1.259 |
| Q9UHB6 | *LIMA1* | LIM domain and actin-binding protein 1 OS=Homo sapiens GN=LIMA1 PE=1 SV=1 - [LIMA1_HUMAN] | 1.297 |
| Q8NES3 | *LFNG* | Isoform 2 of Beta-1,3-N-acetylglucosaminyltransferase lunatic fringe OS=Homo sapiens GN=LFNG - [LFNG_HUMAN] | 0.574 |
| Q9P0V9 | *SEPT10* | Septin-10 OS=Homo sapiens GN=SEPT10 PE=1 SV=2 - [SEP10_HUMAN] | 0.782 |
| P16104 | *H2AFX* | Histone H2A.x OS=Homo sapiens GN=H2AFX PE=1 SV=2 - [H2AX_HUMAN] | 1.313 |
| P78559 | *MAP1A* | Microtubule-associated protein 1A OS=Homo sapiens GN=MAP1A PE=1 SV=6 - [MAP1A_HUMAN] | 0.761 |
| Q9Y6M1 | *IGF2BP2* | Isoform 2 of Insulin-like growth factor 2 mRNA-binding protein 2 OS=Homo sapiens GN=IGF2BP2 - [IF2B2_HUMAN] | 2.104 |
| O14949 | *UQCRQ* | Cytochrome b-c1 complex subunit 8 OS=Homo sapiens GN=UQCRQ PE=1 SV=4 - [QCR8_HUMAN] | 1.200 |
| P35542 | *SAA4* | Serum amyloid A-4 protein OS=Homo sapiens GN=SAA4 PE=1 SV=2 - [SAA4_HUMAN] | 0.535 |
| P05166 | *PCCB* | Propionyl-CoA carboxylase beta chain, mitochondrial OS=Homo sapiens GN=PCCB PE=1 SV=3 - [PCCB_HUMAN] | 0.762 |
| O00712 | *NFIB* | Isoform 3 of Nuclear factor 1 B-type OS=Homo sapiens GN=NFIB - [NFIB_HUMAN] | 0.724 |
| Q9H0V1 | *TMEM168* | Transmembrane protein 168 OS=Homo sapiens GN=TMEM168 PE=1 SV=2 - [TM168_HUMAN] | 1.271 |
| P43246 | *MSH2* | DNA mismatch repair protein Msh2 OS=Homo sapiens GN=MSH2 PE=1 SV=1 - [MSH2_HUMAN] | 1.269 |
| Q14139 | *UBE4A* | Ubiquitin conjugation factor E4 A OS=Homo sapiens GN=UBE4A PE=1 SV=2 - [UBE4A_HUMAN] | 1.125 |
| Q01432 | *AMPD3* | AMP deaminase 3 OS=Homo sapiens GN=AMPD3 PE=1 SV=1 - [AMPD3_HUMAN] | 1.548 |
| Q7Z699 | *SPRED1* | Sprouty-related, EVH1 domain-containing protein 1 OS=Homo sapiens GN=SPRED1 PE=1 SV=2 - [SPRE1_HUMAN] | 0.546 |
| P17812 | *CTPS1* | CTP synthase 1 OS=Homo sapiens GN=CTPS PE=1 SV=2 - [PYRG1_HUMAN] | 1.410 |
| Q9NRY5 | *FAM114A2* | Protein FAM114A2 OS=Homo sapiens GN=FAM114A2 PE=1 SV=4 - [F1142_HUMAN] | 0.812 |
| P14384 | *CPM* | Carboxypeptidase M OS=Homo sapiens GN=CPM PE=1 SV=2 - [CBPM_HUMAN] | 0.409 |
| Q13509 | *TUBB3* | Tubulin beta-3 chain OS=Homo sapiens GN=TUBB3 PE=1 SV=2 - [TBB3_HUMAN] | 1.655 |
| Q04206 | *RELA* | Isoform 2 of Transcription factor p65 OS=Homo sapiens GN=RELA - [TF65_HUMAN] | 1.296 |
| Q13439 | *GOLGA4* | Isoform 3 of Golgin subfamily A member 4 OS=Homo sapiens GN=GOLGA4 - [GOGA4_HUMAN] | 0.757 |
| P04181 | *OAT* | Ornithine aminotransferase, mitochondrial OS=Homo sapiens GN=OAT PE=1 SV=1 - [OAT_HUMAN] | 0.720 |
| Q9NVS9 | *PNPO* | Pyridoxine-5'-phosphate oxidase OS=Homo sapiens GN=PNPO PE=1 SV=1 - [PNPO_HUMAN] | 0.776 |
| Q9BZE4 | *GTPBP4* | Nucleolar GTP-binding protein 1 OS=Homo sapiens GN=GTPBP4 PE=1 SV=3 - [NOG1_HUMAN] | 1.215 |
| P04899 | *GNAI2* | Guanine nucleotide-binding protein G(i) subunit alpha-2 OS=Homo sapiens GN=GNAI2 PE=1 SV=3 - [GNAI2_HUMAN] | 0.798 |
| P17858 | *PFKL* | 6-phosphofructokinase, liver type OS=Homo sapiens GN=PFKL PE=1 SV=6 - [K6PL_HUMAN] | 1.417 |
| Q04864 | *REL* | Proto-oncogene c-Rel OS=Homo sapiens GN=REL PE=1 SV=1 - [REL_HUMAN] | 1.354 |
| P78527 | *PRKDC* | Isoform 2 of DNA-dependent protein kinase catalytic subunit OS=Homo sapiens GN=PRKDC - [PRKDC_HUMAN] | 1.122 |
| Q9H4M9 | *EHD1* | EH domain-containing protein 1 OS=Homo sapiens GN=EHD1 PE=1 SV=2 - [EHD1_HUMAN] | 0.703 |
| Q14112 | *NID2* | Isoform 2 of Nidogen-2 OS=Homo sapiens GN=NID2 - [NID2_HUMAN] | 0.503 |
| Q7KZF4 | *SND1* | Staphylococcal nuclease domain-containing protein 1 OS=Homo sapiens GN=SND1 PE=1 SV=1 - [SND1_HUMAN] | 0.861 |
| Q9Y266 | *NUDC* | Nuclear migration protein nudC OS=Homo sapiens GN=NUDC PE=1 SV=1 - [NUDC_HUMAN] | 1.283 |
| P16219 | *ACADS* | Short-chain specific acyl-CoA dehydrogenase, mitochondrial OS=Homo sapiens GN=ACADS PE=1 SV=1 - [ACADS_HUMAN] | 0.571 |
| Q7Z7B1 | *PIGW* | Phosphatidylinositol-glycan biosynthesis class W protein OS=Homo sapiens GN=PIGW PE=1 SV=1 - [PIGW_HUMAN] | 1.304 |
| P60981 | *DSTN* | Destrin OS=Homo sapiens GN=DSTN PE=1 SV=3 - [DEST_HUMAN] | 0.821 |
| Q03154 | *ACY1* | Aminoacylase-1 OS=Homo sapiens GN=ACY1 PE=1 SV=1 - [ACY1_HUMAN] | 1.304 |
| Q9HBL0 | *TNS1* | Tensin-1 OS=Homo sapiens GN=TNS1 PE=1 SV=2 - [TENS1_HUMAN] | 0.710 |
| O00339 | *MATN2* | Isoform 3 of Matrilin-2 OS=Homo sapiens GN=MATN2 - [MATN2_HUMAN] | 0.466 |
| O00151 | *PDLIM1* | PDZ and LIM domain protein 1 OS=Homo sapiens GN=PDLIM1 PE=1 SV=4 - [PDLI1_HUMAN] | 1.483 |
| Q99714 | *HSD17B10* | Isoform 2 of 3-hydroxyacyl-CoA dehydrogenase type-2 OS=Homo sapiens GN=HSD17B10 - [HCD2_HUMAN] | 0.738 |
| Q9UHG2 | *PCSK1N* | ProSAAS OS=Homo sapiens GN=PCSK1N PE=1 SV=1 - [PCSK1_HUMAN] | 0.674 |
| P40426 | *PBX3* | Isoform PBX3b of Pre-B-cell leukemia transcription factor 3 OS=Homo sapiens GN=PBX3 - [PBX3_HUMAN] | 0.626 |
| Q99715 | *COL12A1* | Isoform 4 of Collagen alpha-1(XII) chain OS=Homo sapiens GN=COL12A1 - [COCA1_HUMAN] | 2.031 |
| Q14554 | *PDIA5* | Protein disulfide-isomerase A5 OS=Homo sapiens GN=PDIA5 PE=1 SV=1 - [PDIA5_HUMAN] | 0.646 |
| P01625 | *#N/A* | Ig kappa chain V-IV region Len OS=Homo sapiens PE=1 SV=2 - [KV402_HUMAN] | 0.454 |
| A3KMH1 | *VWA8* | Isoform 3 of Uncharacterized protein KIAA0564 OS=Homo sapiens GN=KIAA0564 - [K0564_HUMAN] | 0.738 |
| Q9HCC0 | *MCCC2* | Isoform 2 of Methylcrotonoyl-CoA carboxylase beta chain, mitochondrial OS=Homo sapiens GN=MCCC2 - [MCCB_HUMAN] | 0.781 |
| Q684P5 | *RAP1GAP2* | Isoform 3 of Rap1 GTPase-activating protein 2 OS=Homo sapiens GN=RAP1GAP2 - [RPGP2_HUMAN] | 0.782 |
| Q13642 | *FHL1* | Isoform 1 of Four and a half LIM domains protein 1 OS=Homo sapiens GN=FHL1 - [FHL1_HUMAN] | 0.294 |
| O94851 | *MICAL2* | Isoform 2 of Protein MICAL-2 OS=Homo sapiens GN=MICAL2 - [MICA2_HUMAN] | 1.539 |
| Q8IVM0 | *CCDC50* | Coiled-coil domain-containing protein 50 OS=Homo sapiens GN=CCDC50 PE=1 SV=1 - [CCD50_HUMAN] | 0.803 |
| Q9NP80 | *PNPLA8* | Isoform 2 of Calcium-independent phospholipase A2-gamma OS=Homo sapiens GN=PNPLA8 - [PLPL8_HUMAN] | 0.778 |
| Q86VN1 | *VPS36* | Vacuolar protein-sorting-associated protein 36 OS=Homo sapiens GN=VPS36 PE=1 SV=1 - [VPS36_HUMAN] | 1.258 |
| Q9BXR6 | *CFHR5* | Complement factor H-related protein 5 OS=Homo sapiens GN=CFHR5 PE=1 SV=1 - [FHR5_HUMAN] | 0.473 |
| P54136 | *RARS* | Arginine--tRNA ligase, cytoplasmic OS=Homo sapiens GN=RARS PE=1 SV=2 - [SYRC_HUMAN] | 1.190 |
| Q9UGP4 | *LIMD1* | LIM domain-containing protein 1 OS=Homo sapiens GN=LIMD1 PE=1 SV=1 - [LIMD1_HUMAN] | 0.724 |
| P01781 | *#N/A* | Ig heavy chain V-III region GAL OS=Homo sapiens PE=1 SV=1 - [HV320_HUMAN] | 0.465 |
| P15907 | *ST6GAL1* | Beta-galactoside alpha-2,6-sialyltransferase 1 OS=Homo sapiens GN=ST6GAL1 PE=1 SV=1 - [SIAT1_HUMAN] | 0.578 |
| P60484 | *PTEN* | Phosphatidylinositol-3,4,5-trisphosphate 3-phosphatase and dual-specificity protein phosphatase PTEN OS=Homo sapiens GN=PTEN PE=1 SV=1 - [PTEN_HUMAN] | 0.873 |
| P41250 | *GARS* | Glycine--tRNA ligase OS=Homo sapiens GN=GARS PE=1 SV=3 - [SYG_HUMAN] | 1.193 |
| O43324 | *EEF1E1* | Eukaryotic translation elongation factor 1 epsilon-1 OS=Homo sapiens GN=EEF1E1 PE=1 SV=1 - [MCA3_HUMAN] | 1.187 |
| P02792 | *FTL* | Ferritin light chain OS=Homo sapiens GN=FTL PE=1 SV=2 - [FRIL_HUMAN] | 0.712 |
| Q96A49 | *SYAP1* | Synapse-associated protein 1 OS=Homo sapiens GN=SYAP1 PE=1 SV=1 - [SYAP1_HUMAN] | 1.256 |
| P30519 | *HMOX2* | Heme oxygenase 2 OS=Homo sapiens GN=HMOX2 PE=1 SV=2 - [HMOX2_HUMAN] | 1.419 |
| O94925 | *GLS* | Isoform 3 of Glutaminase kidney isoform, mitochondrial OS=Homo sapiens GN=GLS - [GLSK_HUMAN] | 0.855 |
| Q96HA8 | *WDYHV1* | Protein N-terminal glutamine amidohydrolase OS=Homo sapiens GN=WDYHV1 PE=1 SV=2 - [NTAQ1_HUMAN] | 1.675 |
| P07492 | *GRP* | Isoform 2 of Gastrin-releasing peptide OS=Homo sapiens GN=GRP - [GRP_HUMAN] | 0.543 |
| P46781 | *RPS9* | 40S ribosomal protein S9 OS=Homo sapiens GN=RPS9 PE=1 SV=3 - [RS9_HUMAN] | 1.099 |
| P60880 | *SNAP25* | Isoform SNAP-25a of Synaptosomal-associated protein 25 OS=Homo sapiens GN=SNAP25 - [SNP25_HUMAN] | 0.633 |
| P63092 | *GNAS* | Isoform 3 of Guanine nucleotide-binding protein G(s) subunit alpha isoforms short OS=Homo sapiens GN=GNAS - [GNAS2_HUMAN] | 0.694 |
| P62714 | *PPP2CB* | Serine/threonine-protein phosphatase 2A catalytic subunit beta isoform OS=Homo sapiens GN=PPP2CB PE=1 SV=1 - [PP2AB_HUMAN] | 1.126 |
| Q9UBQ7 | *GRHPR* | Glyoxylate reductase/hydroxypyruvate reductase OS=Homo sapiens GN=GRHPR PE=1 SV=1 - [GRHPR_HUMAN] | 0.834 |
| O76003 | *GLRX3* | Glutaredoxin-3 OS=Homo sapiens GN=GLRX3 PE=1 SV=2 - [GLRX3_HUMAN] | 1.251 |
| Q9NPI6 | *DCP1A* | mRNA-decapping enzyme 1A OS=Homo sapiens GN=DCP1A PE=1 SV=2 - [DCP1A_HUMAN] | 1.182 |
| Q9UPR5 | *SLC8A2* | Sodium/calcium exchanger 2 OS=Homo sapiens GN=SLC8A2 PE=2 SV=2 - [NAC2_HUMAN] | 0.587 |
| P53367 | *ARFIP1* | Isoform A of Arfaptin-1 OS=Homo sapiens GN=ARFIP1 - [ARFP1_HUMAN] | 1.152 |
| O60687 | *SRPX2* | Sushi repeat-containing protein SRPX2 OS=Homo sapiens GN=SRPX2 PE=1 SV=1 - [SRPX2_HUMAN] | 1.826 |
| Q8IXJ6 | *SIRT2* | Isoform 2 of NAD-dependent deacetylase sirtuin-2 OS=Homo sapiens GN=SIRT2 - [SIRT2_HUMAN] | 1.273 |
| Q9UJ41 | *RABGEF1* | Isoform 2 of Rab5 GDP/GTP exchange factor OS=Homo sapiens GN=RABGEF1 - [RABX5_HUMAN] | 1.222 |
| O43772 | *SLC25A20* | Mitochondrial carnitine/acylcarnitine carrier protein OS=Homo sapiens GN=SLC25A20 PE=1 SV=1 - [MCAT_HUMAN] | 0.831 |
| Q9Y5Q9 | *GTF3C3* | Isoform 2 of General transcription factor 3C polypeptide 3 OS=Homo sapiens GN=GTF3C3 - [TF3C3_HUMAN] | 1.170 |
| Q8IUX7 | *AEBP1* | Adipocyte enhancer-binding protein 1 OS=Homo sapiens GN=AEBP1 PE=1 SV=1 - [AEBP1_HUMAN] | 1.812 |
| Q96I51 | *WBSCR16* | Williams-Beuren syndrome chromosomal region 16 protein OS=Homo sapiens GN=WBSCR16 PE=1 SV=2 - [WBS16_HUMAN] | 0.863 |
| Q13464 | *ROCK1* | Rho-associated protein kinase 1 OS=Homo sapiens GN=ROCK1 PE=1 SV=1 - [ROCK1_HUMAN] | 0.687 |
| O75061 | *DNAJC6* | Isoform 3 of Putative tyrosine-protein phosphatase auxilin OS=Homo sapiens GN=DNAJC6 - [AUXI_HUMAN] | 0.672 |
| P58546 | *MTPN* | Myotrophin OS=Homo sapiens GN=MTPN PE=1 SV=2 - [MTPN_HUMAN] | 1.199 |
| O00468 | *AGRN* | Agrin OS=Homo sapiens GN=AGRN PE=1 SV=4 - [AGRIN_HUMAN] | 0.485 |
| Q9P2R7 | *SUCLA2* | Isoform 2 of Succinyl-CoA ligase [ADP-forming] subunit beta, mitochondrial OS=Homo sapiens GN=SUCLA2 - [SUCB1_HUMAN] | 0.828 |
| O60513 | *B4GALT4* | Beta-1,4-galactosyltransferase 4 OS=Homo sapiens GN=B4GALT4 PE=1 SV=1 - [B4GT4_HUMAN] | 0.443 |
| P43686 | *PSMC4* | Isoform 2 of 26S protease regulatory subunit 6B OS=Homo sapiens GN=PSMC4 - [PRS6B_HUMAN] | 1.201 |
| P02790 | *HPX* | Hemopexin OS=Homo sapiens GN=HPX PE=1 SV=2 - [HEMO_HUMAN] | 0.676 |
| Q9NWZ8 | *GEMIN8* | Gem-associated protein 8 OS=Homo sapiens GN=GEMIN8 PE=1 SV=1 - [GEMI8_HUMAN] | 1.231 |
| O95292 | *VAPB* | Vesicle-associated membrane protein-associated protein B/C OS=Homo sapiens GN=VAPB PE=1 SV=3 - [VAPB_HUMAN] | 1.275 |
| Q96RF0 | *SNX18* | Isoform 2 of Sorting nexin-18 OS=Homo sapiens GN=SNX18 - [SNX18_HUMAN] | 0.809 |
| O75882 | *ATRN* | Isoform 3 of Attractin OS=Homo sapiens GN=ATRN - [ATRN_HUMAN] | 0.656 |
| P49189 | *ALDH9A1* | 4-trimethylaminobutyraldehyde dehydrogenase OS=Homo sapiens GN=ALDH9A1 PE=1 SV=3 - [AL9A1_HUMAN] | 0.757 |
| P08697 | *SERPINF2* | Alpha-2-antiplasmin OS=Homo sapiens GN=SERPINF2 PE=1 SV=3 - [A2AP_HUMAN] | 0.682 |
| Q9UHG3 | *PCYOX1* | Prenylcysteine oxidase 1 OS=Homo sapiens GN=PCYOX1 PE=1 SV=3 - [PCYOX_HUMAN] | 0.813 |
| Q8IYQ7 | *THNSL1* | Threonine synthase-like 1 OS=Homo sapiens GN=THNSL1 PE=1 SV=2 - [THNS1_HUMAN] | 0.766 |
| P55290 | *CDH13* | Cadherin-13 OS=Homo sapiens GN=CDH13 PE=1 SV=1 - [CAD13_HUMAN] | 0.549 |
| Q08J23 | *NSUN2* | tRNA (cytosine(34)-C(5))-methyltransferase OS=Homo sapiens GN=NSUN2 PE=1 SV=2 - [NSUN2_HUMAN] | 1.314 |
| P16949 | *STMN1* | Stathmin OS=Homo sapiens GN=STMN1 PE=1 SV=3 - [STMN1_HUMAN] | 1.718 |
| O14523 | *C2CD2L* | C2 domain-containing protein 2-like OS=Homo sapiens GN=C2CD2L PE=1 SV=3 - [C2C2L_HUMAN] | 1.304 |
| Q09472 | *EP300* | Histone acetyltransferase p300 OS=Homo sapiens GN=EP300 PE=1 SV=2 - [EP300_HUMAN] | 1.251 |
| Q01484 | *ANK2* | Ankyrin-2 OS=Homo sapiens GN=ANK2 PE=1 SV=3 - [ANK2_HUMAN] | 0.670 |
| Q93084 | *ATP2A3* | Isoform SERCA3D of Sarcoplasmic/endoplasmic reticulum calcium ATPase 3 OS=Homo sapiens GN=ATP2A3 - [AT2A3_HUMAN] | 1.539 |
| Q13614 | *MTMR2* | Myotubularin-related protein 2 OS=Homo sapiens GN=MTMR2 PE=1 SV=4 - [MTMR2_HUMAN] | 1.194 |
| Q8IZH2 | *XRN1* | Isoform 2 of 5'-3' exoribonuclease 1 OS=Homo sapiens GN=XRN1 - [XRN1_HUMAN] | 0.776 |
| O75439 | *PMPCB* | Mitochondrial-processing peptidase subunit beta OS=Homo sapiens GN=PMPCB PE=1 SV=2 - [MPPB_HUMAN] | 1.219 |
| P61026 | *RAB10* | Ras-related protein Rab-10 OS=Homo sapiens GN=RAB10 PE=1 SV=1 - [RAB10_HUMAN] | 1.186 |
| Q9Y5S2 | *CDC42BPB* | Serine/threonine-protein kinase MRCK beta OS=Homo sapiens GN=CDC42BPB PE=1 SV=2 - [MRCKB_HUMAN] | 1.086 |
| Q8NG27 | *PJA1* | Isoform 2 of E3 ubiquitin-protein ligase Praja-1 OS=Homo sapiens GN=PJA1 - [PJA1_HUMAN] | 0.661 |
| P27348 | *YWHAQ* | 14-3-3 protein theta OS=Homo sapiens GN=YWHAQ PE=1 SV=1 - [1433T_HUMAN] | 1.161 |
| Q9Y2H0 | *DLGAP4* | Isoform 3 of Disks large-associated protein 4 OS=Homo sapiens GN=DLGAP4 - [DLGP4_HUMAN] | 1.324 |
| Q9HC78 | *ZBTB20* | Isoform 2 of Zinc finger and BTB domain-containing protein 20 OS=Homo sapiens GN=ZBTB20 - [ZBT20_HUMAN] | 0.756 |
| Q15599 | *SLC9A3R2* | Na(+)/H(+) exchange regulatory cofactor NHE-RF2 OS=Homo sapiens GN=SLC9A3R2 PE=1 SV=2 - [NHRF2_HUMAN] | 0.784 |
| P48147 | *PREP* | Prolyl endopeptidase OS=Homo sapiens GN=PREP PE=1 SV=2 - [PPCE_HUMAN] | 1.305 |
| P02787 | *TF* | Serotransferrin OS=Homo sapiens GN=TF PE=1 SV=3 - [TRFE_HUMAN] | 0.707 |
| Q00688 | *FKBP3* | Peptidyl-prolyl cis-trans isomerase FKBP3 OS=Homo sapiens GN=FKBP3 PE=1 SV=1 - [FKBP3_HUMAN] | 1.246 |
| Q96HE7 | *ERO1L* | ERO1-like protein alpha OS=Homo sapiens GN=ERO1L PE=1 SV=2 - [ERO1A_HUMAN] | 1.606 |
| P31323 | *PRKAR2B* | cAMP-dependent protein kinase type II-beta regulatory subunit OS=Homo sapiens GN=PRKAR2B PE=1 SV=3 - [KAP3_HUMAN] | 0.749 |
| Q9ULA0 | *DNPEP* | Aspartyl aminopeptidase OS=Homo sapiens GN=DNPEP PE=1 SV=1 - [DNPEP_HUMAN] | 1.274 |
| Q9ULR0 | *ISY1* | Pre-mRNA-splicing factor ISY1 homolog OS=Homo sapiens GN=ISY1 PE=1 SV=3 - [ISY1_HUMAN] | 1.105 |
| P51452 | *DUSP3* | Dual specificity protein phosphatase 3 OS=Homo sapiens GN=DUSP3 PE=1 SV=1 - [DUS3_HUMAN] | 0.807 |
| Q5T4S7 | *UBR4* | Isoform 3 of E3 ubiquitin-protein ligase UBR4 OS=Homo sapiens GN=UBR4 - [UBR4_HUMAN] | 1.139 |
| Q9Y5P6 | *GMPPB* | Mannose-1-phosphate guanyltransferase beta OS=Homo sapiens GN=GMPPB PE=1 SV=2 - [GMPPB_HUMAN] | 0.837 |
| Q86UX6 | *STK32C* | Isoform 2 of Serine/threonine-protein kinase 32C OS=Homo sapiens GN=STK32C - [ST32C_HUMAN] | 1.395 |
| Q14118 | *DAG1* | Dystroglycan OS=Homo sapiens GN=DAG1 PE=1 SV=2 - [DAG1_HUMAN] | 0.651 |
| P02768 | *ALB* | Serum albumin OS=Homo sapiens GN=ALB PE=1 SV=2 - [ALBU_HUMAN] | 0.644 |
| P04430 | *#N/A* | Ig kappa chain V-I region BAN OS=Homo sapiens PE=1 SV=1 - [KV122_HUMAN] | 0.754 |
| P0CB43 | *#N/A* | Protein FAM203B OS=Homo sapiens GN=FAM203B PE=3 SV=1 - [F203B_HUMAN] | 1.264 |
| Q96BS2 | *TESC* | Isoform 2 of Tescalcin OS=Homo sapiens GN=TESC - [TESC_HUMAN] | 0.511 |
| Q9NZU5 | *LMCD1* | LIM and cysteine-rich domains protein 1 OS=Homo sapiens GN=LMCD1 PE=1 SV=1 - [LMCD1_HUMAN] | 1.416 |
| O95202 | *LETM1* | LETM1 and EF-hand domain-containing protein 1, mitochondrial OS=Homo sapiens GN=LETM1 PE=1 SV=1 - [LETM1_HUMAN] | 1.326 |
| Q92499 | *DDX1* | ATP-dependent RNA helicase DDX1 OS=Homo sapiens GN=DDX1 PE=1 SV=2 - [DDX1_HUMAN] | 0.877 |
| Q5T0N5 | *FNBP1L* | Isoform 3 of Formin-binding protein 1-like OS=Homo sapiens GN=FNBP1L - [FBP1L_HUMAN] | 1.409 |
| Q7Z4W1 | *DCXR* | L-xylulose reductase OS=Homo sapiens GN=DCXR PE=1 SV=2 - [DCXR_HUMAN] | 0.747 |
| P49768 | *PSEN1* | Isoform 3 of Presenilin-1 OS=Homo sapiens GN=PSEN1 - [PSN1_HUMAN] | 1.230 |
| P07339 | *CTSD* | Cathepsin D OS=Homo sapiens GN=CTSD PE=1 SV=1 - [CATD_HUMAN] | 0.763 |
| Q9UIJ7 | *AK3* | GTP:AMP phosphotransferase, mitochondrial OS=Homo sapiens GN=AK3 PE=1 SV=4 - [KAD3_HUMAN] | 0.855 |
| O60879 | *DIAPH2* | Isoform 2 of Protein diaphanous homolog 2 OS=Homo sapiens GN=DIAPH2 - [DIAP2_HUMAN] | 0.659 |
| Q9Y5A9 | *YTHDF2* | Isoform 2 of YTH domain family protein 2 OS=Homo sapiens GN=YTHDF2 - [YTHD2_HUMAN] | 1.299 |
| Q9H3S7 | *PTPN23* | Tyrosine-protein phosphatase non-receptor type 23 OS=Homo sapiens GN=PTPN23 PE=1 SV=1 - [PTN23_HUMAN] | 0.869 |
| P07093 | *SERPINE2* | Isoform 2 of Glia-derived nexin OS=Homo sapiens GN=SERPINE2 - [GDN_HUMAN] | 0.278 |
| O14802 | *POLR3A* | DNA-directed RNA polymerase III subunit RPC1 OS=Homo sapiens GN=POLR3A PE=1 SV=2 - [RPC1_HUMAN] | 1.401 |
| Q14956 | *GPNMB* | Isoform 2 of Transmembrane glycoprotein NMB OS=Homo sapiens GN=GPNMB - [GPNMB_HUMAN] | 0.666 |
| O60563 | *CCNT1* | Cyclin-T1 OS=Homo sapiens GN=CCNT1 PE=1 SV=1 - [CCNT1_HUMAN] | 0.761 |
| Q6PCE3 | *PGM2L1* | Glucose 1,6-bisphosphate synthase OS=Homo sapiens GN=PGM2L1 PE=1 SV=3 - [PGM2L_HUMAN] | 2.064 |
| P07099 | *EPHX1* | Epoxide hydrolase 1 OS=Homo sapiens GN=EPHX1 PE=1 SV=1 - [HYEP_HUMAN] | 0.726 |
| Q5JSH3 | *WDR44* | Isoform 2 of WD repeat-containing protein 44 OS=Homo sapiens GN=WDR44 - [WDR44_HUMAN] | 1.098 |
| Q9P035 | *PTPLAD1* | 3-hydroxyacyl-CoA dehydratase 3 OS=Homo sapiens GN=PTPLAD1 PE=1 SV=2 - [HACD3_HUMAN] | 1.213 |
| O75094 | *SLIT3* | Isoform 3 of Slit homolog 3 protein OS=Homo sapiens GN=SLIT3 - [SLIT3_HUMAN] | 0.732 |
| P52597 | *HNRNPF* | Heterogeneous nuclear ribonucleoprotein F OS=Homo sapiens GN=HNRNPF PE=1 SV=3 - [HNRPF_HUMAN] | 1.176 |
| Q8TC07 | *TBC1D15* | Isoform 2 of TBC1 domain family member 15 OS=Homo sapiens GN=TBC1D15 - [TBC15_HUMAN] | 0.912 |
| Q13042 | *CDC16* | Isoform 3 of Cell division cycle protein 16 homolog OS=Homo sapiens GN=CDC16 - [CDC16_HUMAN] | 1.113 |
| P02747 | *C1QC* | Complement C1q subcomponent subunit C OS=Homo sapiens GN=C1QC PE=1 SV=3 - [C1QC_HUMAN] | 0.703 |
| P11142 | *HSPA8* | Heat shock cognate 71 kDa protein OS=Homo sapiens GN=HSPA8 PE=1 SV=1 - [HSP7C_HUMAN] | 1.163 |
| Q96KP4 | *CNDP2* | Isoform 2 of Cytosolic non-specific dipeptidase OS=Homo sapiens GN=CNDP2 - [CNDP2_HUMAN] | 0.762 |
| Q92974 | *ARHGEF2* | Isoform 3 of Rho guanine nucleotide exchange factor 2 OS=Homo sapiens GN=ARHGEF2 - [ARHG2_HUMAN] | 1.366 |
| Q9Y3T9 | *NOC2L* | Nucleolar complex protein 2 homolog OS=Homo sapiens GN=NOC2L PE=1 SV=4 - [NOC2L_HUMAN] | 1.263 |
| O60341 | *KDM1A* | Lysine-specific histone demethylase 1A OS=Homo sapiens GN=KDM1A PE=1 SV=2 - [KDM1A_HUMAN] | 1.255 |
| P62857 | *RPS28* | 40S ribosomal protein S28 OS=Homo sapiens GN=RPS28 PE=1 SV=1 - [RS28_HUMAN] | 1.108 |
| Q1KMD3 | *HNRNPUL2* | Heterogeneous nuclear ribonucleoprotein U-like protein 2 OS=Homo sapiens GN=HNRNPUL2 PE=1 SV=1 - [HNRL2_HUMAN] | 1.224 |
| P02774 | *GC* | Vitamin D-binding protein OS=Homo sapiens GN=GC PE=1 SV=1 - [VTDB_HUMAN] | 0.648 |
| Q99439 | *CNN2* | Calponin-2 OS=Homo sapiens GN=CNN2 PE=1 SV=4 - [CNN2_HUMAN] | 1.768 |
| Q14997 | *PSME4* | Proteasome activator complex subunit 4 OS=Homo sapiens GN=PSME4 PE=1 SV=2 - [PSME4_HUMAN] | 1.267 |
| P09917 | *ALOX5* | Arachidonate 5-lipoxygenase OS=Homo sapiens GN=ALOX5 PE=1 SV=2 - [LOX5_HUMAN] | 0.729 |
| Q9BRX2 | *PELO* | Protein pelota homolog OS=Homo sapiens GN=PELO PE=1 SV=2 - [PELO_HUMAN] | 0.798 |
| Q9BRG1 | *VPS25* | Vacuolar protein-sorting-associated protein 25 OS=Homo sapiens GN=VPS25 PE=1 SV=1 - [VPS25_HUMAN] | 1.152 |
| Q15811 | *ITSN1* | Isoform 3 of Intersectin-1 OS=Homo sapiens GN=ITSN1 - [ITSN1_HUMAN] | 1.520 |
| O15066 | *KIF3B* | Kinesin-like protein KIF3B OS=Homo sapiens GN=KIF3B PE=1 SV=1 - [KIF3B_HUMAN] | 1.169 |
| Q9Y624 | *F11R* | Junctional adhesion molecule A OS=Homo sapiens GN=F11R PE=1 SV=1 - [JAM1_HUMAN] | 0.806 |
| P02753 | *RBP4* | Retinol-binding protein 4 OS=Homo sapiens GN=RBP4 PE=1 SV=3 - [RET4_HUMAN] | 0.749 |
| P06241 | *FYN* | Isoform 3 of Tyrosine-protein kinase Fyn OS=Homo sapiens GN=FYN - [FYN_HUMAN] | 0.838 |
| P78406 | *RAE1* | mRNA export factor OS=Homo sapiens GN=RAE1 PE=1 SV=1 - [RAE1L_HUMAN] | 0.868 |
| Q15031 | *LARS2* | Probable leucine--tRNA ligase, mitochondrial OS=Homo sapiens GN=LARS2 PE=1 SV=2 - [SYLM_HUMAN] | 0.876 |
| P29144 | *TPP2* | Tripeptidyl-peptidase 2 OS=Homo sapiens GN=TPP2 PE=1 SV=4 - [TPP2_HUMAN] | 1.197 |
| Q92896 | *GLG1* | Golgi apparatus protein 1 OS=Homo sapiens GN=GLG1 PE=1 SV=2 - [GSLG1_HUMAN] | 0.838 |
| Q9NZJ9 | *NUDT4* | Diphosphoinositol polyphosphate phosphohydrolase 2 OS=Homo sapiens GN=NUDT4 PE=1 SV=2 - [NUDT4_HUMAN] | 0.693 |
| P62834 | *RAP1A* | Ras-related protein Rap-1A OS=Homo sapiens GN=RAP1A PE=1 SV=1 - [RAP1A_HUMAN] | 0.728 |
| Q9H3K6 | *BOLA2* | Isoform 2 of BolA-like protein 2 OS=Homo sapiens GN=BOLA2 - [BOLA2_HUMAN] | 1.398 |
| Q16623 | *STX1A* | Syntaxin-1A OS=Homo sapiens GN=STX1A PE=1 SV=1 - [STX1A_HUMAN] | 0.682 |
| Q8IU81 | *IRF2BP1* | Interferon regulatory factor 2-binding protein 1 OS=Homo sapiens GN=IRF2BP1 PE=1 SV=1 - [I2BP1_HUMAN] | 1.182 |
| Q9GZP4 | *PITHD1* | Isoform 2 of PITH domain-containing protein 1 OS=Homo sapiens GN=PITHD1 - [PITH1_HUMAN] | 0.813 |
| P62979 | *RPS27A* | Ubiquitin-40S ribosomal protein S27a OS=Homo sapiens GN=RPS27A PE=1 SV=2 - [RS27A_HUMAN] | 0.881 |
| Q9Y6D6 | *ARFGEF1* | Brefeldin A-inhibited guanine nucleotide-exchange protein 1 OS=Homo sapiens GN=ARFGEF1 PE=1 SV=2 - [BIG1_HUMAN] | 1.276 |
| P14868 | *DARS* | Aspartate--tRNA ligase, cytoplasmic OS=Homo sapiens GN=DARS PE=1 SV=2 - [SYDC_HUMAN] | 1.145 |
| P25098 | *ADRBK1* | Beta-adrenergic receptor kinase 1 OS=Homo sapiens GN=ADRBK1 PE=1 SV=2 - [ARBK1_HUMAN] | 1.217 |
| P24310 | *COX7A1* | Cytochrome c oxidase subunit 7A1, mitochondrial OS=Homo sapiens GN=COX7A1 PE=1 SV=2 - [CX7A1_HUMAN] | 0.443 |
| Q9Y5W8 | *SNX13* | Isoform 2 of Sorting nexin-13 OS=Homo sapiens GN=SNX13 - [SNX13_HUMAN] | 1.192 |
| Q9UKU7 | *ACAD8* | Isobutyryl-CoA dehydrogenase, mitochondrial OS=Homo sapiens GN=ACAD8 PE=1 SV=1 - [ACAD8_HUMAN] | 0.730 |
| P08133 | *ANXA6* | Annexin A6 OS=Homo sapiens GN=ANXA6 PE=1 SV=3 - [ANXA6_HUMAN] | 0.781 |
| P01008 | *SERPINC1* | Antithrombin-III OS=Homo sapiens GN=SERPINC1 PE=1 SV=1 - [ANT3_HUMAN] | 0.557 |
| Q9NW13 | *RBM28* | RNA-binding protein 28 OS=Homo sapiens GN=RBM28 PE=1 SV=3 - [RBM28_HUMAN] | 1.242 |
| Q96E17 | *RAB3C* | Ras-related protein Rab-3C OS=Homo sapiens GN=RAB3C PE=2 SV=1 - [RAB3C_HUMAN] | 0.590 |
| O95989 | *NUDT3* | Diphosphoinositol polyphosphate phosphohydrolase 1 OS=Homo sapiens GN=NUDT3 PE=1 SV=1 - [NUDT3_HUMAN] | 1.181 |
| Q9Y3F4 | *STRAP* | Serine-threonine kinase receptor-associated protein OS=Homo sapiens GN=STRAP PE=1 SV=1 - [STRAP_HUMAN] | 1.105 |
| Q8WX93 | *PALLD* | Isoform 4 of Palladin OS=Homo sapiens GN=PALLD - [PALLD_HUMAN] | 1.580 |
| Q9UKJ3 | *GPATCH8* | Isoform 2 of G patch domain-containing protein 8 OS=Homo sapiens GN=GPATCH8 - [GPTC8_HUMAN] | 1.121 |
| Q6P161 | *MRPL54* | 39S ribosomal protein L54, mitochondrial OS=Homo sapiens GN=MRPL54 PE=1 SV=1 - [RM54_HUMAN] | 0.868 |
| Q12959 | *DLG1* | Isoform 5 of Disks large homolog 1 OS=Homo sapiens GN=DLG1 - [DLG1_HUMAN] | 0.796 |
| Q96BI3 | *APH1A* | Isoform 2 of Gamma-secretase subunit APH-1A OS=Homo sapiens GN=APH1A - [APH1A_HUMAN] | 1.323 |
| Q8TEA8 | *DTD1* | D-tyrosyl-tRNA(Tyr) deacylase 1 OS=Homo sapiens GN=DTD1 PE=1 SV=2 - [DTD1_HUMAN] | 0.885 |
| P41219 | *PRPH* | Peripherin OS=Homo sapiens GN=PRPH PE=1 SV=2 - [PERI_HUMAN] | 0.167 |
| P21359 | *NF1* | Isoform 1 of Neurofibromin OS=Homo sapiens GN=NF1 - [NF1_HUMAN] | 0.638 |
| Q9H0U3 | *MAGT1* | Magnesium transporter protein 1 OS=Homo sapiens GN=MAGT1 PE=1 SV=1 - [MAGT1_HUMAN] | 0.846 |
| Q5T3U5 | *ABCC10* | Isoform 2 of Multidrug resistance-associated protein 7 OS=Homo sapiens GN=ABCC10 - [MRP7_HUMAN] | 1.819 |
| Q92785 | *DPF2* | Zinc finger protein ubi-d4 OS=Homo sapiens GN=DPF2 PE=1 SV=2 - [REQU_HUMAN] | 1.262 |
| Q9BV57 | *ADI1* | Isoform 2 of 1,2-dihydroxy-3-keto-5-methylthiopentene dioxygenase OS=Homo sapiens GN=ADI1 - [MTND_HUMAN] | 1.577 |
| P54727 | *RAD23B* | UV excision repair protein RAD23 homolog B OS=Homo sapiens GN=RAD23B PE=1 SV=1 - [RD23B_HUMAN] | 1.128 |
| Q13103 | *SPP2* | Secreted phosphoprotein 24 OS=Homo sapiens GN=SPP2 PE=1 SV=1 - [SPP24_HUMAN] | 0.281 |
| Q02127 | *DHODH* | Dihydroorotate dehydrogenase (quinone), mitochondrial OS=Homo sapiens GN=DHODH PE=1 SV=3 - [PYRD_HUMAN] | 0.822 |
| P26640 | *VARS* | Valine--tRNA ligase OS=Homo sapiens GN=VARS PE=1 SV=4 - [SYVC_HUMAN] | 1.182 |
| P13671 | *C6* | Complement component C6 OS=Homo sapiens GN=C6 PE=1 SV=3 - [CO6_HUMAN] | 0.647 |
| Q9P0K7 | *RAI14* | Isoform 3 of Ankycorbin OS=Homo sapiens GN=RAI14 - [RAI14_HUMAN] | 0.861 |
| Q9NX58 | *LYAR* | Cell growth-regulating nucleolar protein OS=Homo sapiens GN=LYAR PE=1 SV=2 - [LYAR_HUMAN] | 1.181 |
| O60884 | *DNAJA2* | DnaJ homolog subfamily A member 2 OS=Homo sapiens GN=DNAJA2 PE=1 SV=1 - [DNJA2_HUMAN] | 1.216 |
| Q9Y259 | *CHKB* | Choline/ethanolamine kinase OS=Homo sapiens GN=CHKB PE=1 SV=3 - [CHKB_HUMAN] | 1.345 |
| Q9P289 | *STK26* | Isoform 2 of Serine/threonine-protein kinase MST4 OS=Homo sapiens GN=MST4 - [MST4_HUMAN] | 0.803 |
| O60888 | *CUTA* | Isoform C of Protein CutA OS=Homo sapiens GN=CUTA - [CUTA_HUMAN] | 0.830 |
| Q9UJX3 | *ANAPC7* | Isoform 2 of Anaphase-promoting complex subunit 7 OS=Homo sapiens GN=ANAPC7 - [APC7_HUMAN] | 0.876 |
| Q96EP5 | *DAZAP1* | Isoform 2 of DAZ-associated protein 1 OS=Homo sapiens GN=DAZAP1 - [DAZP1_HUMAN] | 1.182 |
| Q9NVA2 | *SEPT11* | Septin-11 OS=Homo sapiens GN=SEPT11 PE=1 SV=3 - [SEP11_HUMAN] | 1.290 |
| P01258 | *CALCA* | Calcitonin OS=Homo sapiens GN=CALCA PE=1 SV=2 - [CALC_HUMAN] | 0.558 |
| P28340 | *POLD1* | DNA polymerase delta catalytic subunit OS=Homo sapiens GN=POLD1 PE=1 SV=2 - [DPOD1_HUMAN] | 1.223 |
| P49407 | *ARRB1* | Isoform 1B of Beta-arrestin-1 OS=Homo sapiens GN=ARRB1 - [ARRB1_HUMAN] | 1.268 |
| O75923 | *DYSF* | Isoform 15 of Dysferlin OS=Homo sapiens GN=DYSF - [DYSF_HUMAN] | 0.713 |
| Q6ZUT6 | *C15orf52* | Uncharacterized protein C15orf52 OS=Homo sapiens GN=C15orf52 PE=1 SV=1 - [CO052_HUMAN] | 0.751 |
| Q13885 | *TUBB2A* | Tubulin beta-2A chain OS=Homo sapiens GN=TUBB2A PE=1 SV=1 - [TBB2A_HUMAN] | 1.185 |
| Q9H0D6 | *XRN2* | Isoform 2 of 5'-3' exoribonuclease 2 OS=Homo sapiens GN=XRN2 - [XRN2_HUMAN] | 1.076 |
| P49588 | *AARS* | Alanine--tRNA ligase, cytoplasmic OS=Homo sapiens GN=AARS PE=1 SV=2 - [SYAC_HUMAN] | 1.230 |
| P33316 | *DUT* | Isoform 2 of Deoxyuridine 5'-triphosphate nucleotidohydrolase, mitochondrial OS=Homo sapiens GN=DUT - [DUT_HUMAN] | 0.827 |
| P04066 | *FUCA1* | Tissue alpha-L-fucosidase OS=Homo sapiens GN=FUCA1 PE=1 SV=4 - [FUCO_HUMAN] | 0.653 |
| P55039 | *DRG2* | Developmentally-regulated GTP-binding protein 2 OS=Homo sapiens GN=DRG2 PE=1 SV=1 - [DRG2_HUMAN] | 0.850 |
| O14791 | *APOL1* | Apolipoprotein L1 OS=Homo sapiens GN=APOL1 PE=1 SV=5 - [APOL1_HUMAN] | 0.567 |
| Q9P0J7 | *KCMF1* | E3 ubiquitin-protein ligase KCMF1 OS=Homo sapiens GN=KCMF1 PE=1 SV=2 - [KCMF1_HUMAN] | 1.137 |
| P49821 | *NDUFV1* | Isoform 2 of NADH dehydrogenase [ubiquinone] flavoprotein 1, mitochondrial OS=Homo sapiens GN=NDUFV1 - [NDUV1_HUMAN] | 0.825 |
| Q92643 | *PIGK* | GPI-anchor transamidase OS=Homo sapiens GN=PIGK PE=1 SV=2 - [GPI8_HUMAN] | 1.293 |
| P12694 | *BCKDHA* | 2-oxoisovalerate dehydrogenase subunit alpha, mitochondrial OS=Homo sapiens GN=BCKDHA PE=1 SV=2 - [ODBA_HUMAN] | 0.798 |
| Q9BTW9 | *TBCD* | Tubulin-specific chaperone D OS=Homo sapiens GN=TBCD PE=1 SV=2 - [TBCD_HUMAN] | 1.305 |
| Q5JTD0 | *TJAP1* | Isoform 4 of Tight junction-associated protein 1 OS=Homo sapiens GN=TJAP1 - [TJAP1_HUMAN] | 1.277 |
| Q8NDH3 | *NPEPL1* | Isoform 2 of Probable aminopeptidase NPEPL1 OS=Homo sapiens GN=NPEPL1 - [PEPL1_HUMAN] | 1.495 |
| Q9NZ01 | *TECR* | Trans-2,3-enoyl-CoA reductase OS=Homo sapiens GN=TECR PE=1 SV=1 - [TECR_HUMAN] | 0.849 |
| P49006 | *MARCKSL1* | MARCKS-related protein OS=Homo sapiens GN=MARCKSL1 PE=1 SV=2 - [MRP_HUMAN] | 1.571 |
| Q7L775 | *EPM2AIP1* | EPM2A-interacting protein 1 OS=Homo sapiens GN=EPM2AIP1 PE=1 SV=1 - [EPMIP_HUMAN] | 0.789 |
| Q9H115 | *NAPB* | Beta-soluble NSF attachment protein OS=Homo sapiens GN=NAPB PE=1 SV=2 - [SNAB_HUMAN] | 0.837 |
| P61289 | *PSME3* | Proteasome activator complex subunit 3 OS=Homo sapiens GN=PSME3 PE=1 SV=1 - [PSME3_HUMAN] | 1.254 |
| P19388 | *POLR2E* | DNA-directed RNA polymerases I, II, and III subunit RPABC1 OS=Homo sapiens GN=POLR2E PE=1 SV=4 - [RPAB1_HUMAN] | 1.130 |
| P42892 | *ECE1* | Isoform C of Endothelin-converting enzyme 1 OS=Homo sapiens GN=ECE1 - [ECE1_HUMAN] | 0.784 |
| Q14690 | *PDCD11* | Protein RRP5 homolog OS=Homo sapiens GN=PDCD11 PE=1 SV=3 - [RRP5_HUMAN] | 1.227 |
| Q15118 | *PDK1* | [Pyruvate dehydrogenase [lipoamide]] kinase isozyme 1, mitochondrial OS=Homo sapiens GN=PDK1 PE=1 SV=1 - [PDK1_HUMAN] | 1.820 |
| Q15836 | *VAMP3* | Vesicle-associated membrane protein 3 OS=Homo sapiens GN=VAMP3 PE=1 SV=3 - [VAMP3_HUMAN] | 0.824 |
| P24539 | *ATP5F1* | ATP synthase subunit b, mitochondrial OS=Homo sapiens GN=ATP5F1 PE=1 SV=2 - [AT5F1_HUMAN] | 0.771 |
| Q15418 | *RPS6KA1* | Isoform 3 of Ribosomal protein S6 kinase alpha-1 OS=Homo sapiens GN=RPS6KA1 - [KS6A1_HUMAN] | 1.643 |
| Q4G176 | *ACSF3* | Acyl-CoA synthetase family member 3, mitochondrial OS=Homo sapiens GN=ACSF3 PE=1 SV=3 - [ACSF3_HUMAN] | 0.855 |
| P61960 | *UFM1* | Ubiquitin-fold modifier 1 OS=Homo sapiens GN=UFM1 PE=1 SV=1 - [UFM1_HUMAN] | 0.819 |
| Q13308 | *PTK7* | Isoform 3 of Inactive tyrosine-protein kinase 7 OS=Homo sapiens GN=PTK7 - [PTK7_HUMAN] | 1.509 |
| Q14168 | *MPP2* | Isoform 2 of MAGUK p55 subfamily member 2 OS=Homo sapiens GN=MPP2 - [MPP2_HUMAN] | 0.732 |
| Q96T76 | *MMS19* | Isoform 2 of MMS19 nucleotide excision repair protein homolog OS=Homo sapiens GN=MMS19 - [MMS19_HUMAN] | 1.281 |
| P00450 | *CP* | Ceruloplasmin OS=Homo sapiens GN=CP PE=1 SV=1 - [CERU_HUMAN] | 0.671 |
| P22413 | *ENPP1* | Ectonucleotide pyrophosphatase/phosphodiesterase family member 1 OS=Homo sapiens GN=ENPP1 PE=1 SV=2 - [ENPP1_HUMAN] | 0.414 |
| Q9C0E8 | *KIAA1715* | Isoform 2 of Protein lunapark OS=Homo sapiens GN=LNP - [LNP_HUMAN] | 1.204 |
| O95749 | *GGPS1* | Geranylgeranyl pyrophosphate synthase OS=Homo sapiens GN=GGPS1 PE=1 SV=1 - [GGPPS_HUMAN] | 1.270 |
| O75844 | *ZMPSTE24* | CAAX prenyl protease 1 homolog OS=Homo sapiens GN=ZMPSTE24 PE=1 SV=2 - [FACE1_HUMAN] | 1.200 |
| O15021 | *MAST4* | Isoform 2 of Microtubule-associated serine/threonine-protein kinase 4 OS=Homo sapiens GN=MAST4 - [MAST4_HUMAN] | 0.517 |
| Q8IX30 | *SCUBE3* | Signal peptide, CUB and EGF-like domain-containing protein 3 OS=Homo sapiens GN=SCUBE3 PE=1 SV=1 - [SCUB3_HUMAN] | 0.454 |
| Q9BYC8 | *MRPL32* | 39S ribosomal protein L32, mitochondrial OS=Homo sapiens GN=MRPL32 PE=1 SV=1 - [RM32_HUMAN] | 0.906 |
| Q16762 | *TST* | Thiosulfate sulfurtransferase OS=Homo sapiens GN=TST PE=1 SV=4 - [THTR_HUMAN] | 0.582 |
| P02746 | *C1QB* | Complement C1q subcomponent subunit B OS=Homo sapiens GN=C1QB PE=1 SV=3 - [C1QB_HUMAN] | 0.731 |
| Q92890 | *UFD1L* | Ubiquitin fusion degradation protein 1 homolog OS=Homo sapiens GN=UFD1L PE=1 SV=3 - [UFD1_HUMAN] | 1.109 |
| Q96MK3 | *FAM20A* | Protein FAM20A OS=Homo sapiens GN=FAM20A PE=1 SV=4 - [FA20A_HUMAN] | 0.495 |
| Q9BSG5 | *RTBDN* | Retbindin OS=Homo sapiens GN=RTBDN PE=2 SV=2 - [RTBDN_HUMAN] | 0.695 |
| P0C0L4 | *C4A* | Complement C4-A OS=Homo sapiens GN=C4A PE=1 SV=1 - [CO4A_HUMAN] | 0.399 |
| Q5JPH6 | *EARS2* | Probable glutamate--tRNA ligase, mitochondrial OS=Homo sapiens GN=EARS2 PE=1 SV=2 - [SYEM_HUMAN] | 0.826 |
| O95810 | *SDPR* | Serum deprivation-response protein OS=Homo sapiens GN=SDPR PE=1 SV=3 - [SDPR_HUMAN] | 0.336 |
| P16152 | *CBR1* | Carbonyl reductase [NADPH] 1 OS=Homo sapiens GN=CBR1 PE=1 SV=3 - [CBR1_HUMAN] | 1.369 |
| Q07021 | *C1QBP* | Complement component 1 Q subcomponent-binding protein, mitochondrial OS=Homo sapiens GN=C1QBP PE=1 SV=1 - [C1QBP_HUMAN] | 0.812 |
| Q8TED1 | *GPX8* | Probable glutathione peroxidase 8 OS=Homo sapiens GN=GPX8 PE=1 SV=2 - [GPX8_HUMAN] | 1.670 |
| O95433 | *AHSA1* | Activator of 90 kDa heat shock protein ATPase homolog 1 OS=Homo sapiens GN=AHSA1 PE=1 SV=1 - [AHSA1_HUMAN] | 1.215 |
| P63167 | *DYNLL1* | Dynein light chain 1, cytoplasmic OS=Homo sapiens GN=DYNLL1 PE=1 SV=1 - [DYL1_HUMAN] | 0.843 |
| Q5H9R7 | *PPP6R3* | Isoform 3 of Serine/threonine-protein phosphatase 6 regulatory subunit 3 OS=Homo sapiens GN=PPP6R3 - [PP6R3_HUMAN] | 1.238 |
| Q9P258 | *RCC2* | Protein RCC2 OS=Homo sapiens GN=RCC2 PE=1 SV=2 - [RCC2_HUMAN] | 1.346 |
| P27144 | *AK4* | Adenylate kinase isoenzyme 4, mitochondrial OS=Homo sapiens GN=AK4 PE=1 SV=1 - [KAD4_HUMAN] | 0.660 |
| Q53GG5 | *PDLIM3* | Isoform 2 of PDZ and LIM domain protein 3 OS=Homo sapiens GN=PDLIM3 - [PDLI3_HUMAN] | 1.663 |
| Q86WR0 | *CCDC25* | Coiled-coil domain-containing protein 25 OS=Homo sapiens GN=CCDC25 PE=1 SV=2 - [CCD25_HUMAN] | 0.749 |
| Q15063 | *POSTN* | Isoform 3 of Periostin OS=Homo sapiens GN=POSTN - [POSTN_HUMAN] | 3.431 |
| Q9BR76 | *CORO1B* | Coronin-1B OS=Homo sapiens GN=CORO1B PE=1 SV=1 - [COR1B_HUMAN] | 1.326 |
| Q9NWT1 | *PAK1IP1* | p21-activated protein kinase-interacting protein 1 OS=Homo sapiens GN=PAK1IP1 PE=1 SV=2 - [PK1IP_HUMAN] | 1.540 |
| P62879 | *GNB2* | Guanine nucleotide-binding protein G(I)/G(S)/G(T) subunit beta-2 OS=Homo sapiens GN=GNB2 PE=1 SV=3 - [GBB2_HUMAN] | 0.730 |
| P20962 | *PTMS* | Parathymosin OS=Homo sapiens GN=PTMS PE=1 SV=2 - [PTMS_HUMAN] | 1.295 |
| O15439 | *ABCC4* | Isoform 2 of Multidrug resistance-associated protein 4 OS=Homo sapiens GN=ABCC4 - [MRP4_HUMAN] | 0.645 |
| Q9Y6G9 | *DYNC1LI1* | Cytoplasmic dynein 1 light intermediate chain 1 OS=Homo sapiens GN=DYNC1LI1 PE=1 SV=3 - [DC1L1_HUMAN] | 0.854 |
| Q9H6K4 | *OPA3* | Optic atrophy 3 protein OS=Homo sapiens GN=OPA3 PE=1 SV=1 - [OPA3_HUMAN] | 0.846 |
| Q13085 | *ACACA* | Isoform 3 of Acetyl-CoA carboxylase 1 OS=Homo sapiens GN=ACACA - [ACACA_HUMAN] | 1.251 |
| O43824 | *#N/A* | Putative GTP-binding protein 6 OS=Homo sapiens GN=GTPBP6 PE=2 SV=3 - [GTPB6_HUMAN] | 0.778 |
| P42345 | *MTOR* | Serine/threonine-protein kinase mTOR OS=Homo sapiens GN=MTOR PE=1 SV=1 - [MTOR_HUMAN] | 1.133 |
| P61221 | *ABCE1* | ATP-binding cassette sub-family E member 1 OS=Homo sapiens GN=ABCE1 PE=1 SV=1 - [ABCE1_HUMAN] | 1.295 |
| Q99735 | *MGST2* | Microsomal glutathione S-transferase 2 OS=Homo sapiens GN=MGST2 PE=1 SV=1 - [MGST2_HUMAN] | 1.228 |
| P46199 | *MTIF2* | Translation initiation factor IF-2, mitochondrial OS=Homo sapiens GN=MTIF2 PE=1 SV=2 - [IF2M_HUMAN] | 0.850 |
| Q9Y5I2 | *PCDHA10* | Isoform 2 of Protocadherin alpha-10 OS=Homo sapiens GN=PCDHA10 - [PCDAA_HUMAN] | 0.522 |
| Q8TF09 | *DYNLRB2* | Dynein light chain roadblock-type 2 OS=Homo sapiens GN=DYNLRB2 PE=1 SV=1 - [DLRB2_HUMAN] | 1.117 |
| Q8NBX0 | *SCCPDH* | Saccharopine dehydrogenase-like oxidoreductase OS=Homo sapiens GN=SCCPDH PE=1 SV=1 - [SCPDL_HUMAN] | 0.826 |
| Q13449 | *LSAMP* | Limbic system-associated membrane protein OS=Homo sapiens GN=LSAMP PE=1 SV=2 - [LSAMP_HUMAN] | 0.549 |
| Q9GZT9 | *EGLN1* | Isoform 2 of Egl nine homolog 1 OS=Homo sapiens GN=EGLN1 - [EGLN1_HUMAN] | 1.208 |
| Q7L1Q6 | *BZW1* | Basic leucine zipper and W2 domain-containing protein 1 OS=Homo sapiens GN=BZW1 PE=1 SV=1 - [BZW1_HUMAN] | 1.326 |
| P08603 | *CFH* | Complement factor H OS=Homo sapiens GN=CFH PE=1 SV=4 - [CFAH_HUMAN] | 0.697 |
| Q9H8M2 | *BRD9* | Bromodomain-containing protein 9 OS=Homo sapiens GN=BRD9 PE=1 SV=2 - [BRD9_HUMAN] | 1.205 |
| Q9UBP6 | *METTL1* | tRNA (guanine-N(7)-)-methyltransferase OS=Homo sapiens GN=METTL1 PE=1 SV=1 - [TRMB_HUMAN] | 1.280 |
| P31483 | *TIA1* | Isoform Short of Nucleolysin TIA-1 isoform p40 OS=Homo sapiens GN=TIA1 - [TIA1_HUMAN] | 1.197 |
| Q96A35 | *MRPL24* | 39S ribosomal protein L24, mitochondrial OS=Homo sapiens GN=MRPL24 PE=1 SV=1 - [RM24_HUMAN] | 0.900 |
| P56181 | *NDUFV3* | Isoform 2 of NADH dehydrogenase [ubiquinone] flavoprotein 3, mitochondrial OS=Homo sapiens GN=NDUFV3 - [NDUV3_HUMAN] | 1.165 |
| O60231 | *DHX16* | Putative pre-mRNA-splicing factor ATP-dependent RNA helicase DHX16 OS=Homo sapiens GN=DHX16 PE=1 SV=2 - [DHX16_HUMAN] | 1.165 |
| Q8WW12 | *PCNP* | Isoform 2 of PEST proteolytic signal-containing nuclear protein OS=Homo sapiens GN=PCNP - [PCNP_HUMAN] | 0.844 |
| Q96CM8 | *ACSF2* | Acyl-CoA synthetase family member 2, mitochondrial OS=Homo sapiens GN=ACSF2 PE=1 SV=2 - [ACSF2_HUMAN] | 0.220 |
| Q8N5M1 | *ATPAF2* | ATP synthase mitochondrial F1 complex assembly factor 2 OS=Homo sapiens GN=ATPAF2 PE=1 SV=1 - [ATPF2_HUMAN] | 0.823 |
| P22061 | *PCMT1* | Protein-L-isoaspartate(D-aspartate) O-methyltransferase OS=Homo sapiens GN=PCMT1 PE=1 SV=4 - [PIMT_HUMAN] | 0.861 |
| Q07157 | *TJP1* | Isoform Short of Tight junction protein ZO-1 OS=Homo sapiens GN=TJP1 - [ZO1_HUMAN] | 0.821 |
| Q14061 | *COX17* | Cytochrome c oxidase copper chaperone OS=Homo sapiens GN=COX17 PE=1 SV=2 - [COX17_HUMAN] | 0.639 |
| Q6P587 | *FAHD1* | Acylpyruvase FAHD1, mitochondrial OS=Homo sapiens GN=FAHD1 PE=1 SV=2 - [FAHD1_HUMAN] | 0.781 |
| P60842 | *EIF4A1* | Eukaryotic initiation factor 4A-I OS=Homo sapiens GN=EIF4A1 PE=1 SV=1 - [IF4A1_HUMAN] | 1.132 |
| Q8NBJ5 | *COLGALT1* | Procollagen galactosyltransferase 1 OS=Homo sapiens GN=GLT25D1 PE=1 SV=1 - [GT251_HUMAN] | 1.256 |
| Q9Y316 | *MEMO1* | Isoform 2 of Protein MEMO1 OS=Homo sapiens GN=MEMO1 - [MEMO1_HUMAN] | 1.176 |
| P29692 | *EEF1D* | Isoform 2 of Elongation factor 1-delta OS=Homo sapiens GN=EEF1D - [EF1D_HUMAN] | 1.106 |
| P31948 | *STIP1* | Stress-induced-phosphoprotein 1 OS=Homo sapiens GN=STIP1 PE=1 SV=1 - [STIP1_HUMAN] | 1.388 |
| P47897 | *QARS* | Glutamine--tRNA ligase OS=Homo sapiens GN=QARS PE=1 SV=1 - [SYQ_HUMAN] | 1.120 |
| P07357 | *C8A* | Complement component C8 alpha chain OS=Homo sapiens GN=C8A PE=1 SV=2 - [CO8A_HUMAN] | 0.553 |
| Q9UK59 | *DBR1* | Isoform 2 of Lariat debranching enzyme OS=Homo sapiens GN=DBR1 - [DBR1_HUMAN] | 0.790 |
| P14406 | *#N/A* | Cytochrome c oxidase subunit 7A2, mitochondrial OS=Homo sapiens GN=COX7A2 PE=1 SV=1 - [CX7A2_HUMAN] | 0.823 |
| Q14699 | *RFTN1* | Raftlin OS=Homo sapiens GN=RFTN1 PE=1 SV=4 - [RFTN1_HUMAN] | 0.763 |
| P05165 | *PCCA* | Isoform 2 of Propionyl-CoA carboxylase alpha chain, mitochondrial OS=Homo sapiens GN=PCCA - [PCCA_HUMAN] | 0.753 |
| P01042 | *KNG1* | Isoform LMW of Kininogen-1 OS=Homo sapiens GN=KNG1 - [KNG1_HUMAN] | 0.712 |
| O95817 | *BAG3* | BAG family molecular chaperone regulator 3 OS=Homo sapiens GN=BAG3 PE=1 SV=3 - [BAG3_HUMAN] | 1.293 |
| P67812 | *SEC11A* | Signal peptidase complex catalytic subunit SEC11A OS=Homo sapiens GN=SEC11A PE=1 SV=1 - [SC11A_HUMAN] | 1.309 |
| Q9ULC4 | *MCTS1* | Malignant T cell-amplified sequence 1 OS=Homo sapiens GN=MCTS1 PE=1 SV=1 - [MCTS1_HUMAN] | 1.122 |
| Q16849 | *PTPRN* | Receptor-type tyrosine-protein phosphatase-like N OS=Homo sapiens GN=PTPRN PE=1 SV=1 - [PTPRN_HUMAN] | 0.550 |
| Q5QJ74 | *TBCEL* | Tubulin-specific chaperone cofactor E-like protein OS=Homo sapiens GN=TBCEL PE=2 SV=2 - [TBCEL_HUMAN] | 1.145 |
| Q9NRY6 | *TMEM256-PLSCR3* | Phospholipid scramblase 3 OS=Homo sapiens GN=PLSCR3 PE=1 SV=2 - [PLS3_HUMAN] | 0.743 |
| P48739 | *PITPNB* | Phosphatidylinositol transfer protein beta isoform OS=Homo sapiens GN=PITPNB PE=1 SV=2 - [PIPNB_HUMAN] | 1.326 |
| Q70E73 | *RAPH1* | Isoform RMO1c of Ras-associated and pleckstrin homology domains-containing protein 1 OS=Homo sapiens GN=RAPH1 - [RAPH1_HUMAN] | 0.771 |
| P34913 | *EPHX2* | Epoxide hydrolase 2 OS=Homo sapiens GN=EPHX2 PE=1 SV=2 - [HYES_HUMAN] | 0.836 |
| Q96BN8 | *OTULIN* | Protein FAM105B OS=Homo sapiens GN=FAM105B PE=1 SV=3 - [F105B_HUMAN] | 1.221 |
| Q9H4F8 | *SMOC1* | SPARC-related modular calcium-binding protein 1 OS=Homo sapiens GN=SMOC1 PE=1 SV=1 - [SMOC1_HUMAN] | 0.286 |
| P49441 | *INPP1* | Inositol polyphosphate 1-phosphatase OS=Homo sapiens GN=INPP1 PE=1 SV=1 - [INPP_HUMAN] | 1.283 |
| O43747 | *AP1G1* | AP-1 complex subunit gamma-1 OS=Homo sapiens GN=AP1G1 PE=1 SV=5 - [AP1G1_HUMAN] | 1.173 |
| Q96DH6 | *MSI2* | Isoform 2 of RNA-binding protein Musashi homolog 2 OS=Homo sapiens GN=MSI2 - [MSI2H_HUMAN] | 0.871 |
| Q9H9Q2 | *COPS7B* | Isoform 2 of COP9 signalosome complex subunit 7b OS=Homo sapiens GN=COPS7B - [CSN7B_HUMAN] | 1.204 |
| P50225 | *SULT1A1* | Isoform 2 of Sulfotransferase 1A1 OS=Homo sapiens GN=SULT1A1 - [ST1A1_HUMAN] | 0.462 |
| Q9H3N1 | *TMX1* | Thioredoxin-related transmembrane protein 1 OS=Homo sapiens GN=TMX1 PE=1 SV=1 - [TMX1_HUMAN] | 1.097 |
| O00264 | *PGRMC1* | Membrane-associated progesterone receptor component 1 OS=Homo sapiens GN=PGRMC1 PE=1 SV=3 - [PGRC1_HUMAN] | 0.839 |
| Q92520 | *FAM3C* | Protein FAM3C OS=Homo sapiens GN=FAM3C PE=1 SV=1 - [FAM3C_HUMAN] | 0.746 |
| P19256 | *CD58* | Isoform 2 of Lymphocyte function-associated antigen 3 OS=Homo sapiens GN=CD58 - [LFA3_HUMAN] | 0.823 |
| Q15257 | *PPP2R4* | Isoform 4 of Serine/threonine-protein phosphatase 2A activator OS=Homo sapiens GN=PPP2R4 - [PTPA_HUMAN] | 1.160 |
| Q13683 | *ITGA7* | Isoform Alpha-7X2DA of Integrin alpha-7 OS=Homo sapiens GN=ITGA7 - [ITA7_HUMAN] | 0.422 |
| P50552 | *VASP* | Vasodilator-stimulated phosphoprotein OS=Homo sapiens GN=VASP PE=1 SV=3 - [VASP_HUMAN] | 1.213 |
| Q8IXM3 | *MRPL41* | 39S ribosomal protein L41, mitochondrial OS=Homo sapiens GN=MRPL41 PE=1 SV=1 - [RM41_HUMAN] | 0.910 |
| O00560 | *SDCBP* | Isoform 3 of Syntenin-1 OS=Homo sapiens GN=SDCBP - [SDCB1_HUMAN] | 0.571 |
| O15269 | *SPTLC1* | Serine palmitoyltransferase 1 OS=Homo sapiens GN=SPTLC1 PE=1 SV=1 - [SPTC1_HUMAN] | 1.201 |
| Q9H479 | *FN3K* | Fructosamine-3-kinase OS=Homo sapiens GN=FN3K PE=1 SV=1 - [FN3K_HUMAN] | 0.865 |
| Q9BRX8 | *FAM213A* | Isoform 2 of Redox-regulatory protein FAM213A OS=Homo sapiens GN=FAM213A - [F213A_HUMAN] | 1.760 |
| P00505 | *GOT2* | Aspartate aminotransferase, mitochondrial OS=Homo sapiens GN=GOT2 PE=1 SV=3 - [AATM_HUMAN] | 0.841 |
| P01593 | *#N/A* | Ig kappa chain V-I region AG OS=Homo sapiens PE=1 SV=1 - [KV101_HUMAN] | 0.735 |
| P57076 | *C21orf59* | Uncharacterized protein C21orf59 OS=Homo sapiens GN=C21orf59 PE=1 SV=1 - [CU059_HUMAN] | 1.166 |
| P30046 | *DDT* | D-dopachrome decarboxylase OS=Homo sapiens GN=DDT PE=1 SV=3 - [DOPD_HUMAN] | 1.584 |
| Q92625 | *ANKS1A* | Ankyrin repeat and SAM domain-containing protein 1A OS=Homo sapiens GN=ANKS1A PE=1 SV=4 - [ANS1A_HUMAN] | 1.246 |
| Q9BSH5 | *HDHD3* | Haloacid dehalogenase-like hydrolase domain-containing protein 3 OS=Homo sapiens GN=HDHD3 PE=1 SV=1 - [HDHD3_HUMAN] | 0.698 |
| Q9NVH6 | *TMLHE* | Isoform B of Trimethyllysine dioxygenase, mitochondrial OS=Homo sapiens GN=TMLHE - [TMLH_HUMAN] | 0.783 |
| P25789 | *PSMA4* | Proteasome subunit alpha type-4 OS=Homo sapiens GN=PSMA4 PE=1 SV=1 - [PSA4_HUMAN] | 1.129 |
| Q14008 | *CKAP5* | Isoform 2 of Cytoskeleton-associated protein 5 OS=Homo sapiens GN=CKAP5 - [CKAP5_HUMAN] | 1.115 |
| P61313 | *RPL15* | 60S ribosomal protein L15 OS=Homo sapiens GN=RPL15 PE=1 SV=2 - [RL15_HUMAN] | 1.090 |
| Q12846 | *STX4* | Syntaxin-4 OS=Homo sapiens GN=STX4 PE=1 SV=2 - [STX4_HUMAN] | 0.862 |
| Q9ULC4 | *MCTS1* | Isoform 2 of Malignant T cell-amplified sequence 1 OS=Homo sapiens GN=MCTS1 - [MCTS1_HUMAN] | 1.099 |
| P19021 | *PAM* | Isoform 6 of Peptidyl-glycine alpha-amidating monooxygenase OS=Homo sapiens GN=PAM - [AMD_HUMAN] | 0.684 |
| Q9BZE9 | *ASPSCR1* | Isoform 4 of Tether containing UBX domain for GLUT4 OS=Homo sapiens GN=ASPSCR1 - [ASPC1_HUMAN] | 0.891 |
| Q13432 | *UNC119* | Isoform B of Protein unc-119 homolog A OS=Homo sapiens GN=UNC119 - [U119A_HUMAN] | 1.411 |
| O94888 | *UBXN7* | UBX domain-containing protein 7 OS=Homo sapiens GN=UBXN7 PE=1 SV=2 - [UBXN7_HUMAN] | 0.830 |
| P43155 | *CRAT* | Isoform 2 of Carnitine O-acetyltransferase OS=Homo sapiens GN=CRAT - [CACP_HUMAN] | 0.793 |
| Q9NSE4 | *IARS2* | Isoleucine--tRNA ligase, mitochondrial OS=Homo sapiens GN=IARS2 PE=1 SV=2 - [SYIM_HUMAN] | 0.877 |
| P50991 | *CCT4* | T-complex protein 1 subunit delta OS=Homo sapiens GN=CCT4 PE=1 SV=4 - [TCPD_HUMAN] | 1.141 |
| O75843 | *AP1G2* | AP-1 complex subunit gamma-like 2 OS=Homo sapiens GN=AP1G2 PE=1 SV=1 - [AP1G2_HUMAN] | 1.458 |
| Q92796 | *DLG3* | Isoform 2 of Disks large homolog 3 OS=Homo sapiens GN=DLG3 - [DLG3_HUMAN] | 1.171 |
| P01031 | *C5* | Complement C5 OS=Homo sapiens GN=C5 PE=1 SV=4 - [CO5_HUMAN] | 0.707 |
| Q9NVR0 | *KLHL11* | Kelch-like protein 11 OS=Homo sapiens GN=KLHL11 PE=1 SV=1 - [KLH11_HUMAN] | 1.236 |
| Q15063 | *POSTN* | Periostin OS=Homo sapiens GN=POSTN PE=1 SV=2 - [POSTN_HUMAN] | 3.638 |
| Q13263 | *TRIM28* | Transcription intermediary factor 1-beta OS=Homo sapiens GN=TRIM28 PE=1 SV=5 - [TIF1B_HUMAN] | 1.245 |
| P05023 | *ATP1A1* | Sodium/potassium-transporting ATPase subunit alpha-1 OS=Homo sapiens GN=ATP1A1 PE=1 SV=1 - [AT1A1_HUMAN] | 0.749 |
| P39656 | *DDOST* | Dolichyl-diphosphooligosaccharide--protein glycosyltransferase 48 kDa subunit OS=Homo sapiens GN=DDOST PE=1 SV=4 - [OST48_HUMAN] | 0.886 |
| O95159 | *ZFPL1* | Zinc finger protein-like 1 OS=Homo sapiens GN=ZFPL1 PE=1 SV=2 - [ZFPL1_HUMAN] | 1.296 |
| P01765 | *#N/A* | Ig heavy chain V-III region TIL OS=Homo sapiens PE=1 SV=1 - [HV304_HUMAN] | 0.574 |
| Q9Y4J8 | *DTNA* | Isoform 8 of Dystrobrevin alpha OS=Homo sapiens GN=DTNA - [DTNA_HUMAN] | 0.767 |
| P07358 | *C8B* | Complement component C8 beta chain OS=Homo sapiens GN=C8B PE=1 SV=3 - [CO8B_HUMAN] | 0.640 |
| P07477 | *PRSS1* | Trypsin-1 OS=Homo sapiens GN=PRSS1 PE=1 SV=1 - [TRY1_HUMAN] | 0.678 |
| O95373 | *IPO7* | Importin-7 OS=Homo sapiens GN=IPO7 PE=1 SV=1 - [IPO7_HUMAN] | 1.149 |
| Q9NZJ7 | *MTCH1* | Isoform 2 of Mitochondrial carrier homolog 1 OS=Homo sapiens GN=MTCH1 - [MTCH1_HUMAN] | 0.818 |
| Q15029 | *EFTUD2* | 116 kDa U5 small nuclear ribonucleoprotein component OS=Homo sapiens GN=EFTUD2 PE=1 SV=1 - [U5S1_HUMAN] | 1.102 |
| Q66LE6 | *PPP2R2D* | Serine/threonine-protein phosphatase 2A 55 kDa regulatory subunit B delta isoform OS=Homo sapiens GN=PPP2R2D PE=1 SV=1 - [2ABD_HUMAN] | 1.185 |
| P25440 | *BRD2* | Bromodomain-containing protein 2 OS=Homo sapiens GN=BRD2 PE=1 SV=2 - [BRD2_HUMAN] | 1.207 |
| O00267 | *SUPT5H* | Isoform 2 of Transcription elongation factor SPT5 OS=Homo sapiens GN=SUPT5H - [SPT5H_HUMAN] | 1.086 |
| P43378 | *PTPN9* | Tyrosine-protein phosphatase non-receptor type 9 OS=Homo sapiens GN=PTPN9 PE=1 SV=1 - [PTN9_HUMAN] | 0.819 |
| Q06033 | *ITIH3* | Isoform 2 of Inter-alpha-trypsin inhibitor heavy chain H3 OS=Homo sapiens GN=ITIH3 - [ITIH3_HUMAN] | 3.072 |
| P02461 | *COL3A1* | Collagen alpha-1(III) chain OS=Homo sapiens GN=COL3A1 PE=1 SV=4 - [CO3A1_HUMAN] | 1.597 |
| Q9Y2Z4 | *YARS2* | Tyrosine--tRNA ligase, mitochondrial OS=Homo sapiens GN=YARS2 PE=1 SV=2 - [SYYM_HUMAN] | 0.866 |
| Q9Y4K0 | *LOXL2* | Lysyl oxidase homolog 2 OS=Homo sapiens GN=LOXL2 PE=1 SV=1 - [LOXL2_HUMAN] | 1.415 |
| P06400 | *RB1* | Retinoblastoma-associated protein OS=Homo sapiens GN=RB1 PE=1 SV=2 - [RB_HUMAN] | 1.247 |
| P06756 | *ITGAV* | Isoform 2 of Integrin alpha-V OS=Homo sapiens GN=ITGAV - [ITAV_HUMAN] | 0.859 |
| Q13283 | *G3BP1* | Ras GTPase-activating protein-binding protein 1 OS=Homo sapiens GN=G3BP1 PE=1 SV=1 - [G3BP1_HUMAN] | 1.126 |
| P11498 | *PC* | Pyruvate carboxylase, mitochondrial OS=Homo sapiens GN=PC PE=1 SV=2 - [PYC_HUMAN] | 0.654 |
| Q13488 | *TCIRG1* | Isoform Short of V-type proton ATPase 116 kDa subunit a isoform 3 OS=Homo sapiens GN=TCIRG1 - [VPP3_HUMAN] | 0.814 |
| Q07812 | *BAX* | Isoform Epsilon of Apoptosis regulator BAX OS=Homo sapiens GN=BAX - [BAX_HUMAN] | 1.189 |
| Q16629 | *SRSF7* | Isoform 3 of Serine/arginine-rich splicing factor 7 OS=Homo sapiens GN=SRSF7 - [SRSF7_HUMAN] | 1.123 |
| Q8N163 | *CCAR2* | Protein KIAA1967 OS=Homo sapiens GN=KIAA1967 PE=1 SV=2 - [K1967_HUMAN] | 1.177 |
| Q15036 | *SNX17* | Sorting nexin-17 OS=Homo sapiens GN=SNX17 PE=1 SV=1 - [SNX17_HUMAN] | 0.887 |
| Q9BZI7 | *UPF3B* | Isoform 2 of Regulator of nonsense transcripts 3B OS=Homo sapiens GN=UPF3B - [REN3B_HUMAN] | 0.780 |
| Q13557 | *CAMK2D* | Isoform Delta 12 of Calcium/calmodulin-dependent protein kinase type II subunit delta OS=Homo sapiens GN=CAMK2D - [KCC2D_HUMAN] | 1.223 |
| P19021 | *PAM* | Isoform 2 of Peptidyl-glycine alpha-amidating monooxygenase OS=Homo sapiens GN=PAM - [AMD_HUMAN] | 0.668 |
| O15551 | *CLDN3* | Claudin-3 OS=Homo sapiens GN=CLDN3 PE=1 SV=1 - [CLD3_HUMAN] | 0.630 |
| Q5PRF9 | *SAMD4B* | Protein Smaug homolog 2 OS=Homo sapiens GN=SAMD4B PE=1 SV=1 - [SMAG2_HUMAN] | 1.160 |
| Q9UNZ2 | *NSFL1C* | NSFL1 cofactor p47 OS=Homo sapiens GN=NSFL1C PE=1 SV=2 - [NSF1C_HUMAN] | 0.888 |
| Q7L014 | *DDX46* | Probable ATP-dependent RNA helicase DDX46 OS=Homo sapiens GN=DDX46 PE=1 SV=2 - [DDX46_HUMAN] | 0.911 |
| Q9Y646 | *CPQ* | Plasma glutamate carboxypeptidase OS=Homo sapiens GN=PGCP PE=1 SV=1 - [PGCP_HUMAN] | 0.626 |
| O60506 | *SYNCRIP* | Isoform 2 of Heterogeneous nuclear ribonucleoprotein Q OS=Homo sapiens GN=SYNCRIP - [HNRPQ_HUMAN] | 1.089 |
| Q9H6W3 | *C14orf169* | Isoform 2 of Lysine-specific demethylase NO66 OS=Homo sapiens GN=NO66 - [NO66_HUMAN] | 1.264 |
| Q9H3U1 | *UNC45A* | Isoform 2 of Protein unc-45 homolog A OS=Homo sapiens GN=UNC45A - [UN45A_HUMAN] | 1.146 |
| Q7Z794 | *KRT77* | Keratin, type II cytoskeletal 1b OS=Homo sapiens GN=KRT77 PE=1 SV=3 - [K2C1B_HUMAN] | 0.378 |
| Q16363 | *LAMA4* | Isoform 2 of Laminin subunit alpha-4 OS=Homo sapiens GN=LAMA4 - [LAMA4_HUMAN] | 0.705 |
| P48506 | *GCLC* | Glutamate--cysteine ligase catalytic subunit OS=Homo sapiens GN=GCLC PE=1 SV=2 - [GSH1_HUMAN] | 1.541 |
| Q9NVP1 | *DDX18* | ATP-dependent RNA helicase DDX18 OS=Homo sapiens GN=DDX18 PE=1 SV=2 - [DDX18_HUMAN] | 1.192 |
| O96000 | *NDUFB10* | NADH dehydrogenase [ubiquinone] 1 beta subcomplex subunit 10 OS=Homo sapiens GN=NDUFB10 PE=1 SV=3 - [NDUBA_HUMAN] | 0.884 |
| Q9H488 | *POFUT1* | Isoform 2 of GDP-fucose protein O-fucosyltransferase 1 OS=Homo sapiens GN=POFUT1 - [OFUT1_HUMAN] | 1.179 |
| O00423 | *EML1* | Echinoderm microtubule-associated protein-like 1 OS=Homo sapiens GN=EML1 PE=1 SV=3 - [EMAL1_HUMAN] | 0.778 |
| Q9NX55 | *HYPK* | Huntingtin-interacting protein K OS=Homo sapiens GN=HYPK PE=1 SV=2 - [HYPK_HUMAN] | 0.857 |
| Q04941 | *PLP2* | Proteolipid protein 2 OS=Homo sapiens GN=PLP2 PE=1 SV=1 - [PLP2_HUMAN] | 0.762 |
| Q5SNT2 | *TMEM201* | Isoform SAMP1 of Transmembrane protein 201 OS=Homo sapiens GN=TMEM201 - [TM201_HUMAN] | 1.374 |
| P14314 | *PRKCSH* | Glucosidase 2 subunit beta OS=Homo sapiens GN=PRKCSH PE=1 SV=2 - [GLU2B_HUMAN] | 1.452 |
| Q13643 | *FHL3* | Four and a half LIM domains protein 3 OS=Homo sapiens GN=FHL3 PE=1 SV=4 - [FHL3_HUMAN] | 1.571 |
| Q02252 | *ALDH6A1* | Methylmalonate-semialdehyde dehydrogenase [acylating], mitochondrial OS=Homo sapiens GN=ALDH6A1 PE=1 SV=2 - [MMSA_HUMAN] | 0.739 |
| P01266 | *TG* | Thyroglobulin OS=Homo sapiens GN=TG PE=1 SV=5 - [THYG_HUMAN] | 0.129 |
| P39900 | *MMP12* | Macrophage metalloelastase OS=Homo sapiens GN=MMP12 PE=1 SV=1 - [MMP12_HUMAN] | 0.129 |
| Q7L9L4 | *MOB1B* | MOB kinase activator 1B OS=Homo sapiens GN=MOB1B PE=1 SV=3 - [MOB1B_HUMAN] | 1.230 |
| Q9NUQ9 | *FAM49B* | Protein FAM49B OS=Homo sapiens GN=FAM49B PE=1 SV=1 - [FA49B_HUMAN] | 1.196 |
| Q96D15 | *RCN3* | Reticulocalbin-3 OS=Homo sapiens GN=RCN3 PE=1 SV=1 - [RCN3_HUMAN] | 1.885 |
| P26006 | *ITGA3* | Integrin alpha-3 OS=Homo sapiens GN=ITGA3 PE=1 SV=5 - [ITA3_HUMAN] | 0.693 |
| P0C7X2 | *ZNF688* | Zinc finger protein 688 OS=Homo sapiens GN=ZNF688 PE=2 SV=1 - [ZN688_HUMAN] | 0.569 |
| Q14112 | *NID2* | Nidogen-2 OS=Homo sapiens GN=NID2 PE=1 SV=3 - [NID2_HUMAN] | 0.479 |
| P55809 | *OXCT1* | Succinyl-CoA:3-ketoacid-coenzyme A transferase 1, mitochondrial OS=Homo sapiens GN=OXCT1 PE=1 SV=1 - [SCOT1_HUMAN] | 0.754 |
| Q9H4A3 | *WNK1* | Isoform 2 of Serine/threonine-protein kinase WNK1 OS=Homo sapiens GN=WNK1 - [WNK1_HUMAN] | 1.123 |
| Q9Y2Q9 | *MRPS28* | 28S ribosomal protein S28, mitochondrial OS=Homo sapiens GN=MRPS28 PE=1 SV=1 - [RT28_HUMAN] | 0.835 |
| P05556 | *ITGB1* | Isoform Beta-1B of Integrin beta-1 OS=Homo sapiens GN=ITGB1 - [ITB1_HUMAN] | 0.883 |
| Q04323 | *UBXN1* | UBX domain-containing protein 1 OS=Homo sapiens GN=UBXN1 PE=1 SV=2 - [UBXN1_HUMAN] | 1.171 |
| Q63HN8 | *RNF213* | RING finger protein 213 OS=Homo sapiens GN=RNF213 PE=1 SV=2 - [RN213_HUMAN] | 1.356 |
| Q8IXQ6 | *PARP9* | Isoform 2 of Poly [ADP-ribose] polymerase 9 OS=Homo sapiens GN=PARP9 - [PARP9_HUMAN] | 0.765 |
| P16401 | *HIST1H1B* | Histone H1.5 OS=Homo sapiens GN=HIST1H1B PE=1 SV=3 - [H15_HUMAN] | 2.019 |
| Q8TAT6 | *NPLOC4* | Nuclear protein localization protein 4 homolog OS=Homo sapiens GN=NPLOC4 PE=1 SV=3 - [NPL4_HUMAN] | 1.149 |
| Q9Y5M8 | *SRPRB* | Signal recognition particle receptor subunit beta OS=Homo sapiens GN=SRPRB PE=1 SV=3 - [SRPRB_HUMAN] | 0.871 |
| Q9BX68 | *HINT2* | Histidine triad nucleotide-binding protein 2, mitochondrial OS=Homo sapiens GN=HINT2 PE=1 SV=1 - [HINT2_HUMAN] | 0.827 |
| Q15742 | *NAB2* | Isoform 2 of NGFI-A-binding protein 2 OS=Homo sapiens GN=NAB2 - [NAB2_HUMAN] | 0.698 |
| Q13546 | *RIPK1* | Isoform 2 of Receptor-interacting serine/threonine-protein kinase 1 OS=Homo sapiens GN=RIPK1 - [RIPK1_HUMAN] | 1.158 |
| Q08431 | *MFGE8* | Isoform 3 of Lactadherin OS=Homo sapiens GN=MFGE8 - [MFGM_HUMAN] | 0.645 |
| Q16698 | *DECR1* | 2,4-dienoyl-CoA reductase, mitochondrial OS=Homo sapiens GN=DECR1 PE=1 SV=1 - [DECR_HUMAN] | 0.810 |
| Q4V328 | *GRIPAP1* | Isoform 4 of GRIP1-associated protein 1 OS=Homo sapiens GN=GRIPAP1 - [GRAP1_HUMAN] | 1.138 |
| Q8ND30 | *PPFIBP2* | Liprin-beta-2 OS=Homo sapiens GN=PPFIBP2 PE=1 SV=3 - [LIPB2_HUMAN] | 1.401 |
| O00445 | *SYT5* | Synaptotagmin-5 OS=Homo sapiens GN=SYT5 PE=2 SV=2 - [SYT5_HUMAN] | 0.578 |
| P55786 | *NPEPPS* | Puromycin-sensitive aminopeptidase OS=Homo sapiens GN=NPEPPS PE=1 SV=2 - [PSA_HUMAN] | 1.187 |
| O95260 | *ATE1* | Arginyl-tRNA--protein transferase 1 OS=Homo sapiens GN=ATE1 PE=1 SV=2 - [ATE1_HUMAN] | 0.800 |
| Q9Y6Q5 | *AP1M2* | AP-1 complex subunit mu-2 OS=Homo sapiens GN=AP1M2 PE=1 SV=4 - [AP1M2_HUMAN] | 1.180 |
| P38117 | *ETFB* | Electron transfer flavoprotein subunit beta OS=Homo sapiens GN=ETFB PE=1 SV=3 - [ETFB_HUMAN] | 0.861 |
| P35579 | *MYH9* | Myosin-9 OS=Homo sapiens GN=MYH9 PE=1 SV=4 - [MYH9_HUMAN] | 1.242 |
| Q9BQA9 | *C17orf62* | Uncharacterized protein C17orf62 OS=Homo sapiens GN=C17orf62 PE=1 SV=1 - [CQ062_HUMAN] | 1.397 |
| P04196 | *HRG* | Histidine-rich glycoprotein OS=Homo sapiens GN=HRG PE=1 SV=1 - [HRG_HUMAN] | 0.742 |
| Q4ZHG4 | *FNDC1* | Isoform 2 of Fibronectin type III domain-containing protein 1 OS=Homo sapiens GN=FNDC1 - [FNDC1_HUMAN] | 2.118 |
| Q8TBC5 | *ZSCAN18* | Zinc finger and SCAN domain-containing protein 18 OS=Homo sapiens GN=ZSCAN18 PE=2 SV=2 - [ZSC18_HUMAN] | 0.774 |
| P52926 | *HMGA2* | High mobility group protein HMGI-C OS=Homo sapiens GN=HMGA2 PE=1 SV=1 - [HMGA2_HUMAN] | 1.572 |
| Q92841 | *DDX17* | Probable ATP-dependent RNA helicase DDX17 OS=Homo sapiens GN=DDX17 PE=1 SV=1 - [DDX17_HUMAN] | 1.109 |
| P51003 | *PAPOLA* | Poly(A) polymerase alpha OS=Homo sapiens GN=PAPOLA PE=1 SV=4 - [PAPOA_HUMAN] | 1.117 |
| Q92696 | *RABGGTA* | Geranylgeranyl transferase type-2 subunit alpha OS=Homo sapiens GN=RABGGTA PE=1 SV=2 - [PGTA_HUMAN] | 0.832 |
| Q7L2J0 | *MEPCE* | 7SK snRNA methylphosphate capping enzyme OS=Homo sapiens GN=MEPCE PE=1 SV=1 - [MEPCE_HUMAN] | 1.089 |
| P01591 | *IGJ* | Immunoglobulin J chain OS=Homo sapiens GN=IGJ PE=1 SV=4 - [IGJ_HUMAN] | 0.654 |
| P62136 | *PPP1CA* | Serine/threonine-protein phosphatase PP1-alpha catalytic subunit OS=Homo sapiens GN=PPP1CA PE=1 SV=1 - [PP1A_HUMAN] | 1.154 |
| P56192 | *MARS* | Methionine--tRNA ligase, cytoplasmic OS=Homo sapiens GN=MARS PE=1 SV=2 - [SYMC_HUMAN] | 1.140 |
| O75915 | *ARL6IP5* | PRA1 family protein 3 OS=Homo sapiens GN=ARL6IP5 PE=1 SV=1 - [PRAF3_HUMAN] | 0.900 |
| P62424 | *RPL7A* | 60S ribosomal protein L7a OS=Homo sapiens GN=RPL7A PE=1 SV=2 - [RL7A_HUMAN] | 1.097 |
| Q96EH3 | *MALSU1* | Uncharacterized protein C7orf30 OS=Homo sapiens GN=C7orf30 PE=2 SV=1 - [CG030_HUMAN] | 0.829 |
| P07814 | *EPRS* | Bifunctional glutamate/proline--tRNA ligase OS=Homo sapiens GN=EPRS PE=1 SV=5 - [SYEP_HUMAN] | 1.080 |
| P04406 | *GAPDH* | Glyceraldehyde-3-phosphate dehydrogenase OS=Homo sapiens GN=GAPDH PE=1 SV=3 - [G3P_HUMAN] | 1.182 |
| O75051 | *PLXNA2* | Plexin-A2 OS=Homo sapiens GN=PLXNA2 PE=1 SV=4 - [PLXA2_HUMAN] | 0.671 |
| Q6ZTQ3 | *RASSF6* | Isoform 4 of Ras association domain-containing protein 6 OS=Homo sapiens GN=RASSF6 - [RASF6_HUMAN] | 1.543 |
| O75494 | *SRSF10* | Isoform 4 of Serine/arginine-rich splicing factor 10 OS=Homo sapiens GN=SRSF10 - [SRS10_HUMAN] | 1.149 |
| P07948 | *LYN* | Isoform 2 of Tyrosine-protein kinase Lyn OS=Homo sapiens GN=LYN - [LYN_HUMAN] | 0.706 |
| Q14202 | *ZMYM3* | Isoform 2 of Zinc finger MYM-type protein 3 OS=Homo sapiens GN=ZMYM3 - [ZMYM3_HUMAN] | 1.442 |
| Q8N8S7 | *ENAH* | Isoform 2 of Protein enabled homolog OS=Homo sapiens GN=ENAH - [ENAH_HUMAN] | 1.208 |
| P62081 | *RPS7* | 40S ribosomal protein S7 OS=Homo sapiens GN=RPS7 PE=1 SV=1 - [RS7_HUMAN] | 1.063 |
| Q9Y2S6 | *TMA7* | Coiled-coil domain-containing protein 72 OS=Homo sapiens GN=CCDC72 PE=1 SV=1 - [CCD72_HUMAN] | 0.786 |
| O75569 | *PRKRA* | Interferon-inducible double stranded RNA-dependent protein kinase activator A OS=Homo sapiens GN=PRKRA PE=1 SV=1 - [PRKRA_HUMAN] | 1.178 |
| P02652 | *APOA2* | Apolipoprotein A-II OS=Homo sapiens GN=APOA2 PE=1 SV=1 - [APOA2_HUMAN] | 0.690 |
| Q8NC56 | *LEMD2* | LEM domain-containing protein 2 OS=Homo sapiens GN=LEMD2 PE=1 SV=1 - [LEMD2_HUMAN] | 1.232 |
| P36776 | *LONP1* | Lon protease homolog, mitochondrial OS=Homo sapiens GN=LONP1 PE=1 SV=2 - [LONM_HUMAN] | 0.858 |
| Q9UPT6 | *MAPK8IP3* | C-Jun-amino-terminal kinase-interacting protein 3 OS=Homo sapiens GN=MAPK8IP3 PE=1 SV=3 - [JIP3_HUMAN] | 1.552 |
| Q96AQ6 | *PBXIP1* | Isoform 2 of Pre-B-cell leukemia transcription factor-interacting protein 1 OS=Homo sapiens GN=PBXIP1 - [PBIP1_HUMAN] | 0.658 |
| P30086 | *PEBP1* | Phosphatidylethanolamine-binding protein 1 OS=Homo sapiens GN=PEBP1 PE=1 SV=3 - [PEBP1_HUMAN] | 0.802 |
| Q9NUQ6 | *SPATS2L* | Isoform 2 of SPATS2-like protein OS=Homo sapiens GN=SPATS2L - [SPS2L_HUMAN] | 0.622 |
| Q9BUJ2 | *HNRNPUL1* | Isoform 2 of Heterogeneous nuclear ribonucleoprotein U-like protein 1 OS=Homo sapiens GN=HNRNPUL1 - [HNRL1_HUMAN] | 1.176 |
| P14209 | *CD99* | CD99 antigen OS=Homo sapiens GN=CD99 PE=1 SV=1 - [CD99_HUMAN] | 0.624 |
| Q9UI08 | *EVL* | Ena/VASP-like protein OS=Homo sapiens GN=EVL PE=1 SV=2 - [EVL_HUMAN] | 1.236 |
| Q9UL15 | *BAG5* | BAG family molecular chaperone regulator 5 OS=Homo sapiens GN=BAG5 PE=1 SV=1 - [BAG5_HUMAN] | 1.262 |
| Q9Y3C1 | *NOP16* | Nucleolar protein 16 OS=Homo sapiens GN=NOP16 PE=1 SV=2 - [NOP16_HUMAN] | 1.244 |
| P29120 | *PCSK1* | Neuroendocrine convertase 1 OS=Homo sapiens GN=PCSK1 PE=1 SV=2 - [NEC1_HUMAN] | 0.483 |
| P41227 | *NAA10* | N-alpha-acetyltransferase 10 OS=Homo sapiens GN=NAA10 PE=1 SV=1 - [NAA10_HUMAN] | 1.182 |
| Q9HBL7 | *PLGRKT* | Transmembrane protein C9orf46 OS=Homo sapiens GN=C9orf46 PE=2 SV=1 - [CI046_HUMAN] | 0.588 |
| Q9HCM2 | *PLXNA4* | Plexin-A4 OS=Homo sapiens GN=PLXNA4 PE=1 SV=4 - [PLXA4_HUMAN] | 0.768 |
| Q9BXJ9 | *NAA15* | N-alpha-acetyltransferase 15, NatA auxiliary subunit OS=Homo sapiens GN=NAA15 PE=1 SV=1 - [NAA15_HUMAN] | 1.218 |
| Q13618 | *CUL3* | Isoform 3 of Cullin-3 OS=Homo sapiens GN=CUL3 - [CUL3_HUMAN] | 1.224 |
| Q9GZZ9 | *UBA5* | Ubiquitin-like modifier-activating enzyme 5 OS=Homo sapiens GN=UBA5 PE=1 SV=1 - [UBA5_HUMAN] | 0.785 |
| P51153 | *RAB13* | Ras-related protein Rab-13 OS=Homo sapiens GN=RAB13 PE=1 SV=1 - [RAB13_HUMAN] | 0.860 |
| P20908 | *COL5A1* | Collagen alpha-1(V) chain OS=Homo sapiens GN=COL5A1 PE=1 SV=3 - [CO5A1_HUMAN] | 1.659 |
| P53611 | *RABGGTB* | Geranylgeranyl transferase type-2 subunit beta OS=Homo sapiens GN=RABGGTB PE=1 SV=2 - [PGTB2_HUMAN] | 0.826 |
| O95782 | *AP2A1* | Isoform B of AP-2 complex subunit alpha-1 OS=Homo sapiens GN=AP2A1 - [AP2A1_HUMAN] | 0.907 |
| Q8TF05 | *PPP4R1* | Isoform 2 of Serine/threonine-protein phosphatase 4 regulatory subunit 1 OS=Homo sapiens GN=PPP4R1 - [PP4R1_HUMAN] | 1.427 |
| P36542 | *ATP5C1* | Isoform Heart of ATP synthase subunit gamma, mitochondrial OS=Homo sapiens GN=ATP5C1 - [ATPG_HUMAN] | 0.843 |
| Q8IWV7 | *UBR1* | E3 ubiquitin-protein ligase UBR1 OS=Homo sapiens GN=UBR1 PE=1 SV=1 - [UBR1_HUMAN] | 1.101 |
| P62701 | *RPS4X* | 40S ribosomal protein S4, X isoform OS=Homo sapiens GN=RPS4X PE=1 SV=2 - [RS4X_HUMAN] | 1.088 |
| Q9BZL4 | *PPP1R12C* | Isoform 3 of Protein phosphatase 1 regulatory subunit 12C OS=Homo sapiens GN=PPP1R12C - [PP12C_HUMAN] | 0.820 |
| O43314 | *PPIP5K2* | Isoform 2 of Inositol hexakisphosphate and diphosphoinositol-pentakisphosphate kinase 2 OS=Homo sapiens GN=PPIP5K2 - [VIP2_HUMAN] | 1.268 |
| O94915 | *FRYL* | Protein furry homolog-like OS=Homo sapiens GN=FRYL PE=1 SV=2 - [FRYL_HUMAN] | 1.201 |
| Q92766 | *RREB1* | Isoform 4 of Ras-responsive element-binding protein 1 OS=Homo sapiens GN=RREB1 - [RREB1_HUMAN] | 1.162 |
| O00161 | *SNAP23* | Isoform SNAP-23b of Synaptosomal-associated protein 23 OS=Homo sapiens GN=SNAP23 - [SNP23_HUMAN] | 0.910 |
| P33176 | *KIF5B* | Kinesin-1 heavy chain OS=Homo sapiens GN=KIF5B PE=1 SV=1 - [KINH_HUMAN] | 1.172 |
| O43493 | *TGOLN2* | Isoform 4 of Trans-Golgi network integral membrane protein 2 OS=Homo sapiens GN=TGOLN2 - [TGON2_HUMAN] | 0.724 |
| P27105 | *STOM* | Erythrocyte band 7 integral membrane protein OS=Homo sapiens GN=STOM PE=1 SV=3 - [STOM_HUMAN] | 0.672 |
| P60174 | *TPI1* | Isoform 2 of Triosephosphate isomerase OS=Homo sapiens GN=TPI1 - [TPIS_HUMAN] | 0.252 |
| Q9BVT8 | *TMUB1* | Transmembrane and ubiquitin-like domain-containing protein 1 OS=Homo sapiens GN=TMUB1 PE=1 SV=1 - [TMUB1_HUMAN] | 1.252 |
| Q6PML9 | *SLC30A9* | Zinc transporter 9 OS=Homo sapiens GN=SLC30A9 PE=1 SV=1 - [ZNT9_HUMAN] | 0.871 |
| Q8NE01 | *CNNM3* | Isoform 2 of Metal transporter CNNM3 OS=Homo sapiens GN=CNNM3 - [CNNM3_HUMAN] | 0.764 |
| Q16643 | *DBN1* | Drebrin OS=Homo sapiens GN=DBN1 PE=1 SV=4 - [DREB_HUMAN] | 1.338 |
| Q969G5 | *PRKCDBP* | Protein kinase C delta-binding protein OS=Homo sapiens GN=PRKCDBP PE=1 SV=3 - [PRDBP_HUMAN] | 0.628 |
| P26196 | *DDX6* | Probable ATP-dependent RNA helicase DDX6 OS=Homo sapiens GN=DDX6 PE=1 SV=2 - [DDX6_HUMAN] | 1.186 |
| Q8WUX9 | *CHMP7* | Charged multivesicular body protein 7 OS=Homo sapiens GN=CHMP7 PE=1 SV=1 - [CHMP7_HUMAN] | 1.238 |
| Q9UPY8 | *MAPRE3* | Isoform 2 of Microtubule-associated protein RP/EB family member 3 OS=Homo sapiens GN=MAPRE3 - [MARE3_HUMAN] | 0.844 |
| Q9BQ16 | *SPOCK3* | Isoform 2 of Testican-3 OS=Homo sapiens GN=SPOCK3 - [TICN3_HUMAN] | 0.518 |
| Q96I99 | *SUCLG2* | Succinyl-CoA ligase [GDP-forming] subunit beta, mitochondrial OS=Homo sapiens GN=SUCLG2 PE=1 SV=2 - [SUCB2_HUMAN] | 0.737 |
| O95747 | *OXSR1* | Serine/threonine-protein kinase OSR1 OS=Homo sapiens GN=OXSR1 PE=1 SV=1 - [OXSR1_HUMAN] | 0.861 |
| Q92504 | *SLC39A7* | Zinc transporter SLC39A7 OS=Homo sapiens GN=SLC39A7 PE=1 SV=2 - [S39A7_HUMAN] | 1.174 |
| Q8NE01 | *CNNM3* | Metal transporter CNNM3 OS=Homo sapiens GN=CNNM3 PE=1 SV=1 - [CNNM3_HUMAN] | 0.728 |
| Q32P41 | *TRMT5* | tRNA (guanine(37)-N1)-methyltransferase OS=Homo sapiens GN=TRMT5 PE=1 SV=2 - [TRM5_HUMAN] | 1.218 |
| P78352 | *DLG4* | Disks large homolog 4 OS=Homo sapiens GN=DLG4 PE=1 SV=3 - [DLG4_HUMAN] | 0.770 |
| O43924 | *PDE6D* | Retinal rod rhodopsin-sensitive cGMP 3',5'-cyclic phosphodiesterase subunit delta OS=Homo sapiens GN=PDE6D PE=1 SV=1 - [PDE6D_HUMAN] | 1.146 |
| Q96GM5 | *SMARCD1* | Isoform 2 of SWI/SNF-related matrix-associated actin-dependent regulator of chromatin subfamily D member 1 OS=Homo sapiens GN=SMARCD1 - [SMRD1_HUMAN] | 1.299 |
| Q86SF2 | *GALNT7* | N-acetylgalactosaminyltransferase 7 OS=Homo sapiens GN=GALNT7 PE=1 SV=1 - [GALT7_HUMAN] | 1.535 |
| P52701 | *MSH6* | Isoform GTBP-alt of DNA mismatch repair protein Msh6 OS=Homo sapiens GN=MSH6 - [MSH6_HUMAN] | 1.195 |
| P10643 | *C7* | Complement component C7 OS=Homo sapiens GN=C7 PE=1 SV=2 - [CO7_HUMAN] | 0.703 |
| Q9NZT2 | *OGFR* | Isoform 2 of Opioid growth factor receptor OS=Homo sapiens GN=OGFR - [OGFR_HUMAN] | 1.137 |
| Q8N1G4 | *LRRC47* | Leucine-rich repeat-containing protein 47 OS=Homo sapiens GN=LRRC47 PE=1 SV=1 - [LRC47_HUMAN] | 1.196 |
| Q8IUH5 | *ZDHHC17* | Isoform 3 of Palmitoyltransferase ZDHHC17 OS=Homo sapiens GN=ZDHHC17 - [ZDH17_HUMAN] | 1.182 |
| Q9BUA3 | *C11orf84* | Uncharacterized protein C11orf84 OS=Homo sapiens GN=C11orf84 PE=1 SV=3 - [CK084_HUMAN] | 1.255 |
| O95340 | *PAPSS2* | Bifunctional 3'-phosphoadenosine 5'-phosphosulfate synthase 2 OS=Homo sapiens GN=PAPSS2 PE=1 SV=2 - [PAPS2_HUMAN] | 1.709 |
| Q9P0J1 | *PDP1* | [Pyruvate dehydrogenase [acetyl-transferring]]-phosphatase 1, mitochondrial OS=Homo sapiens GN=PDP1 PE=1 SV=3 - [PDP1_HUMAN] | 0.672 |
| P80404 | *ABAT* | 4-aminobutyrate aminotransferase, mitochondrial OS=Homo sapiens GN=ABAT PE=1 SV=3 - [GABT_HUMAN] | 0.721 |
| Q6QNY0 | *BLOC1S3* | Biogenesis of lysosome-related organelles complex 1 subunit 3 OS=Homo sapiens GN=BLOC1S3 PE=1 SV=1 - [BL1S3_HUMAN] | 1.387 |
| Q13586 | *STIM1* | Stromal interaction molecule 1 OS=Homo sapiens GN=STIM1 PE=1 SV=3 - [STIM1_HUMAN] | 1.457 |
| P20618 | *PSMB1* | Proteasome subunit beta type-1 OS=Homo sapiens GN=PSMB1 PE=1 SV=2 - [PSB1_HUMAN] | 1.104 |
| Q99613 | *EIF3C* | Eukaryotic translation initiation factor 3 subunit C OS=Homo sapiens GN=EIF3C PE=1 SV=1 - [EIF3C_HUMAN] | 0.869 |
| P12955 | *PEPD* | Xaa-Pro dipeptidase OS=Homo sapiens GN=PEPD PE=1 SV=3 - [PEPD_HUMAN] | 0.852 |
| P04003 | *C4BPA* | C4b-binding protein alpha chain OS=Homo sapiens GN=C4BPA PE=1 SV=2 - [C4BPA_HUMAN] | 0.529 |
| Q9H2M9 | *RAB3GAP2* | Rab3 GTPase-activating protein non-catalytic subunit OS=Homo sapiens GN=RAB3GAP2 PE=1 SV=1 - [RBGPR_HUMAN] | 1.282 |
| Q9UM54 | *MYO6* | Isoform 5 of Myosin-VI OS=Homo sapiens GN=MYO6 - [MYO6_HUMAN] | 1.535 |
| P07360 | *C8G* | Complement component C8 gamma chain OS=Homo sapiens GN=C8G PE=1 SV=3 - [CO8G_HUMAN] | 0.595 |
| Q96NY7 | *CLIC6* | Isoform A of Chloride intracellular channel protein 6 OS=Homo sapiens GN=CLIC6 - [CLIC6_HUMAN] | 0.600 |
| Q9NRG1 | *PRTFDC1* | Isoform 2 of Phosphoribosyltransferase domain-containing protein 1 OS=Homo sapiens GN=PRTFDC1 - [PRDC1_HUMAN] | 1.222 |
| Q9H7N4 | *SCAF1* | Splicing factor, arginine/serine-rich 19 OS=Homo sapiens GN=SCAF1 PE=1 SV=3 - [SFR19_HUMAN] | 1.077 |
| P33991 | *MCM4* | DNA replication licensing factor MCM4 OS=Homo sapiens GN=MCM4 PE=1 SV=5 - [MCM4_HUMAN] | 1.335 |
| P33992 | *MCM5* | DNA replication licensing factor MCM5 OS=Homo sapiens GN=MCM5 PE=1 SV=5 - [MCM5_HUMAN] | 1.314 |
| P05543 | *SERPINA7* | Thyroxine-binding globulin OS=Homo sapiens GN=SERPINA7 PE=1 SV=2 - [THBG_HUMAN] | 0.728 |
| Q8WYA6 | *CTNNBL1* | Isoform 3 of Beta-catenin-like protein 1 OS=Homo sapiens GN=CTNNBL1 - [CTBL1_HUMAN] | 1.252 |
| P15924 | *DSP* | Desmoplakin OS=Homo sapiens GN=DSP PE=1 SV=3 - [DESP_HUMAN] | 1.783 |
| P07237 | *P4HB* | Protein disulfide-isomerase OS=Homo sapiens GN=P4HB PE=1 SV=3 - [PDIA1_HUMAN] | 1.213 |
| P08559 | *PDHA1* | Pyruvate dehydrogenase E1 component subunit alpha, somatic form, mitochondrial OS=Homo sapiens GN=PDHA1 PE=1 SV=3 - [ODPA_HUMAN] | 0.887 |
| P07311 | *ACYP1* | Acylphosphatase-1 OS=Homo sapiens GN=ACYP1 PE=1 SV=2 - [ACYP1_HUMAN] | 1.397 |
| P83876 | *TXNL4A* | Thioredoxin-like protein 4A OS=Homo sapiens GN=TXNL4A PE=1 SV=1 - [TXN4A_HUMAN] | 0.912 |
| P62258 | *YWHAE* | 14-3-3 protein epsilon OS=Homo sapiens GN=YWHAE PE=1 SV=1 - [1433E_HUMAN] | 0.874 |
| Q9NVG8 | *TBC1D13* | Isoform 2 of TBC1 domain family member 13 OS=Homo sapiens GN=TBC1D13 - [TBC13_HUMAN] | 1.115 |
| Q08629 | *SPOCK1* | Testican-1 OS=Homo sapiens GN=SPOCK1 PE=1 SV=1 - [TICN1_HUMAN] | 0.611 |
| Q96C36 | *PYCR2* | Pyrroline-5-carboxylate reductase 2 OS=Homo sapiens GN=PYCR2 PE=1 SV=1 - [P5CR2_HUMAN] | 1.272 |
| Q9P0S2 | *COX16* | Cytochrome c oxidase assembly protein COX16 homolog, mitochondrial OS=Homo sapiens GN=COX16 PE=2 SV=1 - [COX16_HUMAN] | 0.774 |
| P50990 | *CCT8* | T-complex protein 1 subunit theta OS=Homo sapiens GN=CCT8 PE=1 SV=4 - [TCPQ_HUMAN] | 1.105 |
| Q96AG4 | *LRRC59* | Leucine-rich repeat-containing protein 59 OS=Homo sapiens GN=LRRC59 PE=1 SV=1 - [LRC59_HUMAN] | 1.138 |
| Q02952 | *AKAP12* | Isoform 3 of A-kinase anchor protein 12 OS=Homo sapiens GN=AKAP12 - [AKA12_HUMAN] | 0.637 |
| Q15661 | *TPSAB1* | Isoform 2 of Tryptase alpha/beta-1 OS=Homo sapiens GN=TPSAB1 - [TRYB1_HUMAN] | 0.390 |
| P01859 | *IGHG2* | Ig gamma-2 chain C region OS=Homo sapiens GN=IGHG2 PE=1 SV=2 - [IGHG2_HUMAN] | 0.501 |
| Q05086 | *UBE3A* | Isoform I of Ubiquitin-protein ligase E3A OS=Homo sapiens GN=UBE3A - [UBE3A_HUMAN] | 0.895 |
| Q96HS1 | *PGAM5* | Isoform 2 of Serine/threonine-protein phosphatase PGAM5, mitochondrial OS=Homo sapiens GN=PGAM5 - [PGAM5_HUMAN] | 1.291 |
| Q9Y4E8 | *USP15* | Isoform 2 of Ubiquitin carboxyl-terminal hydrolase 15 OS=Homo sapiens GN=USP15 - [UBP15_HUMAN] | 1.094 |
| O60825 | *PFKFB2* | Isoform 2 of 6-phosphofructo-2-kinase/fructose-2,6-biphosphatase 2 OS=Homo sapiens GN=PFKFB2 - [F262_HUMAN] | 1.488 |
| Q15262 | *PTPRK* | Receptor-type tyrosine-protein phosphatase kappa OS=Homo sapiens GN=PTPRK PE=1 SV=2 - [PTPRK_HUMAN] | 0.683 |
| Q9Y376 | *CAB39* | Calcium-binding protein 39 OS=Homo sapiens GN=CAB39 PE=1 SV=1 - [CAB39_HUMAN] | 0.876 |
| P28288 | *ABCD3* | Isoform 2 of ATP-binding cassette sub-family D member 3 OS=Homo sapiens GN=ABCD3 - [ABCD3_HUMAN] | 1.354 |
| Q59EK9 | *RUNDC3A* | Isoform 2 of RUN domain-containing protein 3A OS=Homo sapiens GN=RUNDC3A - [RUN3A_HUMAN] | 0.803 |
| P63220 | *RPS21* | 40S ribosomal protein S21 OS=Homo sapiens GN=RPS21 PE=1 SV=1 - [RS21_HUMAN] | 1.060 |
| Q14C86 | *GAPVD1* | Isoform 4 of GTPase-activating protein and VPS9 domain-containing protein 1 OS=Homo sapiens GN=GAPVD1 - [GAPD1_HUMAN] | 1.132 |
| P26572 | *MGAT1* | Alpha-1,3-mannosyl-glycoprotein 2-beta-N-acetylglucosaminyltransferase OS=Homo sapiens GN=MGAT1 PE=2 SV=2 - [MGAT1_HUMAN] | 0.467 |
| Q96FV2 | *SCRN2* | Secernin-2 OS=Homo sapiens GN=SCRN2 PE=2 SV=3 - [SCRN2_HUMAN] | 0.820 |
| O60831 | *PRAF2* | PRA1 family protein 2 OS=Homo sapiens GN=PRAF2 PE=1 SV=1 - [PRAF2_HUMAN] | 0.900 |
| Q5W111 | *SPRYD7* | Isoform 2 of SPRY domain-containing protein 7 OS=Homo sapiens GN=SPRYD7 - [SPRY7_HUMAN] | 0.797 |
| P04080 | *CSTB* | Cystatin-B OS=Homo sapiens GN=CSTB PE=1 SV=2 - [CYTB_HUMAN] | 0.818 |
| Q96GZ6 | *SLC41A3* | Isoform 6 of Solute carrier family 41 member 3 OS=Homo sapiens GN=SLC41A3 - [S41A3_HUMAN] | 0.845 |
| P07858 | *CTSB* | Cathepsin B OS=Homo sapiens GN=CTSB PE=1 SV=3 - [CATB_HUMAN] | 0.492 |
| P08238 | *HSP90AB1* | Heat shock protein HSP 90-beta OS=Homo sapiens GN=HSP90AB1 PE=1 SV=4 - [HS90B_HUMAN] | 1.213 |
| P27635 | *RPL10* | 60S ribosomal protein L10 OS=Homo sapiens GN=RPL10 PE=1 SV=4 - [RL10_HUMAN] | 1.191 |
| Q8NBJ7 | *SUMF2* | Isoform 2 of Sulfatase-modifying factor 2 OS=Homo sapiens GN=SUMF2 - [SUMF2_HUMAN] | 0.821 |
| Q9HCJ6 | *VAT1L* | Synaptic vesicle membrane protein VAT-1 homolog-like OS=Homo sapiens GN=VAT1L PE=1 SV=2 - [VAT1L_HUMAN] | 0.606 |
| Q9NVM6 | *DNAJC17* | DnaJ homolog subfamily C member 17 OS=Homo sapiens GN=DNAJC17 PE=1 SV=1 - [DJC17_HUMAN] | 1.149 |
| Q9NZM3 | *ITSN2* | Isoform 4 of Intersectin-2 OS=Homo sapiens GN=ITSN2 - [ITSN2_HUMAN] | 0.864 |
| O95714 | *HERC2* | E3 ubiquitin-protein ligase HERC2 OS=Homo sapiens GN=HERC2 PE=1 SV=2 - [HERC2_HUMAN] | 1.211 |
| P61086 | *UBE2K* | Ubiquitin-conjugating enzyme E2 K OS=Homo sapiens GN=UBE2K PE=1 SV=3 - [UBE2K_HUMAN] | 1.158 |
| P08069 | *IGF1R* | Insulin-like growth factor 1 receptor OS=Homo sapiens GN=IGF1R PE=1 SV=1 - [IGF1R_HUMAN] | 0.753 |
| O15264 | *MAPK13* | Mitogen-activated protein kinase 13 OS=Homo sapiens GN=MAPK13 PE=1 SV=1 - [MK13_HUMAN] | 1.259 |
| Q05329 | *GAD2* | Glutamate decarboxylase 2 OS=Homo sapiens GN=GAD2 PE=1 SV=1 - [DCE2_HUMAN] | 7.773 |
| Q13242 | *SRSF9* | Serine/arginine-rich splicing factor 9 OS=Homo sapiens GN=SRSF9 PE=1 SV=1 - [SRSF9_HUMAN] | 1.158 |
| P28370 | *SMARCA1* | Probable global transcription activator SNF2L1 OS=Homo sapiens GN=SMARCA1 PE=1 SV=2 - [SMCA1_HUMAN] | 1.132 |
| P83436 | *COG7* | Conserved oligomeric Golgi complex subunit 7 OS=Homo sapiens GN=COG7 PE=1 SV=1 - [COG7_HUMAN] | 1.145 |
| Q04446 | *GBE1* | 1,4-alpha-glucan-branching enzyme OS=Homo sapiens GN=GBE1 PE=1 SV=3 - [GLGB_HUMAN] | 0.816 |
| Q7Z2Z2 | *EFTUD1* | Isoform 2 of Elongation factor Tu GTP-binding domain-containing protein 1 OS=Homo sapiens GN=EFTUD1 - [ETUD1_HUMAN] | 1.283 |
| P17612 | *PRKACA* | Isoform 2 of cAMP-dependent protein kinase catalytic subunit alpha OS=Homo sapiens GN=PRKACA - [KAPCA_HUMAN] | 0.855 |
| P28482 | *MAPK1* | Mitogen-activated protein kinase 1 OS=Homo sapiens GN=MAPK1 PE=1 SV=3 - [MK01_HUMAN] | 1.230 |
| O94763 | *URI1* | Isoform 2 of Unconventional prefoldin RPB5 interactor OS=Homo sapiens GN=RMP - [RMP_HUMAN] | 0.861 |
| P26232 | *CTNNA2* | Isoform 3 of Catenin alpha-2 OS=Homo sapiens GN=CTNNA2 - [CTNA2_HUMAN] | 0.643 |
| Q8TD19 | *NEK9* | Serine/threonine-protein kinase Nek9 OS=Homo sapiens GN=NEK9 PE=1 SV=2 - [NEK9_HUMAN] | 0.901 |
| Q9HB40 | *SCPEP1* | Isoform 2 of Retinoid-inducible serine carboxypeptidase OS=Homo sapiens GN=SCPEP1 - [RISC_HUMAN] | 0.740 |
| Q8WXD5 | *GEMIN6* | Gem-associated protein 6 OS=Homo sapiens GN=GEMIN6 PE=1 SV=1 - [GEMI6_HUMAN] | 0.734 |
| P41236 | *PPP1R2* | Protein phosphatase inhibitor 2 OS=Homo sapiens GN=PPP1R2 PE=1 SV=2 - [IPP2_HUMAN] | 0.912 |
| Q9Y5K8 | *ATP6V1D* | V-type proton ATPase subunit D OS=Homo sapiens GN=ATP6V1D PE=1 SV=1 - [VATD_HUMAN] | 0.852 |
| P35908 | *KRT2* | Keratin, type II cytoskeletal 2 epidermal OS=Homo sapiens GN=KRT2 PE=1 SV=2 - [K22E_HUMAN] | 0.428 |
| P00738 | *HP* | Haptoglobin OS=Homo sapiens GN=HP PE=1 SV=1 - [HPT_HUMAN] | 0.485 |
| Q13888 | *GTF2H2* | General transcription factor IIH subunit 2 OS=Homo sapiens GN=GTF2H2 PE=1 SV=1 - [TF2H2_HUMAN] | 1.129 |
| Q96MG8 | *PCMTD1* | Protein-L-isoaspartate O-methyltransferase domain-containing protein 1 OS=Homo sapiens GN=PCMTD1 PE=2 SV=2 - [PCMD1_HUMAN] | 1.259 |
| Q8IY81 | *FTSJ3* | Putative rRNA methyltransferase 3 OS=Homo sapiens GN=FTSJ3 PE=1 SV=2 - [RRMJ3_HUMAN] | 1.222 |
| P55822 | *SH3BGR* | SH3 domain-binding glutamic acid-rich protein OS=Homo sapiens GN=SH3BGR PE=1 SV=3 - [SH3BG_HUMAN] | 0.344 |
| Q16666 | *IFI16* | Isoform 3 of Gamma-interferon-inducible protein 16 OS=Homo sapiens GN=IFI16 - [IF16_HUMAN] | 1.460 |
| Q96HD1 | *CRELD1* | Cysteine-rich with EGF-like domain protein 1 OS=Homo sapiens GN=CRELD1 PE=1 SV=3 - [CREL1_HUMAN] | 0.838 |
| P18858 | *LIG1* | DNA ligase 1 OS=Homo sapiens GN=LIG1 PE=1 SV=1 - [DNLI1_HUMAN] | 1.224 |
| P13804 | *ETFA* | Electron transfer flavoprotein subunit alpha, mitochondrial OS=Homo sapiens GN=ETFA PE=1 SV=1 - [ETFA_HUMAN] | 0.820 |
| O60220 | *TIMM8A* | Mitochondrial import inner membrane translocase subunit Tim8 A OS=Homo sapiens GN=TIMM8A PE=1 SV=1 - [TIM8A_HUMAN] | 0.853 |
| Q9NPF4 | *OSGEP* | Probable tRNA threonylcarbamoyladenosine biosynthesis protein OSGEP OS=Homo sapiens GN=OSGEP PE=1 SV=1 - [OSGEP_HUMAN] | 1.147 |
| P07203 | *GPX1* | Glutathione peroxidase 1 OS=Homo sapiens GN=GPX1 PE=1 SV=4 - [GPX1_HUMAN] | 0.878 |
| A0MZ66 | *KIAA1598* | Shootin-1 OS=Homo sapiens GN=KIAA1598 PE=1 SV=4 - [SHOT1_HUMAN] | 1.107 |
| Q9UKV3 | *ACIN1* | Isoform 4 of Apoptotic chromatin condensation inducer in the nucleus OS=Homo sapiens GN=ACIN1 - [ACINU_HUMAN] | 1.106 |
| P50502 | *ST13* | Hsc70-interacting protein OS=Homo sapiens GN=ST13 PE=1 SV=2 - [F10A1_HUMAN] | 1.235 |
| Q9H910 | *HN1L* | Isoform 2 of Hematological and neurological expressed 1-like protein OS=Homo sapiens GN=HN1L - [HN1L_HUMAN] | 1.210 |
| O60828 | *PQBP1* | Isoform 2 of Polyglutamine-binding protein 1 OS=Homo sapiens GN=PQBP1 - [PQBP1_HUMAN] | 1.110 |
| Q8WZA1 | *POMGNT1* | Protein O-linked-mannose beta-1,2-N-acetylglucosaminyltransferase 1 OS=Homo sapiens GN=POMGNT1 PE=1 SV=2 - [PMGT1_HUMAN] | 1.277 |
| Q12905 | *ILF2* | Interleukin enhancer-binding factor 2 OS=Homo sapiens GN=ILF2 PE=1 SV=2 - [ILF2_HUMAN] | 1.095 |
| Q9UG01 | *IFT172* | Isoform 2 of Intraflagellar transport protein 172 homolog OS=Homo sapiens GN=IFT172 - [IF172_HUMAN] | 0.883 |
| O75131 | *CPNE3* | Copine-3 OS=Homo sapiens GN=CPNE3 PE=1 SV=1 - [CPNE3_HUMAN] | 1.216 |
| O14735 | *CDIPT* | CDP-diacylglycerol--inositol 3-phosphatidyltransferase OS=Homo sapiens GN=CDIPT PE=1 SV=1 - [CDIPT_HUMAN] | 1.458 |
| Q6NZI2 | *PTRF* | Polymerase I and transcript release factor OS=Homo sapiens GN=PTRF PE=1 SV=1 - [PTRF_HUMAN] | 0.497 |
| Q14155 | *ARHGEF7* | Isoform 1 of Rho guanine nucleotide exchange factor 7 OS=Homo sapiens GN=ARHGEF7 - [ARHG7_HUMAN] | 1.110 |
| Q9BTX3 | *TMEM208* | Isoform 2 of Transmembrane protein 208 OS=Homo sapiens GN=TMEM208 - [TM208_HUMAN] | 1.178 |
| Q86V21 | *AACS* | Isoform 2 of Acetoacetyl-CoA synthetase OS=Homo sapiens GN=AACS - [AACS_HUMAN] | 1.483 |
| P55010 | *EIF5* | Eukaryotic translation initiation factor 5 OS=Homo sapiens GN=EIF5 PE=1 SV=2 - [IF5_HUMAN] | 0.889 |
| P19484 | *TFEB* | Isoform 2 of Transcription factor EB OS=Homo sapiens GN=TFEB - [TFEB_HUMAN] | 1.213 |
| Q9Y3B8 | *REXO2* | Isoform 2 of Oligoribonuclease, mitochondrial OS=Homo sapiens GN=REXO2 - [ORN_HUMAN] | 1.165 |
| Q86U28 | *ISCA2* | Iron-sulfur cluster assembly 2 homolog, mitochondrial OS=Homo sapiens GN=ISCA2 PE=1 SV=2 - [ISCA2_HUMAN] | 0.885 |
| P46939 | *UTRN* | Utrophin OS=Homo sapiens GN=UTRN PE=1 SV=2 - [UTRO_HUMAN] | 0.655 |
| P45973 | *CBX5* | Chromobox protein homolog 5 OS=Homo sapiens GN=CBX5 PE=1 SV=1 - [CBX5_HUMAN] | 1.202 |
| O95298 | *NDUFC2* | NADH dehydrogenase [ubiquinone] 1 subunit C2 OS=Homo sapiens GN=NDUFC2 PE=1 SV=1 - [NDUC2_HUMAN] | 0.892 |
| P22676 | *CALB2* | Calretinin OS=Homo sapiens GN=CALB2 PE=1 SV=2 - [CALB2_HUMAN] | 0.264 |
| P26368 | *U2AF2* | Isoform 2 of Splicing factor U2AF 65 kDa subunit OS=Homo sapiens GN=U2AF2 - [U2AF2_HUMAN] | 0.873 |
| O00566 | *MPHOSPH10* | U3 small nucleolar ribonucleoprotein protein MPP10 OS=Homo sapiens GN=MPHOSPH10 PE=1 SV=2 - [MPP10_HUMAN] | 0.912 |
| Q13825 | *AUH* | Isoform 2 of Methylglutaconyl-CoA hydratase, mitochondrial OS=Homo sapiens GN=AUH - [AUHM_HUMAN] | 0.850 |
| Q9H5N1 | *RABEP2* | Rab GTPase-binding effector protein 2 OS=Homo sapiens GN=RABEP2 PE=1 SV=2 - [RABE2_HUMAN] | 1.573 |
| O14929 | *HAT1* | Isoform B of Histone acetyltransferase type B catalytic subunit OS=Homo sapiens GN=HAT1 - [HAT1_HUMAN] | 1.139 |
| Q13363 | *CTBP1* | C-terminal-binding protein 1 OS=Homo sapiens GN=CTBP1 PE=1 SV=2 - [CTBP1_HUMAN] | 1.138 |
| Q53EL6 | *PDCD4* | Programmed cell death protein 4 OS=Homo sapiens GN=PDCD4 PE=1 SV=2 - [PDCD4_HUMAN] | 0.814 |
| P21757 | *MSR1* | Isoform III of Macrophage scavenger receptor types I and II OS=Homo sapiens GN=MSR1 - [MSRE_HUMAN] | 0.725 |
| Q9Y618 | *NCOR2* | Isoform 5 of Nuclear receptor corepressor 2 OS=Homo sapiens GN=NCOR2 - [NCOR2_HUMAN] | 0.822 |
| Q9UJZ1 | *STOML2* | Stomatin-like protein 2 OS=Homo sapiens GN=STOML2 PE=1 SV=1 - [STML2_HUMAN] | 0.912 |
| P51636 | *CAV2* | Isoform C of Caveolin-2 OS=Homo sapiens GN=CAV2 - [CAV2_HUMAN] | 0.517 |
| O60313 | *OPA1* | Dynamin-like 120 kDa protein, mitochondrial OS=Homo sapiens GN=OPA1 PE=1 SV=3 - [OPA1_HUMAN] | 0.866 |
| Q9BTV4 | *TMEM43* | Transmembrane protein 43 OS=Homo sapiens GN=TMEM43 PE=1 SV=1 - [TMM43_HUMAN] | 0.826 |
| Q10570 | *CPSF1* | Cleavage and polyadenylation specificity factor subunit 1 OS=Homo sapiens GN=CPSF1 PE=1 SV=2 - [CPSF1_HUMAN] | 1.119 |
| O00533 | *CHL1* | Neural cell adhesion molecule L1-like protein OS=Homo sapiens GN=CHL1 PE=1 SV=4 - [CHL1_HUMAN] | 1.915 |
| Q9C0C2 | *TNKS1BP1* | 182 kDa tankyrase-1-binding protein OS=Homo sapiens GN=TNKS1BP1 PE=1 SV=4 - [TB182_HUMAN] | 1.195 |
| O95793 | *STAU1* | Isoform Short of Double-stranded RNA-binding protein Staufen homolog 1 OS=Homo sapiens GN=STAU1 - [STAU1_HUMAN] | 1.109 |
| P13645 | *KRT10* | Keratin, type I cytoskeletal 10 OS=Homo sapiens GN=KRT10 PE=1 SV=6 - [K1C10_HUMAN] | 0.426 |
| P24666 | *ACP1* | Low molecular weight phosphotyrosine protein phosphatase OS=Homo sapiens GN=ACP1 PE=1 SV=3 - [PPAC_HUMAN] | 0.868 |
| Q712K3 | *UBE2R2* | Ubiquitin-conjugating enzyme E2 R2 OS=Homo sapiens GN=UBE2R2 PE=1 SV=1 - [UB2R2_HUMAN] | 1.126 |
| Q9H2G2 | *SLK* | Isoform 2 of STE20-like serine/threonine-protein kinase OS=Homo sapiens GN=SLK - [SLK_HUMAN] | 1.134 |
| Q4KMP7 | *TBC1D10B* | TBC1 domain family member 10B OS=Homo sapiens GN=TBC1D10B PE=1 SV=3 - [TB10B_HUMAN] | 1.153 |
| O00754 | *MAN2B1* | Lysosomal alpha-mannosidase OS=Homo sapiens GN=MAN2B1 PE=1 SV=3 - [MA2B1_HUMAN] | 1.379 |
| P20851 | *C4BPB* | Isoform 2 of C4b-binding protein beta chain OS=Homo sapiens GN=C4BPB - [C4BPB_HUMAN] | 0.586 |
| Q92733 | *PRCC* | Proline-rich protein PRCC OS=Homo sapiens GN=PRCC PE=1 SV=1 - [PRCC_HUMAN] | 1.170 |
| Q15847 | *ADIRF* | Adipose most abundant gene transcript 2 protein OS=Homo sapiens GN=APM2 PE=1 SV=1 - [APM2_HUMAN] | 0.540 |
| Q9NQX4 | *MYO5C* | Myosin-Vc OS=Homo sapiens GN=MYO5C PE=1 SV=2 - [MYO5C_HUMAN] | 0.731 |
| Q9P0I2 | *EMC3* | Isoform 2 of Transmembrane protein 111 OS=Homo sapiens GN=TMEM111 - [TM111_HUMAN] | 0.796 |
| P22314 | *UBA1* | Ubiquitin-like modifier-activating enzyme 1 OS=Homo sapiens GN=UBA1 PE=1 SV=3 - [UBA1_HUMAN] | 0.923 |
| P16284 | *#N/A* | Isoform Delta13 of Platelet endothelial cell adhesion molecule OS=Homo sapiens GN=PECAM1 - [PECA1_HUMAN] | 0.703 |
| Q9H477 | *RBKS* | Ribokinase OS=Homo sapiens GN=RBKS PE=1 SV=1 - [RBSK_HUMAN] | 0.805 |
| Q9NSK0 | *KLC4* | Kinesin light chain 4 OS=Homo sapiens GN=KLC4 PE=1 SV=3 - [KLC4_HUMAN] | 1.247 |
| Q01130 | *SRSF2* | Serine/arginine-rich splicing factor 2 OS=Homo sapiens GN=SRSF2 PE=1 SV=4 - [SRSF2_HUMAN] | 1.486 |
| O15460 | *P4HA2* | Isoform IIa of Prolyl 4-hydroxylase subunit alpha-2 OS=Homo sapiens GN=P4HA2 - [P4HA2_HUMAN] | 1.303 |
| P58335 | *ANTXR2* | Isoform 4 of Anthrax toxin receptor 2 OS=Homo sapiens GN=ANTXR2 - [ANTR2_HUMAN] | 0.755 |
| Q7L0Y3 | *TRMT10C* | Mitochondrial ribonuclease P protein 1 OS=Homo sapiens GN=RG9MTD1 PE=1 SV=2 - [MRRP1_HUMAN] | 0.852 |
| Q8N766 | *EMC1* | Isoform 4 of Uncharacterized protein KIAA0090 OS=Homo sapiens GN=KIAA0090 - [K0090_HUMAN] | 0.854 |
| Q13620 | *CUL4B* | Isoform 2 of Cullin-4B OS=Homo sapiens GN=CUL4B - [CUL4B_HUMAN] | 0.728 |
| P13747 | *HLA-E* | HLA class I histocompatibility antigen, alpha chain E OS=Homo sapiens GN=HLA-E PE=1 SV=3 - [HLAE_HUMAN] | 1.611 |
| P01024 | *C3* | Complement C3 OS=Homo sapiens GN=C3 PE=1 SV=2 - [CO3_HUMAN] | 0.740 |
| P48637 | *GSS* | Glutathione synthetase OS=Homo sapiens GN=GSS PE=1 SV=1 - [GSHB_HUMAN] | 1.214 |
| P08183 | *ABCB1* | Multidrug resistance protein 1 OS=Homo sapiens GN=ABCB1 PE=1 SV=3 - [MDR1_HUMAN] | 0.461 |
| Q99729 | *HNRNPAB* | Isoform 3 of Heterogeneous nuclear ribonucleoprotein A/B OS=Homo sapiens GN=HNRNPAB - [ROAA_HUMAN] | 1.167 |
| P17050 | *NAGA* | Alpha-N-acetylgalactosaminidase OS=Homo sapiens GN=NAGA PE=1 SV=2 - [NAGAB_HUMAN] | 1.291 |
| Q7Z2W4 | *ZC3HAV1* | Zinc finger CCCH-type antiviral protein 1 OS=Homo sapiens GN=ZC3HAV1 PE=1 SV=3 - [ZCCHV_HUMAN] | 0.930 |
| Q14376 | *GALE* | UDP-glucose 4-epimerase OS=Homo sapiens GN=GALE PE=1 SV=2 - [GALE_HUMAN] | 1.351 |
| O94826 | *TOMM70A* | Mitochondrial import receptor subunit TOM70 OS=Homo sapiens GN=TOMM70A PE=1 SV=1 - [TOM70_HUMAN] | 0.845 |
| P62072 | *TIMM10* | Mitochondrial import inner membrane translocase subunit Tim10 OS=Homo sapiens GN=TIMM10 PE=1 SV=1 - [TIM10_HUMAN] | 0.825 |
| Q8WUP2 | *FBLIM1* | Isoform 3 of Filamin-binding LIM protein 1 OS=Homo sapiens GN=FBLIM1 - [FBLI1_HUMAN] | 1.322 |
| Q92854 | *SEMA4D* | Isoform 2 of Semaphorin-4D OS=Homo sapiens GN=SEMA4D - [SEM4D_HUMAN] | 1.198 |
| Q9C0B5 | *ZDHHC5* | Isoform 2 of Palmitoyltransferase ZDHHC5 OS=Homo sapiens GN=ZDHHC5 - [ZDHC5_HUMAN] | 0.754 |
| Q5HYI7 | *MTX3* | Isoform 2 of Metaxin-3 OS=Homo sapiens GN=MTX3 - [MTX3_HUMAN] | 0.820 |
| O75155 | *CAND2* | Isoform 2 of Cullin-associated NEDD8-dissociated protein 2 OS=Homo sapiens GN=CAND2 - [CAND2_HUMAN] | 0.679 |
| Q13158 | *FADD* | Protein FADD OS=Homo sapiens GN=FADD PE=1 SV=1 - [FADD_HUMAN] | 0.826 |
| Q9NVH6 | *TMLHE* | Trimethyllysine dioxygenase, mitochondrial OS=Homo sapiens GN=TMLHE PE=1 SV=1 - [TMLH_HUMAN] | 0.820 |
| Q9BY42 | *RTFDC1* | UPF0549 protein C20orf43 OS=Homo sapiens GN=C20orf43 PE=1 SV=3 - [CT043_HUMAN] | 0.868 |
| P50479 | *PDLIM4* | PDZ and LIM domain protein 4 OS=Homo sapiens GN=PDLIM4 PE=1 SV=2 - [PDLI4_HUMAN] | 0.729 |
| O75934 | *BCAS2* | Pre-mRNA-splicing factor SPF27 OS=Homo sapiens GN=BCAS2 PE=1 SV=1 - [SPF27_HUMAN] | 1.172 |
| P60880 | *SNAP25* | Synaptosomal-associated protein 25 OS=Homo sapiens GN=SNAP25 PE=1 SV=1 - [SNP25_HUMAN] | 0.781 |
| Q13148 | *TARDBP* | TAR DNA-binding protein 43 OS=Homo sapiens GN=TARDBP PE=1 SV=1 - [TADBP_HUMAN] | 1.094 |
| O43674 | *NDUFB5* | NADH dehydrogenase [ubiquinone] 1 beta subcomplex subunit 5, mitochondrial OS=Homo sapiens GN=NDUFB5 PE=1 SV=1 - [NDUB5_HUMAN] | 0.887 |
| P02748 | *C9* | Complement component C9 OS=Homo sapiens GN=C9 PE=1 SV=2 - [CO9_HUMAN] | 0.462 |
| P57737 | *CORO7* | Isoform 2 of Coronin-7 OS=Homo sapiens GN=CORO7 - [CORO7_HUMAN] | 1.296 |
| Q7Z3C6 | *ATG9A* | Isoform 3 of Autophagy-related protein 9A OS=Homo sapiens GN=ATG9A - [ATG9A_HUMAN] | 0.837 |
| Q96DA2 | *RAB39B* | Ras-related protein Rab-39B OS=Homo sapiens GN=RAB39B PE=1 SV=1 - [RB39B_HUMAN] | 0.815 |
| P08572 | *COL4A2* | Collagen alpha-2(IV) chain OS=Homo sapiens GN=COL4A2 PE=1 SV=4 - [CO4A2_HUMAN] | 0.578 |
| A5D8V6 | *VPS37C* | Vacuolar protein sorting-associated protein 37C OS=Homo sapiens GN=VPS37C PE=1 SV=2 - [VP37C_HUMAN] | 1.271 |
| P22105 | *TNXB* | Tenascin-X OS=Homo sapiens GN=TNXB PE=1 SV=3 - [TENX_HUMAN] | 0.469 |
| O75170 | *PPP6R2* | Isoform 6 of Serine/threonine-protein phosphatase 6 regulatory subunit 2 OS=Homo sapiens GN=PPP6R2 - [PP6R2_HUMAN] | 1.250 |
| P01612 | *#N/A* | Ig kappa chain V-I region Mev OS=Homo sapiens PE=1 SV=1 - [KV120_HUMAN] | 0.707 |
| P08493 | *MGP* | Matrix Gla protein OS=Homo sapiens GN=MGP PE=1 SV=2 - [MGP_HUMAN] | 0.516 |
| P19827 | *ITIH1* | Inter-alpha-trypsin inhibitor heavy chain H1 OS=Homo sapiens GN=ITIH1 PE=1 SV=3 - [ITIH1_HUMAN] | 1.530 |
| Q9NRY4 | *ARHGAP35* | Isoform 2 of Rho GTPase-activating protein 35 OS=Homo sapiens GN=ARHGAP35 - [RHG35_HUMAN] | 1.118 |
| Q15170 | *TCEAL1* | Isoform 2 of Transcription elongation factor A protein-like 1 OS=Homo sapiens GN=TCEAL1 - [TCAL1_HUMAN] | 0.872 |
| Q9NY59 | *SMPD3* | Sphingomyelin phosphodiesterase 3 OS=Homo sapiens GN=SMPD3 PE=1 SV=1 - [NSMA2_HUMAN] | 1.394 |
| P54577 | *YARS* | Tyrosine--tRNA ligase, cytoplasmic OS=Homo sapiens GN=YARS PE=1 SV=4 - [SYYC_HUMAN] | 1.206 |
| Q9HD33 | *MRPL47* | Isoform 2 of 39S ribosomal protein L47, mitochondrial OS=Homo sapiens GN=MRPL47 - [RM47_HUMAN] | 0.861 |
| P49368 | *CCT3* | Isoform 2 of T-complex protein 1 subunit gamma OS=Homo sapiens GN=CCT3 - [TCPG_HUMAN] | 1.105 |
| P02743 | *APCS* | Serum amyloid P-component OS=Homo sapiens GN=APCS PE=1 SV=2 - [SAMP_HUMAN] | 0.489 |
| O75746 | *SLC25A12* | Calcium-binding mitochondrial carrier protein Aralar1 OS=Homo sapiens GN=SLC25A12 PE=1 SV=2 - [CMC1_HUMAN] | 0.867 |
| P00352 | *ALDH1A1* | Retinal dehydrogenase 1 OS=Homo sapiens GN=ALDH1A1 PE=1 SV=2 - [AL1A1_HUMAN] | 0.561 |
| P31431 | *SDC4* | Syndecan-4 OS=Homo sapiens GN=SDC4 PE=1 SV=2 - [SDC4_HUMAN] | 0.607 |
| Q01968 | *OCRL* | Isoform B of Inositol polyphosphate 5-phosphatase OCRL-1 OS=Homo sapiens GN=OCRL - [OCRL_HUMAN] | 0.894 |
| Q9UJ14 | *GGT7* | Isoform 3 of Gamma-glutamyltransferase 7 OS=Homo sapiens GN=GGT7 - [GGT7_HUMAN] | 1.467 |
| Q9ULS5 | *TMCC3* | Transmembrane and coiled-coil domains protein 3 OS=Homo sapiens GN=TMCC3 PE=2 SV=3 - [TMCC3_HUMAN] | 1.556 |
| C9JLW8 | *FAM195B* | Protein FAM195B OS=Homo sapiens GN=FAM195B PE=3 SV=1 - [F195B_HUMAN] | 1.184 |
| P12956 | *XRCC6* | X-ray repair cross-complementing protein 6 OS=Homo sapiens GN=XRCC6 PE=1 SV=2 - [XRCC6_HUMAN] | 1.116 |
| P22352 | *GPX3* | Glutathione peroxidase 3 OS=Homo sapiens GN=GPX3 PE=1 SV=2 - [GPX3_HUMAN] | 0.219 |
| O43237 | *DYNC1LI2* | Cytoplasmic dynein 1 light intermediate chain 2 OS=Homo sapiens GN=DYNC1LI2 PE=1 SV=1 - [DC1L2_HUMAN] | 1.160 |
| O14683 | *TP53I11* | Tumor protein p53-inducible protein 11 OS=Homo sapiens GN=TP53I11 PE=2 SV=2 - [P5I11_HUMAN] | 1.503 |
| P25787 | *PSMA2* | Proteasome subunit alpha type-2 OS=Homo sapiens GN=PSMA2 PE=1 SV=2 - [PSA2_HUMAN] | 1.109 |
| P22105 | *TNXB* | Isoform 3 of Tenascin-X OS=Homo sapiens GN=TNXB - [TENX_HUMAN] | 0.433 |
| O60282 | *KIF5C* | Kinesin heavy chain isoform 5C OS=Homo sapiens GN=KIF5C PE=1 SV=1 - [KIF5C_HUMAN] | 1.730 |
| Q8TAQ2 | *SMARCC2* | Isoform 2 of SWI/SNF complex subunit SMARCC2 OS=Homo sapiens GN=SMARCC2 - [SMRC2_HUMAN] | 1.181 |
| P33908 | *MAN1A1* | Mannosyl-oligosaccharide 1,2-alpha-mannosidase IA OS=Homo sapiens GN=MAN1A1 PE=1 SV=3 - [MA1A1_HUMAN] | 0.721 |
| P36954 | *POLR2I* | DNA-directed RNA polymerase II subunit RPB9 OS=Homo sapiens GN=POLR2I PE=1 SV=1 - [RPB9_HUMAN] | 0.785 |
| Q15738 | *NSDHL* | Sterol-4-alpha-carboxylate 3-dehydrogenase, decarboxylating OS=Homo sapiens GN=NSDHL PE=1 SV=2 - [NSDHL_HUMAN] | 1.216 |
| Q96DG6 | *CMBL* | Carboxymethylenebutenolidase homolog OS=Homo sapiens GN=CMBL PE=1 SV=1 - [CMBL_HUMAN] | 0.774 |
| P01620 | *#N/A* | Ig kappa chain V-III region SIE OS=Homo sapiens PE=1 SV=1 - [KV302_HUMAN] | 0.752 |
| Q16853 | *AOC3* | Membrane primary amine oxidase OS=Homo sapiens GN=AOC3 PE=1 SV=3 - [AOC3_HUMAN] | 0.383 |
| P13716 | *ALAD* | Delta-aminolevulinic acid dehydratase OS=Homo sapiens GN=ALAD PE=1 SV=1 - [HEM2_HUMAN] | 0.826 |
| Q8IYB5 | *SMAP1* | Isoform 3 of Stromal membrane-associated protein 1 OS=Homo sapiens GN=SMAP1 - [SMAP1_HUMAN] | 1.621 |
| Q76M96 | *CCDC80* | Coiled-coil domain-containing protein 80 OS=Homo sapiens GN=CCDC80 PE=1 SV=1 - [CCD80_HUMAN] | 2.075 |
| Q16740 | *CLPP* | Putative ATP-dependent Clp protease proteolytic subunit, mitochondrial OS=Homo sapiens GN=CLPP PE=1 SV=1 - [CLPP_HUMAN] | 0.845 |
| P30085 | *CMPK1* | UMP-CMP kinase OS=Homo sapiens GN=CMPK1 PE=1 SV=3 - [KCY_HUMAN] | 1.371 |
| Q7Z5G4 | *GOLGA7* | Golgin subfamily A member 7 OS=Homo sapiens GN=GOLGA7 PE=1 SV=2 - [GOGA7_HUMAN] | 0.872 |
| Q8N806 | *UBR7* | Putative E3 ubiquitin-protein ligase UBR7 OS=Homo sapiens GN=UBR7 PE=1 SV=2 - [UBR7_HUMAN] | 1.123 |
| Q8N3P4 | *VPS8* | Isoform 2 of Vacuolar protein sorting-associated protein 8 homolog OS=Homo sapiens GN=VPS8 - [VPS8_HUMAN] | 1.186 |
| P37802 | *TAGLN2* | Transgelin-2 OS=Homo sapiens GN=TAGLN2 PE=1 SV=3 - [TAGL2_HUMAN] | 1.174 |
| Q9P2L0 | *WDR35* | Isoform 2 of WD repeat-containing protein 35 OS=Homo sapiens GN=WDR35 - [WDR35_HUMAN] | 0.818 |
| Q6PJG2 | *ELMSAN1* | Uncharacterized protein C14orf43 OS=Homo sapiens GN=C14orf43 PE=1 SV=2 - [CN043_HUMAN] | 0.618 |
| O95837 | *GNA14* | Guanine nucleotide-binding protein subunit alpha-14 OS=Homo sapiens GN=GNA14 PE=2 SV=1 - [GNA14_HUMAN] | 0.654 |
| P16930 | *FAH* | Fumarylacetoacetase OS=Homo sapiens GN=FAH PE=1 SV=2 - [FAAA_HUMAN] | 0.720 |
| O95861 | *BPNT1* | 3'(2'),5'-bisphosphate nucleotidase 1 OS=Homo sapiens GN=BPNT1 PE=1 SV=1 - [BPNT1_HUMAN] | 1.226 |
| Q9P0J0 | *NDUFA13* | NADH dehydrogenase [ubiquinone] 1 alpha subcomplex subunit 13 OS=Homo sapiens GN=NDUFA13 PE=1 SV=3 - [NDUAD_HUMAN] | 0.903 |
| Q9NZD2 | *GLTP* | Glycolipid transfer protein OS=Homo sapiens GN=GLTP PE=1 SV=3 - [GLTP_HUMAN] | 1.350 |
| P51570 | *GALK1* | Galactokinase OS=Homo sapiens GN=GALK1 PE=1 SV=1 - [GALK1_HUMAN] | 1.215 |
| Q96T23 | *RSF1* | Isoform 2 of Remodeling and spacing factor 1 OS=Homo sapiens GN=RSF1 - [RSF1_HUMAN] | 1.146 |
| Q96GQ5 | *C16orf58* | UPF0420 protein C16orf58 OS=Homo sapiens GN=C16orf58 PE=1 SV=2 - [CP058_HUMAN] | 1.349 |
| Q9Y320 | *TMX2* | Isoform 2 of Thioredoxin-related transmembrane protein 2 OS=Homo sapiens GN=TMX2 - [TMX2_HUMAN] | 1.137 |
| Q9H1E5 | *TMX4* | Thioredoxin-related transmembrane protein 4 OS=Homo sapiens GN=TMX4 PE=1 SV=1 - [TMX4_HUMAN] | 0.820 |
| Q5GLZ8 | *HERC4* | Isoform 6 of Probable E3 ubiquitin-protein ligase HERC4 OS=Homo sapiens GN=HERC4 - [HERC4_HUMAN] | 1.114 |
| O94992 | *HEXIM1* | Protein HEXIM1 OS=Homo sapiens GN=HEXIM1 PE=1 SV=1 - [HEXI1_HUMAN] | 0.878 |
| P69891 | *HBG2* | Hemoglobin subunit gamma-1 OS=Homo sapiens GN=HBG1 PE=1 SV=2 - [HBG1_HUMAN] | 0.385 |
| Q9P2B4 | *CTTNBP2NL* | CTTNBP2 N-terminal-like protein OS=Homo sapiens GN=CTTNBP2NL PE=1 SV=2 - [CT2NL_HUMAN] | 1.159 |
| O95926 | *SYF2* | Pre-mRNA-splicing factor SYF2 OS=Homo sapiens GN=SYF2 PE=1 SV=1 - [SYF2_HUMAN] | 1.113 |
| Q96QZ7 | *MAGI1* | Isoform 7 of Membrane-associated guanylate kinase, WW and PDZ domain-containing protein 1 OS=Homo sapiens GN=MAGI1 - [MAGI1_HUMAN] | 0.909 |
| Q5JTW2 | *CEP78* | Centrosomal protein of 78 kDa OS=Homo sapiens GN=CEP78 PE=1 SV=1 - [CEP78_HUMAN] | 0.911 |
| P40939 | *HADHA* | Trifunctional enzyme subunit alpha, mitochondrial OS=Homo sapiens GN=HADHA PE=1 SV=2 - [ECHA_HUMAN] | 0.883 |
| P57772 | *EEFSEC* | Selenocysteine-specific elongation factor OS=Homo sapiens GN=EEFSEC PE=1 SV=4 - [SELB_HUMAN] | 0.869 |
| P36955 | *SERPINF1* | Pigment epithelium-derived factor OS=Homo sapiens GN=SERPINF1 PE=1 SV=4 - [PEDF_HUMAN] | 2.112 |
| O60739 | *EIF1B* | Eukaryotic translation initiation factor 1b OS=Homo sapiens GN=EIF1B PE=1 SV=2 - [EIF1B_HUMAN] | 0.871 |
| O15294 | *OGT* | Isoform 4 of UDP-N-acetylglucosamine--peptide N-acetylglucosaminyltransferase 110 kDa subunit OS=Homo sapiens GN=OGT - [OGT1_HUMAN] | 0.928 |
| Q9NQW7 | *XPNPEP1* | Xaa-Pro aminopeptidase 1 OS=Homo sapiens GN=XPNPEP1 PE=1 SV=3 - [XPP1_HUMAN] | 1.114 |
| P11234 | *RALB* | Ras-related protein Ral-B OS=Homo sapiens GN=RALB PE=1 SV=1 - [RALB_HUMAN] | 1.147 |
| Q9P2X3 | *IMPACT* | Isoform 2 of Protein IMPACT OS=Homo sapiens GN=IMPACT - [IMPCT_HUMAN] | 0.821 |
| Q7Z7F7 | *MRPL55* | 39S ribosomal protein L55, mitochondrial OS=Homo sapiens GN=MRPL55 PE=1 SV=1 - [RM55_HUMAN] | 0.847 |
| Q9P0U4 | *CXXC1* | CpG-binding protein OS=Homo sapiens GN=CXXC1 PE=1 SV=2 - [CXXC1_HUMAN] | 1.113 |
| P40227 | *CCT6A* | T-complex protein 1 subunit zeta OS=Homo sapiens GN=CCT6A PE=1 SV=3 - [TCPZ_HUMAN] | 1.103 |
| Q9UHD2 | *TBK1* | Serine/threonine-protein kinase TBK1 OS=Homo sapiens GN=TBK1 PE=1 SV=1 - [TBK1_HUMAN] | 0.911 |
| P21266 | *GSTM3* | Glutathione S-transferase Mu 3 OS=Homo sapiens GN=GSTM3 PE=1 SV=3 - [GSTM3_HUMAN] | 0.660 |
| Q8TB22 | *SPATA20* | Isoform 3 of Spermatogenesis-associated protein 20 OS=Homo sapiens GN=SPATA20 - [SPT20_HUMAN] | 0.774 |
| Q96P47 | *AGAP3* | Arf-GAP with GTPase, ANK repeat and PH domain-containing protein 3 OS=Homo sapiens GN=AGAP3 PE=1 SV=2 - [AGAP3_HUMAN] | 0.879 |
| Q9NVE5 | *USP40* | Ubiquitin carboxyl-terminal hydrolase 40 OS=Homo sapiens GN=USP40 PE=2 SV=3 - [UBP40_HUMAN] | 1.207 |
| P01871 | *#N/A* | Ig mu chain C region OS=Homo sapiens GN=IGHM PE=1 SV=3 - [IGHM_HUMAN] | 0.516 |
| P98171 | *ARHGAP4* | Rho GTPase-activating protein 4 OS=Homo sapiens GN=ARHGAP4 PE=1 SV=2 - [RHG04_HUMAN] | 1.368 |
| Q10713 | *PMPCA* | Mitochondrial-processing peptidase subunit alpha OS=Homo sapiens GN=PMPCA PE=1 SV=2 - [MPPA_HUMAN] | 1.138 |
| P35442 | *THBS2* | Thrombospondin-2 OS=Homo sapiens GN=THBS2 PE=1 SV=2 - [TSP2_HUMAN] | 3.738 |
| Q5T1J5 | *#N/A* | Putative coiled-coil-helix-coiled-coil-helix domain-containing protein CHCHD2P9, mitochondrial OS=Homo sapiens GN=CHCHD2P9 PE=5 SV=1 - [CHCH9_HUMAN] | 0.876 |
| Q16134 | *ETFDH* | Electron transfer flavoprotein-ubiquinone oxidoreductase, mitochondrial OS=Homo sapiens GN=ETFDH PE=1 SV=2 - [ETFD_HUMAN] | 0.853 |
| P02765 | *AHSG* | Alpha-2-HS-glycoprotein OS=Homo sapiens GN=AHSG PE=1 SV=1 - [FETUA_HUMAN] | 0.777 |
| Q16836 | *HADH* | Hydroxyacyl-coenzyme A dehydrogenase, mitochondrial OS=Homo sapiens GN=HADH PE=1 SV=3 - [HCDH_HUMAN] | 0.684 |
| Q6ZMP0 | *THSD4* | Isoform 2 of Thrombospondin type-1 domain-containing protein 4 OS=Homo sapiens GN=THSD4 - [THSD4_HUMAN] | 0.498 |
| Q07065 | *CKAP4* | Cytoskeleton-associated protein 4 OS=Homo sapiens GN=CKAP4 PE=1 SV=2 - [CKAP4_HUMAN] | 1.467 |
| O95562 | *SFT2D2* | Vesicle transport protein SFT2B OS=Homo sapiens GN=SFT2D2 PE=1 SV=1 - [SFT2B_HUMAN] | 0.891 |
| Q8TEQ8 | *PIGO* | Isoform 2 of GPI ethanolamine phosphate transferase 3 OS=Homo sapiens GN=PIGO - [PIGO_HUMAN] | 1.229 |
| P21281 | *ATP6V1B2* | V-type proton ATPase subunit B, brain isoform OS=Homo sapiens GN=ATP6V1B2 PE=1 SV=3 - [VATB2_HUMAN] | 0.903 |
| P11413 | *G6PD* | Glucose-6-phosphate 1-dehydrogenase OS=Homo sapiens GN=G6PD PE=1 SV=4 - [G6PD_HUMAN] | 0.808 |
| Q03135 | *CAV1* | Caveolin-1 OS=Homo sapiens GN=CAV1 PE=1 SV=4 - [CAV1_HUMAN] | 0.498 |
| Q8IZP2 | *#N/A* | Putative protein FAM10A4 OS=Homo sapiens GN=ST13P4 PE=5 SV=1 - [ST134_HUMAN] | 1.217 |
| Q9H3K2 | *GHITM* | Growth hormone-inducible transmembrane protein OS=Homo sapiens GN=GHITM PE=1 SV=2 - [GHITM_HUMAN] | 0.898 |
| Q9NR31 | *SAR1A* | GTP-binding protein SAR1a OS=Homo sapiens GN=SAR1A PE=1 SV=1 - [SAR1A_HUMAN] | 1.167 |
| P09669 | *COX6C* | Cytochrome c oxidase subunit 6C OS=Homo sapiens GN=COX6C PE=1 SV=2 - [COX6C_HUMAN] | 0.859 |
| Q9Y4C2 | *TCAF1* | Isoform 2 of Protein FAM115A OS=Homo sapiens GN=FAM115A - [F115A_HUMAN] | 1.353 |
| P61225 | *RAP2B* | Ras-related protein Rap-2b OS=Homo sapiens GN=RAP2B PE=1 SV=1 - [RAP2B_HUMAN] | 0.791 |
| Q96GA7 | *SDSL* | Serine dehydratase-like OS=Homo sapiens GN=SDSL PE=1 SV=1 - [SDSL_HUMAN] | 0.873 |
| Q96JQ2 | *CLMN* | Calmin OS=Homo sapiens GN=CLMN PE=1 SV=1 - [CLMN_HUMAN] | 1.569 |
| P11717 | *IGF2R* | Cation-independent mannose-6-phosphate receptor OS=Homo sapiens GN=IGF2R PE=1 SV=3 - [MPRI_HUMAN] | 1.280 |
| Q8N9R8 | *SCAI* | Protein SCAI OS=Homo sapiens GN=SCAI PE=1 SV=2 - [SCAI_HUMAN] | 1.238 |
| Q7Z3K3 | *POGZ* | Isoform 5 of Pogo transposable element with ZNF domain OS=Homo sapiens GN=POGZ - [POGZ_HUMAN] | 1.140 |
| O00499 | *BIN1* | Isoform BIN1-10-13 of Myc box-dependent-interacting protein 1 OS=Homo sapiens GN=BIN1 - [BIN1_HUMAN] | 0.744 |
| Q9HB71 | *CACYBP* | Calcyclin-binding protein OS=Homo sapiens GN=CACYBP PE=1 SV=2 - [CYBP_HUMAN] | 1.329 |
| P35237 | *SERPINB6* | Serpin B6 OS=Homo sapiens GN=SERPINB6 PE=1 SV=3 - [SPB6_HUMAN] | 1.132 |
| O43676 | *NDUFB3* | NADH dehydrogenase [ubiquinone] 1 beta subcomplex subunit 3 OS=Homo sapiens GN=NDUFB3 PE=1 SV=3 - [NDUB3_HUMAN] | 0.910 |
| Q15166 | *PON3* | Serum paraoxonase/lactonase 3 OS=Homo sapiens GN=PON3 PE=1 SV=3 - [PON3_HUMAN] | 1.396 |
| P67809 | *YBX1* | Nuclease-sensitive element-binding protein 1 OS=Homo sapiens GN=YBX1 PE=1 SV=3 - [YBOX1_HUMAN] | 1.169 |
| Q9H6V9 | *C2orf43* | UPF0554 protein C2orf43 OS=Homo sapiens GN=C2orf43 PE=1 SV=1 - [CB043_HUMAN] | 1.253 |
| Q8TDB6 | *DTX3L* | E3 ubiquitin-protein ligase DTX3L OS=Homo sapiens GN=DTX3L PE=1 SV=1 - [DTX3L_HUMAN] | 0.825 |
| Q9Y6M5 | *SLC30A1* | Zinc transporter 1 OS=Homo sapiens GN=SLC30A1 PE=1 SV=3 - [ZNT1_HUMAN] | 0.798 |
| Q9NPF5 | *DMAP1* | DNA methyltransferase 1-associated protein 1 OS=Homo sapiens GN=DMAP1 PE=1 SV=1 - [DMAP1_HUMAN] | 1.108 |
| P67936 | *TPM4* | Tropomyosin alpha-4 chain OS=Homo sapiens GN=TPM4 PE=1 SV=3 - [TPM4_HUMAN] | 1.280 |
| Q14676 | *MDC1* | Isoform 4 of Mediator of DNA damage checkpoint protein 1 OS=Homo sapiens GN=MDC1 - [MDC1_HUMAN] | 1.160 |
| P12883 | *MYH7* | Myosin-7 OS=Homo sapiens GN=MYH7 PE=1 SV=5 - [MYH7_HUMAN] | 0.154 |
| Q01469 | *FABP5* | Fatty acid-binding protein, epidermal OS=Homo sapiens GN=FABP5 PE=1 SV=3 - [FABP5_HUMAN] | 3.376 |
| Q9GZY8 | *MFF* | Isoform 4 of Mitochondrial fission factor OS=Homo sapiens GN=MFF - [MFF_HUMAN] | 1.081 |
| Q8IWU6 | *SULF1* | Extracellular sulfatase Sulf-1 OS=Homo sapiens GN=SULF1 PE=1 SV=1 - [SULF1_HUMAN] | 0.537 |
| P02647 | *APOA1* | Apolipoprotein A-I OS=Homo sapiens GN=APOA1 PE=1 SV=1 - [APOA1_HUMAN] | 0.708 |
| P28062 | *PSMB8* | Isoform 2 of Proteasome subunit beta type-8 OS=Homo sapiens GN=PSMB8 - [PSB8_HUMAN] | 1.738 |
| P12429 | *ANXA3* | Annexin A3 OS=Homo sapiens GN=ANXA3 PE=1 SV=3 - [ANXA3_HUMAN] | 0.480 |
| Q13547 | *HDAC1* | Histone deacetylase 1 OS=Homo sapiens GN=HDAC1 PE=1 SV=1 - [HDAC1_HUMAN] | 1.211 |
| Q96S55 | *WRNIP1* | Isoform 2 of ATPase WRNIP1 OS=Homo sapiens GN=WRNIP1 - [WRIP1_HUMAN] | 1.166 |
| Q8WUY1 | *THEM6* | UPF0670 protein C8orf55 OS=Homo sapiens GN=C8orf55 PE=1 SV=2 - [CH055_HUMAN] | 1.201 |
| P40424 | *PBX1* | Isoform PBX1b of Pre-B-cell leukemia transcription factor 1 OS=Homo sapiens GN=PBX1 - [PBX1_HUMAN] | 1.254 |
| P61204 | *ARF3* | ADP-ribosylation factor 3 OS=Homo sapiens GN=ARF3 PE=1 SV=2 - [ARF3_HUMAN] | 1.102 |
| Q92626 | *PXDN* | Isoform 2 of Peroxidasin homolog OS=Homo sapiens GN=PXDN - [PXDN_HUMAN] | 1.316 |
| Q15628 | *TRADD* | Tumor necrosis factor receptor type 1-associated DEATH domain protein OS=Homo sapiens GN=TRADD PE=1 SV=2 - [TRADD_HUMAN] | 1.262 |
| Q13617 | *CUL2* | Cullin-2 OS=Homo sapiens GN=CUL2 PE=1 SV=2 - [CUL2_HUMAN] | 1.162 |
| Q96GD0 | *PDXP* | Pyridoxal phosphate phosphatase OS=Homo sapiens GN=PDXP PE=1 SV=2 - [PLPP_HUMAN] | 1.171 |
| Q12983 | *BNIP3* | BCL2/adenovirus E1B 19 kDa protein-interacting protein 3 OS=Homo sapiens GN=BNIP3 PE=1 SV=2 - [BNIP3_HUMAN] | 1.240 |
| O14647 | *CHD2* | Isoform 2 of Chromodomain-helicase-DNA-binding protein 2 OS=Homo sapiens GN=CHD2 - [CHD2_HUMAN] | 1.138 |
| Q9Y570 | *PPME1* | Protein phosphatase methylesterase 1 OS=Homo sapiens GN=PPME1 PE=1 SV=3 - [PPME1_HUMAN] | 1.204 |
| Q9NZN4 | *EHD2* | EH domain-containing protein 2 OS=Homo sapiens GN=EHD2 PE=1 SV=2 - [EHD2_HUMAN] | 0.555 |
| P63267 | *ACTG2* | Actin, gamma-enteric smooth muscle OS=Homo sapiens GN=ACTG2 PE=1 SV=1 - [ACTH_HUMAN] | 1.233 |
| Q9UNY4 | *TTF2* | Isoform 2 of Transcription termination factor 2 OS=Homo sapiens GN=TTF2 - [TTF2_HUMAN] | 0.285 |
| P08174 | *CD55* | Complement decay-accelerating factor OS=Homo sapiens GN=CD55 PE=1 SV=4 - [DAF_HUMAN] | 0.584 |
| Q15276 | *RABEP1* | Isoform 2 of Rab GTPase-binding effector protein 1 OS=Homo sapiens GN=RABEP1 - [RABE1_HUMAN] | 0.941 |
| P40121 | *CAPG* | Macrophage-capping protein OS=Homo sapiens GN=CAPG PE=1 SV=2 - [CAPG_HUMAN] | 0.682 |
| Q5SZQ8 | *CELF3* | Isoform 2 of CUGBP Elav-like family member 3 OS=Homo sapiens GN=CELF3 - [CELF3_HUMAN] | 0.717 |
| Q9BSA9 | *TMEM175* | Transmembrane protein 175 OS=Homo sapiens GN=TMEM175 PE=1 SV=1 - [TM175_HUMAN] | 0.842 |
| P0DJ07 | *PET100* | Uncharacterized protein C19orf79 OS=Homo sapiens GN=C19orf79 PE=4 SV=1 - [CS079_HUMAN] | 0.763 |
| P11441 | *UBL4A* | Ubiquitin-like protein 4A OS=Homo sapiens GN=UBL4A PE=1 SV=1 - [UBL4A_HUMAN] | 1.116 |
| Q9NT62 | *ATG3* | Isoform 2 of Ubiquitin-like-conjugating enzyme ATG3 OS=Homo sapiens GN=ATG3 - [ATG3_HUMAN] | 0.880 |
| P04179 | *SOD2* | Superoxide dismutase [Mn], mitochondrial OS=Homo sapiens GN=SOD2 PE=1 SV=2 - [SODM_HUMAN] | 0.857 |
| P51608 | *MECP2* | Methyl-CpG-binding protein 2 OS=Homo sapiens GN=MECP2 PE=1 SV=1 - [MECP2_HUMAN] | 0.845 |
| O95166 | *GABARAP* | Gamma-aminobutyric acid receptor-associated protein OS=Homo sapiens GN=GABARAP PE=1 SV=1 - [GBRAP_HUMAN] | 0.882 |
| P11387 | *TOP1* | DNA topoisomerase 1 OS=Homo sapiens GN=TOP1 PE=1 SV=2 - [TOP1_HUMAN] | 1.182 |
| Q9UNF1 | *MAGED2* | Isoform 2 of Melanoma-associated antigen D2 OS=Homo sapiens GN=MAGED2 - [MAGD2_HUMAN] | 1.119 |
| Q8WVY7 | *UBLCP1* | Ubiquitin-like domain-containing CTD phosphatase 1 OS=Homo sapiens GN=UBLCP1 PE=1 SV=2 - [UBCP1_HUMAN] | 1.162 |
| Q9UKY7 | *CDV3* | Protein CDV3 homolog OS=Homo sapiens GN=CDV3 PE=1 SV=1 - [CDV3_HUMAN] | 0.887 |
| P31947 | *SFN* | Isoform 2 of 14-3-3 protein sigma OS=Homo sapiens GN=SFN - [1433S_HUMAN] | 8.999 |
| P00367 | *GLUD1* | Glutamate dehydrogenase 1, mitochondrial OS=Homo sapiens GN=GLUD1 PE=1 SV=2 - [DHE3_HUMAN] | 0.819 |
| P10155 | *TROVE2* | 60 kDa SS-A/Ro ribonucleoprotein OS=Homo sapiens GN=TROVE2 PE=1 SV=2 - [RO60_HUMAN] | 1.055 |
| Q9UL62 | *TRPC5* | Short transient receptor potential channel 5 OS=Homo sapiens GN=TRPC5 PE=1 SV=1 - [TRPC5_HUMAN] | 0.543 |
| O00244 | *ATOX1* | Copper transport protein ATOX1 OS=Homo sapiens GN=ATOX1 PE=1 SV=1 - [ATOX1_HUMAN] | 0.876 |
| O60292 | *SIPA1L3* | Signal-induced proliferation-associated 1-like protein 3 OS=Homo sapiens GN=SIPA1L3 PE=1 SV=3 - [SI1L3_HUMAN] | 1.237 |
| Q92673 | *SORL1* | Sortilin-related receptor OS=Homo sapiens GN=SORL1 PE=1 SV=2 - [SORL_HUMAN] | 1.447 |
| P48449 | *LSS* | Lanosterol synthase OS=Homo sapiens GN=LSS PE=1 SV=1 - [ERG7_HUMAN] | 1.138 |
| P31937 | *HIBADH* | 3-hydroxyisobutyrate dehydrogenase, mitochondrial OS=Homo sapiens GN=HIBADH PE=1 SV=2 - [3HIDH_HUMAN] | 0.890 |
| P19105 | *MYL12A* | Myosin regulatory light chain 12A OS=Homo sapiens GN=MYL12A PE=1 SV=2 - [ML12A_HUMAN] | 1.215 |
| Q9BQS8 | *FYCO1* | FYVE and coiled-coil domain-containing protein 1 OS=Homo sapiens GN=FYCO1 PE=1 SV=3 - [FYCO1_HUMAN] | 0.853 |
| Q14974 | *KPNB1* | Importin subunit beta-1 OS=Homo sapiens GN=KPNB1 PE=1 SV=2 - [IMB1_HUMAN] | 1.128 |
| Q8TB72 | *PUM2* | Isoform 2 of Pumilio homolog 2 OS=Homo sapiens GN=PUM2 - [PUM2_HUMAN] | 1.095 |
| Q01970 | *PLCB3* | 1-phosphatidylinositol-4,5-bisphosphate phosphodiesterase beta-3 OS=Homo sapiens GN=PLCB3 PE=1 SV=2 - [PLCB3_HUMAN] | 1.214 |
| P14854 | *COX6B1* | Cytochrome c oxidase subunit 6B1 OS=Homo sapiens GN=COX6B1 PE=1 SV=2 - [CX6B1_HUMAN] | 0.795 |
| Q7Z404 | *0* | Isoform 2 of Transmembrane channel-like protein 4 OS=Homo sapiens GN=TMC4 - [TMC4_HUMAN] | 0.872 |
| Q08752 | *PPID* | Peptidyl-prolyl cis-trans isomerase D OS=Homo sapiens GN=PPID PE=1 SV=3 - [PPID_HUMAN] | 1.260 |
| Q96KR6 | *FAM210B* | Protein FAM210B OS=Homo sapiens GN=FAM210B PE=1 SV=2 - [F210B_HUMAN] | 0.640 |
| O15440 | *ABCC5* | Multidrug resistance-associated protein 5 OS=Homo sapiens GN=ABCC5 PE=1 SV=2 - [MRP5_HUMAN] | 0.738 |
| O15270 | *SPTLC2* | Serine palmitoyltransferase 2 OS=Homo sapiens GN=SPTLC2 PE=1 SV=1 - [SPTC2_HUMAN] | 1.336 |
| P61254 | *RPL26* | 60S ribosomal protein L26 OS=Homo sapiens GN=RPL26 PE=1 SV=1 - [RL26_HUMAN] | 1.080 |
| P01596 | *#N/A* | Ig kappa chain V-I region CAR OS=Homo sapiens PE=1 SV=1 - [KV104_HUMAN] | 0.659 |
| O43617 | *TRAPPC3* | Trafficking protein particle complex subunit 3 OS=Homo sapiens GN=TRAPPC3 PE=1 SV=1 - [TPPC3_HUMAN] | 1.116 |
| Q9Y296 | *TRAPPC4* | Trafficking protein particle complex subunit 4 OS=Homo sapiens GN=TRAPPC4 PE=1 SV=1 - [TPPC4_HUMAN] | 1.150 |
| P49321 | *NASP* | Isoform 2 of Nuclear autoantigenic sperm protein OS=Homo sapiens GN=NASP - [NASP_HUMAN] | 1.284 |
| Q9UEW8 | *STK39* | STE20/SPS1-related proline-alanine-rich protein kinase OS=Homo sapiens GN=STK39 PE=1 SV=3 - [STK39_HUMAN] | 1.603 |
| Q8N729 | *NPW* | Neuropeptide W OS=Homo sapiens GN=NPW PE=1 SV=2 - [NPW_HUMAN] | 0.406 |
| P23193 | *TCEA1* | Isoform 2 of Transcription elongation factor A protein 1 OS=Homo sapiens GN=TCEA1 - [TCEA1_HUMAN] | 1.147 |
| P42356 | *#N/A* | Phosphatidylinositol 4-kinase alpha OS=Homo sapiens GN=PI4KA PE=1 SV=3 - [PI4KA_HUMAN] | 0.883 |
| Q9NW82 | *WDR70* | WD repeat-containing protein 70 OS=Homo sapiens GN=WDR70 PE=1 SV=1 - [WDR70_HUMAN] | 1.214 |
| Q8N183 | *NDUFAF2* | Mimitin, mitochondrial OS=Homo sapiens GN=NDUFAF2 PE=1 SV=1 - [MIMIT_HUMAN] | 0.768 |
| P17980 | *PSMC3* | 26S protease regulatory subunit 6A OS=Homo sapiens GN=PSMC3 PE=1 SV=3 - [PRS6A_HUMAN] | 1.075 |
| P31751 | *AKT2* | RAC-beta serine/threonine-protein kinase OS=Homo sapiens GN=AKT2 PE=1 SV=2 - [AKT2_HUMAN] | 0.872 |
| O76021 | *RSL1D1* | Ribosomal L1 domain-containing protein 1 OS=Homo sapiens GN=RSL1D1 PE=1 SV=3 - [RL1D1_HUMAN] | 1.190 |
| P17174 | *GOT1* | Aspartate aminotransferase, cytoplasmic OS=Homo sapiens GN=GOT1 PE=1 SV=3 - [AATC_HUMAN] | 1.304 |
| P19634 | *SLC9A1* | Sodium/hydrogen exchanger 1 OS=Homo sapiens GN=SLC9A1 PE=1 SV=2 - [SL9A1_HUMAN] | 0.845 |
| P55287 | *CDH11* | Isoform 2 of Cadherin-11 OS=Homo sapiens GN=CDH11 - [CAD11_HUMAN] | 1.413 |
| Q14651 | *PLS1* | Plastin-1 OS=Homo sapiens GN=PLS1 PE=1 SV=2 - [PLSI_HUMAN] | 0.663 |
| P24534 | *EEF1B2* | Elongation factor 1-beta OS=Homo sapiens GN=EEF1B2 PE=1 SV=3 - [EF1B_HUMAN] | 1.121 |
| O75781 | *PALM* | Isoform 2 of Paralemmin-1 OS=Homo sapiens GN=PALM - [PALM_HUMAN] | 0.712 |
| Q96K21 | *ZFYVE19* | Isoform 3 of Zinc finger FYVE domain-containing protein 19 OS=Homo sapiens GN=ZFYVE19 - [ZFY19_HUMAN] | 1.147 |
| P10606 | *COX5B* | Cytochrome c oxidase subunit 5B, mitochondrial OS=Homo sapiens GN=COX5B PE=1 SV=2 - [COX5B_HUMAN] | 0.842 |
| Q9UKF6 | *CPSF3* | Cleavage and polyadenylation specificity factor subunit 3 OS=Homo sapiens GN=CPSF3 PE=1 SV=1 - [CPSF3_HUMAN] | 1.115 |
| P06730 | *EIF4E* | Eukaryotic translation initiation factor 4E OS=Homo sapiens GN=EIF4E PE=1 SV=2 - [IF4E_HUMAN] | 0.864 |
| Q9NQS3 | *PVRL3* | Poliovirus receptor-related protein 3 OS=Homo sapiens GN=PVRL3 PE=1 SV=1 - [PVRL3_HUMAN] | 0.686 |
| O94901 | *SUN1* | Isoform 5 of SUN domain-containing protein 1 OS=Homo sapiens GN=SUN1 - [SUN1_HUMAN] | 0.876 |
| Q6ZQQ6 | *WDR87* | WD repeat-containing protein 87 OS=Homo sapiens GN=WDR87 PE=2 SV=3 - [WDR87_HUMAN] | 0.763 |
| Q96MW5 | *COG8* | Conserved oligomeric Golgi complex subunit 8 OS=Homo sapiens GN=COG8 PE=1 SV=2 - [COG8_HUMAN] | 1.106 |
| P04632 | *CAPNS1* | Calpain small subunit 1 OS=Homo sapiens GN=CAPNS1 PE=1 SV=1 - [CPNS1_HUMAN] | 1.212 |
| O14649 | *KCNK3* | Potassium channel subfamily K member 3 OS=Homo sapiens GN=KCNK3 PE=1 SV=1 - [KCNK3_HUMAN] | 0.803 |
| Q13822 | *ENPP2* | Ectonucleotide pyrophosphatase/phosphodiesterase family member 2 OS=Homo sapiens GN=ENPP2 PE=1 SV=3 - [ENPP2_HUMAN] | 0.404 |
| O75175 | *CNOT3* | Isoform 2 of CCR4-NOT transcription complex subunit 3 OS=Homo sapiens GN=CNOT3 - [CNOT3_HUMAN] | 1.091 |
| O60237 | *PPP1R12B* | Isoform 4 of Protein phosphatase 1 regulatory subunit 12B OS=Homo sapiens GN=PPP1R12B - [MYPT2_HUMAN] | 0.717 |
| P08575 | *PTPRC* | Isoform 2 of Receptor-type tyrosine-protein phosphatase C OS=Homo sapiens GN=PTPRC - [PTPRC_HUMAN] | 0.755 |
| Q9BS40 | *LXN* | Latexin OS=Homo sapiens GN=LXN PE=1 SV=2 - [LXN_HUMAN] | 0.849 |
| Q96AQ8 | *MCUR1* | Coiled-coil domain-containing protein 90A, mitochondrial OS=Homo sapiens GN=CCDC90A PE=2 SV=1 - [CC90A_HUMAN] | 1.258 |
| Q13045 | *FLII* | Protein flightless-1 homolog OS=Homo sapiens GN=FLII PE=1 SV=2 - [FLII_HUMAN] | 1.188 |
| Q9NYK5 | *MRPL39* | 39S ribosomal protein L39, mitochondrial OS=Homo sapiens GN=MRPL39 PE=1 SV=3 - [RM39_HUMAN] | 0.915 |
| P35222 | *CTNNB1* | Catenin beta-1 OS=Homo sapiens GN=CTNNB1 PE=1 SV=1 - [CTNB1_HUMAN] | 0.822 |
| Q9Y5B9 | *SUPT16H* | FACT complex subunit SPT16 OS=Homo sapiens GN=SUPT16H PE=1 SV=1 - [SP16H_HUMAN] | 1.107 |
| Q9BY44 | *EIF2A* | Isoform 2 of Eukaryotic translation initiation factor 2A OS=Homo sapiens GN=EIF2A - [EIF2A_HUMAN] | 0.850 |
| P48643 | *CCT5* | T-complex protein 1 subunit epsilon OS=Homo sapiens GN=CCT5 PE=1 SV=1 - [TCPE_HUMAN] | 1.093 |
| Q3SY69 | *ALDH1L2* | Isoform 2 of Mitochondrial 10-formyltetrahydrofolate dehydrogenase OS=Homo sapiens GN=ALDH1L2 - [AL1L2_HUMAN] | 1.585 |
| P13591 | *NCAM1* | Isoform 2 of Neural cell adhesion molecule 1 OS=Homo sapiens GN=NCAM1 - [NCAM1_HUMAN] | 0.785 |
| P13521 | *SCG2* | Secretogranin-2 OS=Homo sapiens GN=SCG2 PE=1 SV=2 - [SCG2_HUMAN] | 0.508 |
| P08727 | *KRT19* | Keratin, type I cytoskeletal 19 OS=Homo sapiens GN=KRT19 PE=1 SV=4 - [K1C19_HUMAN] | 2.567 |
| P07942 | *LAMB1* | Laminin subunit beta-1 OS=Homo sapiens GN=LAMB1 PE=1 SV=2 - [LAMB1_HUMAN] | 0.656 |
| Q969P0 | *IGSF8* | Isoform 3 of Immunoglobulin superfamily member 8 OS=Homo sapiens GN=IGSF8 - [IGSF8_HUMAN] | 0.792 |
| O94864 | *SUPT7L* | Isoform 2 of STAGA complex 65 subunit gamma OS=Homo sapiens GN=SUPT7L - [ST65G_HUMAN] | 1.110 |
| O95155 | *UBE4B* | Isoform 3 of Ubiquitin conjugation factor E4 B OS=Homo sapiens GN=UBE4B - [UBE4B_HUMAN] | 1.137 |
| Q8NDI1 | *EHBP1* | Isoform 3 of EH domain-binding protein 1 OS=Homo sapiens GN=EHBP1 - [EHBP1_HUMAN] | 1.112 |
| P55103 | *INHBC* | Inhibin beta C chain OS=Homo sapiens GN=INHBC PE=1 SV=1 - [INHBC_HUMAN] | 0.512 |
| Q9Y3R5 | *DOPEY2* | Isoform 2 of Protein dopey-2 OS=Homo sapiens GN=DOPEY2 - [DOP2_HUMAN] | 1.289 |
| O94855 | *SEC24D* | Protein transport protein Sec24D OS=Homo sapiens GN=SEC24D PE=1 SV=2 - [SC24D_HUMAN] | 0.865 |
| P47756 | *CAPZB* | Isoform 2 of F-actin-capping protein subunit beta OS=Homo sapiens GN=CAPZB - [CAPZB_HUMAN] | 1.110 |
| O15042 | *U2SURP* | Isoform 2 of U2 snRNP-associated SURP motif-containing protein OS=Homo sapiens GN=U2SURP - [SR140_HUMAN] | 1.104 |
| Q6P996 | *PDXDC1* | Pyridoxal-dependent decarboxylase domain-containing protein 1 OS=Homo sapiens GN=PDXDC1 PE=1 SV=2 - [PDXD1_HUMAN] | 1.661 |
| O43181 | *NDUFS4* | NADH dehydrogenase [ubiquinone] iron-sulfur protein 4, mitochondrial OS=Homo sapiens GN=NDUFS4 PE=1 SV=1 - [NDUS4_HUMAN] | 0.871 |
| Q8N442 | *GUF1* | Translation factor GUF1, mitochondrial OS=Homo sapiens GN=GUF1 PE=1 SV=1 - [GUF1_HUMAN] | 1.559 |
| Q68CP9 | *ARID2* | Isoform 2 of AT-rich interactive domain-containing protein 2 OS=Homo sapiens GN=ARID2 - [ARID2_HUMAN] | 1.117 |
| Q9UKF7 | *PITPNC1* | Isoform 2 of Cytoplasmic phosphatidylinositol transfer protein 1 OS=Homo sapiens GN=PITPNC1 - [PITC1_HUMAN] | 1.312 |
| O75390 | *CS* | Citrate synthase, mitochondrial OS=Homo sapiens GN=CS PE=1 SV=2 - [CISY_HUMAN] | 1.181 |
| Q8IZ83 | *ALDH16A1* | Aldehyde dehydrogenase family 16 member A1 OS=Homo sapiens GN=ALDH16A1 PE=1 SV=2 - [A16A1_HUMAN] | 1.128 |
| P05546 | *SERPIND1* | Heparin cofactor 2 OS=Homo sapiens GN=SERPIND1 PE=1 SV=3 - [HEP2_HUMAN] | 0.696 |
| O95613 | *PCNT* | Isoform 2 of Pericentrin OS=Homo sapiens GN=PCNT - [PCNT_HUMAN] | 0.890 |
| P43490 | *NAMPT* | Nicotinamide phosphoribosyltransferase OS=Homo sapiens GN=NAMPT PE=1 SV=1 - [NAMPT_HUMAN] | 1.516 |
| P43686 | *PSMC4* | 26S protease regulatory subunit 6B OS=Homo sapiens GN=PSMC4 PE=1 SV=2 - [PRS6B_HUMAN] | 1.101 |
| Q7L5D6 | *GET4* | Isoform 2 of Golgi to ER traffic protein 4 homolog OS=Homo sapiens GN=GET4 - [GET4_HUMAN] | 1.106 |
| Q9HCG8 | *CWC22* | Pre-mRNA-splicing factor CWC22 homolog OS=Homo sapiens GN=CWC22 PE=1 SV=3 - [CWC22_HUMAN] | 1.129 |
| P14735 | *IDE* | Insulin-degrading enzyme OS=Homo sapiens GN=IDE PE=1 SV=4 - [IDE_HUMAN] | 1.294 |
| Q7Z4S6 | *KIF21A* | Isoform 3 of Kinesin-like protein KIF21A OS=Homo sapiens GN=KIF21A - [KI21A_HUMAN] | 0.824 |
| Q14697 | *GANAB* | Isoform 2 of Neutral alpha-glucosidase AB OS=Homo sapiens GN=GANAB - [GANAB_HUMAN] | 1.584 |
| Q9NTZ6 | *RBM12* | RNA-binding protein 12 OS=Homo sapiens GN=RBM12 PE=1 SV=1 - [RBM12_HUMAN] | 1.094 |
| P27824 | *CANX* | Calnexin OS=Homo sapiens GN=CANX PE=1 SV=2 - [CALX_HUMAN] | 1.159 |
| O75368 | *SH3BGRL* | SH3 domain-binding glutamic acid-rich-like protein OS=Homo sapiens GN=SH3BGRL PE=1 SV=1 - [SH3L1_HUMAN] | 0.858 |
| Q15814 | *TBCC* | Tubulin-specific chaperone C OS=Homo sapiens GN=TBCC PE=1 SV=2 - [TBCC_HUMAN] | 1.185 |
| O75116 | *ROCK2* | Rho-associated protein kinase 2 OS=Homo sapiens GN=ROCK2 PE=1 SV=4 - [ROCK2_HUMAN] | 1.198 |
| P43487 | *RANBP1* | Ran-specific GTPase-activating protein OS=Homo sapiens GN=RANBP1 PE=1 SV=1 - [RANG_HUMAN] | 1.162 |
| O60504 | *SORBS3* | Vinexin OS=Homo sapiens GN=SORBS3 PE=1 SV=2 - [VINEX_HUMAN] | 0.785 |
| Q9P2K5 | *MYEF2* | Isoform 2 of Myelin expression factor 2 OS=Homo sapiens GN=MYEF2 - [MYEF2_HUMAN] | 1.202 |
| P09622 | *DLD* | Dihydrolipoyl dehydrogenase, mitochondrial OS=Homo sapiens GN=DLD PE=1 SV=2 - [DLDH_HUMAN] | 1.103 |
| O43602 | *DCX* | Isoform 2 of Neuronal migration protein doublecortin OS=Homo sapiens GN=DCX - [DCX_HUMAN] | 1.286 |
| Q9H6R4 | *NOL6* | Isoform 2 of Nucleolar protein 6 OS=Homo sapiens GN=NOL6 - [NOL6_HUMAN] | 1.149 |
| O75380 | *NDUFS6* | NADH dehydrogenase [ubiquinone] iron-sulfur protein 6, mitochondrial OS=Homo sapiens GN=NDUFS6 PE=1 SV=1 - [NDUS6_HUMAN] | 0.836 |
| O94979 | *SEC31A* | Isoform 6 of Protein transport protein Sec31A OS=Homo sapiens GN=SEC31A - [SC31A_HUMAN] | 0.885 |
| Q5TZA2 | *CROCC* | Rootletin OS=Homo sapiens GN=CROCC PE=1 SV=1 - [CROCC_HUMAN] | 0.852 |
| Q63ZY3 | *KANK2* | Isoform 3 of KN motif and ankyrin repeat domain-containing protein 2 OS=Homo sapiens GN=KANK2 - [KANK2_HUMAN] | 0.738 |
| P19367 | *HK1* | Isoform 4 of Hexokinase-1 OS=Homo sapiens GN=HK1 - [HXK1_HUMAN] | 1.408 |
| P35052 | *GPC1* | Glypican-1 OS=Homo sapiens GN=GPC1 PE=1 SV=2 - [GPC1_HUMAN] | 0.745 |
| P02679 | *FGG* | Isoform Gamma-A of Fibrinogen gamma chain OS=Homo sapiens GN=FGG - [FIBG_HUMAN] | 0.530 |
| Q13185 | *CBX3* | Chromobox protein homolog 3 OS=Homo sapiens GN=CBX3 PE=1 SV=4 - [CBX3_HUMAN] | 1.177 |
| P06737 | *PYGL* | Glycogen phosphorylase, liver form OS=Homo sapiens GN=PYGL PE=1 SV=4 - [PYGL_HUMAN] | 0.605 |
| P02649 | *APOE* | Apolipoprotein E OS=Homo sapiens GN=APOE PE=1 SV=1 - [APOE_HUMAN] | 0.463 |
| Q14789 | *GOLGB1* | Golgin subfamily B member 1 OS=Homo sapiens GN=GOLGB1 PE=1 SV=2 - [GOGB1_HUMAN] | 0.900 |
| Q32MZ4 | *LRRFIP1* | Isoform 3 of Leucine-rich repeat flightless-interacting protein 1 OS=Homo sapiens GN=LRRFIP1 - [LRRF1_HUMAN] | 1.304 |
| Q86SK9 | *SCD5* | Isoform 2 of Stearoyl-CoA desaturase 5 OS=Homo sapiens GN=SCD5 - [SCD5_HUMAN] | 0.530 |
| Q8WXX5 | *DNAJC9* | DnaJ homolog subfamily C member 9 OS=Homo sapiens GN=DNAJC9 PE=1 SV=1 - [DNJC9_HUMAN] | 0.889 |
| Q9BU76 | *C1orf35* | Isoform 2 of Multiple myeloma tumor-associated protein 2 OS=Homo sapiens GN=MMTAG2 - [MMTA2_HUMAN] | 1.319 |
| P55735 | *SEC13* | Protein SEC13 homolog OS=Homo sapiens GN=SEC13 PE=1 SV=3 - [SEC13_HUMAN] | 0.917 |
| P40763 | *STAT3* | Isoform Del-701 of Signal transducer and activator of transcription 3 OS=Homo sapiens GN=STAT3 - [STAT3_HUMAN] | 1.201 |
| Q14966 | *ZNF638* | Isoform 3 of Zinc finger protein 638 OS=Homo sapiens GN=ZNF638 - [ZN638_HUMAN] | 1.126 |
| Q6P2P2 | *PRMT9* | Putative protein arginine N-methyltransferase 10 OS=Homo sapiens GN=PRMT10 PE=1 SV=1 - [ANM10_HUMAN] | 1.082 |
| Q8WWI1 | *LMO7* | Isoform 3 of LIM domain only protein 7 OS=Homo sapiens GN=LMO7 - [LMO7_HUMAN] | 1.158 |
| Q9H307 | *PNN* | Pinin OS=Homo sapiens GN=PNN PE=1 SV=4 - [PININ_HUMAN] | 1.095 |
| Q9NRF8 | *CTPS2* | CTP synthase 2 OS=Homo sapiens GN=CTPS2 PE=1 SV=1 - [PYRG2_HUMAN] | 1.164 |
| Q9BV44 | *THUMPD3* | THUMP domain-containing protein 3 OS=Homo sapiens GN=THUMPD3 PE=1 SV=1 - [THUM3_HUMAN] | 0.863 |
| Q12788 | *TBL3* | Transducin beta-like protein 3 OS=Homo sapiens GN=TBL3 PE=1 SV=2 - [TBL3_HUMAN] | 1.167 |
| Q02083 | *NAAA* | Isoform 3 of N-acylethanolamine-hydrolyzing acid amidase OS=Homo sapiens GN=NAAA - [NAAA_HUMAN] | 0.782 |
| P49023 | *PXN* | Isoform Alpha of Paxillin OS=Homo sapiens GN=PXN - [PAXI_HUMAN] | 0.823 |
| Q96GC5 | *MRPL48* | 39S ribosomal protein L48, mitochondrial OS=Homo sapiens GN=MRPL48 PE=1 SV=2 - [RM48_HUMAN] | 0.905 |
| Q9NU23 | *LYRM2* | LYR motif-containing protein 2 OS=Homo sapiens GN=LYRM2 PE=1 SV=1 - [LYRM2_HUMAN] | 0.814 |
| Q9BWF3 | *RBM4* | RNA-binding protein 4 OS=Homo sapiens GN=RBM4 PE=1 SV=1 - [RBM4_HUMAN] | 1.152 |
| A8MSI8 | *LYRM9* | UPF0631 protein C17orf108 OS=Homo sapiens GN=C17orf108 PE=3 SV=2 - [CQ108_HUMAN] | 0.632 |
| P07996 | *THBS1* | Thrombospondin-1 OS=Homo sapiens GN=THBS1 PE=1 SV=2 - [TSP1_HUMAN] | 3.008 |
| Q8TEX9 | *IPO4* | Importin-4 OS=Homo sapiens GN=IPO4 PE=1 SV=2 - [IPO4_HUMAN] | 1.113 |
| P54619 | *PRKAG1* | 5'-AMP-activated protein kinase subunit gamma-1 OS=Homo sapiens GN=PRKAG1 PE=1 SV=1 - [AAKG1_HUMAN] | 1.115 |
| P52757 | *CHN2* | Beta-chimaerin OS=Homo sapiens GN=CHN2 PE=1 SV=2 - [CHIO_HUMAN] | 0.755 |
| Q99933 | *BAG1* | Isoform 4 of BAG family molecular chaperone regulator 1 OS=Homo sapiens GN=BAG1 - [BAG1_HUMAN] | 0.812 |
| P30520 | *ADSS* | Adenylosuccinate synthetase isozyme 2 OS=Homo sapiens GN=ADSS PE=1 SV=3 - [PURA2_HUMAN] | 0.859 |
| O75223 | *GGCT* | Gamma-glutamylcyclotransferase OS=Homo sapiens GN=GGCT PE=1 SV=1 - [GGCT_HUMAN] | 1.288 |
| O95671 | *ASMTL* | Isoform 2 of N-acetylserotonin O-methyltransferase-like protein OS=Homo sapiens GN=ASMTL - [ASML_HUMAN] | 0.846 |
| P49747 | *COMP* | Cartilage oligomeric matrix protein OS=Homo sapiens GN=COMP PE=1 SV=2 - [COMP_HUMAN] | 0.432 |
| O95376 | *ARIH2* | E3 ubiquitin-protein ligase ARIH2 OS=Homo sapiens GN=ARIH2 PE=1 SV=1 - [ARI2_HUMAN] | 1.683 |
| Q6YN16 | *HSDL2* | Isoform 2 of Hydroxysteroid dehydrogenase-like protein 2 OS=Homo sapiens GN=HSDL2 - [HSDL2_HUMAN] | 0.838 |
| P50238 | *CRIP1* | Cysteine-rich protein 1 OS=Homo sapiens GN=CRIP1 PE=1 SV=3 - [CRIP1_HUMAN] | 0.771 |
| P54289 | *CACNA2D1* | Isoform 4 of Voltage-dependent calcium channel subunit alpha-2/delta-1 OS=Homo sapiens GN=CACNA2D1 - [CA2D1_HUMAN] | 0.807 |
| O43396 | *TXNL1* | Thioredoxin-like protein 1 OS=Homo sapiens GN=TXNL1 PE=1 SV=3 - [TXNL1_HUMAN] | 0.836 |
| P00403 | *MT-CO2* | Cytochrome c oxidase subunit 2 OS=Homo sapiens GN=MT-CO2 PE=1 SV=1 - [COX2_HUMAN] | 0.841 |
| P40306 | *PSMB10* | Proteasome subunit beta type-10 OS=Homo sapiens GN=PSMB10 PE=1 SV=1 - [PSB10_HUMAN] | 1.443 |
| P01611 | *#N/A* | Ig kappa chain V-I region Wes OS=Homo sapiens PE=1 SV=1 - [KV119_HUMAN] | 0.746 |
| Q9UI09 | *NDUFA12* | NADH dehydrogenase [ubiquinone] 1 alpha subcomplex subunit 12 OS=Homo sapiens GN=NDUFA12 PE=1 SV=1 - [NDUAC_HUMAN] | 0.890 |
| Q53TN4 | *CYBRD1* | Cytochrome b reductase 1 OS=Homo sapiens GN=CYBRD1 PE=1 SV=1 - [CYBR1_HUMAN] | 0.551 |
| P02749 | *APOH* | Beta-2-glycoprotein 1 OS=Homo sapiens GN=APOH PE=1 SV=3 - [APOH_HUMAN] | 0.778 |
| Q08174 | *PCDH1* | Isoform 2 of Protocadherin-1 OS=Homo sapiens GN=PCDH1 - [PCDH1_HUMAN] | 0.724 |
| P63104 | *YWHAZ* | 14-3-3 protein zeta/delta OS=Homo sapiens GN=YWHAZ PE=1 SV=1 - [1433Z_HUMAN] | 0.471 |
| Q6P6C2 | *ALKBH5* | Isoform 2 of Probable alpha-ketoglutarate-dependent dioxygenase ABH5 OS=Homo sapiens GN=ALKBH5 - [ALKB5_HUMAN] | 0.825 |
| Q92841 | *DDX17* | Isoform 4 of Probable ATP-dependent RNA helicase DDX17 OS=Homo sapiens GN=DDX17 - [DDX17_HUMAN] | 1.083 |
| P61803 | *DAD1* | Dolichyl-diphosphooligosaccharide--protein glycosyltransferase subunit DAD1 OS=Homo sapiens GN=DAD1 PE=1 SV=3 - [DAD1_HUMAN] | 0.909 |
| P43121 | *MCAM* | Cell surface glycoprotein MUC18 OS=Homo sapiens GN=MCAM PE=1 SV=2 - [MUC18_HUMAN] | 0.779 |
| Q567U6 | *CCDC93* | Coiled-coil domain-containing protein 93 OS=Homo sapiens GN=CCDC93 PE=1 SV=2 - [CCD93_HUMAN] | 0.917 |
| O60524 | *NEMF* | Isoform 4 of Nuclear export mediator factor NEMF OS=Homo sapiens GN=NEMF - [NEMF_HUMAN] | 0.931 |
| Q07866 | *KLC1* | Isoform N of Kinesin light chain 1 OS=Homo sapiens GN=KLC1 - [KLC1_HUMAN] | 1.303 |
| P40818 | *USP8* | Ubiquitin carboxyl-terminal hydrolase 8 OS=Homo sapiens GN=USP8 PE=1 SV=1 - [UBP8_HUMAN] | 1.160 |
| O75427 | *LRCH4* | Leucine-rich repeat and calponin homology domain-containing protein 4 OS=Homo sapiens GN=LRCH4 PE=1 SV=2 - [LRCH4_HUMAN] | 1.313 |
| Q9Y4G6 | *TLN2* | Talin-2 OS=Homo sapiens GN=TLN2 PE=1 SV=4 - [TLN2_HUMAN] | 0.783 |
| Q8WVM8 | *SCFD1* | Sec1 family domain-containing protein 1 OS=Homo sapiens GN=SCFD1 PE=1 SV=4 - [SCFD1_HUMAN] | 1.079 |
| Q8IXB1 | *DNAJC10* | Isoform 2 of DnaJ homolog subfamily C member 10 OS=Homo sapiens GN=DNAJC10 - [DJC10_HUMAN] | 1.191 |
| Q96AG3 | *SLC25A46* | Solute carrier family 25 member 46 OS=Homo sapiens GN=SLC25A46 PE=1 SV=1 - [S2546_HUMAN] | 0.836 |
| P0CG05 | *#N/A* | Ig lambda-2 chain C regions OS=Homo sapiens GN=IGLC2 PE=1 SV=1 - [LAC2_HUMAN] | 0.702 |
| O75347 | *TBCA* | Tubulin-specific chaperone A OS=Homo sapiens GN=TBCA PE=1 SV=3 - [TBCA_HUMAN] | 1.333 |
| P05362 | *ICAM1* | Intercellular adhesion molecule 1 OS=Homo sapiens GN=ICAM1 PE=1 SV=2 - [ICAM1_HUMAN] | 0.579 |
| Q15286 | *RAB35* | Ras-related protein Rab-35 OS=Homo sapiens GN=RAB35 PE=1 SV=1 - [RAB35_HUMAN] | 0.893 |
| Q9BQ75 | *CMSS1* | Uncharacterized protein C3orf26 OS=Homo sapiens GN=C3orf26 PE=1 SV=2 - [CC026_HUMAN] | 0.882 |
| Q13620 | *CUL4B* | Isoform 3 of Cullin-4B OS=Homo sapiens GN=CUL4B - [CUL4B_HUMAN] | 0.735 |
| Q9Y6I3 | *EPN1* | Isoform 3 of Epsin-1 OS=Homo sapiens GN=EPN1 - [EPN1_HUMAN] | 1.171 |
| P15090 | *FABP4* | Fatty acid-binding protein, adipocyte OS=Homo sapiens GN=FABP4 PE=1 SV=3 - [FABP4_HUMAN] | 0.230 |
| P25786 | *PSMA1* | Proteasome subunit alpha type-1 OS=Homo sapiens GN=PSMA1 PE=1 SV=1 - [PSA1_HUMAN] | 1.099 |
| Q8WXF0 | *SRSF12* | Serine/arginine-rich splicing factor 12 OS=Homo sapiens GN=SRSF12 PE=1 SV=1 - [SRS12_HUMAN] | 1.435 |
| P30876 | *POLR2B* | DNA-directed RNA polymerase II subunit RPB2 OS=Homo sapiens GN=POLR2B PE=1 SV=1 - [RPB2_HUMAN] | 1.074 |
| Q9BW04 | *C1orf116* | Isoform 2 of Specifically androgen-regulated gene protein OS=Homo sapiens GN=SARG - [SARG_HUMAN] | 1.434 |
| Q8TEQ0 | *SNX29* | Isoform 2 of Sorting nexin-29 OS=Homo sapiens GN=SNX29 - [SNX29_HUMAN] | 1.289 |
| Q9BXX0 | *EMILIN2* | EMILIN-2 OS=Homo sapiens GN=EMILIN2 PE=1 SV=3 - [EMIL2_HUMAN] | 0.658 |
| P61769 | *B2M* | Beta-2-microglobulin OS=Homo sapiens GN=B2M PE=1 SV=1 - [B2MG_HUMAN] | 0.652 |
| P61247 | *RPS3A* | 40S ribosomal protein S3a OS=Homo sapiens GN=RPS3A PE=1 SV=2 - [RS3A_HUMAN] | 1.073 |
| Q13492 | *PICALM* | Isoform 3 of Phosphatidylinositol-binding clathrin assembly protein OS=Homo sapiens GN=PICALM - [PICAL_HUMAN] | 1.143 |
| Q10472 | *GALNT1* | Polypeptide N-acetylgalactosaminyltransferase 1 OS=Homo sapiens GN=GALNT1 PE=1 SV=1 - [GALT1_HUMAN] | 1.171 |
| P61106 | *RAB14* | Ras-related protein Rab-14 OS=Homo sapiens GN=RAB14 PE=1 SV=4 - [RAB14_HUMAN] | 1.174 |
| Q96GQ7 | *DDX27* | Probable ATP-dependent RNA helicase DDX27 OS=Homo sapiens GN=DDX27 PE=1 SV=2 - [DDX27_HUMAN] | 1.182 |
| Q96K76 | *USP47* | Isoform 2 of Ubiquitin carboxyl-terminal hydrolase 47 OS=Homo sapiens GN=USP47 - [UBP47_HUMAN] | 1.130 |
| O95154 | *AKR7A3* | Aflatoxin B1 aldehyde reductase member 3 OS=Homo sapiens GN=AKR7A3 PE=1 SV=2 - [ARK73_HUMAN] | 0.798 |
| Q15643 | *TRIP11* | Thyroid receptor-interacting protein 11 OS=Homo sapiens GN=TRIP11 PE=1 SV=3 - [TRIPB_HUMAN] | 1.092 |
| Q13554 | *CAMK2B* | Calcium/calmodulin-dependent protein kinase type II subunit beta OS=Homo sapiens GN=CAMK2B PE=1 SV=3 - [KCC2B_HUMAN] | 0.846 |
| P43155 | *CRAT* | Isoform 3 of Carnitine O-acetyltransferase OS=Homo sapiens GN=CRAT - [CACP_HUMAN] | 0.875 |
| P41180 | *CASR* | Extracellular calcium-sensing receptor OS=Homo sapiens GN=CASR PE=1 SV=2 - [CASR_HUMAN] | 0.490 |
| O15234 | *CASC3* | Protein CASC3 OS=Homo sapiens GN=CASC3 PE=1 SV=2 - [CASC3_HUMAN] | 1.081 |
| P01903 | *HLA-DRA* | HLA class II histocompatibility antigen, DR alpha chain OS=Homo sapiens GN=HLA-DRA PE=1 SV=1 - [DRA_HUMAN] | 0.589 |
| P08294 | *SOD3* | Extracellular superoxide dismutase [Cu-Zn] OS=Homo sapiens GN=SOD3 PE=1 SV=2 - [SODE_HUMAN] | 0.709 |
| Q9H2U2 | *PPA2* | Isoform 3 of Inorganic pyrophosphatase 2, mitochondrial OS=Homo sapiens GN=PPA2 - [IPYR2_HUMAN] | 0.792 |
| Q9UKK3 | *PARP4* | Poly [ADP-ribose] polymerase 4 OS=Homo sapiens GN=PARP4 PE=1 SV=3 - [PARP4_HUMAN] | 1.285 |
| Q92485 | *SMPDL3B* | Isoform 2 of Acid sphingomyelinase-like phosphodiesterase 3b OS=Homo sapiens GN=SMPDL3B - [ASM3B_HUMAN] | 0.670 |
| Q9BZL1 | *UBL5* | Ubiquitin-like protein 5 OS=Homo sapiens GN=UBL5 PE=1 SV=1 - [UBL5_HUMAN] | 0.882 |
| Q96EY7 | *PTCD3* | Pentatricopeptide repeat-containing protein 3, mitochondrial OS=Homo sapiens GN=PTCD3 PE=1 SV=3 - [PTCD3_HUMAN] | 1.075 |
| Q6NYC8 | *PPP1R18* | Phostensin OS=Homo sapiens GN=PPP1R18 PE=1 SV=1 - [PPR18_HUMAN] | 1.246 |
| P04062 | *GBA* | Isoform Short of Glucosylceramidase OS=Homo sapiens GN=GBA - [GLCM_HUMAN] | 1.196 |
| Q9Y3Z3 | *SAMHD1* | Isoform 2 of SAM domain and HD domain-containing protein 1 OS=Homo sapiens GN=SAMHD1 - [SAMH1_HUMAN] | 0.769 |
| Q9BW83 | *IFT27* | Isoform 2 of Intraflagellar transport protein 27 homolog OS=Homo sapiens GN=IFT27 - [IFT27_HUMAN] | 1.177 |
| Q6DD88 | *ATL3* | Atlastin-3 OS=Homo sapiens GN=ATL3 PE=1 SV=1 - [ATLA3_HUMAN] | 1.121 |
| Q96ME7 | *ZNF512* | Zinc finger protein 512 OS=Homo sapiens GN=ZNF512 PE=1 SV=2 - [ZN512_HUMAN] | 1.160 |
| Q96QR8 | *PURB* | Transcriptional activator protein Pur-beta OS=Homo sapiens GN=PURB PE=1 SV=3 - [PURB_HUMAN] | 1.110 |
| P00325 | *ADH1B* | Alcohol dehydrogenase 1B OS=Homo sapiens GN=ADH1B PE=1 SV=2 - [ADH1B_HUMAN] | 0.388 |
| Q8WZ42 | *TTN* | Isoform 5 of Titin OS=Homo sapiens GN=TTN - [TITIN_HUMAN] | 0.587 |
| P11310 | *ACADM* | Medium-chain specific acyl-CoA dehydrogenase, mitochondrial OS=Homo sapiens GN=ACADM PE=1 SV=1 - [ACADM_HUMAN] | 0.816 |
| Q08257 | *CRYZ* | Quinone oxidoreductase OS=Homo sapiens GN=CRYZ PE=1 SV=1 - [QOR_HUMAN] | 0.719 |
| Q92905 | *COPS5* | COP9 signalosome complex subunit 5 OS=Homo sapiens GN=COPS5 PE=1 SV=4 - [CSN5_HUMAN] | 0.905 |
| Q9UKD2 | *MRTO4* | mRNA turnover protein 4 homolog OS=Homo sapiens GN=MRTO4 PE=1 SV=2 - [MRT4_HUMAN] | 1.164 |
| Q9Y6N5 | *SQRDL* | Sulfide:quinone oxidoreductase, mitochondrial OS=Homo sapiens GN=SQRDL PE=1 SV=1 - [SQRD_HUMAN] | 0.557 |
| Q15019 | *SEPT2* | Septin-2 OS=Homo sapiens GN=SEPT2 PE=1 SV=1 - [SEPT2_HUMAN] | 0.898 |
| P29992 | *GNA11* | Guanine nucleotide-binding protein subunit alpha-11 OS=Homo sapiens GN=GNA11 PE=1 SV=2 - [GNA11_HUMAN] | 0.834 |
| Q9H1K0 | *RBSN* | Rabenosyn-5 OS=Homo sapiens GN=ZFYVE20 PE=1 SV=2 - [RBNS5_HUMAN] | 0.881 |
| P02788 | *LTF* | Lactotransferrin OS=Homo sapiens GN=LTF PE=1 SV=6 - [TRFL_HUMAN] | 0.375 |
| Q9BTY2 | *FUCA2* | Plasma alpha-L-fucosidase OS=Homo sapiens GN=FUCA2 PE=1 SV=2 - [FUCO2_HUMAN] | 0.690 |
| Q5T5C0 | *STXBP5* | Isoform 3 of Syntaxin-binding protein 5 OS=Homo sapiens GN=STXBP5 - [STXB5_HUMAN] | 1.356 |
| P17655 | *CAPN2* | Calpain-2 catalytic subunit OS=Homo sapiens GN=CAPN2 PE=1 SV=6 - [CAN2_HUMAN] | 1.252 |
| O15020 | *SPTBN2* | Isoform 2 of Spectrin beta chain, brain 2 OS=Homo sapiens GN=SPTBN2 - [SPTN2_HUMAN] | 1.206 |
| P61962 | *DCAF7* | DDB1- and CUL4-associated factor 7 OS=Homo sapiens GN=DCAF7 PE=1 SV=1 - [DCAF7_HUMAN] | 1.181 |
| Q96B23 | *C18orf25* | Isoform 2 of Uncharacterized protein C18orf25 OS=Homo sapiens GN=C18orf25 - [CR025_HUMAN] | 1.218 |
| Q96E29 | *MTERF3* | Isoform 2 of mTERF domain-containing protein 1, mitochondrial OS=Homo sapiens GN=MTERFD1 - [MTER1_HUMAN] | 1.166 |
| O94788 | *ALDH1A2* | Isoform 2 of Retinal dehydrogenase 2 OS=Homo sapiens GN=ALDH1A2 - [AL1A2_HUMAN] | 0.634 |
| Q9NUM4 | *TMEM106B* | Transmembrane protein 106B OS=Homo sapiens GN=TMEM106B PE=1 SV=2 - [T106B_HUMAN] | 0.926 |
| P00742 | *F10* | Coagulation factor X OS=Homo sapiens GN=F10 PE=1 SV=2 - [FA10_HUMAN] | 0.434 |
| O75063 | *FAM20B* | Glycosaminoglycan xylosylkinase OS=Homo sapiens GN=FAM20B PE=1 SV=1 - [XYLK_HUMAN] | 1.189 |
| Q9P0B6 | *CCDC167* | Coiled-coil domain-containing protein 167 OS=Homo sapiens GN=CCDC167 PE=2 SV=2 - [CC167_HUMAN] | 1.444 |
| P61006 | *RAB8A* | Ras-related protein Rab-8A OS=Homo sapiens GN=RAB8A PE=1 SV=1 - [RAB8A_HUMAN] | 0.900 |
| P36021 | *SLC16A2* | Monocarboxylate transporter 8 OS=Homo sapiens GN=SLC16A2 PE=1 SV=2 - [MOT8_HUMAN] | 0.846 |
| P13796 | *LCP1* | Plastin-2 OS=Homo sapiens GN=LCP1 PE=1 SV=6 - [PLSL_HUMAN] | 0.757 |
| Q14728 | *MFSD10* | Major facilitator superfamily domain-containing protein 10 OS=Homo sapiens GN=MFSD10 PE=2 SV=1 - [MFS10_HUMAN] | 1.193 |
| P01009 | *SERPINA1* | Alpha-1-antitrypsin OS=Homo sapiens GN=SERPINA1 PE=1 SV=3 - [A1AT_HUMAN] | 0.689 |
| Q00610 | *CLTC* | Isoform 2 of Clathrin heavy chain 1 OS=Homo sapiens GN=CLTC - [CLH1_HUMAN] | 1.071 |
| Q9ULK4 | *MED23* | Isoform 2 of Mediator of RNA polymerase II transcription subunit 23 OS=Homo sapiens GN=MED23 - [MED23_HUMAN] | 1.107 |
| O43169 | *CYB5B* | Cytochrome b5 type B OS=Homo sapiens GN=CYB5B PE=1 SV=2 - [CYB5B_HUMAN] | 1.384 |
| O00401 | *WASL* | Neural Wiskott-Aldrich syndrome protein OS=Homo sapiens GN=WASL PE=1 SV=2 - [WASL_HUMAN] | 1.232 |
| Q96N66 | *MBOAT7* | Isoform 3 of Lysophospholipid acyltransferase 7 OS=Homo sapiens GN=MBOAT7 - [MBOA7_HUMAN] | 1.153 |
| Q9ULL5 | *PRR12* | Isoform 2 of Proline-rich protein 12 OS=Homo sapiens GN=PRR12 - [PRR12_HUMAN] | 1.182 |
| O00567 | *NOP56* | Nucleolar protein 56 OS=Homo sapiens GN=NOP56 PE=1 SV=4 - [NOP56_HUMAN] | 1.118 |
| P27338 | *MAOB* | Amine oxidase [flavin-containing] B OS=Homo sapiens GN=MAOB PE=1 SV=3 - [AOFB_HUMAN] | 0.645 |
| Q01844 | *EWSR1* | RNA-binding protein EWS OS=Homo sapiens GN=EWSR1 PE=1 SV=1 - [EWS_HUMAN] | 0.875 |
| Q6ZWT7 | *MBOAT2* | Lysophospholipid acyltransferase 2 OS=Homo sapiens GN=MBOAT2 PE=2 SV=2 - [MBOA2_HUMAN] | 1.268 |
| Q5VWZ2 | *LYPLAL1* | Isoform 2 of Lysophospholipase-like protein 1 OS=Homo sapiens GN=LYPLAL1 - [LYPL1_HUMAN] | 0.851 |
| Q92947 | *GCDH* | Isoform Short of Glutaryl-CoA dehydrogenase, mitochondrial OS=Homo sapiens GN=GCDH - [GCDH_HUMAN] | 0.802 |
| P35612 | *ADD2* | Isoform 2 of Beta-adducin OS=Homo sapiens GN=ADD2 - [ADDB_HUMAN] | 1.540 |
| P14324 | *FDPS* | Farnesyl pyrophosphate synthase OS=Homo sapiens GN=FDPS PE=1 SV=4 - [FPPS_HUMAN] | 1.148 |
| Q8NCA5 | *FAM98A* | Isoform 2 of Protein FAM98A OS=Homo sapiens GN=FAM98A - [FA98A_HUMAN] | 0.914 |
| P26599 | *PTBP1* | Polypyrimidine tract-binding protein 1 OS=Homo sapiens GN=PTBP1 PE=1 SV=1 - [PTBP1_HUMAN] | 1.079 |
| P37268 | *FDFT1* | Squalene synthase OS=Homo sapiens GN=FDFT1 PE=1 SV=1 - [FDFT_HUMAN] | 1.444 |
| P98095 | *FBLN2* | Fibulin-2 OS=Homo sapiens GN=FBLN2 PE=1 SV=2 - [FBLN2_HUMAN] | 1.534 |
| Q6GMV3 | *PTRHD1* | Putative peptidyl-tRNA hydrolase PTRHD1 OS=Homo sapiens GN=PTRHD1 PE=1 SV=1 - [PTRD1_HUMAN] | 1.208 |
| P02671 | *FGA* | Isoform 2 of Fibrinogen alpha chain OS=Homo sapiens GN=FGA - [FIBA_HUMAN] | 0.714 |
| Q9C040 | *TRIM2* | Tripartite motif-containing protein 2 OS=Homo sapiens GN=TRIM2 PE=1 SV=1 - [TRIM2_HUMAN] | 0.862 |
| Q8IXK0 | *PHC2* | Isoform 2 of Polyhomeotic-like protein 2 OS=Homo sapiens GN=PHC2 - [PHC2_HUMAN] | 1.175 |
| Q8TDZ2 | *MICAL1* | Isoform 2 of NEDD9-interacting protein with calponin homology and LIM domains OS=Homo sapiens GN=MICAL1 - [MICA1_HUMAN] | 1.136 |
| Q8IUD2 | *ERC1* | Isoform 4 of ELKS/Rab6-interacting/CAST family member 1 OS=Homo sapiens GN=ERC1 - [RB6I2_HUMAN] | 0.916 |
| P02760 | *AMBP* | Protein AMBP OS=Homo sapiens GN=AMBP PE=1 SV=1 - [AMBP_HUMAN] | 0.650 |
| P30419 | *NMT1* | Glycylpeptide N-tetradecanoyltransferase 1 OS=Homo sapiens GN=NMT1 PE=1 SV=2 - [NMT1_HUMAN] | 1.129 |
| Q9UQE7 | *SMC3* | Structural maintenance of chromosomes protein 3 OS=Homo sapiens GN=SMC3 PE=1 SV=2 - [SMC3_HUMAN] | 0.918 |
| Q14697 | *GANAB* | Neutral alpha-glucosidase AB OS=Homo sapiens GN=GANAB PE=1 SV=3 - [GANAB_HUMAN] | 1.258 |
| Q9UL54 | *TAOK2* | Isoform 2 of Serine/threonine-protein kinase TAO2 OS=Homo sapiens GN=TAOK2 - [TAOK2_HUMAN] | 1.128 |
| Q13595 | *TRA2A* | Transformer-2 protein homolog alpha OS=Homo sapiens GN=TRA2A PE=1 SV=1 - [TRA2A_HUMAN] | 1.125 |
| Q8IY17 | *PNPLA6* | Isoform 2 of Neuropathy target esterase OS=Homo sapiens GN=PNPLA6 - [PLPL6_HUMAN] | 1.132 |
| Q9BVG4 | *PBDC1* | UPF0368 protein Cxorf26 OS=Homo sapiens GN=CXorf26 PE=1 SV=1 - [CX026_HUMAN] | 1.390 |
| Q8N1W1 | *ARHGEF28* | Isoform 4 of Rho-guanine nucleotide exchange factor OS=Homo sapiens GN=RGNEF - [RGNEF_HUMAN] | 1.123 |
| P17706 | *PTPN2* | Isoform PTPA of Tyrosine-protein phosphatase non-receptor type 2 OS=Homo sapiens GN=PTPN2 - [PTN2_HUMAN] | 1.330 |
| P13073 | *COX4I1* | Cytochrome c oxidase subunit 4 isoform 1, mitochondrial OS=Homo sapiens GN=COX4I1 PE=1 SV=1 - [COX41_HUMAN] | 0.562 |
| Q8IV48 | *ERI1* | 3'-5' exoribonuclease 1 OS=Homo sapiens GN=ERI1 PE=1 SV=3 - [ERI1_HUMAN] | 1.126 |
| O15511 | *ARPC5* | Actin-related protein 2/3 complex subunit 5 OS=Homo sapiens GN=ARPC5 PE=1 SV=3 - [ARPC5_HUMAN] | 0.872 |
| P35270 | *SPR* | Sepiapterin reductase OS=Homo sapiens GN=SPR PE=1 SV=1 - [SPRE_HUMAN] | 0.743 |
| P29350 | *PTPN6* | Isoform 3 of Tyrosine-protein phosphatase non-receptor type 6 OS=Homo sapiens GN=PTPN6 - [PTN6_HUMAN] | 0.924 |
| Q15413 | *RYR3* | Isoform 3 of Ryanodine receptor 3 OS=Homo sapiens GN=RYR3 - [RYR3_HUMAN] | 0.720 |
| P48553 | *TRAPPC10* | Trafficking protein particle complex subunit 10 OS=Homo sapiens GN=TRAPPC10 PE=1 SV=2 - [TPC10_HUMAN] | 1.114 |
| P58107 | *#N/A* | Epiplakin OS=Homo sapiens GN=EPPK1 PE=1 SV=2 - [EPIPL_HUMAN] | 1.690 |
| Q16795 | *NDUFA9* | NADH dehydrogenase [ubiquinone] 1 alpha subcomplex subunit 9, mitochondrial OS=Homo sapiens GN=NDUFA9 PE=1 SV=2 - [NDUA9_HUMAN] | 0.894 |
| P62244 | *RPS15A* | 40S ribosomal protein S15a OS=Homo sapiens GN=RPS15A PE=1 SV=2 - [RS15A_HUMAN] | 0.924 |
| Q9UNH7 | *SNX6* | Sorting nexin-6 OS=Homo sapiens GN=SNX6 PE=1 SV=1 - [SNX6_HUMAN] | 1.107 |
| Q9NPA0 | *EMC7* | UPF0480 protein C15orf24 OS=Homo sapiens GN=C15orf24 PE=1 SV=1 - [CO024_HUMAN] | 0.807 |
| Q96QK1 | *VPS35* | Vacuolar protein sorting-associated protein 35 OS=Homo sapiens GN=VPS35 PE=1 SV=2 - [VPS35_HUMAN] | 1.155 |
| Q9HA64 | *FN3KRP* | Ketosamine-3-kinase OS=Homo sapiens GN=FN3KRP PE=1 SV=2 - [KT3K_HUMAN] | 0.900 |
| O76031 | *CLPX* | ATP-dependent Clp protease ATP-binding subunit clpX-like, mitochondrial OS=Homo sapiens GN=CLPX PE=1 SV=2 - [CLPX_HUMAN] | 1.075 |
| Q01105 | *SET* | Isoform 2 of Protein SET OS=Homo sapiens GN=SET - [SET_HUMAN] | 1.098 |
| Q8WUA2 | *PPIL4* | Peptidyl-prolyl cis-trans isomerase-like 4 OS=Homo sapiens GN=PPIL4 PE=1 SV=1 - [PPIL4_HUMAN] | 0.921 |
| Q92538 | *GBF1* | Golgi-specific brefeldin A-resistance guanine nucleotide exchange factor 1 OS=Homo sapiens GN=GBF1 PE=1 SV=2 - [GBF1_HUMAN] | 1.157 |
| Q03252 | *#N/A* | Lamin-B2 OS=Homo sapiens GN=LMNB2 PE=1 SV=3 - [LMNB2_HUMAN] | 0.848 |
| P25398 | *RPS12* | 40S ribosomal protein S12 OS=Homo sapiens GN=RPS12 PE=1 SV=3 - [RS12_HUMAN] | 1.087 |
| Q8TCC3 | *MRPL30* | Isoform 3 of 39S ribosomal protein L30, mitochondrial OS=Homo sapiens GN=MRPL30 - [RM30_HUMAN] | 0.937 |
| P18859 | *ATP5J* | ATP synthase-coupling factor 6, mitochondrial OS=Homo sapiens GN=ATP5J PE=1 SV=1 - [ATP5J_HUMAN] | 0.886 |
| Q92599 | *SEPT8* | Isoform 2 of Septin-8 OS=Homo sapiens GN=SEPT8 - [SEPT8_HUMAN] | 0.798 |
| Q9BV79 | *MECR* | Isoform 2 of Trans-2-enoyl-CoA reductase, mitochondrial OS=Homo sapiens GN=MECR - [MECR_HUMAN] | 1.161 |
| P05164 | *MPO* | Isoform H14 of Myeloperoxidase OS=Homo sapiens GN=MPO - [PERM_HUMAN] | 0.233 |
| Q70UQ0 | *IKBIP* | Isoform 4 of Inhibitor of nuclear factor kappa-B kinase-interacting protein OS=Homo sapiens GN=IKBIP - [IKIP_HUMAN] | 1.219 |
| Q99836 | *MYD88* | Myeloid differentiation primary response protein MyD88 OS=Homo sapiens GN=MYD88 PE=1 SV=1 - [MYD88_HUMAN] | 1.170 |
| Q00577 | *PURA* | Transcriptional activator protein Pur-alpha OS=Homo sapiens GN=PURA PE=1 SV=2 - [PURA_HUMAN] | 0.869 |
| Q8IYS2 | *KIAA2013* | Uncharacterized protein KIAA2013 OS=Homo sapiens GN=KIAA2013 PE=2 SV=1 - [K2013_HUMAN] | 1.156 |
| P04844 | *RPN2* | Dolichyl-diphosphooligosaccharide--protein glycosyltransferase subunit 2 OS=Homo sapiens GN=RPN2 PE=1 SV=3 - [RPN2_HUMAN] | 0.944 |
| Q9Y4L1 | *HYOU1* | Hypoxia up-regulated protein 1 OS=Homo sapiens GN=HYOU1 PE=1 SV=1 - [HYOU1_HUMAN] | 1.163 |
| Q9H0Q0 | *FAM49A* | Protein FAM49A OS=Homo sapiens GN=FAM49A PE=1 SV=1 - [FA49A_HUMAN] | 1.365 |
| Q7L2H7 | *EIF3M* | Eukaryotic translation initiation factor 3 subunit M OS=Homo sapiens GN=EIF3M PE=1 SV=1 - [EIF3M_HUMAN] | 0.902 |
| P06733 | *ENO1* | Alpha-enolase OS=Homo sapiens GN=ENO1 PE=1 SV=2 - [ENOA_HUMAN] | 1.300 |
| Q92823 | *NRCAM* | Isoform 3 of Neuronal cell adhesion molecule OS=Homo sapiens GN=NRCAM - [NRCAM_HUMAN] | 1.284 |
| P16070 | *CD44* | Isoform 12 of CD44 antigen OS=Homo sapiens GN=CD44 - [CD44_HUMAN] | 0.649 |
| O00429 | *DNM1L* | Isoform 3 of Dynamin-1-like protein OS=Homo sapiens GN=DNM1L - [DNM1L_HUMAN] | 1.095 |
| P51858 | *HDGF* | Hepatoma-derived growth factor OS=Homo sapiens GN=HDGF PE=1 SV=1 - [HDGF_HUMAN] | 1.089 |
| Q5TDH0 | *DDI2* | Protein DDI1 homolog 2 OS=Homo sapiens GN=DDI2 PE=1 SV=1 - [DDI2_HUMAN] | 1.356 |
| Q99653 | *CHP1* | Calcium-binding protein p22 OS=Homo sapiens GN=CHP PE=1 SV=3 - [CHP1_HUMAN] | 0.910 |
| Q92934 | *BAD* | Bcl2 antagonist of cell death OS=Homo sapiens GN=BAD PE=1 SV=3 - [BAD_HUMAN] | 1.220 |
| Q2YD98 | *UVSSA* | Isoform 2 of Uncharacterized protein KIAA1530 OS=Homo sapiens GN=KIAA1530 - [K1530_HUMAN] | 1.142 |
| Q01780 | *EXOSC10* | Isoform 2 of Exosome component 10 OS=Homo sapiens GN=EXOSC10 - [EXOSX_HUMAN] | 1.186 |
| Q9UFN0 | *NIPSNAP3A* | Protein NipSnap homolog 3A OS=Homo sapiens GN=NIPSNAP3A PE=1 SV=2 - [NPS3A_HUMAN] | 0.857 |
| P35251 | *RFC1* | Isoform 2 of Replication factor C subunit 1 OS=Homo sapiens GN=RFC1 - [RFC1_HUMAN] | 0.911 |
| P13611 | *VCAN* | Isoform Vint of Versican core protein OS=Homo sapiens GN=VCAN - [CSPG2_HUMAN] | 5.097 |
| P42566 | *EPS15* | Epidermal growth factor receptor substrate 15 OS=Homo sapiens GN=EPS15 PE=1 SV=2 - [EPS15_HUMAN] | 1.163 |
| Q13191 | *CBLB* | Isoform Truncated 2 of E3 ubiquitin-protein ligase CBL-B OS=Homo sapiens GN=CBLB - [CBLB_HUMAN] | 0.872 |
| Q676U5 | *ATG16L1* | Isoform 3 of Autophagy-related protein 16-1 OS=Homo sapiens GN=ATG16L1 - [A16L1_HUMAN] | 1.150 |
| P29972 | *AQP1* | Aquaporin-1 OS=Homo sapiens GN=AQP1 PE=1 SV=3 - [AQP1_HUMAN] | 0.612 |
| Q14624 | *ITIH4* | Isoform 2 of Inter-alpha-trypsin inhibitor heavy chain H4 OS=Homo sapiens GN=ITIH4 - [ITIH4_HUMAN] | 0.759 |
| Q08AE8 | *SPIRE1* | Isoform 2 of Protein spire homolog 1 OS=Homo sapiens GN=SPIRE1 - [SPIR1_HUMAN] | 0.837 |
| Q9UGP8 | *SEC63* | Translocation protein SEC63 homolog OS=Homo sapiens GN=SEC63 PE=1 SV=2 - [SEC63_HUMAN] | 1.218 |
| Q9UJU6 | *DBNL* | Drebrin-like protein OS=Homo sapiens GN=DBNL PE=1 SV=1 - [DBNL_HUMAN] | 1.072 |
| Q9NVH0 | *EXD2* | Exonuclease 3'-5' domain-containing protein 2 OS=Homo sapiens GN=EXD2 PE=1 SV=1 - [EXD2_HUMAN] | 1.174 |
| Q9HB07 | *C12orf10* | UPF0160 protein MYG1, mitochondrial OS=Homo sapiens GN=C12orf10 PE=1 SV=2 - [MYG1_HUMAN] | 1.168 |
| Q96EK9 | *KTI12* | Protein KTI12 homolog OS=Homo sapiens GN=KTI12 PE=1 SV=1 - [KTI12_HUMAN] | 1.136 |
| Q9H0R3 | *TMEM222* | Transmembrane protein 222 OS=Homo sapiens GN=TMEM222 PE=2 SV=2 - [TM222_HUMAN] | 0.705 |
| Q07157 | *TJP1* | Tight junction protein ZO-1 OS=Homo sapiens GN=TJP1 PE=1 SV=3 - [ZO1_HUMAN] | 0.885 |
| Q9ULV4 | *CORO1C* | Coronin-1C OS=Homo sapiens GN=CORO1C PE=1 SV=1 - [COR1C_HUMAN] | 1.194 |
| Q99832 | *CCT7* | T-complex protein 1 subunit eta OS=Homo sapiens GN=CCT7 PE=1 SV=2 - [TCPH_HUMAN] | 1.091 |
| Q16352 | *INA* | Alpha-internexin OS=Homo sapiens GN=INA PE=1 SV=2 - [AINX_HUMAN] | 1.541 |
| O75146 | *HIP1R* | Huntingtin-interacting protein 1-related protein OS=Homo sapiens GN=HIP1R PE=1 SV=2 - [HIP1R_HUMAN] | 1.360 |
| O14519 | *CDK2AP1* | Cyclin-dependent kinase 2-associated protein 1 OS=Homo sapiens GN=CDK2AP1 PE=1 SV=1 - [CDKA1_HUMAN] | 1.103 |
| P37235 | *HPCAL1* | Hippocalcin-like protein 1 OS=Homo sapiens GN=HPCAL1 PE=1 SV=3 - [HPCL1_HUMAN] | 1.195 |
| P41732 | *TSPAN7* | Tetraspanin-7 OS=Homo sapiens GN=TSPAN7 PE=1 SV=2 - [TSN7_HUMAN] | 0.799 |
| Q02338 | *BDH1* | D-beta-hydroxybutyrate dehydrogenase, mitochondrial OS=Homo sapiens GN=BDH1 PE=1 SV=3 - [BDH_HUMAN] | 0.794 |
| A0AVT1 | *UBA6* | Isoform 2 of Ubiquitin-like modifier-activating enzyme 6 OS=Homo sapiens GN=UBA6 - [UBA6_HUMAN] | 1.103 |
| O14933 | *UBE2L6* | Ubiquitin/ISG15-conjugating enzyme E2 L6 OS=Homo sapiens GN=UBE2L6 PE=1 SV=4 - [UB2L6_HUMAN] | 1.599 |
| Q92599 | *SEPT8* | Isoform 3 of Septin-8 OS=Homo sapiens GN=SEPT8 - [SEPT8_HUMAN] | 0.805 |
| Q96F86 | *EDC3* | Enhancer of mRNA-decapping protein 3 OS=Homo sapiens GN=EDC3 PE=1 SV=1 - [EDC3_HUMAN] | 0.803 |
| Q15311 | *RALBP1* | RalA-binding protein 1 OS=Homo sapiens GN=RALBP1 PE=1 SV=3 - [RBP1_HUMAN] | 0.850 |
| Q15223 | *PVRL1* | Poliovirus receptor-related protein 1 OS=Homo sapiens GN=PVRL1 PE=1 SV=3 - [PVRL1_HUMAN] | 1.382 |
| Q12931 | *TRAP1* | Heat shock protein 75 kDa, mitochondrial OS=Homo sapiens GN=TRAP1 PE=1 SV=3 - [TRAP1_HUMAN] | 1.243 |
| P31749 | *AKT1* | RAC-alpha serine/threonine-protein kinase OS=Homo sapiens GN=AKT1 PE=1 SV=2 - [AKT1_HUMAN] | 1.113 |
| Q04637 | *EIF4G1* | Isoform E of Eukaryotic translation initiation factor 4 gamma 1 OS=Homo sapiens GN=EIF4G1 - [IF4G1_HUMAN] | 0.717 |
| Q9H9C1 | *VIPAS39* | VPS33B-interacting protein OS=Homo sapiens GN=VIPAR PE=1 SV=1 - [VIPAR_HUMAN] | 1.150 |
| Q9Y2K2 | *#N/A* | Isoform 3 of Serine/threonine-protein kinase SIK3 OS=Homo sapiens GN=SIK3 - [SIK3_HUMAN] | 0.813 |
| Q5R372 | *RABGAP1L* | Isoform 7 of Rab GTPase-activating protein 1-like OS=Homo sapiens GN=RABGAP1L - [RBG1L_HUMAN] | 1.089 |
| P27797 | *CALR* | Calreticulin OS=Homo sapiens GN=CALR PE=1 SV=1 - [CALR_HUMAN] | 1.188 |
| P08779 | *KRT16* | Keratin, type I cytoskeletal 16 OS=Homo sapiens GN=KRT16 PE=1 SV=4 - [K1C16_HUMAN] | 1.990 |
| Q08380 | *LGALS3BP* | Galectin-3-binding protein OS=Homo sapiens GN=LGALS3BP PE=1 SV=1 - [LG3BP_HUMAN] | 1.409 |
| P29373 | *CRABP2* | Cellular retinoic acid-binding protein 2 OS=Homo sapiens GN=CRABP2 PE=1 SV=2 - [RABP2_HUMAN] | 2.272 |
| P20585 | *MSH3* | DNA mismatch repair protein Msh3 OS=Homo sapiens GN=MSH3 PE=1 SV=4 - [MSH3_HUMAN] | 1.219 |
| P13861 | *PRKAR2A* | cAMP-dependent protein kinase type II-alpha regulatory subunit OS=Homo sapiens GN=PRKAR2A PE=1 SV=2 - [KAP2_HUMAN] | 0.914 |
| Q9NW68 | *BSDC1* | Isoform 9 of BSD domain-containing protein 1 OS=Homo sapiens GN=BSDC1 - [BSDC1_HUMAN] | 1.172 |
| Q01518 | *CAP1* | Adenylyl cyclase-associated protein 1 OS=Homo sapiens GN=CAP1 PE=1 SV=5 - [CAP1_HUMAN] | 1.164 |
| Q9H173 | *SIL1* | Nucleotide exchange factor SIL1 OS=Homo sapiens GN=SIL1 PE=1 SV=1 - [SIL1_HUMAN] | 0.773 |
| Q92692 | *PVRL2* | Poliovirus receptor-related protein 2 OS=Homo sapiens GN=PVRL2 PE=1 SV=1 - [PVRL2_HUMAN] | 0.901 |
| P52907 | *CAPZA1* | F-actin-capping protein subunit alpha-1 OS=Homo sapiens GN=CAPZA1 PE=1 SV=3 - [CAZA1_HUMAN] | 1.145 |
| Q6H8Q1 | *ABLIM2* | Isoform 6 of Actin-binding LIM protein 2 OS=Homo sapiens GN=ABLIM2 - [ABLM2_HUMAN] | 0.807 |
| P67936 | *TPM4* | Isoform 2 of Tropomyosin alpha-4 chain OS=Homo sapiens GN=TPM4 - [TPM4_HUMAN] | 1.448 |
| Q9BQ16 | *SPOCK3* | Isoform 1 of Testican-3 OS=Homo sapiens GN=SPOCK3 - [TICN3_HUMAN] | 0.602 |
| Q15404 | *RSU1* | Ras suppressor protein 1 OS=Homo sapiens GN=RSU1 PE=1 SV=3 - [RSU1_HUMAN] | 0.821 |
| P27816 | *MAP4* | Isoform 6 of Microtubule-associated protein 4 OS=Homo sapiens GN=MAP4 - [MAP4_HUMAN] | 0.891 |
| P62333 | *PSMC6* | 26S protease regulatory subunit 10B OS=Homo sapiens GN=PSMC6 PE=1 SV=1 - [PRS10_HUMAN] | 1.067 |
| Q9NUU7 | *DDX19A* | ATP-dependent RNA helicase DDX19A OS=Homo sapiens GN=DDX19A PE=1 SV=1 - [DD19A_HUMAN] | 1.112 |
| Q15283 | *RASA2* | Ras GTPase-activating protein 2 OS=Homo sapiens GN=RASA2 PE=1 SV=2 - [RASA2_HUMAN] | 1.202 |
| Q9HC35 | *EML4* | Echinoderm microtubule-associated protein-like 4 OS=Homo sapiens GN=EML4 PE=1 SV=3 - [EMAL4_HUMAN] | 1.218 |
| P05408 | *SCG5* | Isoform 2 of Neuroendocrine protein 7B2 OS=Homo sapiens GN=SCG5 - [7B2_HUMAN] | 0.726 |
| Q92597 | *NDRG1* | Protein NDRG1 OS=Homo sapiens GN=NDRG1 PE=1 SV=1 - [NDRG1_HUMAN] | 1.232 |
| Q5VWJ9 | *SNX30* | Sorting nexin-30 OS=Homo sapiens GN=SNX30 PE=1 SV=1 - [SNX30_HUMAN] | 1.155 |
| P30048 | *PRDX3* | Thioredoxin-dependent peroxide reductase, mitochondrial OS=Homo sapiens GN=PRDX3 PE=1 SV=3 - [PRDX3_HUMAN] | 0.844 |
| P35244 | *RPA3* | Replication protein A 14 kDa subunit OS=Homo sapiens GN=RPA3 PE=1 SV=1 - [RFA3_HUMAN] | 0.888 |
| Q9NQ79 | *CRTAC1* | Isoform 3 of Cartilage acidic protein 1 OS=Homo sapiens GN=CRTAC1 - [CRAC1_HUMAN] | 0.755 |
| O15084 | *ANKRD28* | Serine/threonine-protein phosphatase 6 regulatory ankyrin repeat subunit A OS=Homo sapiens GN=ANKRD28 PE=1 SV=5 - [ANR28_HUMAN] | 0.893 |
| P04275 | *VWF* | von Willebrand factor OS=Homo sapiens GN=VWF PE=1 SV=4 - [VWF_HUMAN] | 0.574 |
| Q13361 | *MFAP5* | Microfibrillar-associated protein 5 OS=Homo sapiens GN=MFAP5 PE=1 SV=1 - [MFAP5_HUMAN] | 0.565 |
| Q9UIC8 | *LCMT1* | Isoform 3 of Leucine carboxyl methyltransferase 1 OS=Homo sapiens GN=LCMT1 - [LCMT1_HUMAN] | 1.217 |
| P60228 | *EIF3E* | Eukaryotic translation initiation factor 3 subunit E OS=Homo sapiens GN=EIF3E PE=1 SV=1 - [EIF3E_HUMAN] | 1.084 |
| Q9NUQ2 | *AGPAT5* | 1-acyl-sn-glycerol-3-phosphate acyltransferase epsilon OS=Homo sapiens GN=AGPAT5 PE=1 SV=3 - [PLCE_HUMAN] | 0.869 |
| P60953 | *CDC42* | Cell division control protein 42 homolog OS=Homo sapiens GN=CDC42 PE=1 SV=2 - [CDC42_HUMAN] | 0.911 |
| P39060 | *COL18A1* | Isoform 3 of Collagen alpha-1(XVIII) chain OS=Homo sapiens GN=COL18A1 - [COIA1_HUMAN] | 0.821 |
| Q9NYU2 | *UGGT1* | Isoform 2 of UDP-glucose:glycoprotein glucosyltransferase 1 OS=Homo sapiens GN=UGGT1 - [UGGG1_HUMAN] | 1.214 |
| Q9UHV9 | *PFDN2* | Prefoldin subunit 2 OS=Homo sapiens GN=PFDN2 PE=1 SV=1 - [PFD2_HUMAN] | 1.201 |
| Q9BRP8 | *WIBG* | Isoform 2 of Partner of Y14 and mago OS=Homo sapiens GN=WIBG - [WIBG_HUMAN] | 0.892 |
| P00751 | *CFB* | Complement factor B OS=Homo sapiens GN=CFB PE=1 SV=2 - [CFAB_HUMAN] | 0.801 |
| Q9NUQ8 | *ABCF3* | Isoform 2 of ATP-binding cassette sub-family F member 3 OS=Homo sapiens GN=ABCF3 - [ABCF3_HUMAN] | 0.900 |
| Q9UN37 | *VPS4A* | Vacuolar protein sorting-associated protein 4A OS=Homo sapiens GN=VPS4A PE=1 SV=1 - [VPS4A_HUMAN] | 1.203 |
| Q92598 | *HSPH1* | Isoform Beta of Heat shock protein 105 kDa OS=Homo sapiens GN=HSPH1 - [HS105_HUMAN] | 1.336 |
| P52565 | *ARHGDIA* | Rho GDP-dissociation inhibitor 1 OS=Homo sapiens GN=ARHGDIA PE=1 SV=3 - [GDIR1_HUMAN] | 0.924 |
| P17661 | *DES* | Desmin OS=Homo sapiens GN=DES PE=1 SV=3 - [DESM_HUMAN] | 0.378 |
| Q14677 | *CLINT1* | Clathrin interactor 1 OS=Homo sapiens GN=CLINT1 PE=1 SV=1 - [EPN4_HUMAN] | 1.186 |
| Q68D91 | *MBLAC2* | Metallo-beta-lactamase domain-containing protein 2 OS=Homo sapiens GN=MBLAC2 PE=1 SV=3 - [MBLC2_HUMAN] | 0.862 |
| P25705 | *ATP5A1* | ATP synthase subunit alpha, mitochondrial OS=Homo sapiens GN=ATP5A1 PE=1 SV=1 - [ATPA_HUMAN] | 0.868 |
| Q8TBM8 | *DNAJB14* | Isoform 2 of DnaJ homolog subfamily B member 14 OS=Homo sapiens GN=DNAJB14 - [DJB14_HUMAN] | 0.823 |
| P24158 | *PRTN3* | Myeloblastin OS=Homo sapiens GN=PRTN3 PE=1 SV=3 - [PRTN3_HUMAN] | 0.211 |
| Q9UBB4 | *ATXN10* | Ataxin-10 OS=Homo sapiens GN=ATXN10 PE=1 SV=1 - [ATX10_HUMAN] | 1.119 |
| O43837 | *IDH3B* | Isocitrate dehydrogenase [NAD] subunit beta, mitochondrial OS=Homo sapiens GN=IDH3B PE=1 SV=2 - [IDH3B_HUMAN] | 0.896 |
| Q96GS4 | *C17orf59* | Uncharacterized protein C17orf59 OS=Homo sapiens GN=C17orf59 PE=1 SV=2 - [CQ059_HUMAN] | 1.310 |
| O00116 | *AGPS* | Alkyldihydroxyacetonephosphate synthase, peroxisomal OS=Homo sapiens GN=AGPS PE=1 SV=1 - [ADAS_HUMAN] | 1.148 |
| P05388 | *RPLP0* | 60S acidic ribosomal protein P0 OS=Homo sapiens GN=RPLP0 PE=1 SV=1 - [RLA0_HUMAN] | 1.061 |
| P01603 | *#N/A* | Ig kappa chain V-I region Ka OS=Homo sapiens PE=1 SV=1 - [KV111_HUMAN] | 2.932 |
| Q9H1I8 | *ASCC2* | Activating signal cointegrator 1 complex subunit 2 OS=Homo sapiens GN=ASCC2 PE=1 SV=3 - [ASCC2_HUMAN] | 1.116 |
| Q9Y2S7 | *POLDIP2* | Polymerase delta-interacting protein 2 OS=Homo sapiens GN=POLDIP2 PE=1 SV=1 - [PDIP2_HUMAN] | 0.936 |
| Q96GG9 | *DCUN1D1* | DCN1-like protein 1 OS=Homo sapiens GN=DCUN1D1 PE=1 SV=1 - [DCNL1_HUMAN] | 0.928 |
| Q9GZU8 | *FAM192A* | Protein FAM192A OS=Homo sapiens GN=FAM192A PE=1 SV=1 - [F192A_HUMAN] | 1.173 |
| Q16706 | *MAN2A1* | Alpha-mannosidase 2 OS=Homo sapiens GN=MAN2A1 PE=1 SV=2 - [MA2A1_HUMAN] | 1.231 |
| P07108 | *DBI* | Acyl-CoA-binding protein OS=Homo sapiens GN=DBI PE=1 SV=2 - [ACBP_HUMAN] | 0.846 |
| P08670 | *VIM* | Vimentin OS=Homo sapiens GN=VIM PE=1 SV=4 - [VIME_HUMAN] | 0.742 |
| P01011 | *SERPINA3* | Alpha-1-antichymotrypsin OS=Homo sapiens GN=SERPINA3 PE=1 SV=2 - [AACT_HUMAN] | 0.772 |
| P21964 | *COMT* | Isoform Soluble of Catechol O-methyltransferase OS=Homo sapiens GN=COMT - [COMT_HUMAN] | 1.224 |
| P13667 | *PDIA4* | Protein disulfide-isomerase A4 OS=Homo sapiens GN=PDIA4 PE=1 SV=2 - [PDIA4_HUMAN] | 1.238 |
| P36551 | *CPOX* | Coproporphyrinogen-III oxidase, mitochondrial OS=Homo sapiens GN=CPOX PE=1 SV=3 - [HEM6_HUMAN] | 0.866 |
| P18124 | *RPL7* | 60S ribosomal protein L7 OS=Homo sapiens GN=RPL7 PE=1 SV=1 - [RL7_HUMAN] | 1.051 |
| Q9H3S4 | *TPK1* | Thiamin pyrophosphokinase 1 OS=Homo sapiens GN=TPK1 PE=1 SV=1 - [TPK1_HUMAN] | 0.703 |
| P01034 | *CST3* | Cystatin-C OS=Homo sapiens GN=CST3 PE=1 SV=1 - [CYTC_HUMAN] | 0.631 |
| Q9Y2H5 | *PLEKHA6* | Pleckstrin homology domain-containing family A member 6 OS=Homo sapiens GN=PLEKHA6 PE=1 SV=4 - [PKHA6_HUMAN] | 1.172 |
| Q53FA7 | *TP53I3* | Quinone oxidoreductase PIG3 OS=Homo sapiens GN=TP53I3 PE=1 SV=2 - [QORX_HUMAN] | 1.373 |
| Q12789 | *GTF3C1* | Isoform 2 of General transcription factor 3C polypeptide 1 OS=Homo sapiens GN=GTF3C1 - [TF3C1_HUMAN] | 1.088 |
| Q15424 | *SAFB* | Scaffold attachment factor B1 OS=Homo sapiens GN=SAFB PE=1 SV=4 - [SAFB1_HUMAN] | 1.118 |
| Q14197 | *ICT1* | Peptidyl-tRNA hydrolase ICT1, mitochondrial OS=Homo sapiens GN=ICT1 PE=1 SV=1 - [ICT1_HUMAN] | 0.921 |
| Q6Q788 | *APOA5* | Apolipoprotein A-V OS=Homo sapiens GN=APOA5 PE=1 SV=1 - [APOA5_HUMAN] | 0.456 |
| Q9UMS6 | *SYNPO2* | Synaptopodin-2 OS=Homo sapiens GN=SYNPO2 PE=1 SV=2 - [SYNP2_HUMAN] | 0.708 |
| Q71UM5 | *RPS27L* | 40S ribosomal protein S27-like OS=Homo sapiens GN=RPS27L PE=1 SV=3 - [RS27L_HUMAN] | 0.904 |
| Q9ULX6 | *AKAP8L* | A-kinase anchor protein 8-like OS=Homo sapiens GN=AKAP8L PE=1 SV=3 - [AKP8L_HUMAN] | 1.169 |
| Q96M96 | *FGD4* | FYVE, RhoGEF and PH domain-containing protein 4 OS=Homo sapiens GN=FGD4 PE=1 SV=2 - [FGD4_HUMAN] | 1.259 |
| P11274 | *BCR* | Breakpoint cluster region protein OS=Homo sapiens GN=BCR PE=1 SV=2 - [BCR_HUMAN] | 1.256 |
| O94905 | *ERLIN2* | Erlin-2 OS=Homo sapiens GN=ERLIN2 PE=1 SV=1 - [ERLN2_HUMAN] | 1.381 |
| O75947 | *ATP5H* | ATP synthase subunit d, mitochondrial OS=Homo sapiens GN=ATP5H PE=1 SV=3 - [ATP5H_HUMAN] | 0.885 |
| O43752 | *STX6* | Syntaxin-6 OS=Homo sapiens GN=STX6 PE=1 SV=1 - [STX6_HUMAN] | 1.116 |
| Q7L5Y1 | *ENOSF1* | Mitochondrial enolase superfamily member 1 OS=Homo sapiens GN=ENOSF1 PE=1 SV=1 - [ENOF1_HUMAN] | 1.314 |
| Q8N573 | *OXR1* | Oxidation resistance protein 1 OS=Homo sapiens GN=OXR1 PE=1 SV=2 - [OXR1_HUMAN] | 1.411 |
| P07949 | *RET* | Isoform 2 of Proto-oncogene tyrosine-protein kinase receptor Ret OS=Homo sapiens GN=RET - [RET_HUMAN] | 0.861 |
| P01019 | *AGT* | Angiotensinogen OS=Homo sapiens GN=AGT PE=1 SV=1 - [ANGT_HUMAN] | 0.804 |
| Q9Y2J2 | *EPB41L3* | Isoform B of Band 4.1-like protein 3 OS=Homo sapiens GN=EPB41L3 - [E41L3_HUMAN] | 0.806 |
| Q9H061 | *TMEM126A* | Transmembrane protein 126A OS=Homo sapiens GN=TMEM126A PE=1 SV=1 - [T126A_HUMAN] | 1.094 |
| Q96CW1 | *AP2M1* | Isoform 2 of AP-2 complex subunit mu OS=Homo sapiens GN=AP2M1 - [AP2M1_HUMAN] | 0.949 |
| Q9UMR2 | *DDX19B* | Isoform 2 of ATP-dependent RNA helicase DDX19B OS=Homo sapiens GN=DDX19B - [DD19B_HUMAN] | 1.146 |
| Q6A1A2 | *#N/A* | Putative 3-phosphoinositide-dependent protein kinase 2 OS=Homo sapiens GN=PDPK2 PE=5 SV=1 - [PDPK2_HUMAN] | 1.195 |
| Q8WWM7 | *ATXN2L* | Isoform 6 of Ataxin-2-like protein OS=Homo sapiens GN=ATXN2L - [ATX2L_HUMAN] | 1.126 |
| P31150 | *GDI1* | Rab GDP dissociation inhibitor alpha OS=Homo sapiens GN=GDI1 PE=1 SV=2 - [GDIA_HUMAN] | 1.077 |
| Q9UKS6 | *PACSIN3* | Protein kinase C and casein kinase substrate in neurons protein 3 OS=Homo sapiens GN=PACSIN3 PE=1 SV=2 - [PACN3_HUMAN] | 0.713 |
| Q9Y639 | *NPTN* | Isoform 1 of Neuroplastin OS=Homo sapiens GN=NPTN - [NPTN_HUMAN] | 0.908 |
| Q9NYL4 | *FKBP11* | Peptidyl-prolyl cis-trans isomerase FKBP11 OS=Homo sapiens GN=FKBP11 PE=1 SV=1 - [FKB11_HUMAN] | 0.714 |
| Q14508 | *WFDC2* | Isoform 2 of WAP four-disulfide core domain protein 2 OS=Homo sapiens GN=WFDC2 - [WFDC2_HUMAN] | 0.329 |
| Q9HBM6 | *TAF9B* | Transcription initiation factor TFIID subunit 9B OS=Homo sapiens GN=TAF9B PE=1 SV=1 - [TAF9B_HUMAN] | 1.091 |
| P20742 | *PZP* | Isoform 2 of Pregnancy zone protein OS=Homo sapiens GN=PZP - [PZP_HUMAN] | 0.589 |
| P02763 | *ORM1* | Alpha-1-acid glycoprotein 1 OS=Homo sapiens GN=ORM1 PE=1 SV=1 - [A1AG1_HUMAN] | 0.758 |
| Q12986 | *NFX1* | Isoform 2 of Transcriptional repressor NF-X1 OS=Homo sapiens GN=NFX1 - [NFX1_HUMAN] | 0.586 |
| Q9BRZ2 | *TRIM56* | E3 ubiquitin-protein ligase TRIM56 OS=Homo sapiens GN=TRIM56 PE=1 SV=3 - [TRI56_HUMAN] | 1.101 |
| P45954 | *ACADSB* | Short/branched chain specific acyl-CoA dehydrogenase, mitochondrial OS=Homo sapiens GN=ACADSB PE=1 SV=1 - [ACDSB_HUMAN] | 0.886 |
| Q15020 | *SART3* | Squamous cell carcinoma antigen recognized by T-cells 3 OS=Homo sapiens GN=SART3 PE=1 SV=1 - [SART3_HUMAN] | 0.912 |
| Q9UNW1 | *MINPP1* | Multiple inositol polyphosphate phosphatase 1 OS=Homo sapiens GN=MINPP1 PE=1 SV=1 - [MINP1_HUMAN] | 0.837 |
| Q14520 | *HABP2* | Hyaluronan-binding protein 2 OS=Homo sapiens GN=HABP2 PE=1 SV=1 - [HABP2_HUMAN] | 0.779 |
| P18084 | *ITGB5* | Integrin beta-5 OS=Homo sapiens GN=ITGB5 PE=1 SV=1 - [ITB5_HUMAN] | 0.814 |
| P55209 | *NAP1L1* | Nucleosome assembly protein 1-like 1 OS=Homo sapiens GN=NAP1L1 PE=1 SV=1 - [NP1L1_HUMAN] | 1.119 |
| P78417 | *GSTO1* | Glutathione S-transferase omega-1 OS=Homo sapiens GN=GSTO1 PE=1 SV=2 - [GSTO1_HUMAN] | 0.843 |
| P0CAP2 | *POLR2M* | Isoform 3 of DNA-directed RNA polymerase II subunit GRINL1A OS=Homo sapiens GN=POLR2M - [GRL1A_HUMAN] | 1.120 |
| Q06587 | *RING1* | Isoform 2 of E3 ubiquitin-protein ligase RING1 OS=Homo sapiens GN=RING1 - [RING1_HUMAN] | 1.120 |
| P08582 | *MFI2* | Melanotransferrin OS=Homo sapiens GN=MFI2 PE=1 SV=2 - [TRFM_HUMAN] | 0.707 |
| P39748 | *FEN1* | Flap endonuclease 1 OS=Homo sapiens GN=FEN1 PE=1 SV=1 - [FEN1_HUMAN] | 1.154 |
| P07451 | *CA3* | Carbonic anhydrase 3 OS=Homo sapiens GN=CA3 PE=1 SV=3 - [CAH3_HUMAN] | 0.288 |
| Q96G23 | *CERS2* | Ceramide synthase 2 OS=Homo sapiens GN=CERS2 PE=1 SV=1 - [CERS2_HUMAN] | 1.159 |
| Q8N511 | *TMEM199* | Transmembrane protein 199 OS=Homo sapiens GN=TMEM199 PE=1 SV=1 - [TM199_HUMAN] | 1.178 |
| Q9NYL9 | *TMOD3* | Tropomodulin-3 OS=Homo sapiens GN=TMOD3 PE=1 SV=1 - [TMOD3_HUMAN] | 1.174 |
| P55145 | *MANF* | Mesencephalic astrocyte-derived neurotrophic factor OS=Homo sapiens GN=MANF PE=1 SV=3 - [MANF_HUMAN] | 1.280 |
| P05109 | *S100A8* | Protein S100-A8 OS=Homo sapiens GN=S100A8 PE=1 SV=1 - [S10A8_HUMAN] | 0.306 |
| Q01518 | *CAP1* | Isoform 2 of Adenylyl cyclase-associated protein 1 OS=Homo sapiens GN=CAP1 - [CAP1_HUMAN] | 1.152 |
| Q93009 | *USP7* | Ubiquitin carboxyl-terminal hydrolase 7 OS=Homo sapiens GN=USP7 PE=1 SV=2 - [UBP7_HUMAN] | 1.148 |
| P06744 | *GPI* | Glucose-6-phosphate isomerase OS=Homo sapiens GN=GPI PE=1 SV=4 - [G6PI_HUMAN] | 1.170 |
| P55011 | *SLC12A2* | Isoform 2 of Solute carrier family 12 member 2 OS=Homo sapiens GN=SLC12A2 - [S12A2_HUMAN] | 0.856 |
| P38919 | *EIF4A3* | Eukaryotic initiation factor 4A-III OS=Homo sapiens GN=EIF4A3 PE=1 SV=4 - [IF4A3_HUMAN] | 1.087 |
| P05997 | *COL5A2* | Collagen alpha-2(V) chain OS=Homo sapiens GN=COL5A2 PE=1 SV=3 - [CO5A2_HUMAN] | 1.485 |
| Q9H936 | *SLC25A22* | Mitochondrial glutamate carrier 1 OS=Homo sapiens GN=SLC25A22 PE=1 SV=1 - [GHC1_HUMAN] | 1.300 |
| Q8NHG7 | *SVIP* | Small VCP/p97-interacting protein OS=Homo sapiens GN=SVIP PE=2 SV=1 - [SVIP_HUMAN] | 0.767 |
| Q9HCM4 | *EPB41L5* | Isoform 2 of Band 4.1-like protein 5 OS=Homo sapiens GN=EPB41L5 - [E41L5_HUMAN] | 1.303 |
| Q9Y265 | *RUVBL1* | RuvB-like 1 OS=Homo sapiens GN=RUVBL1 PE=1 SV=1 - [RUVB1_HUMAN] | 1.109 |
| Q12768 | *KIAA0196* | WASH complex subunit strumpellin OS=Homo sapiens GN=KIAA0196 PE=1 SV=1 - [STRUM_HUMAN] | 0.875 |
| O75323 | *GBAS* | Protein NipSnap homolog 2 OS=Homo sapiens GN=GBAS PE=1 SV=1 - [NIPS2_HUMAN] | 0.788 |
| Q08945 | *SSRP1* | FACT complex subunit SSRP1 OS=Homo sapiens GN=SSRP1 PE=1 SV=1 - [SSRP1_HUMAN] | 1.115 |
| Q5J8M3 | *EMC4* | Isoform 3 of Transmembrane protein 85 OS=Homo sapiens GN=TMEM85 - [TMM85_HUMAN] | 0.868 |
| Q92600 | *RQCD1* | Cell differentiation protein RCD1 homolog OS=Homo sapiens GN=RQCD1 PE=1 SV=1 - [RCD1_HUMAN] | 1.075 |
| P61201 | *COPS2* | COP9 signalosome complex subunit 2 OS=Homo sapiens GN=COPS2 PE=1 SV=1 - [CSN2_HUMAN] | 0.927 |
| Q9GZZ7 | *GFRA4* | Isoform GFRalpha4a of GDNF family receptor alpha-4 OS=Homo sapiens GN=GFRA4 - [GFRA4_HUMAN] | 0.705 |
| O60942 | *RNGTT* | Isoform 3 of mRNA-capping enzyme OS=Homo sapiens GN=RNGTT - [MCE1_HUMAN] | 1.099 |
| Q9H2P0 | *ADNP* | Activity-dependent neuroprotector homeobox protein OS=Homo sapiens GN=ADNP PE=1 SV=1 - [ADNP_HUMAN] | 1.090 |
| O00571 | *DDX3X* | ATP-dependent RNA helicase DDX3X OS=Homo sapiens GN=DDX3X PE=1 SV=3 - [DDX3X_HUMAN] | 1.096 |
| P07951 | *TPM2* | Tropomyosin beta chain OS=Homo sapiens GN=TPM2 PE=1 SV=1 - [TPM2_HUMAN] | 0.255 |
| Q9H7D0 | *DOCK5* | Dedicator of cytokinesis protein 5 OS=Homo sapiens GN=DOCK5 PE=1 SV=3 - [DOCK5_HUMAN] | 1.135 |
| Q9BYW2 | *SETD2* | Isoform 3 of Histone-lysine N-methyltransferase SETD2 OS=Homo sapiens GN=SETD2 - [SETD2_HUMAN] | 1.147 |
| Q14894 | *CRYM* | Thiomorpholine-carboxylate dehydrogenase OS=Homo sapiens GN=CRYM PE=1 SV=1 - [CRYM_HUMAN] | 7.228 |
| P26639 | *TARS* | Threonine--tRNA ligase, cytoplasmic OS=Homo sapiens GN=TARS PE=1 SV=3 - [SYTC_HUMAN] | 1.127 |
| P16435 | *POR* | NADPH--cytochrome P450 reductase OS=Homo sapiens GN=POR PE=1 SV=2 - [NCPR_HUMAN] | 0.914 |
| P11233 | *RALA* | Ras-related protein Ral-A OS=Homo sapiens GN=RALA PE=1 SV=1 - [RALA_HUMAN] | 1.155 |
| Q9UNF0 | *PACSIN2* | Isoform 2 of Protein kinase C and casein kinase substrate in neurons protein 2 OS=Homo sapiens GN=PACSIN2 - [PACN2_HUMAN] | 1.148 |
| Q96J02 | *ITCH* | Isoform 2 of E3 ubiquitin-protein ligase Itchy homolog OS=Homo sapiens GN=ITCH - [ITCH_HUMAN] | 1.117 |
| Q15365 | *PCBP1* | Poly(rC)-binding protein 1 OS=Homo sapiens GN=PCBP1 PE=1 SV=2 - [PCBP1_HUMAN] | 0.894 |
| O95299 | *NDUFA10* | NADH dehydrogenase [ubiquinone] 1 alpha subcomplex subunit 10, mitochondrial OS=Homo sapiens GN=NDUFA10 PE=1 SV=1 - [NDUAA_HUMAN] | 0.878 |
| P29622 | *SERPINA4* | Kallistatin OS=Homo sapiens GN=SERPINA4 PE=1 SV=3 - [KAIN_HUMAN] | 0.792 |
| Q96P70 | *IPO9* | Importin-9 OS=Homo sapiens GN=IPO9 PE=1 SV=3 - [IPO9_HUMAN] | 1.132 |
| O60814 | *HIST1H2BK* | Histone H2B type 1-K OS=Homo sapiens GN=HIST1H2BK PE=1 SV=3 - [H2B1K_HUMAN] | 0.803 |
| Q6IC98 | *GRAMD4* | GRAM domain-containing protein 4 OS=Homo sapiens GN=GRAMD4 PE=1 SV=1 - [GRAM4_HUMAN] | 1.208 |
| P36269 | *GGT5* | Isoform 2 of Gamma-glutamyltransferase 5 OS=Homo sapiens GN=GGT5 - [GGT5_HUMAN] | 0.682 |
| Q6EMK4 | *VASN* | Vasorin OS=Homo sapiens GN=VASN PE=1 SV=1 - [VASN_HUMAN] | 0.880 |
| Q9H1B7 | *IRF2BPL* | Interferon regulatory factor 2-binding protein-like OS=Homo sapiens GN=IRF2BPL PE=1 SV=1 - [I2BPL_HUMAN] | 1.112 |
| O43242 | *PSMD3* | 26S proteasome non-ATPase regulatory subunit 3 OS=Homo sapiens GN=PSMD3 PE=1 SV=2 - [PSMD3_HUMAN] | 1.077 |
| P02675 | *FGB* | Fibrinogen beta chain OS=Homo sapiens GN=FGB PE=1 SV=2 - [FIBB_HUMAN] | 0.630 |
| Q9BX79 | *STRA6* | Isoform 3 of Stimulated by retinoic acid gene 6 protein homolog OS=Homo sapiens GN=STRA6 - [STRA6_HUMAN] | 1.546 |
| Q969E4 | *TCEAL3* | Transcription elongation factor A protein-like 3 OS=Homo sapiens GN=TCEAL3 PE=1 SV=1 - [TCAL3_HUMAN] | 0.816 |
| Q3YEC7 | *RABL6* | Putative GTP-binding protein Parf OS=Homo sapiens GN=PARF PE=1 SV=2 - [PARF_HUMAN] | 1.152 |
| Q13724 | *MOGS* | Mannosyl-oligosaccharide glucosidase OS=Homo sapiens GN=MOGS PE=1 SV=5 - [MOGS_HUMAN] | 1.135 |
| Q9UBC2 | *EPS15L1* | Epidermal growth factor receptor substrate 15-like 1 OS=Homo sapiens GN=EPS15L1 PE=1 SV=1 - [EP15R_HUMAN] | 1.083 |
| P29372 | *MPG* | Isoform 2 of DNA-3-methyladenine glycosylase OS=Homo sapiens GN=MPG - [3MG_HUMAN] | 1.093 |
| Q9HC52 | *CBX8* | Chromobox protein homolog 8 OS=Homo sapiens GN=CBX8 PE=1 SV=3 - [CBX8_HUMAN] | 1.201 |
| Q6PFW1 | *PPIP5K1* | Isoform 6 of Inositol hexakisphosphate and diphosphoinositol-pentakisphosphate kinase 1 OS=Homo sapiens GN=PPIP5K1 - [VIP1_HUMAN] | 1.175 |
| P98170 | *XIAP* | Baculoviral IAP repeat-containing protein 4 OS=Homo sapiens GN=XIAP PE=1 SV=2 - [XIAP_HUMAN] | 0.828 |
| Q9ULC3 | *RAB23* | Ras-related protein Rab-23 OS=Homo sapiens GN=RAB23 PE=1 SV=1 - [RAB23_HUMAN] | 0.849 |
| P48556 | *PSMD8* | 26S proteasome non-ATPase regulatory subunit 8 OS=Homo sapiens GN=PSMD8 PE=1 SV=2 - [PSMD8_HUMAN] | 1.093 |
| Q8TCT9 | *HM13* | Isoform 5 of Minor histocompatibility antigen H13 OS=Homo sapiens GN=HM13 - [HM13_HUMAN] | 1.174 |
| P02656 | *APOC3* | Apolipoprotein C-III OS=Homo sapiens GN=APOC3 PE=1 SV=1 - [APOC3_HUMAN] | 0.684 |
| O95865 | *DDAH2* | N(G),N(G)-dimethylarginine dimethylaminohydrolase 2 OS=Homo sapiens GN=DDAH2 PE=1 SV=1 - [DDAH2_HUMAN] | 1.172 |
| Q14839 | *CHD4* | Chromodomain-helicase-DNA-binding protein 4 OS=Homo sapiens GN=CHD4 PE=1 SV=2 - [CHD4_HUMAN] | 1.109 |
| P30405 | *PPIF* | Peptidyl-prolyl cis-trans isomerase F, mitochondrial OS=Homo sapiens GN=PPIF PE=1 SV=1 - [PPIF_HUMAN] | 0.739 |
| Q9UQ80 | *PA2G4* | Proliferation-associated protein 2G4 OS=Homo sapiens GN=PA2G4 PE=1 SV=3 - [PA2G4_HUMAN] | 1.093 |
| Q03519 | *TAP2* | Isoform 2 of Antigen peptide transporter 2 OS=Homo sapiens GN=TAP2 - [TAP2_HUMAN] | 2.259 |
| P11908 | *PRPS2* | Ribose-phosphate pyrophosphokinase 2 OS=Homo sapiens GN=PRPS2 PE=1 SV=2 - [PRPS2_HUMAN] | 0.887 |
| Q9NP79 | *VTA1* | Vacuolar protein sorting-associated protein VTA1 homolog OS=Homo sapiens GN=VTA1 PE=1 SV=1 - [VTA1_HUMAN] | 1.141 |
| Q66K14 | *TBC1D9B* | Isoform 2 of TBC1 domain family member 9B OS=Homo sapiens GN=TBC1D9B - [TBC9B_HUMAN] | 1.083 |
| P51797 | *CLCN6* | Isoform D of Chloride transport protein 6 OS=Homo sapiens GN=CLCN6 - [CLCN6_HUMAN] | 1.300 |
| O43581 | *SYT7* | Synaptotagmin-7 OS=Homo sapiens GN=SYT7 PE=1 SV=3 - [SYT7_HUMAN] | 0.825 |
| P42166 | *TMPO* | Lamina-associated polypeptide 2, isoform alpha OS=Homo sapiens GN=TMPO PE=1 SV=2 - [LAP2A_HUMAN] | 1.188 |
| Q68CZ2 | *TNS3* | Isoform 2 of Tensin-3 OS=Homo sapiens GN=TNS3 - [TENS3_HUMAN] | 0.807 |
| P14866 | *HNRNPL* | Heterogeneous nuclear ribonucleoprotein L OS=Homo sapiens GN=HNRNPL PE=1 SV=2 - [HNRPL_HUMAN] | 1.084 |
| P01834 | *#N/A* | Ig kappa chain C region OS=Homo sapiens GN=IGKC PE=1 SV=1 - [IGKC_HUMAN] | 0.723 |
| Q00403 | *GTF2B* | Transcription initiation factor IIB OS=Homo sapiens GN=GTF2B PE=1 SV=1 - [TF2B_HUMAN] | 0.862 |
| Q6ZVM7 | *TOM1L2* | Isoform 2 of TOM1-like protein 2 OS=Homo sapiens GN=TOM1L2 - [TM1L2_HUMAN] | 0.860 |
| P27482 | *CALML3* | Calmodulin-like protein 3 OS=Homo sapiens GN=CALML3 PE=1 SV=2 - [CALL3_HUMAN] | 0.492 |
| P61970 | *NUTF2* | Nuclear transport factor 2 OS=Homo sapiens GN=NUTF2 PE=1 SV=1 - [NTF2_HUMAN] | 1.146 |
| P84101 | *SERF2* | Small EDRK-rich factor 2 OS=Homo sapiens GN=SERF2 PE=1 SV=1 - [SERF2_HUMAN] | 0.874 |
| Q8WZ74 | *CTTNBP2* | Cortactin-binding protein 2 OS=Homo sapiens GN=CTTNBP2 PE=1 SV=1 - [CTTB2_HUMAN] | 0.695 |
| P30260 | *CDC27* | Cell division cycle protein 27 homolog OS=Homo sapiens GN=CDC27 PE=1 SV=2 - [CDC27_HUMAN] | 0.899 |
| Q5RI15 | *COX20* | Protein FAM36A OS=Homo sapiens GN=FAM36A PE=1 SV=2 - [FA36A_HUMAN] | 0.893 |
| Q99523 | *SORT1* | Sortilin OS=Homo sapiens GN=SORT1 PE=1 SV=3 - [SORT_HUMAN] | 0.868 |
| P06753 | *TPM3* | Tropomyosin alpha-3 chain OS=Homo sapiens GN=TPM3 PE=1 SV=1 - [TPM3_HUMAN] | 0.520 |
| P83881 | *RPL36A* | 60S ribosomal protein L36a OS=Homo sapiens GN=RPL36A PE=1 SV=2 - [RL36A_HUMAN] | 1.104 |
| O75083 | *WDR1* | WD repeat-containing protein 1 OS=Homo sapiens GN=WDR1 PE=1 SV=4 - [WDR1_HUMAN] | 1.134 |
| Q9H7Z7 | *PTGES2* | Prostaglandin E synthase 2 OS=Homo sapiens GN=PTGES2 PE=1 SV=1 - [PGES2_HUMAN] | 0.925 |
| Q6DKK2 | *TTC19* | Tetratricopeptide repeat protein 19, mitochondrial OS=Homo sapiens GN=TTC19 PE=1 SV=4 - [TTC19_HUMAN] | 0.909 |
| Q96DE0 | *NUDT16* | U8 snoRNA-decapping enzyme OS=Homo sapiens GN=NUDT16 PE=1 SV=2 - [NUD16_HUMAN] | 0.820 |
| P78549 | *NTHL1* | Endonuclease III-like protein 1 OS=Homo sapiens GN=NTHL1 PE=1 SV=2 - [NTHL1_HUMAN] | 1.167 |
| Q01804 | *OTUD4* | Isoform 3 of OTU domain-containing protein 4 OS=Homo sapiens GN=OTUD4 - [OTUD4_HUMAN] | 1.183 |
| P08247 | *SYP* | Synaptophysin OS=Homo sapiens GN=SYP PE=1 SV=3 - [SYPH_HUMAN] | 0.869 |
| Q14192 | *FHL2* | Four and a half LIM domains protein 2 OS=Homo sapiens GN=FHL2 PE=1 SV=3 - [FHL2_HUMAN] | 1.353 |
| P28676 | *GCA* | Grancalcin OS=Homo sapiens GN=GCA PE=1 SV=2 - [GRAN_HUMAN] | 1.174 |
| Q7Z2K6 | *ERMP1* | Endoplasmic reticulum metallopeptidase 1 OS=Homo sapiens GN=ERMP1 PE=1 SV=2 - [ERMP1_HUMAN] | 1.445 |
| Q5VT25 | *CDC42BPA* | Isoform 4 of Serine/threonine-protein kinase MRCK alpha OS=Homo sapiens GN=CDC42BPA - [MRCKA_HUMAN] | 0.932 |
| Q9H0B6 | *KLC2* | Kinesin light chain 2 OS=Homo sapiens GN=KLC2 PE=1 SV=1 - [KLC2_HUMAN] | 1.177 |
| P22003 | *BMP5* | Bone morphogenetic protein 5 OS=Homo sapiens GN=BMP5 PE=2 SV=1 - [BMP5_HUMAN] | 0.601 |
| Q13033 | *STRN3* | Isoform Alpha of Striatin-3 OS=Homo sapiens GN=STRN3 - [STRN3_HUMAN] | 1.068 |
| Q15631 | *TSN* | Translin OS=Homo sapiens GN=TSN PE=1 SV=1 - [TSN_HUMAN] | 0.922 |
| P55795 | *HNRNPH2* | Heterogeneous nuclear ribonucleoprotein H2 OS=Homo sapiens GN=HNRNPH2 PE=1 SV=1 - [HNRH2_HUMAN] | 0.925 |
| Q12824 | *SMARCB1* | Isoform B of SWI/SNF-related matrix-associated actin-dependent regulator of chromatin subfamily B member 1 OS=Homo sapiens GN=SMARCB1 - [SNF5_HUMAN] | 1.136 |
| Q9UBX5 | *FBLN5* | Fibulin-5 OS=Homo sapiens GN=FBLN5 PE=1 SV=1 - [FBLN5_HUMAN] | 1.430 |
| P07355 | *ANXA2* | Annexin A2 OS=Homo sapiens GN=ANXA2 PE=1 SV=2 - [ANXA2_HUMAN] | 0.616 |
| P82663 | *MRPS25* | 28S ribosomal protein S25, mitochondrial OS=Homo sapiens GN=MRPS25 PE=1 SV=1 - [RT25_HUMAN] | 0.925 |
| Q9Y394 | *DHRS7* | Isoform 2 of Dehydrogenase/reductase SDR family member 7 OS=Homo sapiens GN=DHRS7 - [DHRS7_HUMAN] | 1.168 |
| P61018 | *RAB4B* | Ras-related protein Rab-4B OS=Homo sapiens GN=RAB4B PE=1 SV=1 - [RAB4B_HUMAN] | 1.245 |
| P51148 | *RAB5C* | Ras-related protein Rab-5C OS=Homo sapiens GN=RAB5C PE=1 SV=2 - [RAB5C_HUMAN] | 1.094 |
| Q6ZUM4 | *ARHGAP27* | Isoform 2 of Rho GTPase-activating protein 27 OS=Homo sapiens GN=ARHGAP27 - [RHG27_HUMAN] | 1.285 |
| O00399 | *DCTN6* | Dynactin subunit 6 OS=Homo sapiens GN=DCTN6 PE=1 SV=1 - [DCTN6_HUMAN] | 1.095 |
| Q9UIC8 | *LCMT1* | Leucine carboxyl methyltransferase 1 OS=Homo sapiens GN=LCMT1 PE=1 SV=2 - [LCMT1_HUMAN] | 1.155 |
| Q3ZCQ8 | *TIMM50* | Mitochondrial import inner membrane translocase subunit TIM50 OS=Homo sapiens GN=TIMM50 PE=1 SV=2 - [TIM50_HUMAN] | 1.109 |
| P17096 | *HMGA1* | Isoform HMG-Y of High mobility group protein HMG-I/HMG-Y OS=Homo sapiens GN=HMGA1 - [HMGA1_HUMAN] | 1.281 |
| Q03164 | *KMT2A* | Isoform 14P-18B of Histone-lysine N-methyltransferase MLL OS=Homo sapiens GN=MLL - [MLL1_HUMAN] | 1.267 |
| Q9UPU7 | *TBC1D2B* | Isoform 2 of TBC1 domain family member 2B OS=Homo sapiens GN=TBC1D2B - [TBD2B_HUMAN] | 1.129 |
| P62191 | *PSMC1* | 26S protease regulatory subunit 4 OS=Homo sapiens GN=PSMC1 PE=1 SV=1 - [PRS4_HUMAN] | 1.055 |
| O75340 | *PDCD6* | Programmed cell death protein 6 OS=Homo sapiens GN=PDCD6 PE=1 SV=1 - [PDCD6_HUMAN] | 1.131 |
| P62158 | *CALM1* | Calmodulin OS=Homo sapiens GN=CALM1 PE=1 SV=2 - [CALM_HUMAN] | 1.117 |
| Q9UKG1 | *APPL1* | DCC-interacting protein 13-alpha OS=Homo sapiens GN=APPL1 PE=1 SV=1 - [DP13A_HUMAN] | 0.903 |
| P00338 | *LDHA* | L-lactate dehydrogenase A chain OS=Homo sapiens GN=LDHA PE=1 SV=2 - [LDHA_HUMAN] | 1.204 |
| Q9UBT2 | *UBA2* | SUMO-activating enzyme subunit 2 OS=Homo sapiens GN=UBA2 PE=1 SV=2 - [SAE2_HUMAN] | 1.094 |
| Q9Y2V7 | *COG6* | Isoform 2 of Conserved oligomeric Golgi complex subunit 6 OS=Homo sapiens GN=COG6 - [COG6_HUMAN] | 1.119 |
| Q96ME1 | *FBXL18* | Isoform 3 of F-box/LRR-repeat protein 18 OS=Homo sapiens GN=FBXL18 - [FXL18_HUMAN] | 1.097 |
| Q6SZW1 | *SARM1* | Isoform 2 of Sterile alpha and TIR motif-containing protein 1 OS=Homo sapiens GN=SARM1 - [SARM1_HUMAN] | 1.126 |
| Q9UGJ1 | *TUBGCP4* | Isoform 2 of Gamma-tubulin complex component 4 OS=Homo sapiens GN=TUBGCP4 - [GCP4_HUMAN] | 1.067 |
| P49961 | *ENTPD1* | Isoform Placental II of Ectonucleoside triphosphate diphosphohydrolase 1 OS=Homo sapiens GN=ENTPD1 - [ENTP1_HUMAN] | 0.828 |
| P22033 | *MUT* | Methylmalonyl-CoA mutase, mitochondrial OS=Homo sapiens GN=MUT PE=1 SV=4 - [MUTA_HUMAN] | 0.869 |
| P33527 | *ABCC1* | Isoform 8 of Multidrug resistance-associated protein 1 OS=Homo sapiens GN=ABCC1 - [MRP1_HUMAN] | 0.684 |
| P01111 | *NRAS* | GTPase NRas OS=Homo sapiens GN=NRAS PE=1 SV=1 - [RASN_HUMAN] | 0.859 |
| Q07955 | *SRSF1* | Isoform ASF-3 of Serine/arginine-rich splicing factor 1 OS=Homo sapiens GN=SRSF1 - [SRSF1_HUMAN] | 1.101 |
| Q02809 | *PLOD1* | Procollagen-lysine,2-oxoglutarate 5-dioxygenase 1 OS=Homo sapiens GN=PLOD1 PE=1 SV=2 - [PLOD1_HUMAN] | 1.269 |
| P23284 | *PPIB* | Peptidyl-prolyl cis-trans isomerase B OS=Homo sapiens GN=PPIB PE=1 SV=2 - [PPIB_HUMAN] | 1.107 |
| P19883 | *FST* | Isoform 2 of Follistatin OS=Homo sapiens GN=FST - [FST_HUMAN] | 1.908 |
| P20645 | *M6PR* | Cation-dependent mannose-6-phosphate receptor OS=Homo sapiens GN=M6PR PE=1 SV=1 - [MPRD_HUMAN] | 1.175 |
| O14498 | *ISLR* | Immunoglobulin superfamily containing leucine-rich repeat protein OS=Homo sapiens GN=ISLR PE=1 SV=1 - [ISLR_HUMAN] | 0.451 |
| Q9UBK9 | *UXT* | Protein UXT OS=Homo sapiens GN=UXT PE=1 SV=1 - [UXT_HUMAN] | 1.109 |
| Q99497 | *PARK7* | Protein DJ-1 OS=Homo sapiens GN=PARK7 PE=1 SV=2 - [PARK7_HUMAN] | 0.735 |
| O95453 | *PARN* | Poly(A)-specific ribonuclease PARN OS=Homo sapiens GN=PARN PE=1 SV=1 - [PARN_HUMAN] | 1.235 |
| Q9UP83 | *COG5* | Isoform 3 of Conserved oligomeric Golgi complex subunit 5 OS=Homo sapiens GN=COG5 - [COG5_HUMAN] | 1.089 |
| Q9HCN8 | *SDF2L1* | Stromal cell-derived factor 2-like protein 1 OS=Homo sapiens GN=SDF2L1 PE=1 SV=2 - [SDF2L_HUMAN] | 1.159 |
| Q9Y3P9 | *RABGAP1* | Rab GTPase-activating protein 1 OS=Homo sapiens GN=RABGAP1 PE=1 SV=3 - [RBGP1_HUMAN] | 1.054 |
| O43809 | *NUDT21* | Cleavage and polyadenylation specificity factor subunit 5 OS=Homo sapiens GN=NUDT21 PE=1 SV=1 - [CPSF5_HUMAN] | 1.080 |
| Q9Y276 | *BCS1L* | Mitochondrial chaperone BCS1 OS=Homo sapiens GN=BCS1L PE=1 SV=1 - [BCS1_HUMAN] | 0.926 |
| Q9Y6X5 | *ENPP4* | Ectonucleotide pyrophosphatase/phosphodiesterase family member 4 OS=Homo sapiens GN=ENPP4 PE=1 SV=3 - [ENPP4_HUMAN] | 0.856 |
| Q07960 | *ARHGAP1* | Rho GTPase-activating protein 1 OS=Homo sapiens GN=ARHGAP1 PE=1 SV=1 - [RHG01_HUMAN] | 1.152 |
| Q8NHP8 | *PLBD2* | Putative phospholipase B-like 2 OS=Homo sapiens GN=PLBD2 PE=1 SV=2 - [PLBL2_HUMAN] | 1.206 |
| O14562 | *UBFD1* | Ubiquitin domain-containing protein UBFD1 OS=Homo sapiens GN=UBFD1 PE=1 SV=2 - [UBFD1_HUMAN] | 1.173 |
| Q9NZ08 | *ERAP1* | Endoplasmic reticulum aminopeptidase 1 OS=Homo sapiens GN=ERAP1 PE=1 SV=3 - [ERAP1_HUMAN] | 1.204 |
| P49589 | *CARS* | Cysteine--tRNA ligase, cytoplasmic OS=Homo sapiens GN=CARS PE=1 SV=3 - [SYCC_HUMAN] | 1.074 |
| Q15386 | *UBE3C* | Ubiquitin-protein ligase E3C OS=Homo sapiens GN=UBE3C PE=1 SV=3 - [UBE3C_HUMAN] | 1.103 |
| A0FGR8 | *ESYT2* | Isoform 2 of Extended synaptotagmin-2 OS=Homo sapiens GN=ESYT2 - [ESYT2_HUMAN] | 1.349 |
| P30049 | *ATP5D* | ATP synthase subunit delta, mitochondrial OS=Homo sapiens GN=ATP5D PE=1 SV=2 - [ATPD_HUMAN] | 0.886 |
| Q96H79 | *ZC3HAV1L* | Zinc finger CCCH-type antiviral protein 1-like OS=Homo sapiens GN=ZC3HAV1L PE=1 SV=2 - [ZCCHL_HUMAN] | 1.161 |
| A6NHQ2 | *FBLL1* | rRNA/tRNA 2'-O-methyltransferase fibrillarin-like protein 1 OS=Homo sapiens GN=FBLL1 PE=3 SV=1 - [FBLL1_HUMAN] | 0.858 |
| Q9C0C4 | *SEMA4C* | Semaphorin-4C OS=Homo sapiens GN=SEMA4C PE=1 SV=2 - [SEM4C_HUMAN] | 1.270 |
| P14618 | *PKM* | Pyruvate kinase isozymes M1/M2 OS=Homo sapiens GN=PKM2 PE=1 SV=4 - [KPYM_HUMAN] | 1.196 |
| Q14194 | *CRMP1* | Dihydropyrimidinase-related protein 1 OS=Homo sapiens GN=CRMP1 PE=1 SV=1 - [DPYL1_HUMAN] | 1.156 |
| P17844 | *DDX5* | Probable ATP-dependent RNA helicase DDX5 OS=Homo sapiens GN=DDX5 PE=1 SV=1 - [DDX5_HUMAN] | 1.087 |
| P02655 | *APOC4-APOC2* | Apolipoprotein C-II OS=Homo sapiens GN=APOC2 PE=1 SV=1 - [APOC2_HUMAN] | 0.719 |
| P51808 | *DYNLT3* | Dynein light chain Tctex-type 3 OS=Homo sapiens GN=DYNLT3 PE=1 SV=1 - [DYLT3_HUMAN] | 0.865 |
| P09871 | *C1S* | Complement C1s subcomponent OS=Homo sapiens GN=C1S PE=1 SV=1 - [C1S_HUMAN] | 0.763 |
| P62495 | *ETF1* | Eukaryotic peptide chain release factor subunit 1 OS=Homo sapiens GN=ETF1 PE=1 SV=3 - [ERF1_HUMAN] | 1.154 |
| P49720 | *PSMB3* | Proteasome subunit beta type-3 OS=Homo sapiens GN=PSMB3 PE=1 SV=2 - [PSB3_HUMAN] | 1.082 |
| Q9UIQ6 | *LNPEP* | Isoform 3 of Leucyl-cystinyl aminopeptidase OS=Homo sapiens GN=LNPEP - [LCAP_HUMAN] | 0.918 |
| Q9HC38 | *GLOD4* | Isoform 2 of Glyoxalase domain-containing protein 4 OS=Homo sapiens GN=GLOD4 - [GLOD4_HUMAN] | 0.919 |
| Q9HD42 | *CHMP1A* | Isoform 2 of Charged multivesicular body protein 1a OS=Homo sapiens GN=CHMP1A - [CHM1A_HUMAN] | 1.369 |
| P56545 | *CTBP2* | C-terminal-binding protein 2 OS=Homo sapiens GN=CTBP2 PE=1 SV=1 - [CTBP2_HUMAN] | 1.104 |
| Q9NXF1 | *TEX10* | Testis-expressed sequence 10 protein OS=Homo sapiens GN=TEX10 PE=1 SV=2 - [TEX10_HUMAN] | 1.109 |
| Q5VW32 | *BROX* | BRO1 domain-containing protein BROX OS=Homo sapiens GN=BROX PE=1 SV=1 - [BROX_HUMAN] | 1.140 |
| Q7Z478 | *DHX29* | ATP-dependent RNA helicase DHX29 OS=Homo sapiens GN=DHX29 PE=1 SV=2 - [DHX29_HUMAN] | 0.893 |
| Q92466 | *DDB2* | Isoform D2 of DNA damage-binding protein 2 OS=Homo sapiens GN=DDB2 - [DDB2_HUMAN] | 0.849 |
| Q15042 | *RAB3GAP1* | Rab3 GTPase-activating protein catalytic subunit OS=Homo sapiens GN=RAB3GAP1 PE=1 SV=3 - [RB3GP_HUMAN] | 1.136 |
| Q8IVT2 | *MISP* | Uncharacterized protein C19orf21 OS=Homo sapiens GN=C19orf21 PE=1 SV=1 - [CS021_HUMAN] | 0.755 |
| P07741 | *APRT* | Adenine phosphoribosyltransferase OS=Homo sapiens GN=APRT PE=1 SV=2 - [APT_HUMAN] | 1.358 |
| Q9BVP2 | *GNL3* | Isoform 2 of Guanine nucleotide-binding protein-like 3 OS=Homo sapiens GN=GNL3 - [GNL3_HUMAN] | 1.100 |
| Q86X55 | *CARM1* | Isoform 2 of Histone-arginine methyltransferase CARM1 OS=Homo sapiens GN=CARM1 - [CARM1_HUMAN] | 1.117 |
| P09467 | *FBP1* | Fructose-1,6-bisphosphatase 1 OS=Homo sapiens GN=FBP1 PE=1 SV=5 - [F16P1_HUMAN] | 0.802 |
| Q05682 | *CALD1* | Caldesmon OS=Homo sapiens GN=CALD1 PE=1 SV=3 - [CALD1_HUMAN] | 0.672 |
| Q99798 | *ACO2* | Aconitate hydratase, mitochondrial OS=Homo sapiens GN=ACO2 PE=1 SV=2 - [ACON_HUMAN] | 1.231 |
| Q7Z4H3 | *HDDC2* | Isoform 2 of HD domain-containing protein 2 OS=Homo sapiens GN=HDDC2 - [HDDC2_HUMAN] | 0.853 |
| P06731 | *CEACAM5* | Carcinoembryonic antigen-related cell adhesion molecule 5 OS=Homo sapiens GN=CEACAM5 PE=1 SV=3 - [CEAM5_HUMAN] | 2.005 |
| Q9UEE9 | *CFDP1* | Isoform 2 of Craniofacial development protein 1 OS=Homo sapiens GN=CFDP1 - [CFDP1_HUMAN] | 1.355 |
| Q99459 | *CDC5L* | Cell division cycle 5-like protein OS=Homo sapiens GN=CDC5L PE=1 SV=2 - [CDC5L_HUMAN] | 1.086 |
| P82970 | *HMGN5* | High mobility group nucleosome-binding domain-containing protein 5 OS=Homo sapiens GN=HMGN5 PE=1 SV=1 - [HMGN5_HUMAN] | 0.797 |
| P78344 | *EIF4G2* | Isoform 2 of Eukaryotic translation initiation factor 4 gamma 2 OS=Homo sapiens GN=EIF4G2 - [IF4G2_HUMAN] | 1.034 |
| P41218 | *MNDA* | Myeloid cell nuclear differentiation antigen OS=Homo sapiens GN=MNDA PE=1 SV=1 - [MNDA_HUMAN] | 0.649 |
| Q5VWQ0 | *RSBN1* | Round spermatid basic protein 1 OS=Homo sapiens GN=RSBN1 PE=2 SV=2 - [RSBN1_HUMAN] | 0.871 |
| Q9H444 | *CHMP4B* | Charged multivesicular body protein 4b OS=Homo sapiens GN=CHMP4B PE=1 SV=1 - [CHM4B_HUMAN] | 1.158 |
| Q86VB7 | *CD163* | Isoform 3 of Scavenger receptor cysteine-rich type 1 protein M130 OS=Homo sapiens GN=CD163 - [C163A_HUMAN] | 0.664 |
| Q86TM6 | *SYVN1* | Isoform 2 of E3 ubiquitin-protein ligase synoviolin OS=Homo sapiens GN=SYVN1 - [SYVN1_HUMAN] | 1.158 |
| Q92614 | *MYO18A* | Isoform 4 of Myosin-XVIIIa OS=Homo sapiens GN=MYO18A - [MY18A_HUMAN] | 1.184 |
| Q14318 | *FKBP8* | Peptidyl-prolyl cis-trans isomerase FKBP8 OS=Homo sapiens GN=FKBP8 PE=1 SV=2 - [FKBP8_HUMAN] | 0.901 |
| P49917 | *LIG4* | DNA ligase 4 OS=Homo sapiens GN=LIG4 PE=1 SV=2 - [DNLI4_HUMAN] | 0.922 |
| Q68DH5 | *LMBRD2* | LMBR1 domain-containing protein 2 OS=Homo sapiens GN=LMBRD2 PE=1 SV=1 - [LMBD2_HUMAN] | 1.182 |
| Q9NQY0 | *BIN3* | Isoform 2 of Bridging integrator 3 OS=Homo sapiens GN=BIN3 - [BIN3_HUMAN] | 1.229 |
| Q8NEU8 | *APPL2* | DCC-interacting protein 13-beta OS=Homo sapiens GN=APPL2 PE=1 SV=3 - [DP13B_HUMAN] | 1.181 |
| Q8N6M0 | *OTUD6B* | OTU domain-containing protein 6B OS=Homo sapiens GN=OTUD6B PE=1 SV=1 - [OTU6B_HUMAN] | 1.266 |
| P05121 | *SERPINE1* | Plasminogen activator inhibitor 1 OS=Homo sapiens GN=SERPINE1 PE=1 SV=1 - [PAI1_HUMAN] | 1.638 |
| Q8NCH0 | *CHST14* | Carbohydrate sulfotransferase 14 OS=Homo sapiens GN=CHST14 PE=1 SV=2 - [CHSTE_HUMAN] | 0.685 |
| Q9H1Z4 | *WDR13* | WD repeat-containing protein 13 OS=Homo sapiens GN=WDR13 PE=1 SV=2 - [WDR13_HUMAN] | 1.059 |
| Q9UBQ5 | *EIF3K* | Eukaryotic translation initiation factor 3 subunit K OS=Homo sapiens GN=EIF3K PE=1 SV=1 - [EIF3K_HUMAN] | 1.045 |
| Q16851 | *UGP2* | UTP--glucose-1-phosphate uridylyltransferase OS=Homo sapiens GN=UGP2 PE=1 SV=5 - [UGPA_HUMAN] | 1.095 |
| P51812 | *RPS6KA3* | Ribosomal protein S6 kinase alpha-3 OS=Homo sapiens GN=RPS6KA3 PE=1 SV=1 - [KS6A3_HUMAN] | 1.247 |
| P20073 | *ANXA7* | Isoform 2 of Annexin A7 OS=Homo sapiens GN=ANXA7 - [ANXA7_HUMAN] | 0.919 |
| Q6IA17 | *SIGIRR* | Single Ig IL-1-related receptor OS=Homo sapiens GN=SIGIRR PE=1 SV=3 - [SIGIR_HUMAN] | 1.184 |
| P18754 | *RCC1* | Regulator of chromosome condensation OS=Homo sapiens GN=RCC1 PE=1 SV=1 - [RCC1_HUMAN] | 1.144 |
| O94856 | *NFASC* | Isoform 4 of Neurofascin OS=Homo sapiens GN=NFASC - [NFASC_HUMAN] | 0.847 |
| Q15417 | *CNN3* | Calponin-3 OS=Homo sapiens GN=CNN3 PE=1 SV=1 - [CNN3_HUMAN] | 1.295 |
| Q13561 | *DCTN2* | Dynactin subunit 2 OS=Homo sapiens GN=DCTN2 PE=1 SV=4 - [DCTN2_HUMAN] | 1.115 |
| Q9NXG2 | *THUMPD1* | THUMP domain-containing protein 1 OS=Homo sapiens GN=THUMPD1 PE=1 SV=2 - [THUM1_HUMAN] | 1.128 |
| Q9UKB1 | *FBXW11* | Isoform A of F-box/WD repeat-containing protein 11 OS=Homo sapiens GN=FBXW11 - [FBW1B_HUMAN] | 0.890 |
| Q92752 | *TNR* | Isoform 2 of Tenascin-R OS=Homo sapiens GN=TNR - [TENR_HUMAN] | 0.601 |
| Q15149 | *PLEC* | Isoform 8 of Plectin OS=Homo sapiens GN=PLEC - [PLEC_HUMAN] | 0.854 |
| O00541 | *PES1* | Isoform 2 of Pescadillo homolog OS=Homo sapiens GN=PES1 - [PESC_HUMAN] | 1.122 |
| Q15477 | *SKIV2L* | Helicase SKI2W OS=Homo sapiens GN=SKIV2L PE=1 SV=3 - [SKIV2_HUMAN] | 0.903 |
| Q9Y2Q0 | *ATP8A1* | Isoform 2 of Probable phospholipid-transporting ATPase IA OS=Homo sapiens GN=ATP8A1 - [AT8A1_HUMAN] | 0.802 |
| Q09028 | *RBBP4* | Isoform 4 of Histone-binding protein RBBP4 OS=Homo sapiens GN=RBBP4 - [RBBP4_HUMAN] | 1.218 |
| O75396 | *SEC22B* | Vesicle-trafficking protein SEC22b OS=Homo sapiens GN=SEC22B PE=1 SV=4 - [SC22B_HUMAN] | 0.848 |
| Q9H8L6 | *MMRN2* | Multimerin-2 OS=Homo sapiens GN=MMRN2 PE=1 SV=2 - [MMRN2_HUMAN] | 0.767 |
| P43405 | *SYK* | Isoform Short of Tyrosine-protein kinase SYK OS=Homo sapiens GN=SYK - [KSYK_HUMAN] | 1.202 |
| P19823 | *ITIH2* | Inter-alpha-trypsin inhibitor heavy chain H2 OS=Homo sapiens GN=ITIH2 PE=1 SV=2 - [ITIH2_HUMAN] | 1.508 |
| P49711 | *CTCF* | Transcriptional repressor CTCF OS=Homo sapiens GN=CTCF PE=1 SV=1 - [CTCF_HUMAN] | 1.108 |
| P30040 | *ERP29* | Endoplasmic reticulum resident protein 29 OS=Homo sapiens GN=ERP29 PE=1 SV=4 - [ERP29_HUMAN] | 0.877 |
| O00330 | *PDHX* | Pyruvate dehydrogenase protein X component, mitochondrial OS=Homo sapiens GN=PDHX PE=1 SV=3 - [ODPX_HUMAN] | 1.085 |
| P08243 | *ASNS* | Asparagine synthetase [glutamine-hydrolyzing] OS=Homo sapiens GN=ASNS PE=1 SV=4 - [ASNS_HUMAN] | 1.379 |
| P62745 | *RHOB* | Rho-related GTP-binding protein RhoB OS=Homo sapiens GN=RHOB PE=1 SV=1 - [RHOB_HUMAN] | 0.861 |
| Q8WX93 | *PALLD* | Isoform 7 of Palladin OS=Homo sapiens GN=PALLD - [PALLD_HUMAN] | 1.227 |
| A1X283 | *SH3PXD2B* | SH3 and PX domain-containing protein 2B OS=Homo sapiens GN=SH3PXD2B PE=1 SV=3 - [SPD2B_HUMAN] | 0.860 |
| Q15006 | *EMC2* | Tetratricopeptide repeat protein 35 OS=Homo sapiens GN=TTC35 PE=1 SV=1 - [TTC35_HUMAN] | 0.912 |
| Q96D53 | *ADCK4* | Isoform 2 of Uncharacterized aarF domain-containing protein kinase 4 OS=Homo sapiens GN=ADCK4 - [ADCK4_HUMAN] | 1.125 |
| Q9Y520 | *PRRC2C* | Isoform 2 of Protein PRRC2C OS=Homo sapiens GN=PRRC2C - [PRC2C_HUMAN] | 1.120 |
| P55084 | *HADHB* | Trifunctional enzyme subunit beta, mitochondrial OS=Homo sapiens GN=HADHB PE=1 SV=3 - [ECHB_HUMAN] | 0.909 |
| P78371 | *CCT2* | T-complex protein 1 subunit beta OS=Homo sapiens GN=CCT2 PE=1 SV=4 - [TCPB_HUMAN] | 1.079 |
| Q53GT1 | *KLHL22* | Kelch-like protein 22 OS=Homo sapiens GN=KLHL22 PE=1 SV=2 - [KLH22_HUMAN] | 1.102 |
| O43765 | *SGTA* | Small glutamine-rich tetratricopeptide repeat-containing protein alpha OS=Homo sapiens GN=SGTA PE=1 SV=1 - [SGTA_HUMAN] | 1.133 |
| Q9P2R3 | *ANKFY1* | Ankyrin repeat and FYVE domain-containing protein 1 OS=Homo sapiens GN=ANKFY1 PE=1 SV=2 - [ANFY1_HUMAN] | 1.078 |
| P42167 | *TMPO* | Lamina-associated polypeptide 2, isoforms beta/gamma OS=Homo sapiens GN=TMPO PE=1 SV=2 - [LAP2B_HUMAN] | 1.107 |
| Q6L8Q7 | *PDE12* | Isoform 2 of 2',5'-phosphodiesterase 12 OS=Homo sapiens GN=PDE12 - [PDE12_HUMAN] | 0.918 |
| O94906 | *PRPF6* | Pre-mRNA-processing factor 6 OS=Homo sapiens GN=PRPF6 PE=1 SV=1 - [PRP6_HUMAN] | 1.078 |
| P09493 | *TPM1* | Isoform 6 of Tropomyosin alpha-1 chain OS=Homo sapiens GN=TPM1 - [TPM1_HUMAN] | 0.601 |
| Q8TB96 | *ITFG1* | T-cell immunomodulatory protein OS=Homo sapiens GN=ITFG1 PE=1 SV=1 - [TIP_HUMAN] | 0.819 |
| O00268 | *TAF4* | Transcription initiation factor TFIID subunit 4 OS=Homo sapiens GN=TAF4 PE=1 SV=2 - [TAF4_HUMAN] | 1.135 |
| Q99961 | *SH3GL1* | Endophilin-A2 OS=Homo sapiens GN=SH3GL1 PE=1 SV=1 - [SH3G1_HUMAN] | 1.158 |
| Q53GS7 | *GLE1* | Isoform 2 of Nucleoporin GLE1 OS=Homo sapiens GN=GLE1 - [GLE1_HUMAN] | 0.894 |
| Q9H814 | *PHAX* | Phosphorylated adapter RNA export protein OS=Homo sapiens GN=PHAX PE=1 SV=1 - [PHAX_HUMAN] | 0.906 |
| Q9Y243 | *AKT3* | Isoform 2 of RAC-gamma serine/threonine-protein kinase OS=Homo sapiens GN=AKT3 - [AKT3_HUMAN] | 0.853 |
| Q15046 | *KARS* | Lysine--tRNA ligase OS=Homo sapiens GN=KARS PE=1 SV=3 - [SYK_HUMAN] | 1.129 |
| Q8WUW1 | *BRK1* | Protein BRICK1 OS=Homo sapiens GN=BRK1 PE=1 SV=1 - [BRK1_HUMAN] | 0.937 |
| Q9NUL5 | *C19orf66* | Isoform 3 of UPF0515 protein C19orf66 OS=Homo sapiens GN=C19orf66 - [CS066_HUMAN] | 0.875 |
| P19022 | *CDH2* | Cadherin-2 OS=Homo sapiens GN=CDH2 PE=1 SV=4 - [CADH2_HUMAN] | 0.751 |
| P23142 | *FBLN1* | Fibulin-1 OS=Homo sapiens GN=FBLN1 PE=1 SV=4 - [FBLN1_HUMAN] | 1.288 |
| O75691 | *UTP20* | Small subunit processome component 20 homolog OS=Homo sapiens GN=UTP20 PE=1 SV=3 - [UTP20_HUMAN] | 1.094 |
| Q9Y2R0 | *COA3* | Coiled-coil domain-containing protein 56 OS=Homo sapiens GN=CCDC56 PE=1 SV=1 - [CCD56_HUMAN] | 0.856 |
| P17900 | *GM2A* | Ganglioside GM2 activator OS=Homo sapiens GN=GM2A PE=1 SV=4 - [SAP3_HUMAN] | 1.193 |
| P50583 | *NUDT2* | Bis(5'-nucleosyl)-tetraphosphatase [asymmetrical] OS=Homo sapiens GN=NUDT2 PE=1 SV=3 - [AP4A_HUMAN] | 1.178 |
| P35232 | *PHB* | Prohibitin OS=Homo sapiens GN=PHB PE=1 SV=1 - [PHB_HUMAN] | 0.890 |
| Q10589 | *BST2* | Bone marrow stromal antigen 2 OS=Homo sapiens GN=BST2 PE=1 SV=1 - [BST2_HUMAN] | 1.537 |
| O00165 | *HAX1* | Isoform 5 of HCLS1-associated protein X-1 OS=Homo sapiens GN=HAX1 - [HAX1_HUMAN] | 1.053 |
| Q96KN1 | *FAM84B* | Protein FAM84B OS=Homo sapiens GN=FAM84B PE=1 SV=1 - [FA84B_HUMAN] | 0.838 |
| P46109 | *CRKL* | Crk-like protein OS=Homo sapiens GN=CRKL PE=1 SV=1 - [CRKL_HUMAN] | 1.113 |
| P0CW22 | *0* | 40S ribosomal protein S17-like OS=Homo sapiens GN=RPS17L PE=3 SV=1 - [RS17L_HUMAN] | 1.121 |
| Q99829 | *CPNE1* | Copine-1 OS=Homo sapiens GN=CPNE1 PE=1 SV=1 - [CPNE1_HUMAN] | 1.156 |
| P05496 | *ATP5G1* | ATP synthase lipid-binding protein, mitochondrial OS=Homo sapiens GN=ATP5G1 PE=2 SV=2 - [AT5G1_HUMAN] | 0.880 |
| Q14515 | *SPARCL1* | SPARC-like protein 1 OS=Homo sapiens GN=SPARCL1 PE=1 SV=2 - [SPRL1_HUMAN] | 0.809 |
| P35749 | *MYH11* | Myosin-11 OS=Homo sapiens GN=MYH11 PE=1 SV=3 - [MYH11_HUMAN] | 0.636 |
| Q15650 | *TRIP4* | Activating signal cointegrator 1 OS=Homo sapiens GN=TRIP4 PE=1 SV=4 - [TRIP4_HUMAN] | 1.080 |
| Q9P013 | *CWC15* | Protein CWC15 homolog OS=Homo sapiens GN=CWC15 PE=1 SV=2 - [CWC15_HUMAN] | 1.096 |
| Q06830 | *PRDX1* | Peroxiredoxin-1 OS=Homo sapiens GN=PRDX1 PE=1 SV=1 - [PRDX1_HUMAN] | 0.808 |
| O95169 | *NDUFB8* | NADH dehydrogenase [ubiquinone] 1 beta subcomplex subunit 8, mitochondrial OS=Homo sapiens GN=NDUFB8 PE=1 SV=1 - [NDUB8_HUMAN] | 0.910 |
| P17987 | *TCP1* | T-complex protein 1 subunit alpha OS=Homo sapiens GN=TCP1 PE=1 SV=1 - [TCPA_HUMAN] | 1.073 |
| Q96FQ6 | *S100A16* | Protein S100-A16 OS=Homo sapiens GN=S100A16 PE=1 SV=1 - [S10AG_HUMAN] | 0.714 |
| Q15113 | *PCOLCE* | Procollagen C-endopeptidase enhancer 1 OS=Homo sapiens GN=PCOLCE PE=1 SV=2 - [PCOC1_HUMAN] | 1.384 |
| P04075 | *ALDOA* | Fructose-bisphosphate aldolase A OS=Homo sapiens GN=ALDOA PE=1 SV=2 - [ALDOA_HUMAN] | 1.268 |
| Q92888 | *ARHGEF1* | Isoform 2 of Rho guanine nucleotide exchange factor 1 OS=Homo sapiens GN=ARHGEF1 - [ARHG1_HUMAN] | 1.121 |
| P07902 | *GALT* | Galactose-1-phosphate uridylyltransferase OS=Homo sapiens GN=GALT PE=1 SV=3 - [GALT_HUMAN] | 0.916 |
| Q96BZ8 | *LENG1* | Leukocyte receptor cluster member 1 OS=Homo sapiens GN=LENG1 PE=1 SV=1 - [LENG1_HUMAN] | 1.170 |
| Q9NXW2 | *DNAJB12* | DnaJ homolog subfamily B member 12 OS=Homo sapiens GN=DNAJB12 PE=1 SV=4 - [DJB12_HUMAN] | 0.929 |
| P04004 | *VTN* | Vitronectin OS=Homo sapiens GN=VTN PE=1 SV=1 - [VTNC_HUMAN] | 0.570 |
| Q9Y2B0 | *CNPY2* | Protein canopy homolog 2 OS=Homo sapiens GN=CNPY2 PE=1 SV=1 - [CNPY2_HUMAN] | 0.908 |
| Q9BZF1 | *OSBPL8* | Isoform 2 of Oxysterol-binding protein-related protein 8 OS=Homo sapiens GN=OSBPL8 - [OSBL8_HUMAN] | 1.075 |
| Q96KR1 | *ZFR* | Zinc finger RNA-binding protein OS=Homo sapiens GN=ZFR PE=1 SV=2 - [ZFR_HUMAN] | 1.074 |
| Q7Z7H5 | *TMED4* | Isoform 3 of Transmembrane emp24 domain-containing protein 4 OS=Homo sapiens GN=TMED4 - [TMED4_HUMAN] | 1.105 |
| O94875 | *SORBS2* | Isoform 3 of Sorbin and SH3 domain-containing protein 2 OS=Homo sapiens GN=SORBS2 - [SRBS2_HUMAN] | 0.764 |
| Q14240 | *EIF4A2* | Eukaryotic initiation factor 4A-II OS=Homo sapiens GN=EIF4A2 PE=1 SV=2 - [IF4A2_HUMAN] | 0.898 |
| Q86X29 | *LSR* | Lipolysis-stimulated lipoprotein receptor OS=Homo sapiens GN=LSR PE=1 SV=4 - [LSR_HUMAN] | 0.734 |
| P27361 | *MAPK3* | Mitogen-activated protein kinase 3 OS=Homo sapiens GN=MAPK3 PE=1 SV=4 - [MK03_HUMAN] | 1.145 |
| O75822 | *EIF3J* | Eukaryotic translation initiation factor 3 subunit J OS=Homo sapiens GN=EIF3J PE=1 SV=2 - [EIF3J_HUMAN] | 1.104 |
| Q14764 | *MVP* | Major vault protein OS=Homo sapiens GN=MVP PE=1 SV=4 - [MVP_HUMAN] | 1.270 |
| Q9UPQ9 | *TNRC6B* | Isoform 2 of Trinucleotide repeat-containing gene 6B protein OS=Homo sapiens GN=TNRC6B - [TNR6B_HUMAN] | 1.099 |
| P05026 | *ATP1B1* | Isoform 2 of Sodium/potassium-transporting ATPase subunit beta-1 OS=Homo sapiens GN=ATP1B1 - [AT1B1_HUMAN] | 0.820 |
| P28066 | *PSMA5* | Proteasome subunit alpha type-5 OS=Homo sapiens GN=PSMA5 PE=1 SV=3 - [PSA5_HUMAN] | 1.067 |
| P40925 | *MDH1* | Malate dehydrogenase, cytoplasmic OS=Homo sapiens GN=MDH1 PE=1 SV=4 - [MDHC_HUMAN] | 0.944 |
| P49792 | *RANBP2* | E3 SUMO-protein ligase RanBP2 OS=Homo sapiens GN=RANBP2 PE=1 SV=2 - [RBP2_HUMAN] | 0.929 |
| Q9Y285 | *FARSA* | Phenylalanine--tRNA ligase alpha chain OS=Homo sapiens GN=FARSA PE=1 SV=3 - [SYFA_HUMAN] | 1.242 |
| Q86V48 | *LUZP1* | Isoform 2 of Leucine zipper protein 1 OS=Homo sapiens GN=LUZP1 - [LUZP1_HUMAN] | 1.179 |
| Q9UM47 | *NOTCH3* | Neurogenic locus notch homolog protein 3 OS=Homo sapiens GN=NOTCH3 PE=1 SV=2 - [NOTC3_HUMAN] | 1.245 |
| P52569 | *SLC7A2* | Low affinity cationic amino acid transporter 2 OS=Homo sapiens GN=SLC7A2 PE=1 SV=2 - [CTR2_HUMAN] | 0.772 |
| O15327 | *INPP4B* | Type II inositol-3,4-bisphosphate 4-phosphatase OS=Homo sapiens GN=INPP4B PE=2 SV=4 - [INP4B_HUMAN] | 0.772 |
| Q5XXA6 | *ANO1* | Anoctamin-1 OS=Homo sapiens GN=ANO1 PE=1 SV=1 - [ANO1_HUMAN] | 1.233 |
| Q96LD8 | *SENP8* | Sentrin-specific protease 8 OS=Homo sapiens GN=SENP8 PE=1 SV=1 - [SENP8_HUMAN] | 0.934 |
| Q49A26 | *GLYR1* | Isoform 5 of Putative oxidoreductase GLYR1 OS=Homo sapiens GN=GLYR1 - [GLYR1_HUMAN] | 1.112 |
| Q969G3 | *SMARCE1* | Isoform 2 of SWI/SNF-related matrix-associated actin-dependent regulator of chromatin subfamily E member 1 OS=Homo sapiens GN=SMARCE1 - [SMCE1_HUMAN] | 1.131 |
| P23280 | *CA6* | Carbonic anhydrase 6 OS=Homo sapiens GN=CA6 PE=1 SV=3 - [CAH6_HUMAN] | 8.600 |
| Q15208 | *STK38* | Serine/threonine-protein kinase 38 OS=Homo sapiens GN=STK38 PE=1 SV=1 - [STK38_HUMAN] | 1.182 |
| Q99504 | *EYA3* | Isoform 2 of Eyes absent homolog 3 OS=Homo sapiens GN=EYA3 - [EYA3_HUMAN] | 1.157 |
| Q01650 | *SLC7A5* | Large neutral amino acids transporter small subunit 1 OS=Homo sapiens GN=SLC7A5 PE=1 SV=2 - [LAT1_HUMAN] | 1.313 |
| P49590 | *HARS2* | Probable histidine--tRNA ligase, mitochondrial OS=Homo sapiens GN=HARS2 PE=1 SV=1 - [SYHM_HUMAN] | 1.170 |
| P23258 | *TUBG1* | Tubulin gamma-1 chain OS=Homo sapiens GN=TUBG1 PE=1 SV=2 - [TBG1_HUMAN] | 1.082 |
| P68400 | *CSNK2A1* | Casein kinase II subunit alpha OS=Homo sapiens GN=CSNK2A1 PE=1 SV=1 - [CSK21_HUMAN] | 1.067 |
| P06881 | *CALCA* | Calcitonin gene-related peptide 1 OS=Homo sapiens GN=CALCA PE=1 SV=3 - [CALCA_HUMAN] | 0.503 |
| Q9NYH9 | *UTP6* | U3 small nucleolar RNA-associated protein 6 homolog OS=Homo sapiens GN=UTP6 PE=1 SV=2 - [UTP6_HUMAN] | 1.085 |
| O15204 | *ADAMDEC1* | ADAM DEC1 OS=Homo sapiens GN=ADAMDEC1 PE=1 SV=2 - [ADEC1_HUMAN] | 0.601 |
| Q14103 | *HNRNPD* | Isoform 3 of Heterogeneous nuclear ribonucleoprotein D0 OS=Homo sapiens GN=HNRNPD - [HNRPD_HUMAN] | 1.097 |
| Q49MG5 | *MAP9* | Microtubule-associated protein 9 OS=Homo sapiens GN=MAP9 PE=1 SV=3 - [MAP9_HUMAN] | 1.155 |
| Q15388 | *TOMM20* | Mitochondrial import receptor subunit TOM20 homolog OS=Homo sapiens GN=TOMM20 PE=1 SV=1 - [TOM20_HUMAN] | 1.106 |
| Q969H8 | *MYDGF* | UPF0556 protein C19orf10 OS=Homo sapiens GN=C19orf10 PE=1 SV=1 - [CS010_HUMAN] | 0.830 |
| P04083 | *ANXA1* | Annexin A1 OS=Homo sapiens GN=ANXA1 PE=1 SV=2 - [ANXA1_HUMAN] | 0.668 |
| Q96D46 | *NMD3* | 60S ribosomal export protein NMD3 OS=Homo sapiens GN=NMD3 PE=1 SV=1 - [NMD3_HUMAN] | 0.890 |
| Q9NUP9 | *LIN7C* | Protein lin-7 homolog C OS=Homo sapiens GN=LIN7C PE=1 SV=1 - [LIN7C_HUMAN] | 0.874 |
| P08263 | *GSTA1* | Glutathione S-transferase A1 OS=Homo sapiens GN=GSTA1 PE=1 SV=3 - [GSTA1_HUMAN] | 10.196 |
| Q99848 | *EBNA1BP2* | Probable rRNA-processing protein EBP2 OS=Homo sapiens GN=EBNA1BP2 PE=1 SV=2 - [EBP2_HUMAN] | 1.114 |
| P53004 | *BLVRA* | Biliverdin reductase A OS=Homo sapiens GN=BLVRA PE=1 SV=2 - [BIEA_HUMAN] | 0.849 |
| P12830 | *CDH1* | Cadherin-1 OS=Homo sapiens GN=CDH1 PE=1 SV=3 - [CADH1_HUMAN] | 0.823 |
| Q9NSD9 | *FARSB* | Phenylalanine--tRNA ligase beta chain OS=Homo sapiens GN=FARSB PE=1 SV=3 - [SYFB_HUMAN] | 1.201 |
| Q8IXM2 | *C17orf49* | Chromatin complexes subunit BAP18 OS=Homo sapiens GN=BAP18 PE=1 SV=1 - [BAP18_HUMAN] | 1.172 |
| Q96S66 | *CLCC1* | Chloride channel CLIC-like protein 1 OS=Homo sapiens GN=CLCC1 PE=1 SV=1 - [CLCC1_HUMAN] | 0.882 |
| O75015 | *FCGR3B* | Low affinity immunoglobulin gamma Fc region receptor III-B OS=Homo sapiens GN=FCGR3B PE=1 SV=2 - [FCG3B_HUMAN] | 0.630 |
| P62913 | *RPL11* | Isoform 2 of 60S ribosomal protein L11 OS=Homo sapiens GN=RPL11 - [RL11_HUMAN] | 1.063 |
| P49770 | *EIF2B2* | Translation initiation factor eIF-2B subunit beta OS=Homo sapiens GN=EIF2B2 PE=1 SV=3 - [EI2BB_HUMAN] | 1.095 |
| Q13435 | *SF3B2* | Splicing factor 3B subunit 2 OS=Homo sapiens GN=SF3B2 PE=1 SV=2 - [SF3B2_HUMAN] | 0.932 |
| P45880 | *VDAC2* | Isoform 2 of Voltage-dependent anion-selective channel protein 2 OS=Homo sapiens GN=VDAC2 - [VDAC2_HUMAN] | 0.898 |
| Q9P273 | *TENM3* | Teneurin-3 OS=Homo sapiens GN=ODZ3 PE=2 SV=3 - [TEN3_HUMAN] | 0.540 |
| Q99567 | *NUP88* | Nuclear pore complex protein Nup88 OS=Homo sapiens GN=NUP88 PE=1 SV=2 - [NUP88_HUMAN] | 1.115 |
| Q99828 | *CIB1* | Calcium and integrin-binding protein 1 OS=Homo sapiens GN=CIB1 PE=1 SV=4 - [CIB1_HUMAN] | 0.857 |
| O95487 | *SEC24B* | Isoform 2 of Protein transport protein Sec24B OS=Homo sapiens GN=SEC24B - [SC24B_HUMAN] | 1.063 |
| O95249 | *GOSR1* | Golgi SNAP receptor complex member 1 OS=Homo sapiens GN=GOSR1 PE=1 SV=1 - [GOSR1_HUMAN] | 1.142 |
| Q9UMS4 | *PRPF19* | Pre-mRNA-processing factor 19 OS=Homo sapiens GN=PRPF19 PE=1 SV=1 - [PRP19_HUMAN] | 1.082 |
| P05387 | *RPLP2* | 60S acidic ribosomal protein P2 OS=Homo sapiens GN=RPLP2 PE=1 SV=1 - [RLA2_HUMAN] | 1.066 |
| P26641 | *EEF1G* | Elongation factor 1-gamma OS=Homo sapiens GN=EEF1G PE=1 SV=3 - [EF1G_HUMAN] | 1.079 |
| Q9NQW7 | *XPNPEP1* | Isoform 2 of Xaa-Pro aminopeptidase 1 OS=Homo sapiens GN=XPNPEP1 - [XPP1_HUMAN] | 1.089 |
| Q9Y3E7 | *CHMP3* | Isoform 4 of Charged multivesicular body protein 3 OS=Homo sapiens GN=CHMP3 - [CHMP3_HUMAN] | 1.155 |
| Q8IVD9 | *NUDCD3* | NudC domain-containing protein 3 OS=Homo sapiens GN=NUDCD3 PE=1 SV=3 - [NUDC3_HUMAN] | 1.108 |
| O43318 | *MAP3K7* | Isoform 1D of Mitogen-activated protein kinase kinase kinase 7 OS=Homo sapiens GN=MAP3K7 - [M3K7_HUMAN] | 1.101 |
| Q9UBN7 | *HDAC6* | Histone deacetylase 6 OS=Homo sapiens GN=HDAC6 PE=1 SV=2 - [HDAC6_HUMAN] | 1.075 |
| P63098 | *PPP3R1* | Calcineurin subunit B type 1 OS=Homo sapiens GN=PPP3R1 PE=1 SV=2 - [CANB1_HUMAN] | 1.105 |
| Q7Z3B4 | *NUP54* | Isoform 2 of Nucleoporin p54 OS=Homo sapiens GN=NUP54 - [NUP54_HUMAN] | 1.113 |
| O43660 | *PLRG1* | Pleiotropic regulator 1 OS=Homo sapiens GN=PLRG1 PE=1 SV=1 - [PLRG1_HUMAN] | 1.080 |
| O00479 | *HMGN4* | High mobility group nucleosome-binding domain-containing protein 4 OS=Homo sapiens GN=HMGN4 PE=1 SV=3 - [HMGN4_HUMAN] | 1.292 |
| Q7L7X3 | *TAOK1* | Serine/threonine-protein kinase TAO1 OS=Homo sapiens GN=TAOK1 PE=1 SV=1 - [TAOK1_HUMAN] | 0.918 |
| Q03518 | *TAP1* | Antigen peptide transporter 1 OS=Homo sapiens GN=TAP1 PE=1 SV=2 - [TAP1_HUMAN] | 1.919 |
| P39687 | *ANP32A* | Acidic leucine-rich nuclear phosphoprotein 32 family member A OS=Homo sapiens GN=ANP32A PE=1 SV=1 - [AN32A_HUMAN] | 1.109 |
| Q92783 | *STAM* | Isoform 2 of Signal transducing adapter molecule 1 OS=Homo sapiens GN=STAM - [STAM1_HUMAN] | 1.158 |
| Q15084 | *PDIA6* | Protein disulfide-isomerase A6 OS=Homo sapiens GN=PDIA6 PE=1 SV=1 - [PDIA6_HUMAN] | 1.151 |
| Q14444 | *CAPRIN1* | Isoform 2 of Caprin-1 OS=Homo sapiens GN=CAPRIN1 - [CAPR1_HUMAN] | 1.112 |
| P14621 | *ACYP2* | Acylphosphatase-2 OS=Homo sapiens GN=ACYP2 PE=1 SV=2 - [ACYP2_HUMAN] | 0.894 |
| O43290 | *SART1* | U4/U6.U5 tri-snRNP-associated protein 1 OS=Homo sapiens GN=SART1 PE=1 SV=1 - [SNUT1_HUMAN] | 1.096 |
| Q14596 | *NBR1* | Isoform 2 of Next to BRCA1 gene 1 protein OS=Homo sapiens GN=NBR1 - [NBR1_HUMAN] | 0.879 |
| O14531 | *DPYSL4* | Dihydropyrimidinase-related protein 4 OS=Homo sapiens GN=DPYSL4 PE=1 SV=2 - [DPYL4_HUMAN] | 1.732 |
| O43819 | *SCO2* | Protein SCO2 homolog, mitochondrial OS=Homo sapiens GN=SCO2 PE=1 SV=3 - [SCO2_HUMAN] | 1.204 |
| Q9H2U1 | *DHX36* | Isoform 3 of Probable ATP-dependent RNA helicase DHX36 OS=Homo sapiens GN=DHX36 - [DHX36_HUMAN] | 0.874 |
| Q9BX95 | *SGPP1* | Sphingosine-1-phosphate phosphatase 1 OS=Homo sapiens GN=SGPP1 PE=1 SV=2 - [SGPP1_HUMAN] | 1.128 |
| Q96A65 | *EXOC4* | Exocyst complex component 4 OS=Homo sapiens GN=EXOC4 PE=1 SV=1 - [EXOC4_HUMAN] | 0.929 |
| P16112 | *#N/A* | Isoform 3 of Aggrecan core protein OS=Homo sapiens GN=ACAN - [PGCA_HUMAN] | 1.616 |
| P52306 | *RAP1GDS1* | Isoform 2 of Rap1 GTPase-GDP dissociation stimulator 1 OS=Homo sapiens GN=RAP1GDS1 - [GDS1_HUMAN] | 1.091 |
| P49840 | *GSK3A* | Glycogen synthase kinase-3 alpha OS=Homo sapiens GN=GSK3A PE=1 SV=2 - [GSK3A_HUMAN] | 0.946 |
| Q92817 | *EVPL* | Envoplakin OS=Homo sapiens GN=EVPL PE=1 SV=3 - [EVPL_HUMAN] | 1.386 |
| P28838 | *LAP3* | Isoform 2 of Cytosol aminopeptidase OS=Homo sapiens GN=LAP3 - [AMPL_HUMAN] | 1.170 |
| Q86U44 | *METTL3* | N6-adenosine-methyltransferase 70 kDa subunit OS=Homo sapiens GN=METTL3 PE=1 SV=2 - [MTA70_HUMAN] | 0.933 |
| Q14137 | *BOP1* | Ribosome biogenesis protein BOP1 OS=Homo sapiens GN=BOP1 PE=1 SV=2 - [BOP1_HUMAN] | 1.113 |
| P19075 | *TSPAN8* | Tetraspanin-8 OS=Homo sapiens GN=TSPAN8 PE=1 SV=1 - [TSN8_HUMAN] | 0.613 |
| P98082 | *DAB2* | Isoform 2 of Disabled homolog 2 OS=Homo sapiens GN=DAB2 - [DAB2_HUMAN] | 0.803 |
| Q5UCC4 | *EMC10* | Isoform 2 of UPF0510 protein INM02 OS=Homo sapiens GN=C19orf63 - [INM02_HUMAN] | 0.903 |
| Q01813 | *PFKP* | 6-phosphofructokinase type C OS=Homo sapiens GN=PFKP PE=1 SV=2 - [K6PP_HUMAN] | 1.291 |
| Q8N6H7 | *ARFGAP2* | ADP-ribosylation factor GTPase-activating protein 2 OS=Homo sapiens GN=ARFGAP2 PE=1 SV=1 - [ARFG2_HUMAN] | 1.107 |
| O14745 | *SLC9A3R1* | Na(+)/H(+) exchange regulatory cofactor NHE-RF1 OS=Homo sapiens GN=SLC9A3R1 PE=1 SV=4 - [NHRF1_HUMAN] | 1.391 |
| Q14558 | *PRPSAP1* | Phosphoribosyl pyrophosphate synthase-associated protein 1 OS=Homo sapiens GN=PRPSAP1 PE=1 SV=2 - [KPRA_HUMAN] | 0.895 |
| P62269 | *RPS18* | 40S ribosomal protein S18 OS=Homo sapiens GN=RPS18 PE=1 SV=3 - [RS18_HUMAN] | 1.073 |
| Q6XQN6 | *NAPRT* | Isoform 3 of Nicotinate phosphoribosyltransferase OS=Homo sapiens GN=NAPRT1 - [PNCB_HUMAN] | 1.196 |
| P15104 | *GLUL* | Glutamine synthetase OS=Homo sapiens GN=GLUL PE=1 SV=4 - [GLNA_HUMAN] | 0.858 |
| P48730 | *CSNK1D* | Isoform 2 of Casein kinase I isoform delta OS=Homo sapiens GN=CSNK1D - [KC1D_HUMAN] | 1.070 |
| Q96Q05 | *TRAPPC9* | Isoform 3 of Trafficking protein particle complex subunit 9 OS=Homo sapiens GN=TRAPPC9 - [TPPC9_HUMAN] | 1.085 |
| Q99719 | *SEPT5* | Septin-5 OS=Homo sapiens GN=SEPT5 PE=1 SV=1 - [SEPT5_HUMAN] | 1.161 |
| Q9UMY1 | *NOL7* | Isoform 2 of Nucleolar protein 7 OS=Homo sapiens GN=NOL7 - [NOL7_HUMAN] | 1.079 |
| P05155 | *SERPING1* | Plasma protease C1 inhibitor OS=Homo sapiens GN=SERPING1 PE=1 SV=2 - [IC1_HUMAN] | 0.814 |
| P00519 | *ABL1* | Tyrosine-protein kinase ABL1 OS=Homo sapiens GN=ABL1 PE=1 SV=4 - [ABL1_HUMAN] | 1.164 |
| P61764 | *STXBP1* | Syntaxin-binding protein 1 OS=Homo sapiens GN=STXBP1 PE=1 SV=1 - [STXB1_HUMAN] | 0.879 |
| P04150 | *NR3C1* | Isoform GR-A beta of Glucocorticoid receptor OS=Homo sapiens GN=NR3C1 - [GCR_HUMAN] | 1.120 |
| Q9Y530 | *OARD1* | O-acetyl-ADP-ribose deacetylase C6orf130 OS=Homo sapiens GN=C6orf130 PE=1 SV=2 - [CF130_HUMAN] | 0.894 |
| Q969S9 | *GFM2* | Isoform 2 of Ribosome-releasing factor 2, mitochondrial OS=Homo sapiens GN=GFM2 - [RRF2M_HUMAN] | 0.816 |
| Q96IY4 | *CPB2* | Carboxypeptidase B2 OS=Homo sapiens GN=CPB2 PE=1 SV=2 - [CBPB2_HUMAN] | 0.783 |
| Q96HY7 | *DHTKD1* | Probable 2-oxoglutarate dehydrogenase E1 component DHKTD1, mitochondrial OS=Homo sapiens GN=DHTKD1 PE=2 SV=2 - [DHTK1_HUMAN] | 0.860 |
| O75348 | *ATP6V1G1* | V-type proton ATPase subunit G 1 OS=Homo sapiens GN=ATP6V1G1 PE=1 SV=3 - [VATG1_HUMAN] | 0.919 |
| P53007 | *SLC25A1* | Tricarboxylate transport protein, mitochondrial OS=Homo sapiens GN=SLC25A1 PE=1 SV=2 - [TXTP_HUMAN] | 0.873 |
| P42224 | *STAT1* | Isoform Beta of Signal transducer and activator of transcription 1-alpha/beta OS=Homo sapiens GN=STAT1 - [STAT1_HUMAN] | 1.538 |
| Q9UHD8 | *SEPT9* | Isoform 7 of Septin-9 OS=Homo sapiens GN=SEPT9 - [SEPT9_HUMAN] | 1.138 |
| Q9Y4I1 | *MYO5A* | Isoform 2 of Myosin-Va OS=Homo sapiens GN=MYO5A - [MYO5A_HUMAN] | 0.877 |
| Q96K76 | *USP47* | Ubiquitin carboxyl-terminal hydrolase 47 OS=Homo sapiens GN=USP47 PE=1 SV=3 - [UBP47_HUMAN] | 1.131 |
| Q9BZZ5 | *API5* | Isoform 1 of Apoptosis inhibitor 5 OS=Homo sapiens GN=API5 - [API5_HUMAN] | 1.099 |
| P35573 | *AGL* | Isoform 5 of Glycogen debranching enzyme OS=Homo sapiens GN=AGL - [GDE_HUMAN] | 0.901 |
| Q8NI27 | *THOC2* | THO complex subunit 2 OS=Homo sapiens GN=THOC2 PE=1 SV=2 - [THOC2_HUMAN] | 1.077 |
| Q13796 | *SHROOM2* | Protein Shroom2 OS=Homo sapiens GN=SHROOM2 PE=1 SV=1 - [SHRM2_HUMAN] | 1.186 |
| Q5JTH9 | *RRP12* | Isoform 2 of RRP12-like protein OS=Homo sapiens GN=RRP12 - [RRP12_HUMAN] | 1.139 |
| P98194 | *ATP2C1* | Isoform 2 of Calcium-transporting ATPase type 2C member 1 OS=Homo sapiens GN=ATP2C1 - [AT2C1_HUMAN] | 0.894 |
| Q6STE5 | *SMARCD3* | Isoform 2 of SWI/SNF-related matrix-associated actin-dependent regulator of chromatin subfamily D member 3 OS=Homo sapiens GN=SMARCD3 - [SMRD3_HUMAN] | 0.806 |
| P83916 | *CBX1* | Chromobox protein homolog 1 OS=Homo sapiens GN=CBX1 PE=1 SV=1 - [CBX1_HUMAN] | 1.084 |
| Q14108 | *SCARB2* | Lysosome membrane protein 2 OS=Homo sapiens GN=SCARB2 PE=1 SV=2 - [SCRB2_HUMAN] | 1.159 |
| P02654 | *APOC1* | Apolipoprotein C-I OS=Homo sapiens GN=APOC1 PE=1 SV=1 - [APOC1_HUMAN] | 1.420 |
| Q9HC36 | *RNMTL1* | RNA methyltransferase-like protein 1 OS=Homo sapiens GN=RNMTL1 PE=1 SV=2 - [RMTL1_HUMAN] | 1.145 |
| O15160 | *POLR1C* | Isoform 2 of DNA-directed RNA polymerases I and III subunit RPAC1 OS=Homo sapiens GN=POLR1C - [RPAC1_HUMAN] | 1.108 |
| Q14573 | *ITPR3* | Inositol 1,4,5-trisphosphate receptor type 3 OS=Homo sapiens GN=ITPR3 PE=1 SV=2 - [ITPR3_HUMAN] | 1.155 |
| Q13423 | *NNT* | NAD(P) transhydrogenase, mitochondrial OS=Homo sapiens GN=NNT PE=1 SV=3 - [NNTM_HUMAN] | 1.158 |
| Q6ZXV5 | *TMTC3* | Isoform 2 of Transmembrane and TPR repeat-containing protein 3 OS=Homo sapiens GN=TMTC3 - [TMTC3_HUMAN] | 1.091 |
| P15170 | *GSPT1* | Eukaryotic peptide chain release factor GTP-binding subunit ERF3A OS=Homo sapiens GN=GSPT1 PE=1 SV=1 - [ERF3A_HUMAN] | 1.231 |
| P61160 | *ACTR2* | Actin-related protein 2 OS=Homo sapiens GN=ACTR2 PE=1 SV=1 - [ARP2_HUMAN] | 1.059 |
| Q96MW1 | *CCDC43* | Coiled-coil domain-containing protein 43 OS=Homo sapiens GN=CCDC43 PE=1 SV=2 - [CCD43_HUMAN] | 1.166 |
| P20711 | *DDC* | Aromatic-L-amino-acid decarboxylase OS=Homo sapiens GN=DDC PE=1 SV=2 - [DDC_HUMAN] | 1.351 |
| Q8NCW6 | *GALNT11* | Isoform 2 of Polypeptide N-acetylgalactosaminyltransferase 11 OS=Homo sapiens GN=GALNT11 - [GLT11_HUMAN] | 0.896 |
| Q53ET0 | *CRTC2* | CREB-regulated transcription coactivator 2 OS=Homo sapiens GN=CRTC2 PE=1 SV=2 - [CRTC2_HUMAN] | 1.099 |
| Q86YT6 | *MIB1* | E3 ubiquitin-protein ligase MIB1 OS=Homo sapiens GN=MIB1 PE=2 SV=1 - [MIB1_HUMAN] | 1.092 |
| P10644 | *PRKAR1A* | cAMP-dependent protein kinase type I-alpha regulatory subunit OS=Homo sapiens GN=PRKAR1A PE=1 SV=1 - [KAP0_HUMAN] | 0.916 |
| O95456 | *PSMG1* | Isoform 2 of Proteasome assembly chaperone 1 OS=Homo sapiens GN=PSMG1 - [PSMG1_HUMAN] | 1.165 |
| Q9HAU5 | *UPF2* | Regulator of nonsense transcripts 2 OS=Homo sapiens GN=UPF2 PE=1 SV=1 - [RENT2_HUMAN] | 0.944 |
| Q8N5M4 | *TTC9C* | Tetratricopeptide repeat protein 9C OS=Homo sapiens GN=TTC9C PE=1 SV=1 - [TTC9C_HUMAN] | 1.167 |
| O00231 | *PSMD11* | 26S proteasome non-ATPase regulatory subunit 11 OS=Homo sapiens GN=PSMD11 PE=1 SV=3 - [PSD11_HUMAN] | 1.068 |
| Q09666 | *AHNAK* | Neuroblast differentiation-associated protein AHNAK OS=Homo sapiens GN=AHNAK PE=1 SV=2 - [AHNK_HUMAN] | 0.757 |
| P08729 | *KRT7* | Keratin, type II cytoskeletal 7 OS=Homo sapiens GN=KRT7 PE=1 SV=5 - [K2C7_HUMAN] | 1.411 |
| O75475 | *PSIP1* | PC4 and SFRS1-interacting protein OS=Homo sapiens GN=PSIP1 PE=1 SV=1 - [PSIP1_HUMAN] | 0.882 |
| P16035 | *TIMP2* | Metalloproteinase inhibitor 2 OS=Homo sapiens GN=TIMP2 PE=1 SV=2 - [TIMP2_HUMAN] | 0.727 |
| Q13616 | *CUL1* | Cullin-1 OS=Homo sapiens GN=CUL1 PE=1 SV=2 - [CUL1_HUMAN] | 1.082 |
| Q8NBU5 | *ATAD1* | ATPase family AAA domain-containing protein 1 OS=Homo sapiens GN=ATAD1 PE=1 SV=1 - [ATAD1_HUMAN] | 1.150 |
| P31040 | *SDHA* | Succinate dehydrogenase [ubiquinone] flavoprotein subunit, mitochondrial OS=Homo sapiens GN=SDHA PE=1 SV=2 - [DHSA_HUMAN] | 0.903 |
| Q9NZ72 | *STMN3* | Stathmin-3 OS=Homo sapiens GN=STMN3 PE=1 SV=3 - [STMN3_HUMAN] | 1.125 |
| O60701 | *UGDH* | UDP-glucose 6-dehydrogenase OS=Homo sapiens GN=UGDH PE=1 SV=1 - [UGDH_HUMAN] | 1.164 |
| P09012 | *SNRPA* | U1 small nuclear ribonucleoprotein A OS=Homo sapiens GN=SNRPA PE=1 SV=3 - [SNRPA_HUMAN] | 0.934 |
| Q96KP1 | *EXOC2* | Exocyst complex component 2 OS=Homo sapiens GN=EXOC2 PE=1 SV=1 - [EXOC2_HUMAN] | 0.820 |
| Q9UL18 | *AGO1* | Protein argonaute-1 OS=Homo sapiens GN=EIF2C1 PE=1 SV=3 - [AGO1_HUMAN] | 1.076 |
| Q7L5N1 | *COPS6* | COP9 signalosome complex subunit 6 OS=Homo sapiens GN=COPS6 PE=1 SV=1 - [CSN6_HUMAN] | 0.932 |
| P06576 | *ATP5B* | ATP synthase subunit beta, mitochondrial OS=Homo sapiens GN=ATP5B PE=1 SV=3 - [ATPB_HUMAN] | 0.900 |
| Q16610 | *ECM1* | Extracellular matrix protein 1 OS=Homo sapiens GN=ECM1 PE=1 SV=2 - [ECM1_HUMAN] | 1.357 |
| Q8N573 | *OXR1* | Isoform 2 of Oxidation resistance protein 1 OS=Homo sapiens GN=OXR1 - [OXR1_HUMAN] | 1.411 |
| P81605 | *DCD* | Dermcidin OS=Homo sapiens GN=DCD PE=1 SV=2 - [DCD_HUMAN] | 0.841 |
| Q99426 | *TBCB* | Tubulin-folding cofactor B OS=Homo sapiens GN=TBCB PE=1 SV=2 - [TBCB_HUMAN] | 0.919 |
| Q9BUQ8 | *DDX23* | Probable ATP-dependent RNA helicase DDX23 OS=Homo sapiens GN=DDX23 PE=1 SV=3 - [DDX23_HUMAN] | 1.080 |
| Q6NVY1 | *HIBCH* | 3-hydroxyisobutyryl-CoA hydrolase, mitochondrial OS=Homo sapiens GN=HIBCH PE=1 SV=2 - [HIBCH_HUMAN] | 0.891 |
| Q7L4I2 | *RSRC2* | Isoform 2 of Arginine/serine-rich coiled-coil protein 2 OS=Homo sapiens GN=RSRC2 - [RSRC2_HUMAN] | 1.070 |
| P48681 | *NES* | Nestin OS=Homo sapiens GN=NES PE=1 SV=2 - [NEST_HUMAN] | 1.161 |
| Q99598 | *TSNAX* | Translin-associated protein X OS=Homo sapiens GN=TSNAX PE=1 SV=1 - [TSNAX_HUMAN] | 0.936 |
| Q86X29 | *LSR* | Isoform 3 of Lipolysis-stimulated lipoprotein receptor OS=Homo sapiens GN=LSR - [LSR_HUMAN] | 0.690 |
| Q9HBU1 | *BARX1* | Isoform 2 of Homeobox protein BarH-like 1 OS=Homo sapiens GN=BARX1 - [BARX1_HUMAN] | 1.334 |
| P61158 | *ACTR3* | Actin-related protein 3 OS=Homo sapiens GN=ACTR3 PE=1 SV=3 - [ARP3_HUMAN] | 1.064 |
| P54253 | *ATXN1* | Ataxin-1 OS=Homo sapiens GN=ATXN1 PE=1 SV=2 - [ATX1_HUMAN] | 1.188 |
| Q13445 | *TMED1* | Transmembrane emp24 domain-containing protein 1 OS=Homo sapiens GN=TMED1 PE=1 SV=1 - [TMED1_HUMAN] | 1.064 |
| Q9BWH6 | *RPAP1* | Isoform 2 of RNA polymerase II-associated protein 1 OS=Homo sapiens GN=RPAP1 - [RPAP1_HUMAN] | 1.050 |
| Q05655 | *PRKCD* | Protein kinase C delta type OS=Homo sapiens GN=PRKCD PE=1 SV=2 - [KPCD_HUMAN] | 0.810 |
| P04155 | *TFF1* | Trefoil factor 1 OS=Homo sapiens GN=TFF1 PE=1 SV=1 - [TFF1_HUMAN] | 3.104 |
| Q14160 | *SCRIB* | Protein scribble homolog OS=Homo sapiens GN=SCRIB PE=1 SV=4 - [SCRIB_HUMAN] | 1.085 |
| P45974 | *USP5* | Isoform Short of Ubiquitin carboxyl-terminal hydrolase 5 OS=Homo sapiens GN=USP5 - [UBP5_HUMAN] | 1.044 |
| P07195 | *LDHB* | L-lactate dehydrogenase B chain OS=Homo sapiens GN=LDHB PE=1 SV=2 - [LDHB_HUMAN] | 0.808 |
| P41214 | *EIF2D* | Eukaryotic translation initiation factor 2D OS=Homo sapiens GN=EIF2D PE=1 SV=3 - [EIF2D_HUMAN] | 1.142 |
| Q9UBX1 | *CTSF* | Cathepsin F OS=Homo sapiens GN=CTSF PE=1 SV=1 - [CATF_HUMAN] | 0.646 |
| P84095 | *RHOG* | Rho-related GTP-binding protein RhoG OS=Homo sapiens GN=RHOG PE=1 SV=1 - [RHOG_HUMAN] | 0.899 |
| P12532 | *CKMT1A* | Creatine kinase U-type, mitochondrial OS=Homo sapiens GN=CKMT1A PE=1 SV=1 - [KCRU_HUMAN] | 1.148 |
| Q9Y2R9 | *MRPS7* | 28S ribosomal protein S7, mitochondrial OS=Homo sapiens GN=MRPS7 PE=1 SV=2 - [RT07_HUMAN] | 0.942 |
| Q8N6Y2 | *LRRC17* | Isoform 2 of Leucine-rich repeat-containing protein 17 OS=Homo sapiens GN=LRRC17 - [LRC17_HUMAN] | 0.736 |
| P00568 | *AK1* | Adenylate kinase isoenzyme 1 OS=Homo sapiens GN=AK1 PE=1 SV=3 - [KAD1_HUMAN] | 0.925 |
| O94887 | *FARP2* | FERM, RhoGEF and pleckstrin domain-containing protein 2 OS=Homo sapiens GN=FARP2 PE=1 SV=3 - [FARP2_HUMAN] | 0.835 |
| Q07666 | *KHDRBS1* | Isoform 2 of KH domain-containing, RNA-binding, signal transduction-associated protein 1 OS=Homo sapiens GN=KHDRBS1 - [KHDR1_HUMAN] | 1.099 |
| P10301 | *RRAS* | Ras-related protein R-Ras OS=Homo sapiens GN=RRAS PE=1 SV=1 - [RRAS_HUMAN] | 0.793 |
| Q9P2J5 | *LARS* | Leucine--tRNA ligase, cytoplasmic OS=Homo sapiens GN=LARS PE=1 SV=2 - [SYLC_HUMAN] | 1.076 |
| P49368 | *CCT3* | T-complex protein 1 subunit gamma OS=Homo sapiens GN=CCT3 PE=1 SV=4 - [TCPG_HUMAN] | 1.076 |
| Q9UJC5 | *SH3BGRL2* | SH3 domain-binding glutamic acid-rich-like protein 2 OS=Homo sapiens GN=SH3BGRL2 PE=1 SV=2 - [SH3L2_HUMAN] | 0.826 |
| O43708 | *GSTZ1* | Isoform 2 of Maleylacetoacetate isomerase OS=Homo sapiens GN=GSTZ1 - [MAAI_HUMAN] | 1.197 |
| Q9H706 | *GAREM* | Isoform 2 of Protein FAM59A OS=Homo sapiens GN=FAM59A - [FA59A_HUMAN] | 0.871 |
| P28074 | *PSMB5* | Proteasome subunit beta type-5 OS=Homo sapiens GN=PSMB5 PE=1 SV=3 - [PSB5_HUMAN] | 0.885 |
| P25774 | *CTSS* | Cathepsin S OS=Homo sapiens GN=CTSS PE=1 SV=3 - [CATS_HUMAN] | 1.683 |
| Q9UBU6 | *FAM8A1* | Protein FAM8A1 OS=Homo sapiens GN=FAM8A1 PE=1 SV=1 - [FA8A1_HUMAN] | 1.085 |
| P23434 | *GCSH* | Glycine cleavage system H protein, mitochondrial OS=Homo sapiens GN=GCSH PE=1 SV=2 - [GCSH_HUMAN] | 0.772 |
| Q9Y6U3 | *SCIN* | Adseverin OS=Homo sapiens GN=SCIN PE=1 SV=4 - [ADSV_HUMAN] | 1.633 |
| Q93052 | *LPP* | Lipoma-preferred partner OS=Homo sapiens GN=LPP PE=1 SV=1 - [LPP_HUMAN] | 1.066 |
| Q96E39 | *RBMXL1* | RNA binding motif protein, X-linked-like-1 OS=Homo sapiens GN=RBMXL1 PE=1 SV=1 - [RMXL1_HUMAN] | 1.118 |
| Q9Y6U3 | *SCIN* | Isoform 3 of Adseverin OS=Homo sapiens GN=SCIN - [ADSV_HUMAN] | 1.696 |
| O95171 | *SCEL* | Isoform 2 of Sciellin OS=Homo sapiens GN=SCEL - [SCEL_HUMAN] | 0.718 |
| Q9H0E2 | *TOLLIP* | Toll-interacting protein OS=Homo sapiens GN=TOLLIP PE=1 SV=1 - [TOLIP_HUMAN] | 0.875 |
| Q96S15 | *WDR24* | Isoform 2 of WD repeat-containing protein 24 OS=Homo sapiens GN=WDR24 - [WDR24_HUMAN] | 0.904 |
| P01023 | *A2M* | Alpha-2-macroglobulin OS=Homo sapiens GN=A2M PE=1 SV=3 - [A2MG_HUMAN] | 0.770 |
| P52788 | *SMS* | Spermine synthase OS=Homo sapiens GN=SMS PE=1 SV=2 - [SPSY_HUMAN] | 1.069 |
| Q9BX40 | *LSM14B* | Protein LSM14 homolog B OS=Homo sapiens GN=LSM14B PE=1 SV=1 - [LS14B_HUMAN] | 1.114 |
| Q12765 | *SCRN1* | Secernin-1 OS=Homo sapiens GN=SCRN1 PE=1 SV=2 - [SCRN1_HUMAN] | 0.899 |
| Q96I25 | *RBM17* | Splicing factor 45 OS=Homo sapiens GN=RBM17 PE=1 SV=1 - [SPF45_HUMAN] | 1.056 |
| Q96AY3 | *FKBP10* | Peptidyl-prolyl cis-trans isomerase FKBP10 OS=Homo sapiens GN=FKBP10 PE=1 SV=1 - [FKB10_HUMAN] | 1.174 |
| Q6PUV4 | *CPLX2* | Complexin-2 OS=Homo sapiens GN=CPLX2 PE=1 SV=2 - [CPLX2_HUMAN] | 1.337 |
| Q8WVM7 | *STAG1* | Cohesin subunit SA-1 OS=Homo sapiens GN=STAG1 PE=1 SV=3 - [STAG1_HUMAN] | 0.906 |
| P16870 | *CPE* | Isoform 2 of Carboxypeptidase E OS=Homo sapiens GN=CPE - [CBPE_HUMAN] | 0.730 |
| P02686 | *MBP* | Isoform 2 of Myelin basic protein OS=Homo sapiens GN=MBP - [MBP_HUMAN] | 0.898 |
| O60832 | *DKC1* | H/ACA ribonucleoprotein complex subunit 4 OS=Homo sapiens GN=DKC1 PE=1 SV=3 - [DKC1_HUMAN] | 0.933 |
| P11182 | *DBT* | Lipoamide acyltransferase component of branched-chain alpha-keto acid dehydrogenase complex, mitochondrial OS=Homo sapiens GN=DBT PE=1 SV=3 - [ODB2_HUMAN] | 0.821 |
| Q9Y606 | *PUS1* | Isoform 2 of tRNA pseudouridine synthase A, mitochondrial OS=Homo sapiens GN=PUS1 - [TRUA_HUMAN] | 1.101 |
| Q5QP82 | *DCAF10* | Isoform 2 of DDB1- and CUL4-associated factor 10 OS=Homo sapiens GN=DCAF10 - [DCA10_HUMAN] | 1.069 |
| P55327 | *TPD52* | Isoform 2 of Tumor protein D52 OS=Homo sapiens GN=TPD52 - [TPD52_HUMAN] | 1.195 |
| P37108 | *SRP14* | Signal recognition particle 14 kDa protein OS=Homo sapiens GN=SRP14 PE=1 SV=2 - [SRP14_HUMAN] | 0.917 |
| Q9BXW7 | *CECR5* | Isoform 1 of Cat eye syndrome critical region protein 5 OS=Homo sapiens GN=CECR5 - [CECR5_HUMAN] | 0.907 |
| P19387 | *POLR2C* | DNA-directed RNA polymerase II subunit RPB3 OS=Homo sapiens GN=POLR2C PE=1 SV=2 - [RPB3_HUMAN] | 1.041 |
| A6NHR9 | *SMCHD1* | Structural maintenance of chromosomes flexible hinge domain-containing protein 1 OS=Homo sapiens GN=SMCHD1 PE=1 SV=2 - [SMHD1_HUMAN] | 1.149 |
| Q9UBE0 | *SAE1* | SUMO-activating enzyme subunit 1 OS=Homo sapiens GN=SAE1 PE=1 SV=1 - [SAE1_HUMAN] | 1.077 |
| Q9BVJ6 | *UTP14A* | Isoform 2 of U3 small nucleolar RNA-associated protein 14 homolog A OS=Homo sapiens GN=UTP14A - [UT14A_HUMAN] | 0.932 |
| Q9UGI8 | *TES* | Isoform 2 of Testin OS=Homo sapiens GN=TES - [TES_HUMAN] | 1.201 |
| P68036 | *UBE2L3* | Ubiquitin-conjugating enzyme E2 L3 OS=Homo sapiens GN=UBE2L3 PE=1 SV=1 - [UB2L3_HUMAN] | 1.121 |
| Q8WXF1 | *PSPC1* | Isoform 2 of Paraspeckle component 1 OS=Homo sapiens GN=PSPC1 - [PSPC1_HUMAN] | 0.901 |
| Q05193 | *DNM1* | Isoform 3 of Dynamin-1 OS=Homo sapiens GN=DNM1 - [DYN1_HUMAN] | 1.241 |
| P50402 | *EMD* | Emerin OS=Homo sapiens GN=EMD PE=1 SV=1 - [EMD_HUMAN] | 0.955 |
| Q3KR37 | *GRAMD1B* | Isoform 3 of GRAM domain-containing protein 1B OS=Homo sapiens GN=GRAMD1B - [GRM1B_HUMAN] | 1.158 |
| O14576 | *DYNC1I1* | Isoform 3 of Cytoplasmic dynein 1 intermediate chain 1 OS=Homo sapiens GN=DYNC1I1 - [DC1I1_HUMAN] | 1.267 |
| P40855 | *PEX19* | Isoform 5 of Peroxisomal biogenesis factor 19 OS=Homo sapiens GN=PEX19 - [PEX19_HUMAN] | 1.151 |
| Q96EY1 | *DNAJA3* | DnaJ homolog subfamily A member 3, mitochondrial OS=Homo sapiens GN=DNAJA3 PE=1 SV=2 - [DNJA3_HUMAN] | 0.896 |
| Q8WUM0 | *NUP133* | Nuclear pore complex protein Nup133 OS=Homo sapiens GN=NUP133 PE=1 SV=2 - [NU133_HUMAN] | 0.938 |
| P47985 | *UQCRFS1* | Cytochrome b-c1 complex subunit Rieske, mitochondrial OS=Homo sapiens GN=UQCRFS1 PE=1 SV=2 - [UCRI_HUMAN] | 1.083 |
| Q9NQP4 | *PFDN4* | Prefoldin subunit 4 OS=Homo sapiens GN=PFDN4 PE=1 SV=1 - [PFD4_HUMAN] | 1.101 |
| P51798 | *CLCN7* | H(+)/Cl(-) exchange transporter 7 OS=Homo sapiens GN=CLCN7 PE=1 SV=2 - [CLCN7_HUMAN] | 1.234 |
| Q92752 | *TNR* | Tenascin-R OS=Homo sapiens GN=TNR PE=1 SV=3 - [TENR_HUMAN] | 0.678 |
| A6NHR9 | *SMCHD1* | Isoform 2 of Structural maintenance of chromosomes flexible hinge domain-containing protein 1 OS=Homo sapiens GN=SMCHD1 - [SMHD1_HUMAN] | 1.114 |
| Q96EP0 | *RNF31* | Isoform 3 of RING finger protein 31 OS=Homo sapiens GN=RNF31 - [RNF31_HUMAN] | 0.920 |
| Q8TBA6 | *GOLGA5* | Isoform 2 of Golgin subfamily A member 5 OS=Homo sapiens GN=GOLGA5 - [GOGA5_HUMAN] | 1.088 |
| P61956 | *SUMO2* | Small ubiquitin-related modifier 2 OS=Homo sapiens GN=SUMO2 PE=1 SV=2 - [SUMO2_HUMAN] | 1.210 |
| Q9HAB8 | *PPCS* | Phosphopantothenate--cysteine ligase OS=Homo sapiens GN=PPCS PE=1 SV=2 - [PPCS_HUMAN] | 1.096 |
| Q99996 | *AKAP9* | Isoform 5 of A-kinase anchor protein 9 OS=Homo sapiens GN=AKAP9 - [AKAP9_HUMAN] | 0.943 |
| Q99623 | *PHB2* | Prohibitin-2 OS=Homo sapiens GN=PHB2 PE=1 SV=2 - [PHB2_HUMAN] | 0.906 |
| P61020 | *RAB5B* | Ras-related protein Rab-5B OS=Homo sapiens GN=RAB5B PE=1 SV=1 - [RAB5B_HUMAN] | 0.834 |
| O75688 | *PPM1B* | Isoform Beta-2 of Protein phosphatase 1B OS=Homo sapiens GN=PPM1B - [PPM1B_HUMAN] | 1.043 |
| O95391 | *SLU7* | Pre-mRNA-splicing factor SLU7 OS=Homo sapiens GN=SLU7 PE=1 SV=2 - [SLU7_HUMAN] | 1.066 |
| P31946 | *YWHAB* | Isoform Short of 14-3-3 protein beta/alpha OS=Homo sapiens GN=YWHAB - [1433B_HUMAN] | 1.061 |
| Q9BQ39 | *DDX50* | ATP-dependent RNA helicase DDX50 OS=Homo sapiens GN=DDX50 PE=1 SV=1 - [DDX50_HUMAN] | 0.924 |
| Q8WWY3 | *PRPF31* | Isoform 2 of U4/U6 small nuclear ribonucleoprotein Prp31 OS=Homo sapiens GN=PRPF31 - [PRP31_HUMAN] | 0.952 |
| Q9BZQ8 | *FAM129A* | Protein Niban OS=Homo sapiens GN=FAM129A PE=1 SV=1 - [NIBAN_HUMAN] | 0.761 |
| P35658 | *NUP214* | Isoform 2 of Nuclear pore complex protein Nup214 OS=Homo sapiens GN=NUP214 - [NU214_HUMAN] | 0.927 |
| Q16656 | *NRF1* | Isoform Short of Nuclear respiratory factor 1 OS=Homo sapiens GN=NRF1 - [NRF1_HUMAN] | 1.088 |
| O95757 | *HSPA4L* | Heat shock 70 kDa protein 4L OS=Homo sapiens GN=HSPA4L PE=1 SV=3 - [HS74L_HUMAN] | 1.425 |
| Q9H6A0 | *DENND2D* | Isoform 2 of DENN domain-containing protein 2D OS=Homo sapiens GN=DENND2D - [DEN2D_HUMAN] | 0.837 |
| P36543 | *ATP6V1E1* | V-type proton ATPase subunit E 1 OS=Homo sapiens GN=ATP6V1E1 PE=1 SV=1 - [VATE1_HUMAN] | 0.941 |
| Q14498 | *RBM39* | Isoform 2 of RNA-binding protein 39 OS=Homo sapiens GN=RBM39 - [RBM39_HUMAN] | 0.933 |
| Q8WU79 | *SMAP2* | Isoform 2 of Stromal membrane-associated protein 2 OS=Homo sapiens GN=SMAP2 - [SMAP2_HUMAN] | 1.131 |
| Q8N6N7 | *ACBD7* | Acyl-CoA-binding domain-containing protein 7 OS=Homo sapiens GN=ACBD7 PE=1 SV=1 - [ACBD7_HUMAN] | 1.193 |
| P60520 | *GABARAPL2* | Gamma-aminobutyric acid receptor-associated protein-like 2 OS=Homo sapiens GN=GABARAPL2 PE=1 SV=1 - [GBRL2_HUMAN] | 1.330 |
| Q99460 | *PSMD1* | Isoform 2 of 26S proteasome non-ATPase regulatory subunit 1 OS=Homo sapiens GN=PSMD1 - [PSMD1_HUMAN] | 0.955 |
| O43678 | *NDUFA2* | NADH dehydrogenase [ubiquinone] 1 alpha subcomplex subunit 2 OS=Homo sapiens GN=NDUFA2 PE=1 SV=3 - [NDUA2_HUMAN] | 0.909 |
| Q14254 | *FLOT2* | Flotillin-2 OS=Homo sapiens GN=FLOT2 PE=1 SV=2 - [FLOT2_HUMAN] | 1.189 |
| P98088 | *#N/A* | Mucin-5AC (Fragments) OS=Homo sapiens GN=MUC5AC PE=1 SV=3 - [MUC5A_HUMAN] | 2.865 |
| Q9HAV0 | *GNB4* | Guanine nucleotide-binding protein subunit beta-4 OS=Homo sapiens GN=GNB4 PE=1 SV=3 - [GBB4_HUMAN] | 0.875 |
| Q9Y2L9 | *LRCH1* | Isoform 2 of Leucine-rich repeat and calponin homology domain-containing protein 1 OS=Homo sapiens GN=LRCH1 - [LRCH1_HUMAN] | 1.172 |
| Q92844 | *TANK* | Isoform Short of TRAF family member-associated NF-kappa-B activator OS=Homo sapiens GN=TANK - [TANK_HUMAN] | 1.100 |
| P62873 | *GNB1* | Guanine nucleotide-binding protein G(I)/G(S)/G(T) subunit beta-1 OS=Homo sapiens GN=GNB1 PE=1 SV=3 - [GBB1_HUMAN] | 0.891 |
| P17568 | *NDUFB7* | NADH dehydrogenase [ubiquinone] 1 beta subcomplex subunit 7 OS=Homo sapiens GN=NDUFB7 PE=1 SV=4 - [NDUB7_HUMAN] | 0.940 |
| Q14257 | *RCN2* | Reticulocalbin-2 OS=Homo sapiens GN=RCN2 PE=1 SV=1 - [RCN2_HUMAN] | 1.139 |
| Q92665 | *MRPS31* | 28S ribosomal protein S31, mitochondrial OS=Homo sapiens GN=MRPS31 PE=1 SV=3 - [RT31_HUMAN] | 0.953 |
| Q9P2M7 | *CGN* | Cingulin OS=Homo sapiens GN=CGN PE=1 SV=2 - [CING_HUMAN] | 1.636 |
| Q7Z4I7 | *LIMS2* | Isoform 3 of LIM and senescent cell antigen-like-containing domain protein 2 OS=Homo sapiens GN=LIMS2 - [LIMS2_HUMAN] | 0.799 |
| Q9UPT8 | *ZC3H4* | Zinc finger CCCH domain-containing protein 4 OS=Homo sapiens GN=ZC3H4 PE=1 SV=3 - [ZC3H4_HUMAN] | 1.059 |
| P12110 | *COL6A2* | Collagen alpha-2(VI) chain OS=Homo sapiens GN=COL6A2 PE=1 SV=4 - [CO6A2_HUMAN] | 0.759 |
| Q8WXI9 | *GATAD2B* | Transcriptional repressor p66-beta OS=Homo sapiens GN=GATAD2B PE=1 SV=1 - [P66B_HUMAN] | 0.941 |
| P53992 | *SEC24C* | Protein transport protein Sec24C OS=Homo sapiens GN=SEC24C PE=1 SV=3 - [SC24C_HUMAN] | 1.081 |
| P53618 | *COPB1* | Coatomer subunit beta OS=Homo sapiens GN=COPB1 PE=1 SV=3 - [COPB_HUMAN] | 1.116 |
| Q9H832 | *UBE2Z* | Isoform 2 of Ubiquitin-conjugating enzyme E2 Z OS=Homo sapiens GN=UBE2Z - [UBE2Z_HUMAN] | 1.073 |
| P09497 | *CLTB* | Isoform Non-brain of Clathrin light chain B OS=Homo sapiens GN=CLTB - [CLCB_HUMAN] | 1.098 |
| P55263 | *ADK* | Isoform Short of Adenosine kinase OS=Homo sapiens GN=ADK - [ADK_HUMAN] | 0.942 |
| P54819 | *AK2* | Isoform 4 of Adenylate kinase 2, mitochondrial OS=Homo sapiens GN=AK2 - [KAD2_HUMAN] | 0.773 |
| P42285 | *SKIV2L2* | Superkiller viralicidic activity 2-like 2 OS=Homo sapiens GN=SKIV2L2 PE=1 SV=3 - [SK2L2_HUMAN] | 1.061 |
| Q9NVD7 | *#N/A* | Alpha-parvin OS=Homo sapiens GN=PARVA PE=1 SV=1 - [PARVA_HUMAN] | 0.882 |
| Q9BTC0 | *DIDO1* | Death-inducer obliterator 1 OS=Homo sapiens GN=DIDO1 PE=1 SV=5 - [DIDO1_HUMAN] | 0.905 |
| P11172 | *UMPS* | Uridine 5'-monophosphate synthase OS=Homo sapiens GN=UMPS PE=1 SV=1 - [UMPS_HUMAN] | 0.919 |
| P98175 | *RBM10* | Isoform 4 of RNA-binding protein 10 OS=Homo sapiens GN=RBM10 - [RBM10_HUMAN] | 1.320 |
| P51668 | *UBE2D1* | Ubiquitin-conjugating enzyme E2 D1 OS=Homo sapiens GN=UBE2D1 PE=1 SV=1 - [UB2D1_HUMAN] | 1.137 |
| Q9Y6K5 | *OAS3* | 2'-5'-oligoadenylate synthase 3 OS=Homo sapiens GN=OAS3 PE=1 SV=3 - [OAS3_HUMAN] | 1.587 |
| Q9UPN3 | *MACF1* | Isoform 3 of Microtubule-actin cross-linking factor 1, isoforms 1/2/3/5 OS=Homo sapiens GN=MACF1 - [MACF1_HUMAN] | 1.126 |
| Q8N490 | *PNKD* | Isoform 2 of Probable hydrolase PNKD OS=Homo sapiens GN=PNKD - [PNKD_HUMAN] | 0.907 |
| O95502 | *NPTXR* | Neuronal pentraxin receptor OS=Homo sapiens GN=NPTXR PE=2 SV=2 - [NPTXR_HUMAN] | 0.796 |
| Q9UKV5 | *AMFR* | E3 ubiquitin-protein ligase AMFR OS=Homo sapiens GN=AMFR PE=1 SV=2 - [AMFR2_HUMAN] | 1.092 |
| Q9NPL8 | *TIMMDC1* | Translocase of inner mitochondrial membrane domain-containing protein 1 OS=Homo sapiens GN=TIMMDC1 PE=1 SV=2 - [TIDC1_HUMAN] | 0.905 |
| Q9NV35 | *NUDT15* | Probable 7,8-dihydro-8-oxoguanine triphosphatase NUDT15 OS=Homo sapiens GN=NUDT15 PE=1 SV=1 - [NUD15_HUMAN] | 0.859 |
| Q8IZP9 | *GPR64* | Isoform 9 of G-protein coupled receptor 64 OS=Homo sapiens GN=GPR64 - [GPR64_HUMAN] | 0.789 |
| P23919 | *DTYMK* | Thymidylate kinase OS=Homo sapiens GN=DTYMK PE=1 SV=4 - [KTHY_HUMAN] | 1.132 |
| P00488 | *F13A1* | Coagulation factor XIII A chain OS=Homo sapiens GN=F13A1 PE=1 SV=4 - [F13A_HUMAN] | 0.640 |
| P12109 | *COL6A1* | Collagen alpha-1(VI) chain OS=Homo sapiens GN=COL6A1 PE=1 SV=3 - [CO6A1_HUMAN] | 0.765 |
| Q96EB1 | *ELP4* | Elongator complex protein 4 OS=Homo sapiens GN=ELP4 PE=1 SV=2 - [ELP4_HUMAN] | 0.931 |
| Q9NRZ5 | *AGPAT4* | 1-acyl-sn-glycerol-3-phosphate acyltransferase delta OS=Homo sapiens GN=AGPAT4 PE=1 SV=1 - [PLCD_HUMAN] | 1.171 |
| Q9UPZ6 | *THSD7A* | Thrombospondin type-1 domain-containing protein 7A OS=Homo sapiens GN=THSD7A PE=1 SV=4 - [THS7A_HUMAN] | 0.876 |
| P83111 | *LACTB* | Serine beta-lactamase-like protein LACTB, mitochondrial OS=Homo sapiens GN=LACTB PE=1 SV=2 - [LACTB_HUMAN] | 0.880 |
| Q9H7B2 | *RPF2* | Ribosome production factor 2 homolog OS=Homo sapiens GN=RPF2 PE=1 SV=2 - [RPF2_HUMAN] | 1.090 |
| Q9H1E3 | *NUCKS1* | Nuclear ubiquitous casein and cyclin-dependent kinases substrate OS=Homo sapiens GN=NUCKS1 PE=1 SV=1 - [NUCKS_HUMAN] | 0.913 |
| Q9BU89 | *DOHH* | Deoxyhypusine hydroxylase OS=Homo sapiens GN=DOHH PE=1 SV=1 - [DOHH_HUMAN] | 0.898 |
| P00558 | *PGK1* | Phosphoglycerate kinase 1 OS=Homo sapiens GN=PGK1 PE=1 SV=3 - [PGK1_HUMAN] | 1.128 |
| Q15843 | *NEDD8* | NEDD8 OS=Homo sapiens GN=NEDD8 PE=1 SV=1 - [NEDD8_HUMAN] | 0.909 |
| P52209 | *PGD* | 6-phosphogluconate dehydrogenase, decarboxylating OS=Homo sapiens GN=PGD PE=1 SV=3 - [6PGD_HUMAN] | 1.225 |
| Q9UBI9 | *HECA* | Headcase protein homolog OS=Homo sapiens GN=HECA PE=1 SV=1 - [HDC_HUMAN] | 0.882 |
| Q96CS3 | *FAF2* | FAS-associated factor 2 OS=Homo sapiens GN=FAF2 PE=1 SV=2 - [FAF2_HUMAN] | 1.114 |
| Q9H019 | *MTFR1L* | Isoform 3 of Protein FAM54B OS=Homo sapiens GN=FAM54B - [FA54B_HUMAN] | 0.901 |
| O75334 | *PPFIA2* | Liprin-alpha-2 OS=Homo sapiens GN=PPFIA2 PE=1 SV=1 - [LIPA2_HUMAN] | 0.757 |
| Q6P1M3 | *LLGL2* | Isoform A of Lethal(2) giant larvae protein homolog 2 OS=Homo sapiens GN=LLGL2 - [L2GL2_HUMAN] | 1.238 |
| Q9NV79 | *PCMTD2* | Isoform 2 of Protein-L-isoaspartate O-methyltransferase domain-containing protein 2 OS=Homo sapiens GN=PCMTD2 - [PCMD2_HUMAN] | 1.105 |
| Q9Y512 | *SAMM50* | Sorting and assembly machinery component 50 homolog OS=Homo sapiens GN=SAMM50 PE=1 SV=3 - [SAM50_HUMAN] | 0.895 |
| P11279 | *LAMP1* | Lysosome-associated membrane glycoprotein 1 OS=Homo sapiens GN=LAMP1 PE=1 SV=3 - [LAMP1_HUMAN] | 0.793 |
| P50150 | *GNG4* | Guanine nucleotide-binding protein G(I)/G(S)/G(O) subunit gamma-4 OS=Homo sapiens GN=GNG4 PE=1 SV=1 - [GBG4_HUMAN] | 1.176 |
| Q9GZQ3 | *COMMD5* | COMM domain-containing protein 5 OS=Homo sapiens GN=COMMD5 PE=1 SV=1 - [COMD5_HUMAN] | 0.939 |
| P09455 | *RBP1* | Retinol-binding protein 1 OS=Homo sapiens GN=RBP1 PE=1 SV=2 - [RET1_HUMAN] | 0.832 |
| Q66PJ3 | *ARL6IP4* | Isoform 5 of ADP-ribosylation factor-like protein 6-interacting protein 4 OS=Homo sapiens GN=ARL6IP4 - [AR6P4_HUMAN] | 0.893 |
| P15428 | *HPGD* | 15-hydroxyprostaglandin dehydrogenase [NAD+] OS=Homo sapiens GN=HPGD PE=1 SV=1 - [PGDH_HUMAN] | 0.627 |
| Q9P1Y6 | *PHRF1* | Isoform 2 of PHD and RING finger domain-containing protein 1 OS=Homo sapiens GN=PHRF1 - [PHRF1_HUMAN] | 1.096 |
| Q99988 | *GDF15* | Growth/differentiation factor 15 OS=Homo sapiens GN=GDF15 PE=1 SV=3 - [GDF15_HUMAN] | 2.995 |
| Q99633 | *PRPF18* | Pre-mRNA-splicing factor 18 OS=Homo sapiens GN=PRPF18 PE=1 SV=1 - [PRP18_HUMAN] | 1.049 |
| Q96KM6 | *ZNF512B* | Zinc finger protein 512B OS=Homo sapiens GN=ZNF512B PE=1 SV=1 - [Z512B_HUMAN] | 1.107 |
| Q8N568 | *DCLK2* | Isoform 2 of Serine/threonine-protein kinase DCLK2 OS=Homo sapiens GN=DCLK2 - [DCLK2_HUMAN] | 1.218 |
| O43866 | *CD5L* | CD5 antigen-like OS=Homo sapiens GN=CD5L PE=1 SV=1 - [CD5L_HUMAN] | 0.830 |
| Q9NP72 | *RAB18* | Ras-related protein Rab-18 OS=Homo sapiens GN=RAB18 PE=1 SV=1 - [RAB18_HUMAN] | 1.065 |
| P08195 | *SLC3A2* | Isoform 2 of 4F2 cell-surface antigen heavy chain OS=Homo sapiens GN=SLC3A2 - [4F2_HUMAN] | 0.903 |
| Q96B45 | *C10orf32* | UPF0693 protein C10orf32 OS=Homo sapiens GN=C10orf32 PE=1 SV=1 - [CJ032_HUMAN] | 1.136 |
| O15294 | *OGT* | Isoform 1 of UDP-N-acetylglucosamine--peptide N-acetylglucosaminyltransferase 110 kDa subunit OS=Homo sapiens GN=OGT - [OGT1_HUMAN] | 1.039 |
| Q15714 | *TSC22D1* | Isoform 2 of TSC22 domain family protein 1 OS=Homo sapiens GN=TSC22D1 - [T22D1_HUMAN] | 0.915 |
| P00390 | *GSR* | Isoform Cytoplasmic of Glutathione reductase, mitochondrial OS=Homo sapiens GN=GSR - [GSHR_HUMAN] | 1.114 |
| Q9Y2G5 | *POFUT2* | Isoform B of GDP-fucose protein O-fucosyltransferase 2 OS=Homo sapiens GN=POFUT2 - [OFUT2_HUMAN] | 1.116 |
| Q765P7 | *MTSS1L* | MTSS1-like protein OS=Homo sapiens GN=MTSS1L PE=1 SV=1 - [MTSSL_HUMAN] | 1.264 |
| P19784 | *CSNK2A2* | Casein kinase II subunit alpha' OS=Homo sapiens GN=CSNK2A2 PE=1 SV=1 - [CSK22_HUMAN] | 1.084 |
| P54886 | *ALDH18A1* | Isoform Short of Delta-1-pyrroline-5-carboxylate synthase OS=Homo sapiens GN=ALDH18A1 - [P5CS_HUMAN] | 0.858 |
| Q9NRN5 | *OLFML3* | Isoform 2 of Olfactomedin-like protein 3 OS=Homo sapiens GN=OLFML3 - [OLFL3_HUMAN] | 1.252 |
| Q5VZ89 | *#N/A* | Isoform 6 of DENN domain-containing protein 4C OS=Homo sapiens GN=DENND4C - [DEN4C_HUMAN] | 0.937 |
| Q6WCQ1 | *MPRIP* | Myosin phosphatase Rho-interacting protein OS=Homo sapiens GN=MPRIP PE=1 SV=3 - [MPRIP_HUMAN] | 0.944 |
| Q9BSJ8 | *ESYT1* | Extended synaptotagmin-1 OS=Homo sapiens GN=ESYT1 PE=1 SV=1 - [ESYT1_HUMAN] | 0.889 |
| Q7Z6M4 | *MTERF4* | mTERF domain-containing protein 2 OS=Homo sapiens GN=MTERFD2 PE=1 SV=3 - [MTER2_HUMAN] | 0.892 |
| P62906 | *RPL10A* | 60S ribosomal protein L10a OS=Homo sapiens GN=RPL10A PE=1 SV=2 - [RL10A_HUMAN] | 1.054 |
| Q7L0J3 | *SV2A* | Isoform 2 of Synaptic vesicle glycoprotein 2A OS=Homo sapiens GN=SV2A - [SV2A_HUMAN] | 0.799 |
| Q7L8C5 | *SYT13* | Synaptotagmin-13 OS=Homo sapiens GN=SYT13 PE=1 SV=1 - [SYT13_HUMAN] | 0.866 |
| P00491 | *PNP* | Purine nucleoside phosphorylase OS=Homo sapiens GN=PNP PE=1 SV=2 - [PNPH_HUMAN] | 1.190 |
| Q6UXI9 | *NPNT* | Isoform 2 of Nephronectin OS=Homo sapiens GN=NPNT - [NPNT_HUMAN] | 0.632 |
| P56385 | *ATP5I* | ATP synthase subunit e, mitochondrial OS=Homo sapiens GN=ATP5I PE=1 SV=2 - [ATP5I_HUMAN] | 0.905 |
| P53990 | *IST1* | Isoform 2 of IST1 homolog OS=Homo sapiens GN=IST1 - [IST1_HUMAN] | 1.108 |
| P53803 | *POLR2K* | DNA-directed RNA polymerases I, II, and III subunit RPABC4 OS=Homo sapiens GN=POLR2K PE=2 SV=1 - [RPAB4_HUMAN] | 1.090 |
| Q6Q0C0 | *TRAF7* | Isoform 2 of E3 ubiquitin-protein ligase TRAF7 OS=Homo sapiens GN=TRAF7 - [TRAF7_HUMAN] | 0.911 |
| Q8N0X4 | *CLYBL* | Isoform 2 of Citrate lyase subunit beta-like protein, mitochondrial OS=Homo sapiens GN=CLYBL - [CLYBL_HUMAN] | 0.785 |
| P22087 | *FBL* | rRNA 2'-O-methyltransferase fibrillarin OS=Homo sapiens GN=FBL PE=1 SV=2 - [FBRL_HUMAN] | 1.073 |
| P57081 | *WDR4* | Isoform 3 of tRNA (guanine-N(7)-)-methyltransferase subunit WDR4 OS=Homo sapiens GN=WDR4 - [WDR4_HUMAN] | 1.136 |
| Q8WXG6 | *MADD* | Isoform 6 of MAP kinase-activating death domain protein OS=Homo sapiens GN=MADD - [MADD_HUMAN] | 1.106 |
| Q9Y4X5 | *ARIH1* | E3 ubiquitin-protein ligase ARIH1 OS=Homo sapiens GN=ARIH1 PE=1 SV=2 - [ARI1_HUMAN] | 1.077 |
| Q86WG5 | *SBF2* | Myotubularin-related protein 13 OS=Homo sapiens GN=SBF2 PE=1 SV=1 - [MTMRD_HUMAN] | 0.925 |
| P09543 | *CNP* | Isoform CNPI of 2',3'-cyclic-nucleotide 3'-phosphodiesterase OS=Homo sapiens GN=CNP - [CN37_HUMAN] | 0.928 |
| Q15008 | *PSMD6* | 26S proteasome non-ATPase regulatory subunit 6 OS=Homo sapiens GN=PSMD6 PE=1 SV=1 - [PSMD6_HUMAN] | 1.047 |
| P15531 | *NME1* | Nucleoside diphosphate kinase A OS=Homo sapiens GN=NME1 PE=1 SV=1 - [NDKA_HUMAN] | 0.934 |
| P46020 | *PHKA1* | Isoform 2 of Phosphorylase b kinase regulatory subunit alpha, skeletal muscle isoform OS=Homo sapiens GN=PHKA1 - [KPB1_HUMAN] | 0.919 |
| Q9BXS5 | *AP1M1* | AP-1 complex subunit mu-1 OS=Homo sapiens GN=AP1M1 PE=1 SV=3 - [AP1M1_HUMAN] | 1.076 |
| O43172 | *PRPF4* | Isoform 2 of U4/U6 small nuclear ribonucleoprotein Prp4 OS=Homo sapiens GN=PRPF4 - [PRP4_HUMAN] | 1.059 |
| O15145 | *ARPC3* | Actin-related protein 2/3 complex subunit 3 OS=Homo sapiens GN=ARPC3 PE=1 SV=3 - [ARPC3_HUMAN] | 1.089 |
| P60660 | *MYL6* | Myosin light polypeptide 6 OS=Homo sapiens GN=MYL6 PE=1 SV=2 - [MYL6_HUMAN] | 1.131 |
| Q0VDF9 | *HSPA14* | Heat shock 70 kDa protein 14 OS=Homo sapiens GN=HSPA14 PE=1 SV=1 - [HSP7E_HUMAN] | 1.085 |
| Q9UHR4 | *BAIAP2L1* | Brain-specific angiogenesis inhibitor 1-associated protein 2-like protein 1 OS=Homo sapiens GN=BAIAP2L1 PE=1 SV=2 - [BI2L1_HUMAN] | 0.787 |
| Q9NYB0 | *TERF2IP* | Telomeric repeat-binding factor 2-interacting protein 1 OS=Homo sapiens GN=TERF2IP PE=1 SV=1 - [TE2IP_HUMAN] | 0.879 |
| Q16777 | *HIST2H2AC* | Histone H2A type 2-C OS=Homo sapiens GN=HIST2H2AC PE=1 SV=4 - [H2A2C_HUMAN] | 1.427 |
| Q13418 | *ILK* | Integrin-linked protein kinase OS=Homo sapiens GN=ILK PE=1 SV=2 - [ILK_HUMAN] | 0.895 |
| Q658Y4 | *FAM91A1* | Protein FAM91A1 OS=Homo sapiens GN=FAM91A1 PE=1 SV=3 - [F91A1_HUMAN] | 0.904 |
| Q14980 | *NUMA1* | Isoform 2 of Nuclear mitotic apparatus protein 1 OS=Homo sapiens GN=NUMA1 - [NUMA1_HUMAN] | 1.128 |
| P51553 | *IDH3G* | Isocitrate dehydrogenase [NAD] subunit gamma, mitochondrial OS=Homo sapiens GN=IDH3G PE=1 SV=1 - [IDH3G_HUMAN] | 0.923 |
| P13473 | *LAMP2* | Lysosome-associated membrane glycoprotein 2 OS=Homo sapiens GN=LAMP2 PE=1 SV=2 - [LAMP2_HUMAN] | 0.892 |
| Q9BY43 | *CHMP4A* | Charged multivesicular body protein 4a OS=Homo sapiens GN=CHMP4A PE=1 SV=3 - [CHM4A_HUMAN] | 1.145 |
| Q86UE4 | *MTDH* | Protein LYRIC OS=Homo sapiens GN=MTDH PE=1 SV=2 - [LYRIC_HUMAN] | 0.921 |
| Q99733 | *NAP1L4* | Nucleosome assembly protein 1-like 4 OS=Homo sapiens GN=NAP1L4 PE=1 SV=1 - [NP1L4_HUMAN] | 1.091 |
| Q8IX12 | *CCAR1* | Isoform 2 of Cell division cycle and apoptosis regulator protein 1 OS=Homo sapiens GN=CCAR1 - [CCAR1_HUMAN] | 0.959 |
| P35475 | *IDUA* | Alpha-L-iduronidase OS=Homo sapiens GN=IDUA PE=1 SV=2 - [IDUA_HUMAN] | 0.863 |
| O15111 | *CHUK* | Inhibitor of nuclear factor kappa-B kinase subunit alpha OS=Homo sapiens GN=CHUK PE=1 SV=2 - [IKKA_HUMAN] | 0.931 |
| O14656 | *TOR1A* | Isoform 2 of Torsin-1A OS=Homo sapiens GN=TOR1A - [TOR1A_HUMAN] | 1.096 |
| Q9Y508 | *RNF114* | Isoform 2 of RING finger protein 114 OS=Homo sapiens GN=RNF114 - [RN114_HUMAN] | 0.919 |
| P62633 | *CNBP* | Isoform 3 of Cellular nucleic acid-binding protein OS=Homo sapiens GN=CNBP - [CNBP_HUMAN] | 0.904 |
| O43432 | *EIF4G3* | Eukaryotic translation initiation factor 4 gamma 3 OS=Homo sapiens GN=EIF4G3 PE=1 SV=2 - [IF4G3_HUMAN] | 1.128 |
| P29279 | *CTGF* | Isoform 2 of Connective tissue growth factor OS=Homo sapiens GN=CTGF - [CTGF_HUMAN] | 1.866 |
| Q9NUV9 | *GIMAP4* | GTPase IMAP family member 4 OS=Homo sapiens GN=GIMAP4 PE=1 SV=1 - [GIMA4_HUMAN] | 0.810 |
| P01033 | *TIMP1* | Metalloproteinase inhibitor 1 OS=Homo sapiens GN=TIMP1 PE=1 SV=1 - [TIMP1_HUMAN] | 0.741 |
| Q8WUY3 | *PRUNE2* | Isoform 4 of Protein prune homolog 2 OS=Homo sapiens GN=PRUNE2 - [PRUN2_HUMAN] | 0.921 |
| Q9H4A6 | *GOLPH3* | Golgi phosphoprotein 3 OS=Homo sapiens GN=GOLPH3 PE=1 SV=1 - [GOLP3_HUMAN] | 1.200 |
| P60903 | *S100A10* | Protein S100-A10 OS=Homo sapiens GN=S100A10 PE=1 SV=2 - [S10AA_HUMAN] | 0.641 |
| Q6UXV4 | *APOOL* | Apolipoprotein O-like OS=Homo sapiens GN=APOOL PE=1 SV=1 - [APOOL_HUMAN] | 0.920 |
| P02545 | *LMNA* | Isoform C of Prelamin-A/C OS=Homo sapiens GN=LMNA - [LMNA_HUMAN] | 0.903 |
| Q99584 | *S100A13* | Protein S100-A13 OS=Homo sapiens GN=S100A13 PE=1 SV=1 - [S10AD_HUMAN] | 0.862 |
| P63151 | *PPP2R2A* | Serine/threonine-protein phosphatase 2A 55 kDa regulatory subunit B alpha isoform OS=Homo sapiens GN=PPP2R2A PE=1 SV=1 - [2ABA_HUMAN] | 1.113 |
| P01743 | *#N/A* | Ig heavy chain V-I region HG3 OS=Homo sapiens PE=4 SV=1 - [HV102_HUMAN] | 0.849 |
| Q9GZT8 | *NIF3L1* | Isoform 2 of NIF3-like protein 1 OS=Homo sapiens GN=NIF3L1 - [NIF3L_HUMAN] | 1.042 |
| P05114 | *HMGN1* | Non-histone chromosomal protein HMG-14 OS=Homo sapiens GN=HMGN1 PE=1 SV=3 - [HMGN1_HUMAN] | 1.083 |
| Q9Y679 | *AUP1* | Isoform 3 of Ancient ubiquitous protein 1 OS=Homo sapiens GN=AUP1 - [AUP1_HUMAN] | 1.108 |
| P11277 | *SPTB* | Isoform 2 of Spectrin beta chain, erythrocyte OS=Homo sapiens GN=SPTB - [SPTB1_HUMAN] | 0.742 |
| Q9C0B1 | *FTO* | Alpha-ketoglutarate-dependent dioxygenase FTO OS=Homo sapiens GN=FTO PE=1 SV=3 - [FTO_HUMAN] | 0.888 |
| Q9P0M9 | *MRPL27* | 39S ribosomal protein L27, mitochondrial OS=Homo sapiens GN=MRPL27 PE=1 SV=1 - [RM27_HUMAN] | 0.853 |
| Q9Y2A7 | *NCKAP1* | Nck-associated protein 1 OS=Homo sapiens GN=NCKAP1 PE=1 SV=1 - [NCKP1_HUMAN] | 0.963 |
| Q9Y696 | *CLIC4* | Chloride intracellular channel protein 4 OS=Homo sapiens GN=CLIC4 PE=1 SV=4 - [CLIC4_HUMAN] | 1.164 |
| Q96SB3 | *#N/A* | Neurabin-2 OS=Homo sapiens GN=PPP1R9B PE=1 SV=2 - [NEB2_HUMAN] | 0.926 |
| Q86YB8 | *ERO1LB* | ERO1-like protein beta OS=Homo sapiens GN=ERO1LB PE=1 SV=2 - [ERO1B_HUMAN] | 1.172 |
| P51665 | *PSMD7* | 26S proteasome non-ATPase regulatory subunit 7 OS=Homo sapiens GN=PSMD7 PE=1 SV=2 - [PSD7_HUMAN] | 1.058 |
| Q6PJI9 | *WDR59* | Isoform 4 of WD repeat-containing protein 59 OS=Homo sapiens GN=WDR59 - [WDR59_HUMAN] | 1.124 |
| Q15155 | *NOMO1* | Nodal modulator 1 OS=Homo sapiens GN=NOMO1 PE=1 SV=5 - [NOMO1_HUMAN] | 1.127 |
| O95295 | *SNAPIN* | SNARE-associated protein Snapin OS=Homo sapiens GN=SNAPIN PE=1 SV=1 - [SNAPN_HUMAN] | 1.135 |
| Q8TAE8 | *GADD45GIP1* | Growth arrest and DNA damage-inducible proteins-interacting protein 1 OS=Homo sapiens GN=GADD45GIP1 PE=1 SV=1 - [G45IP_HUMAN] | 0.940 |
| Q9NZB2 | *FAM120A* | Isoform D of Constitutive coactivator of PPAR-gamma-like protein 1 OS=Homo sapiens GN=FAM120A - [F120A_HUMAN] | 0.946 |
| Q6PGP7 | *TTC37* | Tetratricopeptide repeat protein 37 OS=Homo sapiens GN=TTC37 PE=1 SV=1 - [TTC37_HUMAN] | 0.923 |
| Q02880 | *TOP2B* | Isoform Beta-1 of DNA topoisomerase 2-beta OS=Homo sapiens GN=TOP2B - [TOP2B_HUMAN] | 0.898 |
| P06865 | *HEXA* | Beta-hexosaminidase subunit alpha OS=Homo sapiens GN=HEXA PE=1 SV=2 - [HEXA_HUMAN] | 1.166 |
| Q9UN86 | *G3BP2* | Isoform B of Ras GTPase-activating protein-binding protein 2 OS=Homo sapiens GN=G3BP2 - [G3BP2_HUMAN] | 1.071 |
| P19086 | *GNAZ* | Guanine nucleotide-binding protein G(z) subunit alpha OS=Homo sapiens GN=GNAZ PE=2 SV=3 - [GNAZ_HUMAN] | 0.855 |
| P49406 | *MRPL19* | 39S ribosomal protein L19, mitochondrial OS=Homo sapiens GN=MRPL19 PE=1 SV=2 - [RM19_HUMAN] | 0.939 |
| Q9BRX9 | *WDR83* | WD repeat domain-containing protein 83 OS=Homo sapiens GN=WDR83 PE=1 SV=1 - [WDR83_HUMAN] | 1.050 |
| O94772 | *LY6H* | Lymphocyte antigen 6H OS=Homo sapiens GN=LY6H PE=2 SV=1 - [LY6H_HUMAN] | 1.647 |
| P05204 | *HMGN2* | Non-histone chromosomal protein HMG-17 OS=Homo sapiens GN=HMGN2 PE=1 SV=3 - [HMGN2_HUMAN] | 1.186 |
| P49458 | *SRP9* | Signal recognition particle 9 kDa protein OS=Homo sapiens GN=SRP9 PE=1 SV=2 - [SRP09_HUMAN] | 0.917 |
| Q16543 | *CDC37* | Hsp90 co-chaperone Cdc37 OS=Homo sapiens GN=CDC37 PE=1 SV=1 - [CDC37_HUMAN] | 1.056 |
| Q9BZE1 | *MRPL37* | 39S ribosomal protein L37, mitochondrial OS=Homo sapiens GN=MRPL37 PE=1 SV=2 - [RM37_HUMAN] | 0.937 |
| Q9ULB1 | *NRXN1* | Neurexin-1-alpha OS=Homo sapiens GN=NRXN1 PE=2 SV=1 - [NRX1A_HUMAN] | 0.804 |
| Q7Z7E8 | *UBE2Q1* | Isoform 2 of Ubiquitin-conjugating enzyme E2 Q1 OS=Homo sapiens GN=UBE2Q1 - [UB2Q1_HUMAN] | 1.080 |
| Q9NY12 | *GAR1* | Isoform 2 of H/ACA ribonucleoprotein complex subunit 1 OS=Homo sapiens GN=GAR1 - [GAR1_HUMAN] | 0.933 |
| P48047 | *ATP5O* | ATP synthase subunit O, mitochondrial OS=Homo sapiens GN=ATP5O PE=1 SV=1 - [ATPO_HUMAN] | 0.933 |
| O43427 | *FIBP* | Isoform Short of Acidic fibroblast growth factor intracellular-binding protein OS=Homo sapiens GN=FIBP - [FIBP_HUMAN] | 0.929 |
| Q9BY50 | *SEC11C* | Signal peptidase complex catalytic subunit SEC11C OS=Homo sapiens GN=SEC11C PE=1 SV=3 - [SC11C_HUMAN] | 1.108 |
| P49427 | *CDC34* | Ubiquitin-conjugating enzyme E2 R1 OS=Homo sapiens GN=CDC34 PE=1 SV=2 - [UB2R1_HUMAN] | 1.146 |
| P33897 | *ABCD1* | ATP-binding cassette sub-family D member 1 OS=Homo sapiens GN=ABCD1 PE=1 SV=2 - [ABCD1_HUMAN] | 1.192 |
| O00170 | *AIP* | AH receptor-interacting protein OS=Homo sapiens GN=AIP PE=1 SV=2 - [AIP_HUMAN] | 1.081 |
| Q9Y6C2 | *EMILIN1* | EMILIN-1 OS=Homo sapiens GN=EMILIN1 PE=1 SV=2 - [EMIL1_HUMAN] | 1.281 |
| Q9UGV2 | *NDRG3* | Isoform 3 of Protein NDRG3 OS=Homo sapiens GN=NDRG3 - [NDRG3_HUMAN] | 1.075 |
| O60763 | *USO1* | General vesicular transport factor p115 OS=Homo sapiens GN=USO1 PE=1 SV=2 - [USO1_HUMAN] | 0.918 |
| Q9UBS9 | *SUCO* | Protein osteopotentia homolog OS=Homo sapiens GN=C1orf9 PE=2 SV=1 - [OSPT_HUMAN] | 1.140 |
| Q8IWZ3 | *ANKHD1* | Ankyrin repeat and KH domain-containing protein 1 OS=Homo sapiens GN=ANKHD1 PE=1 SV=1 - [ANKH1_HUMAN] | 0.893 |
| Q9H5X1 | *FAM96A* | MIP18 family protein FAM96A OS=Homo sapiens GN=FAM96A PE=1 SV=1 - [FA96A_HUMAN] | 1.087 |
| P30837 | *ALDH1B1* | Aldehyde dehydrogenase X, mitochondrial OS=Homo sapiens GN=ALDH1B1 PE=1 SV=3 - [AL1B1_HUMAN] | 0.791 |
| P00387 | *CYB5R3* | Isoform 2 of NADH-cytochrome b5 reductase 3 OS=Homo sapiens GN=CYB5R3 - [NB5R3_HUMAN] | 1.157 |
| O75964 | *ATP5L* | ATP synthase subunit g, mitochondrial OS=Homo sapiens GN=ATP5L PE=1 SV=3 - [ATP5L_HUMAN] | 0.908 |
| P23396 | *RPS3* | 40S ribosomal protein S3 OS=Homo sapiens GN=RPS3 PE=1 SV=2 - [RS3_HUMAN] | 1.049 |
| Q8WUA4 | *GTF3C2* | General transcription factor 3C polypeptide 2 OS=Homo sapiens GN=GTF3C2 PE=1 SV=2 - [TF3C2_HUMAN] | 1.073 |
| Q9Y4P8 | *WIPI2* | Isoform 3 of WD repeat domain phosphoinositide-interacting protein 2 OS=Homo sapiens GN=WIPI2 - [WIPI2_HUMAN] | 0.917 |
| Q8TDY2 | *RB1CC1* | Isoform 2 of RB1-inducible coiled-coil protein 1 OS=Homo sapiens GN=RB1CC1 - [RBCC1_HUMAN] | 1.083 |
| P17028 | *ZNF24* | Zinc finger protein 24 OS=Homo sapiens GN=ZNF24 PE=1 SV=4 - [ZNF24_HUMAN] | 1.086 |
| P27658 | *COL8A1* | Collagen alpha-1(VIII) chain OS=Homo sapiens GN=COL8A1 PE=1 SV=2 - [CO8A1_HUMAN] | 0.784 |
| P52943 | *CRIP2* | Cysteine-rich protein 2 OS=Homo sapiens GN=CRIP2 PE=1 SV=1 - [CRIP2_HUMAN] | 0.848 |
| Q96EK7 | *FAM120B* | Isoform 2 of Constitutive coactivator of peroxisome proliferator-activated receptor gamma OS=Homo sapiens GN=FAM120B - [F120B_HUMAN] | 0.943 |
| Q9NY47 | *CACNA2D2* | Isoform 4 of Voltage-dependent calcium channel subunit alpha-2/delta-2 OS=Homo sapiens GN=CACNA2D2 - [CA2D2_HUMAN] | 0.827 |
| Q15075 | *EEA1* | Early endosome antigen 1 OS=Homo sapiens GN=EEA1 PE=1 SV=2 - [EEA1_HUMAN] | 0.951 |
| Q96C86 | *DCPS* | Scavenger mRNA-decapping enzyme DcpS OS=Homo sapiens GN=DCPS PE=1 SV=2 - [DCPS_HUMAN] | 1.101 |
| Q7Z4H8 | *KDELC2* | Isoform 3 of KDEL motif-containing protein 2 OS=Homo sapiens GN=KDELC2 - [KDEL2_HUMAN] | 1.109 |
| O43491 | *EPB41L2* | Band 4.1-like protein 2 OS=Homo sapiens GN=EPB41L2 PE=1 SV=1 - [E41L2_HUMAN] | 0.796 |
| O75251 | *NDUFS7* | NADH dehydrogenase [ubiquinone] iron-sulfur protein 7, mitochondrial OS=Homo sapiens GN=NDUFS7 PE=1 SV=3 - [NDUS7_HUMAN] | 0.943 |
| Q9Y450 | *HBS1L* | Isoform 3 of HBS1-like protein OS=Homo sapiens GN=HBS1L - [HBS1L_HUMAN] | 0.919 |
| P82912 | *MRPS11* | Isoform 2 of 28S ribosomal protein S11, mitochondrial OS=Homo sapiens GN=MRPS11 - [RT11_HUMAN] | 0.942 |
| Q9H977 | *WDR54* | WD repeat-containing protein 54 OS=Homo sapiens GN=WDR54 PE=1 SV=1 - [WDR54_HUMAN] | 1.124 |
| P28347 | *TEAD1* | Transcriptional enhancer factor TEF-1 OS=Homo sapiens GN=TEAD1 PE=1 SV=2 - [TEAD1_HUMAN] | 0.925 |
| Q9BYT8 | *NLN* | Neurolysin, mitochondrial OS=Homo sapiens GN=NLN PE=1 SV=1 - [NEUL_HUMAN] | 1.173 |
| P19474 | *TRIM21* | E3 ubiquitin-protein ligase TRIM21 OS=Homo sapiens GN=TRIM21 PE=1 SV=1 - [RO52_HUMAN] | 1.186 |
| Q6ZNJ1 | *NBEAL2* | Isoform 2 of Neurobeachin-like protein 2 OS=Homo sapiens GN=NBEAL2 - [NBEL2_HUMAN] | 1.179 |
| P27986 | *PIK3R1* | Isoform 3 of Phosphatidylinositol 3-kinase regulatory subunit alpha OS=Homo sapiens GN=PIK3R1 - [P85A_HUMAN] | 0.906 |
| Q9Y371 | *SH3GLB1* | Endophilin-B1 OS=Homo sapiens GN=SH3GLB1 PE=1 SV=1 - [SHLB1_HUMAN] | 1.097 |
| P62841 | *RPS15* | 40S ribosomal protein S15 OS=Homo sapiens GN=RPS15 PE=1 SV=2 - [RS15_HUMAN] | 1.027 |
| P21127 | *#N/A* | Isoform SV5 of Cyclin-dependent kinase 11B OS=Homo sapiens GN=CDK11B - [CD11B_HUMAN] | 1.056 |
| P63244 | *GNB2L1* | Guanine nucleotide-binding protein subunit beta-2-like 1 OS=Homo sapiens GN=GNB2L1 PE=1 SV=3 - [GBLP_HUMAN] | 0.945 |
| P15529 | *CD46* | Isoform 3 of Membrane cofactor protein OS=Homo sapiens GN=CD46 - [MCP_HUMAN] | 0.894 |
| O43252 | *PAPSS1* | Bifunctional 3'-phosphoadenosine 5'-phosphosulfate synthase 1 OS=Homo sapiens GN=PAPSS1 PE=1 SV=2 - [PAPS1_HUMAN] | 1.094 |
| P50148 | *GNAQ* | Guanine nucleotide-binding protein G(q) subunit alpha OS=Homo sapiens GN=GNAQ PE=1 SV=4 - [GNAQ_HUMAN] | 0.918 |
| P83731 | *RPL24* | 60S ribosomal protein L24 OS=Homo sapiens GN=RPL24 PE=1 SV=1 - [RL24_HUMAN] | 1.041 |
| Q12805 | *EFEMP1* | Isoform 2 of EGF-containing fibulin-like extracellular matrix protein 1 OS=Homo sapiens GN=EFEMP1 - [FBLN3_HUMAN] | 1.223 |
| Q7KZ85 | *SUPT6H* | Transcription elongation factor SPT6 OS=Homo sapiens GN=SUPT6H PE=1 SV=2 - [SPT6H_HUMAN] | 0.962 |
| Q9NX14 | *NDUFB11* | NADH dehydrogenase [ubiquinone] 1 beta subcomplex subunit 11, mitochondrial OS=Homo sapiens GN=NDUFB11 PE=1 SV=1 - [NDUBB_HUMAN] | 0.932 |
| Q9BYV8 | *CEP41* | Isoform 3 of Centrosomal protein of 41 kDa OS=Homo sapiens GN=CEP41 - [CEP41_HUMAN] | 1.156 |
| Q9HAS0 | *C17orf75* | Protein Njmu-R1 OS=Homo sapiens GN=C17orf75 PE=1 SV=2 - [NJMU_HUMAN] | 0.921 |
| P06727 | *APOA4* | Apolipoprotein A-IV OS=Homo sapiens GN=APOA4 PE=1 SV=3 - [APOA4_HUMAN] | 0.525 |
| Q5BKZ1 | *ZNF326* | Zinc finger protein 326 OS=Homo sapiens GN=ZNF326 PE=1 SV=2 - [ZN326_HUMAN] | 1.085 |
| O43175 | *PHGDH* | D-3-phosphoglycerate dehydrogenase OS=Homo sapiens GN=PHGDH PE=1 SV=4 - [SERA_HUMAN] | 1.970 |
| Q92734 | *TFG* | Protein TFG OS=Homo sapiens GN=TFG PE=1 SV=2 - [TFG_HUMAN] | 0.918 |
| Q9GZS1 | *POLR1E* | Isoform 2 of DNA-directed RNA polymerase I subunit RPA49 OS=Homo sapiens GN=POLR1E - [RPA49_HUMAN] | 1.059 |
| Q8WVF1 | *OSCP1* | Isoform 2 of Protein OSCP1 OS=Homo sapiens GN=OSCP1 - [OSCP1_HUMAN] | 1.103 |
| P11277 | *SPTB* | Spectrin beta chain, erythrocyte OS=Homo sapiens GN=SPTB PE=1 SV=5 - [SPTB1_HUMAN] | 0.747 |
| Q8WXA9 | *SREK1* | Splicing regulatory glutamine/lysine-rich protein 1 OS=Homo sapiens GN=SREK1 PE=1 SV=1 - [SREK1_HUMAN] | 1.086 |
| O95573 | *ACSL3* | Long-chain-fatty-acid--CoA ligase 3 OS=Homo sapiens GN=ACSL3 PE=1 SV=3 - [ACSL3_HUMAN] | 0.919 |
| Q8TCD1 | *C18orf32* | UPF0729 protein C18orf32 OS=Homo sapiens GN=C18orf32 PE=2 SV=1 - [CR032_HUMAN] | 1.119 |
| P17480 | *UBTF* | Isoform UBF2 of Nucleolar transcription factor 1 OS=Homo sapiens GN=UBTF - [UBF1_HUMAN] | 1.082 |
| O00142 | *TK2* | Isoform Short of Thymidine kinase 2, mitochondrial OS=Homo sapiens GN=TK2 - [KITM_HUMAN] | 0.843 |
| Q8NE71 | *ABCF1* | Isoform 2 of ATP-binding cassette sub-family F member 1 OS=Homo sapiens GN=ABCF1 - [ABCF1_HUMAN] | 0.918 |
| O14828 | *SCAMP3* | Secretory carrier-associated membrane protein 3 OS=Homo sapiens GN=SCAMP3 PE=1 SV=3 - [SCAM3_HUMAN] | 1.122 |
| P82914 | *MRPS15* | 28S ribosomal protein S15, mitochondrial OS=Homo sapiens GN=MRPS15 PE=1 SV=1 - [RT15_HUMAN] | 0.904 |
| Q6P4E1 | *CASC4* | Isoform 2 of Protein CASC4 OS=Homo sapiens GN=CASC4 - [CASC4_HUMAN] | 0.902 |
| Q9H6Z4 | *RANBP3* | Isoform 2 of Ran-binding protein 3 OS=Homo sapiens GN=RANBP3 - [RANB3_HUMAN] | 0.946 |
| Q8N7H5 | *PAF1* | Isoform 2 of RNA polymerase II-associated factor 1 homolog OS=Homo sapiens GN=PAF1 - [PAF1_HUMAN] | 1.072 |
| P62917 | *RPL8* | 60S ribosomal protein L8 OS=Homo sapiens GN=RPL8 PE=1 SV=2 - [RL8_HUMAN] | 1.053 |
| O60783 | *MRPS14* | 28S ribosomal protein S14, mitochondrial OS=Homo sapiens GN=MRPS14 PE=1 SV=1 - [RT14_HUMAN] | 0.918 |
| Q9HBU6 | *ETNK1* | Ethanolamine kinase 1 OS=Homo sapiens GN=ETNK1 PE=1 SV=1 - [EKI1_HUMAN] | 0.943 |
| O14818 | *PSMA7* | Proteasome subunit alpha type-7 OS=Homo sapiens GN=PSMA7 PE=1 SV=1 - [PSA7_HUMAN] | 1.045 |
| O75880 | *SCO1* | Protein SCO1 homolog, mitochondrial OS=Homo sapiens GN=SCO1 PE=1 SV=1 - [SCO1_HUMAN] | 0.868 |
| O15173 | *PGRMC2* | Membrane-associated progesterone receptor component 2 OS=Homo sapiens GN=PGRMC2 PE=1 SV=1 - [PGRC2_HUMAN] | 1.075 |
| P01613 | *#N/A* | Ig kappa chain V-I region Ni OS=Homo sapiens PE=1 SV=1 - [KV121_HUMAN] | 0.902 |
| Q6NXG1 | *ESRP1* | Isoform 2 of Epithelial splicing regulatory protein 1 OS=Homo sapiens GN=ESRP1 - [ESRP1_HUMAN] | 0.881 |
| O94906 | *PRPF6* | Isoform 2 of Pre-mRNA-processing factor 6 OS=Homo sapiens GN=PRPF6 - [PRP6_HUMAN] | 1.074 |
| Q01433 | *AMPD2* | Isoform Ex1A-2-3 of AMP deaminase 2 OS=Homo sapiens GN=AMPD2 - [AMPD2_HUMAN] | 0.895 |
| Q9P2E9 | *RRBP1* | Isoform 2 of Ribosome-binding protein 1 OS=Homo sapiens GN=RRBP1 - [RRBP1_HUMAN] | 0.886 |
| Q9BUH6 | *C9orf142* | Uncharacterized protein C9orf142 OS=Homo sapiens GN=C9orf142 PE=1 SV=2 - [CI142_HUMAN] | 0.945 |
| P07197 | *NEFM* | Neurofilament medium polypeptide OS=Homo sapiens GN=NEFM PE=1 SV=3 - [NFM_HUMAN] | 0.792 |
| O95139 | *NDUFB6* | NADH dehydrogenase [ubiquinone] 1 beta subcomplex subunit 6 OS=Homo sapiens GN=NDUFB6 PE=1 SV=3 - [NDUB6_HUMAN] | 0.949 |
| Q14914 | *PTGR1* | Prostaglandin reductase 1 OS=Homo sapiens GN=PTGR1 PE=1 SV=2 - [PTGR1_HUMAN] | 0.826 |
| P08236 | *GUSB* | Isoform Short of Beta-glucuronidase OS=Homo sapiens GN=GUSB - [BGLR_HUMAN] | 1.134 |
| Q9HA77 | *CARS2* | Probable cysteine--tRNA ligase, mitochondrial OS=Homo sapiens GN=CARS2 PE=1 SV=1 - [SYCM_HUMAN] | 0.910 |
| P36873 | *PPP1CC* | Serine/threonine-protein phosphatase PP1-gamma catalytic subunit OS=Homo sapiens GN=PPP1CC PE=1 SV=1 - [PP1G_HUMAN] | 0.968 |
| P09874 | *PARP1* | Poly [ADP-ribose] polymerase 1 OS=Homo sapiens GN=PARP1 PE=1 SV=4 - [PARP1_HUMAN] | 1.100 |
| P07910 | *HNRNPC* | Isoform C1 of Heterogeneous nuclear ribonucleoproteins C1/C2 OS=Homo sapiens GN=HNRNPC - [HNRPC_HUMAN] | 0.954 |
| Q9Y2S0 | *POLR1D* | DNA-directed RNA polymerases I and III subunit RPAC2 OS=Homo sapiens GN=POLR1D PE=1 SV=1 - [RPAC2_HUMAN] | 1.060 |
| O43852 | *CALU* | Calumenin OS=Homo sapiens GN=CALU PE=1 SV=2 - [CALU_HUMAN] | 1.120 |
| Q93100 | *PHKB* | Isoform 2 of Phosphorylase b kinase regulatory subunit beta OS=Homo sapiens GN=PHKB - [KPBB_HUMAN] | 1.087 |
| P01857 | *IGHG1* | Ig gamma-1 chain C region OS=Homo sapiens GN=IGHG1 PE=1 SV=1 - [IGHG1_HUMAN] | 0.809 |
| P78560 | *CRADD* | Death domain-containing protein CRADD OS=Homo sapiens GN=CRADD PE=1 SV=1 - [CRADD_HUMAN] | 1.056 |
| Q9UQ90 | *SPG7* | Isoform 2 of Paraplegin OS=Homo sapiens GN=SPG7 - [SPG7_HUMAN] | 1.115 |
| O75367 | *H2AFY* | Isoform 1 of Core histone macro-H2A.1 OS=Homo sapiens GN=H2AFY - [H2AY_HUMAN] | 0.892 |
| P54578 | *USP14* | Ubiquitin carboxyl-terminal hydrolase 14 OS=Homo sapiens GN=USP14 PE=1 SV=3 - [UBP14_HUMAN] | 0.945 |
| Q5SRH9 | *TTC39A* | Isoform 2 of Tetratricopeptide repeat protein 39A OS=Homo sapiens GN=TTC39A - [TT39A_HUMAN] | 1.273 |
| P12111 | *COL6A3* | Collagen alpha-3(VI) chain OS=Homo sapiens GN=COL6A3 PE=1 SV=5 - [CO6A3_HUMAN] | 0.787 |
| O75643 | *SNRNP200* | U5 small nuclear ribonucleoprotein 200 kDa helicase OS=Homo sapiens GN=SNRNP200 PE=1 SV=2 - [U520_HUMAN] | 1.046 |
| Q9NS86 | *LANCL2* | LanC-like protein 2 OS=Homo sapiens GN=LANCL2 PE=1 SV=1 - [LANC2_HUMAN] | 0.941 |
| Q9UPP1 | *PHF8* | Isoform 3 of Histone lysine demethylase PHF8 OS=Homo sapiens GN=PHF8 - [PHF8_HUMAN] | 1.084 |
| P09493 | *TPM1* | Isoform 5 of Tropomyosin alpha-1 chain OS=Homo sapiens GN=TPM1 - [TPM1_HUMAN] | 0.908 |
| P13674 | *P4HA1* | Prolyl 4-hydroxylase subunit alpha-1 OS=Homo sapiens GN=P4HA1 PE=1 SV=2 - [P4HA1_HUMAN] | 1.244 |
| O00442 | *RTCA* | RNA 3'-terminal phosphate cyclase OS=Homo sapiens GN=RTCD1 PE=1 SV=1 - [RTC1_HUMAN] | 0.934 |
| O75306 | *NDUFS2* | NADH dehydrogenase [ubiquinone] iron-sulfur protein 2, mitochondrial OS=Homo sapiens GN=NDUFS2 PE=1 SV=2 - [NDUS2_HUMAN] | 0.954 |
| Q8IW45 | *CARKD* | Isoform 4 of Carbohydrate kinase domain-containing protein OS=Homo sapiens GN=CARKD - [CARKD_HUMAN] | 0.940 |
| O95218 | *ZRANB2* | Isoform 2 of Zinc finger Ran-binding domain-containing protein 2 OS=Homo sapiens GN=ZRANB2 - [ZRAB2_HUMAN] | 1.230 |
| Q9P2D0 | *IBTK* | Isoform 2 of Inhibitor of Bruton tyrosine kinase OS=Homo sapiens GN=IBTK - [IBTK_HUMAN] | 0.942 |
| Q13685 | *AAMP* | Angio-associated migratory cell protein OS=Homo sapiens GN=AAMP PE=1 SV=2 - [AAMP_HUMAN] | 0.911 |
| O15085 | *ARHGEF11* | Rho guanine nucleotide exchange factor 11 OS=Homo sapiens GN=ARHGEF11 PE=1 SV=1 - [ARHGB_HUMAN] | 1.155 |
| Q9NV96 | *TMEM30A* | Isoform 2 of Cell cycle control protein 50A OS=Homo sapiens GN=TMEM30A - [CC50A_HUMAN] | 0.915 |
| Q16891 | *IMMT* | Isoform 3 of Mitochondrial inner membrane protein OS=Homo sapiens GN=IMMT - [IMMT_HUMAN] | 0.944 |
| P13010 | *XRCC5* | X-ray repair cross-complementing protein 5 OS=Homo sapiens GN=XRCC5 PE=1 SV=3 - [XRCC5_HUMAN] | 1.073 |
| Q9NWB6 | *ARGLU1* | Arginine and glutamate-rich protein 1 OS=Homo sapiens GN=ARGLU1 PE=1 SV=1 - [ARGL1_HUMAN] | 1.085 |
| Q16204 | *CCDC6* | Coiled-coil domain-containing protein 6 OS=Homo sapiens GN=CCDC6 PE=1 SV=2 - [CCDC6_HUMAN] | 1.123 |
| Q16595 | *FXN* | Isoform 2 of Frataxin, mitochondrial OS=Homo sapiens GN=FXN - [FRDA_HUMAN] | 0.943 |
| P49755 | *TMED10* | Transmembrane emp24 domain-containing protein 10 OS=Homo sapiens GN=TMED10 PE=1 SV=2 - [TMEDA_HUMAN] | 1.088 |
| P02730 | *SLC4A1* | Band 3 anion transport protein OS=Homo sapiens GN=SLC4A1 PE=1 SV=3 - [B3AT_HUMAN] | 0.628 |
| Q9H7B4 | *SMYD3* | Isoform 2 of SET and MYND domain-containing protein 3 OS=Homo sapiens GN=SMYD3 - [SMYD3_HUMAN] | 0.864 |
| Q4G0N4 | *NADK2* | Isoform 2 of NAD kinase domain-containing protein 1 OS=Homo sapiens GN=NADKD1 - [NAKD1_HUMAN] | 0.846 |
| Q9UJ70 | *NAGK* | N-acetyl-D-glucosamine kinase OS=Homo sapiens GN=NAGK PE=1 SV=4 - [NAGK_HUMAN] | 0.918 |
| Q9Y3Y2 | *CHTOP* | Isoform 3 of Chromatin target of PRMT1 protein OS=Homo sapiens GN=CHTOP - [CHTOP_HUMAN] | 1.061 |
| O95232 | *LUC7L3* | Luc7-like protein 3 OS=Homo sapiens GN=LUC7L3 PE=1 SV=2 - [LC7L3_HUMAN] | 1.082 |
| Q6P1N9 | *TATDN1* | Putative deoxyribonuclease TATDN1 OS=Homo sapiens GN=TATDN1 PE=1 SV=2 - [TATD1_HUMAN] | 1.108 |
| Q96CW5 | *TUBGCP3* | Isoform 3 of Gamma-tubulin complex component 3 OS=Homo sapiens GN=TUBGCP3 - [GCP3_HUMAN] | 0.933 |
| P18077 | *RPL35A* | 60S ribosomal protein L35a OS=Homo sapiens GN=RPL35A PE=1 SV=2 - [RL35A_HUMAN] | 1.080 |
| Q92797 | *SYMPK* | Symplekin OS=Homo sapiens GN=SYMPK PE=1 SV=2 - [SYMPK_HUMAN] | 1.090 |
| Q5VZK9 | *LRRC16A* | Isoform 2 of Leucine-rich repeat-containing protein 16A OS=Homo sapiens GN=LRRC16A - [LR16A_HUMAN] | 1.121 |
| Q8WVC0 | *LEO1* | RNA polymerase-associated protein LEO1 OS=Homo sapiens GN=LEO1 PE=1 SV=1 - [LEO1_HUMAN] | 0.939 |
| P51531 | *SMARCA2* | Isoform Short of Probable global transcription activator SNF2L2 OS=Homo sapiens GN=SMARCA2 - [SMCA2_HUMAN] | 1.041 |
| Q9Y490 | *TLN1* | Talin-1 OS=Homo sapiens GN=TLN1 PE=1 SV=3 - [TLN1_HUMAN] | 0.926 |
| P98164 | *LRP2* | Low-density lipoprotein receptor-related protein 2 OS=Homo sapiens GN=LRP2 PE=1 SV=3 - [LRP2_HUMAN] | 0.696 |
| Q86TX2 | *ACOT1* | Acyl-coenzyme A thioesterase 1 OS=Homo sapiens GN=ACOT1 PE=1 SV=1 - [ACOT1_HUMAN] | 0.909 |
| Q92945 | *KHSRP* | Isoform 2 of Far upstream element-binding protein 2 OS=Homo sapiens GN=KHSRP - [FUBP2_HUMAN] | 1.067 |
| O60828 | *PQBP1* | Isoform 6 of Polyglutamine-binding protein 1 OS=Homo sapiens GN=PQBP1 - [PQBP1_HUMAN] | 1.111 |
| P50452 | *SERPINB8* | Serpin B8 OS=Homo sapiens GN=SERPINB8 PE=1 SV=2 - [SPB8_HUMAN] | 0.924 |
| O14975 | *SLC27A2* | Very long-chain acyl-CoA synthetase OS=Homo sapiens GN=SLC27A2 PE=1 SV=2 - [S27A2_HUMAN] | 1.194 |
| Q6ZVF9 | *GPRIN3* | G protein-regulated inducer of neurite outgrowth 3 OS=Homo sapiens GN=GPRIN3 PE=2 SV=2 - [GRIN3_HUMAN] | 0.832 |
| Q9H3U7 | *SMOC2* | SPARC-related modular calcium-binding protein 2 OS=Homo sapiens GN=SMOC2 PE=2 SV=2 - [SMOC2_HUMAN] | 0.779 |
| Q05707 | *COL14A1* | Isoform 3 of Collagen alpha-1(XIV) chain OS=Homo sapiens GN=COL14A1 - [COEA1_HUMAN] | 0.728 |
| O95858 | *TSPAN15* | Tetraspanin-15 OS=Homo sapiens GN=TSPAN15 PE=2 SV=1 - [TSN15_HUMAN] | 0.803 |
| Q9NR30 | *DDX21* | Nucleolar RNA helicase 2 OS=Homo sapiens GN=DDX21 PE=1 SV=5 - [DDX21_HUMAN] | 1.097 |
| O95619 | *YEATS4* | YEATS domain-containing protein 4 OS=Homo sapiens GN=YEATS4 PE=1 SV=1 - [YETS4_HUMAN] | 1.062 |
| P04264 | *KRT1* | Keratin, type II cytoskeletal 1 OS=Homo sapiens GN=KRT1 PE=1 SV=6 - [K2C1_HUMAN] | 0.728 |
| Q9BUJ2 | *HNRNPUL1* | Isoform 4 of Heterogeneous nuclear ribonucleoprotein U-like protein 1 OS=Homo sapiens GN=HNRNPUL1 - [HNRL1_HUMAN] | 0.930 |
| Q8TBR7 | *FAM57A* | Isoform 1 of Protein FAM57A OS=Homo sapiens GN=FAM57A - [FA57A_HUMAN] | 1.441 |
| Q6PJT7 | *ZC3H14* | Isoform 4 of Zinc finger CCCH domain-containing protein 14 OS=Homo sapiens GN=ZC3H14 - [ZC3HE_HUMAN] | 1.057 |
| P54252 | *ATXN3* | Isoform 2 of Ataxin-3 OS=Homo sapiens GN=ATXN3 - [ATX3_HUMAN] | 1.086 |
| Q15181 | *PPA1* | Inorganic pyrophosphatase OS=Homo sapiens GN=PPA1 PE=1 SV=2 - [IPYR_HUMAN] | 1.115 |
| Q6UWY5 | *OLFML1* | Olfactomedin-like protein 1 OS=Homo sapiens GN=OLFML1 PE=1 SV=2 - [OLFL1_HUMAN] | 0.697 |
| Q68D10 | *SPTY2D1* | Isoform 3 of Protein SPT2 homolog OS=Homo sapiens GN=SPTY2D1 - [SPT2_HUMAN] | 1.068 |
| Q53GS9 | *USP39* | U4/U6.U5 tri-snRNP-associated protein 2 OS=Homo sapiens GN=USP39 PE=1 SV=2 - [SNUT2_HUMAN] | 1.070 |
| P14373 | *TRIM27* | Isoform Beta of Zinc finger protein RFP OS=Homo sapiens GN=TRIM27 - [TRI27_HUMAN] | 1.190 |
| Q96CP2 | *FLYWCH2* | FLYWCH family member 2 OS=Homo sapiens GN=FLYWCH2 PE=1 SV=1 - [FWCH2_HUMAN] | 1.135 |
| O60268 | *KIAA0513* | Isoform 2 of Uncharacterized protein KIAA0513 OS=Homo sapiens GN=KIAA0513 - [K0513_HUMAN] | 0.849 |
| Q6NXT6 | *TAPT1* | Transmembrane anterior posterior transformation protein 1 homolog OS=Homo sapiens GN=TAPT1 PE=1 SV=1 - [TAPT1_HUMAN] | 1.113 |
| P49756 | *RBM25* | RNA-binding protein 25 OS=Homo sapiens GN=RBM25 PE=1 SV=3 - [RBM25_HUMAN] | 1.036 |
| O75391 | *SPAG7* | Sperm-associated antigen 7 OS=Homo sapiens GN=SPAG7 PE=1 SV=2 - [SPAG7_HUMAN] | 1.080 |
| Q13049 | *TRIM32* | E3 ubiquitin-protein ligase TRIM32 OS=Homo sapiens GN=TRIM32 PE=1 SV=2 - [TRI32_HUMAN] | 0.945 |
| P21399 | *ACO1* | Cytoplasmic aconitate hydratase OS=Homo sapiens GN=ACO1 PE=1 SV=3 - [ACOC_HUMAN] | 1.083 |
| Q92608 | *DOCK2* | Dedicator of cytokinesis protein 2 OS=Homo sapiens GN=DOCK2 PE=1 SV=2 - [DOCK2_HUMAN] | 1.205 |
| Q4J6C6 | *PREPL* | Isoform 4 of Prolyl endopeptidase-like OS=Homo sapiens GN=PREPL - [PPCEL_HUMAN] | 0.873 |
| Q0VD83 | *APOBR* | Isoform 2 of Apolipoprotein B receptor OS=Homo sapiens GN=APOBR - [APOBR_HUMAN] | 0.913 |
| O60934 | *NBN* | Nibrin OS=Homo sapiens GN=NBN PE=1 SV=1 - [NBN_HUMAN] | 1.050 |
| Q8TCJ2 | *STT3B* | Dolichyl-diphosphooligosaccharide--protein glycosyltransferase subunit STT3B OS=Homo sapiens GN=STT3B PE=1 SV=1 - [STT3B_HUMAN] | 0.921 |
| Q8N9I0 | *SYT2* | Synaptotagmin-2 OS=Homo sapiens GN=SYT2 PE=1 SV=2 - [SYT2_HUMAN] | 0.747 |
| Q16891 | *IMMT* | Isoform 2 of Mitochondrial inner membrane protein OS=Homo sapiens GN=IMMT - [IMMT_HUMAN] | 0.931 |
| P46782 | *RPS5* | 40S ribosomal protein S5 OS=Homo sapiens GN=RPS5 PE=1 SV=4 - [RS5_HUMAN] | 1.062 |
| Q9UNE2 | *RPH3AL* | Isoform 2 of Rab effector Noc2 OS=Homo sapiens GN=RPH3AL - [RPH3L_HUMAN] | 0.771 |
| Q9H0H0 | *INTS2* | Integrator complex subunit 2 OS=Homo sapiens GN=INTS2 PE=1 SV=2 - [INT2_HUMAN] | 1.033 |
| O95777 | *LSM8* | N-alpha-acetyltransferase 38, NatC auxiliary subunit OS=Homo sapiens GN=NAA38 PE=1 SV=3 - [NAA38_HUMAN] | 1.092 |
| P53621 | *COPA* | Coatomer subunit alpha OS=Homo sapiens GN=COPA PE=1 SV=2 - [COPA_HUMAN] | 1.074 |
| Q9HBL8 | *NMRAL1* | NmrA-like family domain-containing protein 1 OS=Homo sapiens GN=NMRAL1 PE=1 SV=1 - [NMRL1_HUMAN] | 1.117 |
| P49915 | *GMPS* | GMP synthase [glutamine-hydrolyzing] OS=Homo sapiens GN=GMPS PE=1 SV=1 - [GUAA_HUMAN] | 0.935 |
| Q9Y223 | *GNE* | Isoform 3 of Bifunctional UDP-N-acetylglucosamine 2-epimerase/N-acetylmannosamine kinase OS=Homo sapiens GN=GNE - [GLCNE_HUMAN] | 1.077 |
| Q14527 | *HLTF* | Isoform 2 of Helicase-like transcription factor OS=Homo sapiens GN=HLTF - [HLTF_HUMAN] | 1.134 |
| P49789 | *FHIT* | Bis(5'-adenosyl)-triphosphatase OS=Homo sapiens GN=FHIT PE=1 SV=3 - [FHIT_HUMAN] | 0.875 |
| Q6NV74 | *KIAA1211L* | Uncharacterized protein C2orf55 OS=Homo sapiens GN=C2orf55 PE=1 SV=3 - [CB055_HUMAN] | 0.899 |
| Q9NVT9 | *ARMC1* | Armadillo repeat-containing protein 1 OS=Homo sapiens GN=ARMC1 PE=1 SV=1 - [ARMC1_HUMAN] | 0.927 |
| Q9UBS4 | *DNAJB11* | DnaJ homolog subfamily B member 11 OS=Homo sapiens GN=DNAJB11 PE=1 SV=1 - [DJB11_HUMAN] | 1.104 |
| Q969X5 | *ERGIC1* | Isoform 2 of Endoplasmic reticulum-Golgi intermediate compartment protein 1 OS=Homo sapiens GN=ERGIC1 - [ERGI1_HUMAN] | 1.069 |
| Q13637 | *RAB32* | Ras-related protein Rab-32 OS=Homo sapiens GN=RAB32 PE=1 SV=3 - [RAB32_HUMAN] | 0.886 |
| Q8TB61 | *SLC35B2* | Isoform 3 of Adenosine 3'-phospho 5'-phosphosulfate transporter 1 OS=Homo sapiens GN=SLC35B2 - [S35B2_HUMAN] | 1.139 |
| Q9Y6L7 | *TLL2* | Tolloid-like protein 2 OS=Homo sapiens GN=TLL2 PE=1 SV=1 - [TLL2_HUMAN] | 0.720 |
| O15541 | *RNF113A* | RING finger protein 113A OS=Homo sapiens GN=RNF113A PE=1 SV=1 - [R113A_HUMAN] | 1.099 |
| Q8NEB9 | *PIK3C3* | Phosphatidylinositol 3-kinase catalytic subunit type 3 OS=Homo sapiens GN=PIK3C3 PE=1 SV=1 - [PK3C3_HUMAN] | 0.911 |
| O15305 | *PMM2* | Phosphomannomutase 2 OS=Homo sapiens GN=PMM2 PE=1 SV=1 - [PMM2_HUMAN] | 1.093 |
| O00410 | *IPO5* | Importin-5 OS=Homo sapiens GN=IPO5 PE=1 SV=4 - [IPO5_HUMAN] | 1.082 |
| P61088 | *UBE2N* | Ubiquitin-conjugating enzyme E2 N OS=Homo sapiens GN=UBE2N PE=1 SV=1 - [UBE2N_HUMAN] | 1.074 |
| Q13287 | *NMI* | N-myc-interactor OS=Homo sapiens GN=NMI PE=1 SV=2 - [NMI_HUMAN] | 1.129 |
| P27540 | *ARNT* | Isoform 2 of Aryl hydrocarbon receptor nuclear translocator OS=Homo sapiens GN=ARNT - [ARNT_HUMAN] | 1.100 |
| Q05084 | *ICA1* | Islet cell autoantigen 1 OS=Homo sapiens GN=ICA1 PE=1 SV=2 - [ICA69_HUMAN] | 0.877 |
| Q16531 | *DDB1* | DNA damage-binding protein 1 OS=Homo sapiens GN=DDB1 PE=1 SV=1 - [DDB1_HUMAN] | 0.944 |
| Q0ZGT2 | *NEXN* | Isoform 2 of Nexilin OS=Homo sapiens GN=NEXN - [NEXN_HUMAN] | 1.249 |
| O00154 | *ACOT7* | Isoform 2 of Cytosolic acyl coenzyme A thioester hydrolase OS=Homo sapiens GN=ACOT7 - [BACH_HUMAN] | 1.126 |
| O43805 | *SSNA1* | Sjoegren syndrome nuclear autoantigen 1 OS=Homo sapiens GN=SSNA1 PE=1 SV=2 - [SSNA1_HUMAN] | 1.088 |
| Q9Y6B6 | *SAR1B* | GTP-binding protein SAR1b OS=Homo sapiens GN=SAR1B PE=1 SV=1 - [SAR1B_HUMAN] | 0.930 |
| O14964 | *HGS* | Isoform 2 of Hepatocyte growth factor-regulated tyrosine kinase substrate OS=Homo sapiens GN=HGS - [HGS_HUMAN] | 1.081 |
| P55081 | *MFAP1* | Microfibrillar-associated protein 1 OS=Homo sapiens GN=MFAP1 PE=1 SV=2 - [MFAP1_HUMAN] | 1.043 |
| O14579 | *COPE* | Coatomer subunit epsilon OS=Homo sapiens GN=COPE PE=1 SV=3 - [COPE_HUMAN] | 1.081 |
| P34059 | *GALNS* | N-acetylgalactosamine-6-sulfatase OS=Homo sapiens GN=GALNS PE=1 SV=1 - [GALNS_HUMAN] | 0.905 |
| Q04637 | *EIF4G1* | Isoform D of Eukaryotic translation initiation factor 4 gamma 1 OS=Homo sapiens GN=EIF4G1 - [IF4G1_HUMAN] | 1.051 |
| Q9BYJ9 | *YTHDF1* | YTH domain family protein 1 OS=Homo sapiens GN=YTHDF1 PE=1 SV=1 - [YTHD1_HUMAN] | 0.976 |
| Q9BRK4 | *LZTS2* | Leucine zipper putative tumor suppressor 2 OS=Homo sapiens GN=LZTS2 PE=1 SV=2 - [LZTS2_HUMAN] | 1.068 |
| P48059 | *LIMS1* | LIM and senescent cell antigen-like-containing domain protein 1 OS=Homo sapiens GN=LIMS1 PE=1 SV=4 - [LIMS1_HUMAN] | 0.910 |
| Q8IZ21 | *PHACTR4* | Isoform 3 of Phosphatase and actin regulator 4 OS=Homo sapiens GN=PHACTR4 - [PHAR4_HUMAN] | 1.077 |
| P34932 | *HSPA4* | Heat shock 70 kDa protein 4 OS=Homo sapiens GN=HSPA4 PE=1 SV=4 - [HSP74_HUMAN] | 1.069 |
| Q9Y3E1 | *0* | Hepatoma-derived growth factor-related protein 3 OS=Homo sapiens GN=HDGFRP3 PE=1 SV=1 - [HDGR3_HUMAN] | 0.911 |
| P43243 | *MATR3* | Matrin-3 OS=Homo sapiens GN=MATR3 PE=1 SV=2 - [MATR3_HUMAN] | 1.065 |
| Q5M9Q1 | *NKAPL* | NKAP-like protein OS=Homo sapiens GN=NKAPL PE=1 SV=3 - [NKAPL_HUMAN] | 1.054 |
| Q9Y5X1 | *SNX9* | Sorting nexin-9 OS=Homo sapiens GN=SNX9 PE=1 SV=1 - [SNX9_HUMAN] | 1.113 |
| P35613 | *BSG* | Isoform 2 of Basigin OS=Homo sapiens GN=BSG - [BASI_HUMAN] | 0.887 |
| Q04917 | *YWHAH* | 14-3-3 protein eta OS=Homo sapiens GN=YWHAH PE=1 SV=4 - [1433F_HUMAN] | 1.089 |
| P10398 | *ARAF* | Serine/threonine-protein kinase A-Raf OS=Homo sapiens GN=ARAF PE=1 SV=2 - [ARAF_HUMAN] | 0.945 |
| Q9Y547 | *HSPB11* | Heat shock protein beta-11 OS=Homo sapiens GN=HSPB11 PE=1 SV=1 - [HSB11_HUMAN] | 0.922 |
| Q9H0F7 | *ARL6* | ADP-ribosylation factor-like protein 6 OS=Homo sapiens GN=ARL6 PE=1 SV=1 - [ARL6_HUMAN] | 0.819 |
| P48382 | *RFX5* | DNA-binding protein RFX5 OS=Homo sapiens GN=RFX5 PE=1 SV=1 - [RFX5_HUMAN] | 0.890 |
| P20338 | *RAB4A* | Ras-related protein Rab-4A OS=Homo sapiens GN=RAB4A PE=1 SV=2 - [RAB4A_HUMAN] | 1.099 |
| Q93008 | *USP9X* | Isoform 2 of Probable ubiquitin carboxyl-terminal hydrolase FAF-X OS=Homo sapiens GN=USP9X - [USP9X_HUMAN] | 0.917 |
| Q12873 | *CHD3* | Chromodomain-helicase-DNA-binding protein 3 OS=Homo sapiens GN=CHD3 PE=1 SV=3 - [CHD3_HUMAN] | 1.127 |
| Q13162 | *PRDX4* | Peroxiredoxin-4 OS=Homo sapiens GN=PRDX4 PE=1 SV=1 - [PRDX4_HUMAN] | 1.186 |
| P15121 | *AKR1B1* | Aldose reductase OS=Homo sapiens GN=AKR1B1 PE=1 SV=3 - [ALDR_HUMAN] | 0.915 |
| O15372 | *EIF3H* | Eukaryotic translation initiation factor 3 subunit H OS=Homo sapiens GN=EIF3H PE=1 SV=1 - [EIF3H_HUMAN] | 0.952 |
| O94766 | *B3GAT3* | Galactosylgalactosylxylosylprotein 3-beta-glucuronosyltransferase 3 OS=Homo sapiens GN=B3GAT3 PE=1 SV=2 - [B3GA3_HUMAN] | 1.123 |
| Q9NQZ5 | *STARD7* | StAR-related lipid transfer protein 7, mitochondrial OS=Homo sapiens GN=STARD7 PE=1 SV=2 - [STAR7_HUMAN] | 0.933 |
| Q92615 | *LARP4B* | La-related protein 4B OS=Homo sapiens GN=LARP4B PE=1 SV=3 - [LAR4B_HUMAN] | 1.046 |
| P02452 | *COL1A1* | Collagen alpha-1(I) chain OS=Homo sapiens GN=COL1A1 PE=1 SV=5 - [CO1A1_HUMAN] | 1.367 |
| Q13948 | *CUX1* | Isoform 8 of Protein CASP OS=Homo sapiens GN=CUX1 - [CASP_HUMAN] | 1.071 |
| Q9UDY2 | *TJP2* | Isoform C1 of Tight junction protein ZO-2 OS=Homo sapiens GN=TJP2 - [ZO2_HUMAN] | 1.240 |
| P18031 | *PTPN1* | Tyrosine-protein phosphatase non-receptor type 1 OS=Homo sapiens GN=PTPN1 PE=1 SV=1 - [PTN1_HUMAN] | 1.063 |
| Q96IZ0 | *PAWR* | PRKC apoptosis WT1 regulator protein OS=Homo sapiens GN=PAWR PE=1 SV=1 - [PAWR_HUMAN] | 0.901 |
| Q9NQG5 | *RPRD1B* | Regulation of nuclear pre-mRNA domain-containing protein 1B OS=Homo sapiens GN=RPRD1B PE=1 SV=1 - [RPR1B_HUMAN] | 1.071 |
| Q9BW71 | *HIRIP3* | Isoform 2 of HIRA-interacting protein 3 OS=Homo sapiens GN=HIRIP3 - [HIRP3_HUMAN] | 1.165 |
| Q13275 | *SEMA3F* | Semaphorin-3F OS=Homo sapiens GN=SEMA3F PE=2 SV=2 - [SEM3F_HUMAN] | 1.698 |
| P11137 | *MAP2* | Isoform 3 of Microtubule-associated protein 2 OS=Homo sapiens GN=MAP2 - [MAP2_HUMAN] | 0.835 |
| Q9NR19 | *ACSS2* | Acetyl-coenzyme A synthetase, cytoplasmic OS=Homo sapiens GN=ACSS2 PE=1 SV=1 - [ACSA_HUMAN] | 1.113 |
| Q8TBF5 | *PIGX* | Phosphatidylinositol-glycan biosynthesis class X protein OS=Homo sapiens GN=PIGX PE=2 SV=3 - [PIGX_HUMAN] | 1.081 |
| P40616 | *ARL1* | ADP-ribosylation factor-like protein 1 OS=Homo sapiens GN=ARL1 PE=1 SV=1 - [ARL1_HUMAN] | 1.066 |
| Q9Y3A3 | *MOB4* | Isoform 2 of MOB-like protein phocein OS=Homo sapiens GN=MOB4 - [PHOCN_HUMAN] | 1.049 |
| P60604 | *UBE2G2* | Ubiquitin-conjugating enzyme E2 G2 OS=Homo sapiens GN=UBE2G2 PE=1 SV=1 - [UB2G2_HUMAN] | 1.105 |
| Q14BN4 | *SLMAP* | Isoform 2 of Sarcolemmal membrane-associated protein OS=Homo sapiens GN=SLMAP - [SLMAP_HUMAN] | 1.154 |
| P43897 | *TSFM* | Elongation factor Ts, mitochondrial OS=Homo sapiens GN=TSFM PE=1 SV=2 - [EFTS_HUMAN] | 0.958 |
| P62993 | *GRB2* | Isoform 2 of Growth factor receptor-bound protein 2 OS=Homo sapiens GN=GRB2 - [GRB2_HUMAN] | 1.075 |
| P23526 | *AHCY* | Adenosylhomocysteinase OS=Homo sapiens GN=AHCY PE=1 SV=4 - [SAHH_HUMAN] | 1.148 |
| Q9NUQ7 | *UFSP2* | Ufm1-specific protease 2 OS=Homo sapiens GN=UFSP2 PE=2 SV=3 - [UFSP2_HUMAN] | 0.932 |
| O94804 | *STK10* | Serine/threonine-protein kinase 10 OS=Homo sapiens GN=STK10 PE=1 SV=1 - [STK10_HUMAN] | 0.946 |
| Q9Y3Q3 | *TMED3* | Transmembrane emp24 domain-containing protein 3 OS=Homo sapiens GN=TMED3 PE=1 SV=1 - [TMED3_HUMAN] | 1.348 |
| P15927 | *RPA2* | Replication protein A 32 kDa subunit OS=Homo sapiens GN=RPA2 PE=1 SV=1 - [RFA2_HUMAN] | 0.938 |
| Q5TFE4 | *NT5DC1* | 5'-nucleotidase domain-containing protein 1 OS=Homo sapiens GN=NT5DC1 PE=1 SV=1 - [NT5D1_HUMAN] | 1.158 |
| Q07507 | *DPT* | Dermatopontin OS=Homo sapiens GN=DPT PE=2 SV=2 - [DERM_HUMAN] | 0.753 |
| Q6NXR4 | *TTI2* | TELO2-interacting protein 2 OS=Homo sapiens GN=TTI2 PE=1 SV=1 - [TTI2_HUMAN] | 1.082 |
| Q13510 | *ASAH1* | Acid ceramidase OS=Homo sapiens GN=ASAH1 PE=1 SV=5 - [ASAH1_HUMAN] | 0.870 |
| Q8WWM9 | *CYGB* | Cytoglobin OS=Homo sapiens GN=CYGB PE=1 SV=1 - [CYGB_HUMAN] | 1.163 |
| P04843 | *RPN1* | Dolichyl-diphosphooligosaccharide--protein glycosyltransferase subunit 1 OS=Homo sapiens GN=RPN1 PE=1 SV=1 - [RPN1_HUMAN] | 0.959 |
| Q9NX46 | *ADPRHL2* | Poly(ADP-ribose) glycohydrolase ARH3 OS=Homo sapiens GN=ADPRHL2 PE=1 SV=1 - [ARHL2_HUMAN] | 1.098 |
| Q9BZF9 | *UACA* | Uveal autoantigen with coiled-coil domains and ankyrin repeats OS=Homo sapiens GN=UACA PE=1 SV=2 - [UACA_HUMAN] | 1.119 |
| P21796 | *VDAC1* | Voltage-dependent anion-selective channel protein 1 OS=Homo sapiens GN=VDAC1 PE=1 SV=2 - [VDAC1_HUMAN] | 0.905 |
| Q5JSZ5 | *PRRC2B* | Protein PRRC2B OS=Homo sapiens GN=PRRC2B PE=1 SV=2 - [PRC2B_HUMAN] | 1.071 |
| P35914 | *HMGCL* | Hydroxymethylglutaryl-CoA lyase, mitochondrial OS=Homo sapiens GN=HMGCL PE=1 SV=2 - [HMGCL_HUMAN] | 0.892 |
| Q9H8Y8 | *GORASP2* | Golgi reassembly-stacking protein 2 OS=Homo sapiens GN=GORASP2 PE=1 SV=3 - [GORS2_HUMAN] | 1.086 |
| P23588 | *EIF4B* | Eukaryotic translation initiation factor 4B OS=Homo sapiens GN=EIF4B PE=1 SV=2 - [IF4B_HUMAN] | 0.946 |
| P02549 | *SPTA1* | Isoform 2 of Spectrin alpha chain, erythrocyte OS=Homo sapiens GN=SPTA1 - [SPTA1_HUMAN] | 0.673 |
| O60716 | *CTNND1* | Isoform 1A of Catenin delta-1 OS=Homo sapiens GN=CTNND1 - [CTND1_HUMAN] | 0.911 |
| P00492 | *HPRT1* | Hypoxanthine-guanine phosphoribosyltransferase OS=Homo sapiens GN=HPRT1 PE=1 SV=2 - [HPRT_HUMAN] | 0.920 |
| Q15369 | *TCEB1* | Transcription elongation factor B polypeptide 1 OS=Homo sapiens GN=TCEB1 PE=1 SV=1 - [ELOC_HUMAN] | 1.082 |
| Q9NUL3 | *STAU2* | Isoform 4 of Double-stranded RNA-binding protein Staufen homolog 2 OS=Homo sapiens GN=STAU2 - [STAU2_HUMAN] | 1.115 |
| P35221 | *CTNNA1* | Catenin alpha-1 OS=Homo sapiens GN=CTNNA1 PE=1 SV=1 - [CTNA1_HUMAN] | 0.903 |
| Q9P2E9 | *RRBP1* | Ribosome-binding protein 1 OS=Homo sapiens GN=RRBP1 PE=1 SV=4 - [RRBP1_HUMAN] | 0.935 |
| Q0JRZ9 | *FCHO2* | Isoform 2 of FCH domain only protein 2 OS=Homo sapiens GN=FCHO2 - [FCHO2_HUMAN] | 0.900 |
| P05452 | *CLEC3B* | Tetranectin OS=Homo sapiens GN=CLEC3B PE=1 SV=3 - [TETN_HUMAN] | 1.225 |
| Q96JH7 | *VCPIP1* | Deubiquitinating protein VCIP135 OS=Homo sapiens GN=VCPIP1 PE=1 SV=2 - [VCIP1_HUMAN] | 0.921 |
| P02751 | *FN1* | Isoform 12 of Fibronectin OS=Homo sapiens GN=FN1 - [FINC_HUMAN] | 0.775 |
| Q13813 | *SPTAN1* | Isoform 3 of Spectrin alpha chain, brain OS=Homo sapiens GN=SPTAN1 - [SPTA2_HUMAN] | 0.782 |
| Q14244 | *MAP7* | Isoform 2 of Ensconsin OS=Homo sapiens GN=MAP7 - [MAP7_HUMAN] | 0.883 |
| Q9BVC6 | *TMEM109* | Transmembrane protein 109 OS=Homo sapiens GN=TMEM109 PE=1 SV=1 - [TM109_HUMAN] | 0.931 |
| P50851 | *LRBA* | Isoform 2 of Lipopolysaccharide-responsive and beige-like anchor protein OS=Homo sapiens GN=LRBA - [LRBA_HUMAN] | 1.105 |
| Q8N1B4 | *VPS52* | Vacuolar protein sorting-associated protein 52 homolog OS=Homo sapiens GN=VPS52 PE=1 SV=1 - [VPS52_HUMAN] | 1.083 |
| Q9UL46 | *PSME2* | Proteasome activator complex subunit 2 OS=Homo sapiens GN=PSME2 PE=1 SV=4 - [PSME2_HUMAN] | 1.140 |
| P07686 | *HEXB* | Beta-hexosaminidase subunit beta OS=Homo sapiens GN=HEXB PE=1 SV=3 - [HEXB_HUMAN] | 0.905 |
| Q8IZR5 | *CMTM4* | Isoform 3 of CKLF-like MARVEL transmembrane domain-containing protein 4 OS=Homo sapiens GN=CMTM4 - [CKLF4_HUMAN] | 1.176 |
| Q8IYS1 | *PM20D2* | Peptidase M20 domain-containing protein 2 OS=Homo sapiens GN=PM20D2 PE=1 SV=2 - [P20D2_HUMAN] | 1.167 |
| Q9UI30 | *TRMT112* | tRNA methyltransferase 112 homolog OS=Homo sapiens GN=TRMT112 PE=1 SV=1 - [TR112_HUMAN] | 0.925 |
| Q9UQN3 | *CHMP2B* | Charged multivesicular body protein 2b OS=Homo sapiens GN=CHMP2B PE=1 SV=1 - [CHM2B_HUMAN] | 0.921 |
| Q9NZC3 | *GDE1* | Glycerophosphodiester phosphodiesterase 1 OS=Homo sapiens GN=GDE1 PE=1 SV=1 - [GDE1_HUMAN] | 1.190 |
| Q9Y6X4 | *FAM169A* | Protein FAM169A OS=Homo sapiens GN=FAM169A PE=1 SV=2 - [F169A_HUMAN] | 0.903 |
| Q15363 | *TMED2* | Transmembrane emp24 domain-containing protein 2 OS=Homo sapiens GN=TMED2 PE=1 SV=1 - [TMED2_HUMAN] | 1.098 |
| P13929 | *ENO3* | Isoform 3 of Beta-enolase OS=Homo sapiens GN=ENO3 - [ENOB_HUMAN] | 0.609 |
| P53701 | *HCCS* | Cytochrome c-type heme lyase OS=Homo sapiens GN=HCCS PE=1 SV=1 - [CCHL_HUMAN] | 1.090 |
| Q14315 | *FLNC* | Isoform 2 of Filamin-C OS=Homo sapiens GN=FLNC - [FLNC_HUMAN] | 0.566 |
| Q96FW1 | *OTUB1* | Ubiquitin thioesterase OTUB1 OS=Homo sapiens GN=OTUB1 PE=1 SV=2 - [OTUB1_HUMAN] | 1.101 |
| Q9NUU6 | *FAM105A* | Protein FAM105A OS=Homo sapiens GN=FAM105A PE=2 SV=1 - [F105A_HUMAN] | 1.182 |
| Q92616 | *GCN1L1* | Translational activator GCN1 OS=Homo sapiens GN=GCN1L1 PE=1 SV=6 - [GCN1L_HUMAN] | 1.061 |
| P48444 | *ARCN1* | Coatomer subunit delta OS=Homo sapiens GN=ARCN1 PE=1 SV=1 - [COPD_HUMAN] | 1.085 |
| Q96HQ2 | *CDKN2AIPNL* | Isoform 2 of CDKN2AIP N-terminal-like protein OS=Homo sapiens GN=CDKN2AIPNL - [C2AIL_HUMAN] | 1.082 |
| P25445 | *FAS* | Isoform 6 of Tumor necrosis factor receptor superfamily member 6 OS=Homo sapiens GN=FAS - [TNR6_HUMAN] | 0.878 |
| Q8IWF6 | *DENND6A* | Protein FAM116A OS=Homo sapiens GN=FAM116A PE=2 SV=1 - [F116A_HUMAN] | 1.034 |
| Q8N2K0 | *ABHD12* | Monoacylglycerol lipase ABHD12 OS=Homo sapiens GN=ABHD12 PE=2 SV=2 - [ABD12_HUMAN] | 1.076 |
| Q12913 | *PTPRJ* | Receptor-type tyrosine-protein phosphatase eta OS=Homo sapiens GN=PTPRJ PE=1 SV=3 - [PTPRJ_HUMAN] | 1.150 |
| O43865 | *AHCYL1* | Putative adenosylhomocysteinase 2 OS=Homo sapiens GN=AHCYL1 PE=1 SV=2 - [SAHH2_HUMAN] | 1.178 |
| P50416 | *CPT1A* | Isoform 2 of Carnitine O-palmitoyltransferase 1, liver isoform OS=Homo sapiens GN=CPT1A - [CPT1A_HUMAN] | 1.178 |
| O15068 | *MCF2L* | Guanine nucleotide exchange factor DBS OS=Homo sapiens GN=MCF2L PE=1 SV=2 - [MCF2L_HUMAN] | 0.880 |
| Q6UX07 | *DHRS13* | Isoform 2 of Dehydrogenase/reductase SDR family member 13 OS=Homo sapiens GN=DHRS13 - [DHR13_HUMAN] | 1.097 |
| Q08170 | *SRSF4* | Serine/arginine-rich splicing factor 4 OS=Homo sapiens GN=SRSF4 PE=1 SV=2 - [SRSF4_HUMAN] | 1.115 |
| P52429 | *DGKE* | Diacylglycerol kinase epsilon OS=Homo sapiens GN=DGKE PE=2 SV=1 - [DGKE_HUMAN] | 1.124 |
| O94827 | *PLEKHG5* | Isoform 4 of Pleckstrin homology domain-containing family G member 5 OS=Homo sapiens GN=PLEKHG5 - [PKHG5_HUMAN] | 1.068 |
| P10645 | *CHGA* | Chromogranin-A OS=Homo sapiens GN=CHGA PE=1 SV=7 - [CMGA_HUMAN] | 1.331 |
| Q92932 | *PTPRN2* | Receptor-type tyrosine-protein phosphatase N2 OS=Homo sapiens GN=PTPRN2 PE=1 SV=2 - [PTPR2_HUMAN] | 0.803 |
| Q96EY1 | *DNAJA3* | Isoform 2 of DnaJ homolog subfamily A member 3, mitochondrial OS=Homo sapiens GN=DNAJA3 - [DNJA3_HUMAN] | 1.100 |
| O94832 | *MYO1D* | Myosin-Id OS=Homo sapiens GN=MYO1D PE=1 SV=2 - [MYO1D_HUMAN] | 1.087 |
| O00139 | *KIF2A* | Isoform 2 of Kinesin-like protein KIF2A OS=Homo sapiens GN=KIF2A - [KIF2A_HUMAN] | 1.068 |
| Q8WTS6 | *SETD7* | Histone-lysine N-methyltransferase SETD7 OS=Homo sapiens GN=SETD7 PE=1 SV=1 - [SETD7_HUMAN] | 1.124 |
| Q8NC96 | *NECAP1* | Adaptin ear-binding coat-associated protein 1 OS=Homo sapiens GN=NECAP1 PE=1 SV=2 - [NECP1_HUMAN] | 0.934 |
| P12270 | *TPR* | Nucleoprotein TPR OS=Homo sapiens GN=TPR PE=1 SV=3 - [TPR_HUMAN] | 0.933 |
| P29590 | *PML* | Isoform PML-6 of Protein PML OS=Homo sapiens GN=PML - [PML_HUMAN] | 1.140 |
| O75874 | *IDH1* | Isocitrate dehydrogenase [NADP] cytoplasmic OS=Homo sapiens GN=IDH1 PE=1 SV=2 - [IDHC_HUMAN] | 1.089 |
| Q8N9N2 | *ASCC1* | Isoform 2 of Activating signal cointegrator 1 complex subunit 1 OS=Homo sapiens GN=ASCC1 - [ASCC1_HUMAN] | 1.050 |
| Q96BM9 | *ARL8A* | ADP-ribosylation factor-like protein 8A OS=Homo sapiens GN=ARL8A PE=1 SV=1 - [ARL8A_HUMAN] | 1.095 |
| O00233 | *PSMD9* | Isoform p27-S of 26S proteasome non-ATPase regulatory subunit 9 OS=Homo sapiens GN=PSMD9 - [PSMD9_HUMAN] | 1.039 |
| Q8TAF3 | *WDR48* | Isoform 4 of WD repeat-containing protein 48 OS=Homo sapiens GN=WDR48 - [WDR48_HUMAN] | 0.916 |
| O75683 | *SURF6* | Surfeit locus protein 6 OS=Homo sapiens GN=SURF6 PE=1 SV=3 - [SURF6_HUMAN] | 1.108 |
| Q14978 | *NOLC1* | Nucleolar and coiled-body phosphoprotein 1 OS=Homo sapiens GN=NOLC1 PE=1 SV=2 - [NOLC1_HUMAN] | 1.084 |
| Q9BT78 | *COPS4* | COP9 signalosome complex subunit 4 OS=Homo sapiens GN=COPS4 PE=1 SV=1 - [CSN4_HUMAN] | 0.948 |
| Q12756 | *KIF1A* | Kinesin-like protein KIF1A OS=Homo sapiens GN=KIF1A PE=1 SV=2 - [KIF1A_HUMAN] | 0.896 |
| Q13315 | *ATM* | Serine-protein kinase ATM OS=Homo sapiens GN=ATM PE=1 SV=3 - [ATM_HUMAN] | 0.930 |
| P61923 | *COPZ1* | Coatomer subunit zeta-1 OS=Homo sapiens GN=COPZ1 PE=1 SV=1 - [COPZ1_HUMAN] | 1.092 |
| Q14232 | *EIF2B1* | Translation initiation factor eIF-2B subunit alpha OS=Homo sapiens GN=EIF2B1 PE=1 SV=1 - [EI2BA_HUMAN] | 1.058 |
| Q96RT1 | *ERBB2IP* | Isoform 7 of Protein LAP2 OS=Homo sapiens GN=ERBB2IP - [LAP2_HUMAN] | 1.036 |
| Q9Y5P4 | *COL4A3BP* | Isoform 2 of Collagen type IV alpha-3-binding protein OS=Homo sapiens GN=COL4A3BP - [C43BP_HUMAN] | 1.087 |
| Q6ZRP7 | *QSOX2* | Sulfhydryl oxidase 2 OS=Homo sapiens GN=QSOX2 PE=1 SV=3 - [QSOX2_HUMAN] | 1.155 |
| O14936 | *CASK* | Isoform 3 of Peripheral plasma membrane protein CASK OS=Homo sapiens GN=CASK - [CSKP_HUMAN] | 0.882 |
| P14678 | *SNRPB* | Isoform SM-B of Small nuclear ribonucleoprotein-associated proteins B and B' OS=Homo sapiens GN=SNRPB - [RSMB_HUMAN] | 1.034 |
| P02751 | *FN1* | Isoform 6 of Fibronectin OS=Homo sapiens GN=FN1 - [FINC_HUMAN] | 0.761 |
| Q15056 | *EIF4H* | Isoform Short of Eukaryotic translation initiation factor 4H OS=Homo sapiens GN=EIF4H - [IF4H_HUMAN] | 1.031 |
| O95479 | *H6PD* | GDH/6PGL endoplasmic bifunctional protein OS=Homo sapiens GN=H6PD PE=1 SV=2 - [G6PE_HUMAN] | 0.842 |
| P09619 | *PDGFRB* | Platelet-derived growth factor receptor beta OS=Homo sapiens GN=PDGFRB PE=1 SV=1 - [PGFRB_HUMAN] | 1.185 |
| Q9UMS0 | *NFU1* | Isoform 2 of NFU1 iron-sulfur cluster scaffold homolog, mitochondrial OS=Homo sapiens GN=NFU1 - [NFU1_HUMAN] | 0.945 |
| O60841 | *EIF5B* | Eukaryotic translation initiation factor 5B OS=Homo sapiens GN=EIF5B PE=1 SV=4 - [IF2P_HUMAN] | 0.963 |
| Q5VU43 | *PDE4DIP* | Isoform 2 of Myomegalin OS=Homo sapiens GN=PDE4DIP - [MYOME_HUMAN] | 0.961 |
| P49914 | *MTHFS* | 5-formyltetrahydrofolate cyclo-ligase OS=Homo sapiens GN=MTHFS PE=1 SV=2 - [MTHFS_HUMAN] | 0.921 |
| Q9NY47 | *CACNA2D2* | Isoform 2 of Voltage-dependent calcium channel subunit alpha-2/delta-2 OS=Homo sapiens GN=CACNA2D2 - [CA2D2_HUMAN] | 0.836 |
| Q969M1 | *TOMM40L* | Mitochondrial import receptor subunit TOM40B OS=Homo sapiens GN=TOMM40L PE=2 SV=1 - [TM40L_HUMAN] | 0.927 |
| P49593 | *PPM1F* | Protein phosphatase 1F OS=Homo sapiens GN=PPM1F PE=1 SV=3 - [PPM1F_HUMAN] | 1.147 |
| Q9BYG3 | *NIFK* | MKI67 FHA domain-interacting nucleolar phosphoprotein OS=Homo sapiens GN=MKI67IP PE=1 SV=1 - [MK67I_HUMAN] | 1.115 |
| Q05707 | *COL14A1* | Isoform 2 of Collagen alpha-1(XIV) chain OS=Homo sapiens GN=COL14A1 - [COEA1_HUMAN] | 0.757 |
| Q9NWS0 | *PIH1D1* | PIH1 domain-containing protein 1 OS=Homo sapiens GN=PIH1D1 PE=1 SV=1 - [PIHD1_HUMAN] | 0.924 |
| P46778 | *RPL21* | 60S ribosomal protein L21 OS=Homo sapiens GN=RPL21 PE=1 SV=2 - [RL21_HUMAN] | 0.942 |
| Q9BPX5 | *ARPC5L* | Actin-related protein 2/3 complex subunit 5-like protein OS=Homo sapiens GN=ARPC5L PE=1 SV=1 - [ARP5L_HUMAN] | 1.103 |
| Q9H330 | *TMEM245* | Isoform 3 of Transmembrane protein C9orf5 OS=Homo sapiens GN=C9orf5 - [CI005_HUMAN] | 1.076 |
| O75027 | *ABCB7* | ATP-binding cassette sub-family B member 7, mitochondrial OS=Homo sapiens GN=ABCB7 PE=1 SV=2 - [ABCB7_HUMAN] | 1.078 |
| P51580 | *TPMT* | Thiopurine S-methyltransferase OS=Homo sapiens GN=TPMT PE=1 SV=1 - [TPMT_HUMAN] | 1.178 |
| Q13367 | *AP3B2* | AP-3 complex subunit beta-2 OS=Homo sapiens GN=AP3B2 PE=1 SV=2 - [AP3B2_HUMAN] | 1.046 |
| P46736 | *BRCC3* | Isoform 1 of Lys-63-specific deubiquitinase BRCC36 OS=Homo sapiens GN=BRCC3 - [BRCC3_HUMAN] | 0.961 |
| A1L0T0 | *ILVBL* | Acetolactate synthase-like protein OS=Homo sapiens GN=ILVBL PE=1 SV=2 - [ILVBL_HUMAN] | 1.071 |
| Q9NQ48 | *LZTFL1* | Leucine zipper transcription factor-like protein 1 OS=Homo sapiens GN=LZTFL1 PE=1 SV=1 - [LZTL1_HUMAN] | 0.899 |
| Q99436 | *PSMB7* | Proteasome subunit beta type-7 OS=Homo sapiens GN=PSMB7 PE=1 SV=1 - [PSB7_HUMAN] | 1.063 |
| Q53HC9 | *TSSC1* | Protein TSSC1 OS=Homo sapiens GN=TSSC1 PE=1 SV=2 - [TSSC1_HUMAN] | 1.061 |
| Q9NV96 | *TMEM30A* | Isoform 3 of Cell cycle control protein 50A OS=Homo sapiens GN=TMEM30A - [CC50A_HUMAN] | 0.931 |
| Q13200 | *PSMD2* | 26S proteasome non-ATPase regulatory subunit 2 OS=Homo sapiens GN=PSMD2 PE=1 SV=3 - [PSMD2_HUMAN] | 1.033 |
| P00167 | *CYB5A* | Isoform 2 of Cytochrome b5 OS=Homo sapiens GN=CYB5A - [CYB5_HUMAN] | 1.222 |
| P57740 | *NUP107* | Nuclear pore complex protein Nup107 OS=Homo sapiens GN=NUP107 PE=1 SV=1 - [NU107_HUMAN] | 1.088 |
| Q9H7D7 | *WDR26* | Isoform 2 of WD repeat-containing protein 26 OS=Homo sapiens GN=WDR26 - [WDR26_HUMAN] | 1.068 |
| Q2TAY7 | *SMU1* | WD40 repeat-containing protein SMU1 OS=Homo sapiens GN=SMU1 PE=1 SV=2 - [SMU1_HUMAN] | 0.961 |
| Q9NS15 | *LTBP3* | Isoform 2 of Latent-transforming growth factor beta-binding protein 3 OS=Homo sapiens GN=LTBP3 - [LTBP3_HUMAN] | 1.124 |
| Q9Y365 | *STARD10* | PCTP-like protein OS=Homo sapiens GN=STARD10 PE=2 SV=2 - [PCTL_HUMAN] | 1.111 |
| P05787 | *KRT8* | Keratin, type II cytoskeletal 8 OS=Homo sapiens GN=KRT8 PE=1 SV=7 - [K2C8_HUMAN] | 1.093 |
| O94811 | *TPPP* | Tubulin polymerization-promoting protein OS=Homo sapiens GN=TPPP PE=1 SV=1 - [TPPP_HUMAN] | 1.137 |
| Q9Y262 | *EIF3L* | Eukaryotic translation initiation factor 3 subunit L OS=Homo sapiens GN=EIF3L PE=1 SV=1 - [EIF3L_HUMAN] | 1.040 |
| Q9NRG9 | *AAAS* | Aladin OS=Homo sapiens GN=AAAS PE=1 SV=1 - [AAAS_HUMAN] | 1.114 |
| Q9UG63 | *ABCF2* | ATP-binding cassette sub-family F member 2 OS=Homo sapiens GN=ABCF2 PE=1 SV=2 - [ABCF2_HUMAN] | 1.042 |
| P35219 | *CA8* | Carbonic anhydrase-related protein OS=Homo sapiens GN=CA8 PE=1 SV=3 - [CAH8_HUMAN] | 1.167 |
| Q9ULJ8 | *PPP1R9A* | Neurabin-1 OS=Homo sapiens GN=PPP1R9A PE=1 SV=2 - [NEB1_HUMAN] | 0.753 |
| Q01085 | *TIAL1* | Nucleolysin TIAR OS=Homo sapiens GN=TIAL1 PE=1 SV=1 - [TIAR_HUMAN] | 1.037 |
| Q13405 | *MRPL49* | 39S ribosomal protein L49, mitochondrial OS=Homo sapiens GN=MRPL49 PE=1 SV=1 - [RM49_HUMAN] | 0.959 |
| Q13247 | *SRSF6* | Isoform SRP55-3 of Serine/arginine-rich splicing factor 6 OS=Homo sapiens GN=SRSF6 - [SRSF6_HUMAN] | 0.917 |
| Q13641 | *TPBG* | Trophoblast glycoprotein OS=Homo sapiens GN=TPBG PE=1 SV=1 - [TPBG_HUMAN] | 0.838 |
| O14787 | *TNPO2* | Isoform 2 of Transportin-2 OS=Homo sapiens GN=TNPO2 - [TNPO2_HUMAN] | 1.070 |
| Q01082 | *SPTBN1* | Spectrin beta chain, brain 1 OS=Homo sapiens GN=SPTBN1 PE=1 SV=2 - [SPTB2_HUMAN] | 0.903 |
| P16157 | *ANK1* | Isoform Er9 of Ankyrin-1 OS=Homo sapiens GN=ANK1 - [ANK1_HUMAN] | 0.813 |
| Q9NVV4 | *MTPAP* | Poly(A) RNA polymerase, mitochondrial OS=Homo sapiens GN=MTPAP PE=1 SV=1 - [PAPD1_HUMAN] | 0.932 |
| Q15154 | *PCM1* | Isoform 2 of Pericentriolar material 1 protein OS=Homo sapiens GN=PCM1 - [PCM1_HUMAN] | 1.073 |
| Q86YS7 | *C2CD5* | Uncharacterized protein KIAA0528 OS=Homo sapiens GN=KIAA0528 PE=1 SV=1 - [K0528_HUMAN] | 0.944 |
| Q9UNP9 | *PPIE* | Isoform B of Peptidyl-prolyl cis-trans isomerase E OS=Homo sapiens GN=PPIE - [PPIE_HUMAN] | 0.887 |
| P02042 | *HBD* | Hemoglobin subunit delta OS=Homo sapiens GN=HBD PE=1 SV=2 - [HBD_HUMAN] | 0.747 |
| Q96KG9 | *SCYL1* | Isoform 5 of N-terminal kinase-like protein OS=Homo sapiens GN=SCYL1 - [NTKL_HUMAN] | 1.050 |
| Q7Z406 | *MYH14* | Isoform 6 of Myosin-14 OS=Homo sapiens GN=MYH14 - [MYH14_HUMAN] | 0.876 |
| Q4G0F5 | *VPS26B* | Vacuolar protein sorting-associated protein 26B OS=Homo sapiens GN=VPS26B PE=1 SV=2 - [VP26B_HUMAN] | 1.079 |
| Q9BPY8 | *HOPX* | Homeodomain-only protein OS=Homo sapiens GN=HOPX PE=1 SV=1 - [HOP_HUMAN] | 0.811 |
| Q9GZZ1 | *NAA50* | N-alpha-acetyltransferase 50 OS=Homo sapiens GN=NAA50 PE=1 SV=1 - [NAA50_HUMAN] | 1.064 |
| P12259 | *F5* | Coagulation factor V OS=Homo sapiens GN=F5 PE=1 SV=4 - [FA5_HUMAN] | 0.752 |
| Q86Y56 | *DNAAF5* | Isoform 2 of HEAT repeat-containing protein 2 OS=Homo sapiens GN=HEATR2 - [HEAT2_HUMAN] | 1.097 |
| Q99541 | *PLIN2* | Perilipin-2 OS=Homo sapiens GN=PLIN2 PE=1 SV=2 - [PLIN2_HUMAN] | 1.202 |
| P50453 | *SERPINB9* | Serpin B9 OS=Homo sapiens GN=SERPINB9 PE=1 SV=1 - [SPB9_HUMAN] | 1.146 |
| Q8IXJ6 | *SIRT2* | Isoform 4 of NAD-dependent deacetylase sirtuin-2 OS=Homo sapiens GN=SIRT2 - [SIRT2_HUMAN] | 1.131 |
| Q9Y2W1 | *THRAP3* | Thyroid hormone receptor-associated protein 3 OS=Homo sapiens GN=THRAP3 PE=1 SV=2 - [TR150_HUMAN] | 1.063 |
| Q8WWQ0 | *PHIP* | PH-interacting protein OS=Homo sapiens GN=PHIP PE=1 SV=2 - [PHIP_HUMAN] | 0.919 |
| Q86VZ4 | *LRP11* | Isoform 2 of Low-density lipoprotein receptor-related protein 11 OS=Homo sapiens GN=LRP11 - [LRP11_HUMAN] | 1.073 |
| P40222 | *TXLNA* | Alpha-taxilin OS=Homo sapiens GN=TXLNA PE=1 SV=3 - [TXLNA_HUMAN] | 1.053 |
| O14976 | *GAK* | Cyclin-G-associated kinase OS=Homo sapiens GN=GAK PE=1 SV=2 - [GAK_HUMAN] | 1.053 |
| P62316 | *SNRPD2* | Small nuclear ribonucleoprotein Sm D2 OS=Homo sapiens GN=SNRPD2 PE=1 SV=1 - [SMD2_HUMAN] | 1.036 |
| Q9HCU5 | *PREB* | Prolactin regulatory element-binding protein OS=Homo sapiens GN=PREB PE=1 SV=2 - [PREB_HUMAN] | 1.107 |
| Q9UKA9 | *PTBP2* | Polypyrimidine tract-binding protein 2 OS=Homo sapiens GN=PTBP2 PE=1 SV=1 - [PTBP2_HUMAN] | 0.825 |
| Q5T1M5 | *FKBP15* | Isoform 2 of FK506-binding protein 15 OS=Homo sapiens GN=FKBP15 - [FKB15_HUMAN] | 0.959 |
| Q6ZRV2 | *FAM83H* | Protein FAM83H OS=Homo sapiens GN=FAM83H PE=1 SV=3 - [FA83H_HUMAN] | 1.052 |
| Q53GG5 | *PDLIM3* | Isoform 3 of PDZ and LIM domain protein 3 OS=Homo sapiens GN=PDLIM3 - [PDLI3_HUMAN] | 1.153 |
| Q8WWH5 | *TRUB1* | Probable tRNA pseudouridine synthase 1 OS=Homo sapiens GN=TRUB1 PE=1 SV=1 - [TRUB1_HUMAN] | 1.203 |
| O94776 | *MTA2* | Metastasis-associated protein MTA2 OS=Homo sapiens GN=MTA2 PE=1 SV=1 - [MTA2_HUMAN] | 1.043 |
| Q92930 | *RAB8B* | Ras-related protein Rab-8B OS=Homo sapiens GN=RAB8B PE=1 SV=2 - [RAB8B_HUMAN] | 0.918 |
| O43395 | *PRPF3* | U4/U6 small nuclear ribonucleoprotein Prp3 OS=Homo sapiens GN=PRPF3 PE=1 SV=2 - [PRPF3_HUMAN] | 1.052 |
| O43615 | *TIMM44* | Mitochondrial import inner membrane translocase subunit TIM44 OS=Homo sapiens GN=TIMM44 PE=1 SV=2 - [TIM44_HUMAN] | 0.911 |
| Q5SSJ5 | *HP1BP3* | Heterochromatin protein 1-binding protein 3 OS=Homo sapiens GN=HP1BP3 PE=1 SV=1 - [HP1B3_HUMAN] | 1.097 |
| P18887 | *XRCC1* | DNA repair protein XRCC1 OS=Homo sapiens GN=XRCC1 PE=1 SV=2 - [XRCC1_HUMAN] | 1.053 |
| P16452 | *EPB42* | Erythrocyte membrane protein band 4.2 OS=Homo sapiens GN=EPB42 PE=1 SV=3 - [EPB42_HUMAN] | 0.741 |
| P62699 | *YPEL5* | Protein yippee-like 5 OS=Homo sapiens GN=YPEL5 PE=2 SV=1 - [YPEL5_HUMAN] | 1.055 |
| Q6PIU2 | *NCEH1* | Isoform 3 of Neutral cholesterol ester hydrolase 1 OS=Homo sapiens GN=NCEH1 - [NCEH1_HUMAN] | 0.900 |
| O43681 | *ASNA1* | ATPase ASNA1 OS=Homo sapiens GN=ASNA1 PE=1 SV=2 - [ASNA_HUMAN] | 1.072 |
| P27695 | *APEX1* | DNA-(apurinic or apyrimidinic site) lyase OS=Homo sapiens GN=APEX1 PE=1 SV=2 - [APEX1_HUMAN] | 1.049 |
| Q9BVG9 | *PTDSS2* | Phosphatidylserine synthase 2 OS=Homo sapiens GN=PTDSS2 PE=1 SV=1 - [PTSS2_HUMAN] | 1.130 |
| Q9H553 | *ALG2* | Isoform 2 of Alpha-1,3/1,6-mannosyltransferase ALG2 OS=Homo sapiens GN=ALG2 - [ALG2_HUMAN] | 0.940 |
| Q9UJJ9 | *GNPTG* | N-acetylglucosamine-1-phosphotransferase subunit gamma OS=Homo sapiens GN=GNPTG PE=1 SV=1 - [GNPTG_HUMAN] | 0.876 |
| Q9NZN5 | *ARHGEF12* | Isoform 2 of Rho guanine nucleotide exchange factor 12 OS=Homo sapiens GN=ARHGEF12 - [ARHGC_HUMAN] | 1.050 |
| Q13277 | *STX3* | Isoform B of Syntaxin-3 OS=Homo sapiens GN=STX3 - [STX3_HUMAN] | 1.183 |
| O00519 | *FAAH* | Fatty-acid amide hydrolase 1 OS=Homo sapiens GN=FAAH PE=1 SV=2 - [FAAH1_HUMAN] | 1.124 |
| A1L390 | *PLEKHG3* | Isoform 2 of Pleckstrin homology domain-containing family G member 3 OS=Homo sapiens GN=PLEKHG3 - [PKHG3_HUMAN] | 1.100 |
| Q9UPU5 | *USP24* | Ubiquitin carboxyl-terminal hydrolase 24 OS=Homo sapiens GN=USP24 PE=1 SV=3 - [UBP24_HUMAN] | 1.051 |
| Q9UQ35 | *SRRM2* | Isoform 2 of Serine/arginine repetitive matrix protein 2 OS=Homo sapiens GN=SRRM2 - [SRRM2_HUMAN] | 1.078 |
| Q7Z3B1 | *NEGR1* | Neuronal growth regulator 1 OS=Homo sapiens GN=NEGR1 PE=1 SV=3 - [NEGR1_HUMAN] | 0.804 |
| Q8IZ81 | *ELMOD2* | ELMO domain-containing protein 2 OS=Homo sapiens GN=ELMOD2 PE=1 SV=1 - [ELMD2_HUMAN] | 1.103 |
| Q99547 | *MPHOSPH6* | M-phase phosphoprotein 6 OS=Homo sapiens GN=MPHOSPH6 PE=1 SV=2 - [MPH6_HUMAN] | 1.068 |
| Q15185 | *PTGES3* | Prostaglandin E synthase 3 OS=Homo sapiens GN=PTGES3 PE=1 SV=1 - [TEBP_HUMAN] | 0.938 |
| Q9Y3D9 | *MRPS23* | 28S ribosomal protein S23, mitochondrial OS=Homo sapiens GN=MRPS23 PE=1 SV=2 - [RT23_HUMAN] | 0.930 |
| O43399 | *TPD52L2* | Isoform 2 of Tumor protein D54 OS=Homo sapiens GN=TPD52L2 - [TPD54_HUMAN] | 0.938 |
| Q02543 | *RPL18A* | 60S ribosomal protein L18a OS=Homo sapiens GN=RPL18A PE=1 SV=2 - [RL18A_HUMAN] | 1.035 |
| P30043 | *BLVRB* | Flavin reductase (NADPH) OS=Homo sapiens GN=BLVRB PE=1 SV=3 - [BLVRB_HUMAN] | 0.831 |
| O00154 | *ACOT7* | Isoform 6 of Cytosolic acyl coenzyme A thioester hydrolase OS=Homo sapiens GN=ACOT7 - [BACH_HUMAN] | 1.095 |
| Q15773 | *MLF2* | Myeloid leukemia factor 2 OS=Homo sapiens GN=MLF2 PE=1 SV=1 - [MLF2_HUMAN] | 1.161 |
| Q9NWZ3 | *IRAK4* | Isoform 2 of Interleukin-1 receptor-associated kinase 4 OS=Homo sapiens GN=IRAK4 - [IRAK4_HUMAN] | 1.161 |
| P61009 | *SPCS3* | Signal peptidase complex subunit 3 OS=Homo sapiens GN=SPCS3 PE=1 SV=1 - [SPCS3_HUMAN] | 1.054 |
| O43815 | *STRN* | Isoform 2 of Striatin OS=Homo sapiens GN=STRN - [STRN_HUMAN] | 1.071 |
| P63241 | *EIF5A* | Eukaryotic translation initiation factor 5A-1 OS=Homo sapiens GN=EIF5A PE=1 SV=2 - [IF5A1_HUMAN] | 0.960 |
| P00915 | *CA1* | Carbonic anhydrase 1 OS=Homo sapiens GN=CA1 PE=1 SV=2 - [CAH1_HUMAN] | 0.757 |
| Q8N4C8 | *MINK1* | Isoform 5 of Misshapen-like kinase 1 OS=Homo sapiens GN=MINK1 - [MINK1_HUMAN] | 0.939 |
| Q8N335 | *GPD1L* | Glycerol-3-phosphate dehydrogenase 1-like protein OS=Homo sapiens GN=GPD1L PE=1 SV=1 - [GPD1L_HUMAN] | 0.893 |
| P49366 | *DHPS* | Isoform Short of Deoxyhypusine synthase OS=Homo sapiens GN=DHPS - [DHYS_HUMAN] | 0.893 |
| Q96T37 | *RBM15* | Isoform 2 of Putative RNA-binding protein 15 OS=Homo sapiens GN=RBM15 - [RBM15_HUMAN] | 0.951 |
| Q96N67 | *DOCK7* | Isoform 4 of Dedicator of cytokinesis protein 7 OS=Homo sapiens GN=DOCK7 - [DOCK7_HUMAN] | 1.063 |
| Q99996 | *AKAP9* | Isoform 3 of A-kinase anchor protein 9 OS=Homo sapiens GN=AKAP9 - [AKAP9_HUMAN] | 0.962 |
| O76024 | *WFS1* | Wolframin OS=Homo sapiens GN=WFS1 PE=1 SV=2 - [WFS1_HUMAN] | 1.144 |
| P61981 | *YWHAG* | 14-3-3 protein gamma OS=Homo sapiens GN=YWHAG PE=1 SV=2 - [1433G_HUMAN] | 0.954 |
| Q9Y5Z4 | *HEBP2* | Heme-binding protein 2 OS=Homo sapiens GN=HEBP2 PE=1 SV=1 - [HEBP2_HUMAN] | 0.926 |
| Q9UHQ4 | *BCAP29* | B-cell receptor-associated protein 29 OS=Homo sapiens GN=BCAP29 PE=1 SV=2 - [BAP29_HUMAN] | 0.925 |
| Q15746 | *MYLK* | Isoform 5 of Myosin light chain kinase, smooth muscle OS=Homo sapiens GN=MYLK - [MYLK_HUMAN] | 0.851 |
| Q9BSJ2 | *TUBGCP2* | Gamma-tubulin complex component 2 OS=Homo sapiens GN=TUBGCP2 PE=1 SV=2 - [GCP2_HUMAN] | 0.942 |
| Q13325 | *IFIT5* | Interferon-induced protein with tetratricopeptide repeats 5 OS=Homo sapiens GN=IFIT5 PE=1 SV=1 - [IFIT5_HUMAN] | 1.264 |
| Q14185 | *DOCK1* | Dedicator of cytokinesis protein 1 OS=Homo sapiens GN=DOCK1 PE=1 SV=2 - [DOCK1_HUMAN] | 1.103 |
| Q8WYA0 | *IFT81* | Intraflagellar transport protein 81 homolog OS=Homo sapiens GN=IFT81 PE=1 SV=1 - [IFT81_HUMAN] | 0.950 |
| Q96JE9 | *MAP6* | Microtubule-associated protein 6 OS=Homo sapiens GN=MAP6 PE=1 SV=2 - [MAP6_HUMAN] | 0.903 |
| P38606 | *ATP6V1A* | V-type proton ATPase catalytic subunit A OS=Homo sapiens GN=ATP6V1A PE=1 SV=2 - [VATA_HUMAN] | 0.958 |
| Q14767 | *LTBP2* | Latent-transforming growth factor beta-binding protein 2 OS=Homo sapiens GN=LTBP2 PE=1 SV=3 - [LTBP2_HUMAN] | 1.250 |
| Q14571 | *ITPR2* | Inositol 1,4,5-trisphosphate receptor type 2 OS=Homo sapiens GN=ITPR2 PE=1 SV=2 - [ITPR2_HUMAN] | 0.918 |
| Q9HCD5 | *NCOA5* | Nuclear receptor coactivator 5 OS=Homo sapiens GN=NCOA5 PE=1 SV=2 - [NCOA5_HUMAN] | 1.043 |
| Q9Y5L4 | *TIMM13* | Mitochondrial import inner membrane translocase subunit Tim13 OS=Homo sapiens GN=TIMM13 PE=1 SV=1 - [TIM13_HUMAN] | 0.922 |
| Q96ED9 | *HOOK2* | Isoform 2 of Protein Hook homolog 2 OS=Homo sapiens GN=HOOK2 - [HOOK2_HUMAN] | 0.940 |
| Q92900 | *UPF1* | Isoform 2 of Regulator of nonsense transcripts 1 OS=Homo sapiens GN=UPF1 - [RENT1_HUMAN] | 1.033 |
| Q9NP61 | *ARFGAP3* | ADP-ribosylation factor GTPase-activating protein 3 OS=Homo sapiens GN=ARFGAP3 PE=1 SV=1 - [ARFG3_HUMAN] | 1.124 |
| P41091 | *EIF2S3* | Eukaryotic translation initiation factor 2 subunit 3 OS=Homo sapiens GN=EIF2S3 PE=1 SV=3 - [IF2G_HUMAN] | 1.039 |
| Q8IY22 | *CMIP* | Isoform 3 of C-Maf-inducing protein OS=Homo sapiens GN=CMIP - [CMIP_HUMAN] | 0.908 |
| O43493 | *TGOLN2* | Isoform TGN46 of Trans-Golgi network integral membrane protein 2 OS=Homo sapiens GN=TGOLN2 - [TGON2_HUMAN] | 0.874 |
| P47755 | *CAPZA2* | F-actin-capping protein subunit alpha-2 OS=Homo sapiens GN=CAPZA2 PE=1 SV=3 - [CAZA2_HUMAN] | 1.034 |
| Q9H204 | *MED28* | Mediator of RNA polymerase II transcription subunit 28 OS=Homo sapiens GN=MED28 PE=1 SV=1 - [MED28_HUMAN] | 1.076 |
| Q9BTE3 | *MCMBP* | Isoform 2 of Mini-chromosome maintenance complex-binding protein OS=Homo sapiens GN=MCMBP - [MCMBP_HUMAN] | 1.114 |
| Q13442 | *PDAP1* | 28 kDa heat- and acid-stable phosphoprotein OS=Homo sapiens GN=PDAP1 PE=1 SV=1 - [HAP28_HUMAN] | 1.045 |
| P35659 | *DEK* | Protein DEK OS=Homo sapiens GN=DEK PE=1 SV=1 - [DEK_HUMAN] | 1.080 |
| P69849 | *NOMO3* | Nodal modulator 3 OS=Homo sapiens GN=NOMO3 PE=2 SV=2 - [NOMO3_HUMAN] | 1.079 |
| Q9UHY1 | *NRBP1* | Nuclear receptor-binding protein OS=Homo sapiens GN=NRBP1 PE=1 SV=1 - [NRBP_HUMAN] | 0.955 |
| P63279 | *UBE2I* | SUMO-conjugating enzyme UBC9 OS=Homo sapiens GN=UBE2I PE=1 SV=1 - [UBC9_HUMAN] | 1.103 |
| Q16576 | *RBBP7* | Histone-binding protein RBBP7 OS=Homo sapiens GN=RBBP7 PE=1 SV=1 - [RBBP7_HUMAN] | 0.930 |
| P34947 | *GRK5* | G protein-coupled receptor kinase 5 OS=Homo sapiens GN=GRK5 PE=1 SV=1 - [GRK5_HUMAN] | 0.885 |
| Q15582 | *TGFBI* | Transforming growth factor-beta-induced protein ig-h3 OS=Homo sapiens GN=TGFBI PE=1 SV=1 - [BGH3_HUMAN] | 0.813 |
| Q16630 | *CPSF6* | Isoform 3 of Cleavage and polyadenylation specificity factor subunit 6 OS=Homo sapiens GN=CPSF6 - [CPSF6_HUMAN] | 1.044 |
| Q96CV9 | *OPTN* | Isoform 3 of Optineurin OS=Homo sapiens GN=OPTN - [OPTN_HUMAN] | 1.098 |
| Q96ID5 | *IGSF21* | Immunoglobulin superfamily member 21 OS=Homo sapiens GN=IGSF21 PE=2 SV=1 - [IGS21_HUMAN] | 1.189 |
| Q96AE7 | *TTC17* | Tetratricopeptide repeat protein 17 OS=Homo sapiens GN=TTC17 PE=1 SV=1 - [TTC17_HUMAN] | 0.936 |
| O14524 | *TMEM194A* | Isoform 2 of Transmembrane protein 194A OS=Homo sapiens GN=TMEM194A - [T194A_HUMAN] | 0.942 |
| Q99447 | *PCYT2* | Ethanolamine-phosphate cytidylyltransferase OS=Homo sapiens GN=PCYT2 PE=1 SV=1 - [PCY2_HUMAN] | 0.958 |
| Q96IJ6 | *GMPPA* | Mannose-1-phosphate guanyltransferase alpha OS=Homo sapiens GN=GMPPA PE=1 SV=1 - [GMPPA_HUMAN] | 0.947 |
| Q9BWM7 | *SFXN3* | Sideroflexin-3 OS=Homo sapiens GN=SFXN3 PE=2 SV=2 - [SFXN3_HUMAN] | 0.916 |
| P08579 | *SNRPB2* | U2 small nuclear ribonucleoprotein B'' OS=Homo sapiens GN=SNRPB2 PE=1 SV=1 - [RU2B_HUMAN] | 0.975 |
| Q9H981 | *ACTR8* | Actin-related protein 8 OS=Homo sapiens GN=ACTR8 PE=1 SV=2 - [ARP8_HUMAN] | 0.936 |
| Q9NR45 | *NANS* | Sialic acid synthase OS=Homo sapiens GN=NANS PE=1 SV=2 - [SIAS_HUMAN] | 0.901 |
| P51649 | *ALDH5A1* | Succinate-semialdehyde dehydrogenase, mitochondrial OS=Homo sapiens GN=ALDH5A1 PE=1 SV=2 - [SSDH_HUMAN] | 0.898 |
| Q9C0H9 | *#N/A* | Isoform 2 of SRC kinase signaling inhibitor 1 OS=Homo sapiens GN=SRCIN1 - [SRCN1_HUMAN] | 1.247 |
| Q8IV36 | *HID1* | Isoform 3 of UPF0663 transmembrane protein C17orf28 OS=Homo sapiens GN=C17orf28 - [CQ028_HUMAN] | 1.098 |
| Q16181 | *SEPT7* | Isoform 2 of Septin-7 OS=Homo sapiens GN=SEPT7 - [SEPT7_HUMAN] | 0.934 |
| Q8N8A2 | *ANKRD44* | Isoform 5 of Serine/threonine-protein phosphatase 6 regulatory ankyrin repeat subunit B OS=Homo sapiens GN=ANKRD44 - [ANR44_HUMAN] | 1.106 |
| Q9BW85 | *CCDC94* | Coiled-coil domain-containing protein 94 OS=Homo sapiens GN=CCDC94 PE=1 SV=1 - [CCD94_HUMAN] | 1.096 |
| Q99808 | *SLC29A1* | Equilibrative nucleoside transporter 1 OS=Homo sapiens GN=SLC29A1 PE=1 SV=3 - [S29A1_HUMAN] | 0.908 |
| Q15005 | *SPCS2* | Signal peptidase complex subunit 2 OS=Homo sapiens GN=SPCS2 PE=1 SV=3 - [SPCS2_HUMAN] | 1.056 |
| Q86X29 | *LSR* | Isoform 2 of Lipolysis-stimulated lipoprotein receptor OS=Homo sapiens GN=LSR - [LSR_HUMAN] | 0.857 |
| Q86X95 | *CIR1* | Isoform 2 of Corepressor interacting with RBPJ 1 OS=Homo sapiens GN=CIR1 - [CIR1_HUMAN] | 0.882 |
| P23497 | *SP100* | Isoform SpAlt-C of Nuclear autoantigen Sp-100 OS=Homo sapiens GN=SP100 - [SP100_HUMAN] | 1.135 |
| Q9BRK5 | *SDF4* | 45 kDa calcium-binding protein OS=Homo sapiens GN=SDF4 PE=1 SV=1 - [CAB45_HUMAN] | 0.927 |
| P61011 | *SRP54* | Signal recognition particle 54 kDa protein OS=Homo sapiens GN=SRP54 PE=1 SV=1 - [SRP54_HUMAN] | 1.032 |
| O14672 | *ADAM10* | Disintegrin and metalloproteinase domain-containing protein 10 OS=Homo sapiens GN=ADAM10 PE=1 SV=1 - [ADA10_HUMAN] | 0.891 |
| Q7Z2K8 | *GPRIN1* | G protein-regulated inducer of neurite outgrowth 1 OS=Homo sapiens GN=GPRIN1 PE=1 SV=2 - [GRIN1_HUMAN] | 1.137 |
| P51159 | *RAB27A* | Ras-related protein Rab-27A OS=Homo sapiens GN=RAB27A PE=1 SV=3 - [RB27A_HUMAN] | 0.793 |
| P27448 | *MARK3* | Isoform 5 of MAP/microtubule affinity-regulating kinase 3 OS=Homo sapiens GN=MARK3 - [MARK3_HUMAN] | 0.957 |
| Q8IZP0 | *ABI1* | Isoform 10 of Abl interactor 1 OS=Homo sapiens GN=ABI1 - [ABI1_HUMAN] | 1.033 |
| Q9Y282 | *ERGIC3* | Isoform 2 of Endoplasmic reticulum-Golgi intermediate compartment protein 3 OS=Homo sapiens GN=ERGIC3 - [ERGI3_HUMAN] | 0.942 |
| Q9NWS8 | *RMND1* | Isoform 2 of Required for meiotic nuclear division protein 1 homolog OS=Homo sapiens GN=RMND1 - [RMND1_HUMAN] | 0.917 |
| Q8N1G2 | *CMTR1* | Cap-specific mRNA (nucleoside-2'-O-)-methyltransferase 1 OS=Homo sapiens GN=FTSJD2 PE=1 SV=1 - [MTR1_HUMAN] | 1.106 |
| Q9Y2H1 | *STK38L* | Serine/threonine-protein kinase 38-like OS=Homo sapiens GN=STK38L PE=1 SV=3 - [ST38L_HUMAN] | 1.044 |
| P09110 | *ACAA1* | 3-ketoacyl-CoA thiolase, peroxisomal OS=Homo sapiens GN=ACAA1 PE=1 SV=2 - [THIK_HUMAN] | 1.122 |
| Q9Y230 | *RUVBL2* | RuvB-like 2 OS=Homo sapiens GN=RUVBL2 PE=1 SV=3 - [RUVB2_HUMAN] | 1.066 |
| Q9ULJ6 | *ZMIZ1* | Isoform 2 of Zinc finger MIZ domain-containing protein 1 OS=Homo sapiens GN=ZMIZ1 - [ZMIZ1_HUMAN] | 0.947 |
| P51911 | *CNN1* | Calponin-1 OS=Homo sapiens GN=CNN1 PE=1 SV=2 - [CNN1_HUMAN] | 0.775 |
| Q96PV6 | *LENG8* | Leukocyte receptor cluster member 8 OS=Homo sapiens GN=LENG8 PE=1 SV=2 - [LENG8_HUMAN] | 0.917 |
| P55060 | *CSE1L* | Isoform 3 of Exportin-2 OS=Homo sapiens GN=CSE1L - [XPO2_HUMAN] | 1.033 |
| A0JNW5 | *UHRF1BP1L* | UHRF1-binding protein 1-like OS=Homo sapiens GN=UHRF1BP1L PE=1 SV=2 - [UH1BL_HUMAN] | 0.932 |
| Q9UKV8 | *AGO2* | Isoform 2 of Protein argonaute-2 OS=Homo sapiens GN=EIF2C2 - [AGO2_HUMAN] | 1.097 |
| P24557 | *TBXAS1* | Thromboxane-A synthase OS=Homo sapiens GN=TBXAS1 PE=1 SV=3 - [THAS_HUMAN] | 0.887 |
| Q9Y3D7 | *CORO7* | Mitochondrial import inner membrane translocase subunit TIM16 OS=Homo sapiens GN=PAM16 PE=1 SV=2 - [TIM16_HUMAN] | 0.933 |
| P31146 | *CORO1A* | Coronin-1A OS=Homo sapiens GN=CORO1A PE=1 SV=4 - [COR1A_HUMAN] | 1.059 |
| P11166 | *SLC2A1* | Solute carrier family 2, facilitated glucose transporter member 1 OS=Homo sapiens GN=SLC2A1 PE=1 SV=2 - [GTR1_HUMAN] | 0.750 |
| Q96TA2 | *YME1L1* | Isoform 2 of ATP-dependent zinc metalloprotease YME1L1 OS=Homo sapiens GN=YME1L1 - [YMEL1_HUMAN] | 1.047 |
| Q7Z7G0 | *ABI3BP* | Target of Nesh-SH3 OS=Homo sapiens GN=ABI3BP PE=1 SV=1 - [TARSH_HUMAN] | 0.712 |
| Q14157 | *UBAP2L* | Isoform 4 of Ubiquitin-associated protein 2-like OS=Homo sapiens GN=UBAP2L - [UBP2L_HUMAN] | 1.059 |
| Q96AE4 | *FUBP1* | Far upstream element-binding protein 1 OS=Homo sapiens GN=FUBP1 PE=1 SV=3 - [FUBP1_HUMAN] | 0.953 |
| P09661 | *SNRPA1* | U2 small nuclear ribonucleoprotein A' OS=Homo sapiens GN=SNRPA1 PE=1 SV=2 - [RU2A_HUMAN] | 1.032 |
| O14879 | *IFIT3* | Interferon-induced protein with tetratricopeptide repeats 3 OS=Homo sapiens GN=IFIT3 PE=1 SV=1 - [IFIT3_HUMAN] | 1.219 |
| P54920 | *NAPA* | Alpha-soluble NSF attachment protein OS=Homo sapiens GN=NAPA PE=1 SV=3 - [SNAA_HUMAN] | 0.965 |
| O60256 | *PRPSAP2* | Phosphoribosyl pyrophosphate synthase-associated protein 2 OS=Homo sapiens GN=PRPSAP2 PE=1 SV=1 - [KPRB_HUMAN] | 0.966 |
| P49959 | *MRE11A* | Double-strand break repair protein MRE11A OS=Homo sapiens GN=MRE11A PE=1 SV=3 - [MRE11_HUMAN] | 1.060 |
| P62263 | *RPS14* | 40S ribosomal protein S14 OS=Homo sapiens GN=RPS14 PE=1 SV=3 - [RS14_HUMAN] | 1.087 |
| Q13505 | *MTX1* | Isoform 3 of Metaxin-1 OS=Homo sapiens GN=MTX1 - [MTX1_HUMAN] | 1.061 |
| Q9H0X4 | *ITFG3* | Protein ITFG3 OS=Homo sapiens GN=ITFG3 PE=1 SV=1 - [ITFG3_HUMAN] | 0.925 |
| Q96I15 | *SCLY* | Selenocysteine lyase OS=Homo sapiens GN=SCLY PE=1 SV=2 - [SCLY_HUMAN] | 0.947 |
| Q9NV70 | *EXOC1* | Isoform 2 of Exocyst complex component 1 OS=Homo sapiens GN=EXOC1 - [EXOC1_HUMAN] | 0.966 |
| P15880 | *RPS2* | 40S ribosomal protein S2 OS=Homo sapiens GN=RPS2 PE=1 SV=2 - [RS2_HUMAN] | 0.965 |
| P25685 | *DNAJB1* | DnaJ homolog subfamily B member 1 OS=Homo sapiens GN=DNAJB1 PE=1 SV=4 - [DNJB1_HUMAN] | 1.093 |
| Q02447 | *SP3* | Isoform 4 of Transcription factor Sp3 OS=Homo sapiens GN=SP3 - [SP3_HUMAN] | 0.969 |
| Q12849 | *GRSF1* | G-rich sequence factor 1 OS=Homo sapiens GN=GRSF1 PE=1 SV=3 - [GRSF1_HUMAN] | 1.075 |
| Q9P2N5 | *RBM27* | RNA-binding protein 27 OS=Homo sapiens GN=RBM27 PE=1 SV=2 - [RBM27_HUMAN] | 1.047 |
| O75533 | *SF3B1* | Splicing factor 3B subunit 1 OS=Homo sapiens GN=SF3B1 PE=1 SV=3 - [SF3B1_HUMAN] | 0.975 |
| P0C628 | *#N/A* | Olfactory receptor 5AC1 OS=Homo sapiens GN=OR5AC1 PE=3 SV=1 - [O5AC1_HUMAN] | 1.077 |
| P00918 | *CA2* | Carbonic anhydrase 2 OS=Homo sapiens GN=CA2 PE=1 SV=2 - [CAH2_HUMAN] | 0.788 |
| Q9UK41 | *VPS28* | Vacuolar protein sorting-associated protein 28 homolog OS=Homo sapiens GN=VPS28 PE=1 SV=1 - [VPS28_HUMAN] | 0.909 |
| Q8N122 | *RPTOR* | Regulatory-associated protein of mTOR OS=Homo sapiens GN=RPTOR PE=1 SV=1 - [RPTOR_HUMAN] | 0.964 |
| Q96FC7 | *PHYHIPL* | Isoform 2 of Phytanoyl-CoA hydroxylase-interacting protein-like OS=Homo sapiens GN=PHYHIPL - [PHIPL_HUMAN] | 1.105 |
| P06703 | *S100A6* | Protein S100-A6 OS=Homo sapiens GN=S100A6 PE=1 SV=1 - [S10A6_HUMAN] | 0.836 |
| O15400 | *STX7* | Isoform 2 of Syntaxin-7 OS=Homo sapiens GN=STX7 - [STX7_HUMAN] | 1.066 |
| Q9BQA1 | *WDR77* | Methylosome protein 50 OS=Homo sapiens GN=WDR77 PE=1 SV=1 - [MEP50_HUMAN] | 1.058 |
| O95425 | *SVIL* | Isoform 2 of Supervillin OS=Homo sapiens GN=SVIL - [SVIL_HUMAN] | 1.138 |
| P19338 | *NCL* | Nucleolin OS=Homo sapiens GN=NCL PE=1 SV=3 - [NUCL_HUMAN] | 1.040 |
| P46063 | *RECQL* | ATP-dependent DNA helicase Q1 OS=Homo sapiens GN=RECQL PE=1 SV=3 - [RECQ1_HUMAN] | 1.061 |
| Q92925 | *SMARCD2* | Isoform 3 of SWI/SNF-related matrix-associated actin-dependent regulator of chromatin subfamily D member 2 OS=Homo sapiens GN=SMARCD2 - [SMRD2_HUMAN] | 1.050 |
| Q9H0W9 | *C11orf54* | Isoform 4 of Ester hydrolase C11orf54 OS=Homo sapiens GN=C11orf54 - [CK054_HUMAN] | 0.923 |
| Q15056 | *EIF4H* | Eukaryotic translation initiation factor 4H OS=Homo sapiens GN=EIF4H PE=1 SV=5 - [IF4H_HUMAN] | 0.975 |
| P25325 | *MPST* | 3-mercaptopyruvate sulfurtransferase OS=Homo sapiens GN=MPST PE=1 SV=3 - [THTM_HUMAN] | 0.868 |
| Q9P2J9 | *PDP2* | [Pyruvate dehydrogenase [acetyl-transferring]]-phosphatase 2, mitochondrial OS=Homo sapiens GN=PDP2 PE=2 SV=2 - [PDP2_HUMAN] | 0.854 |
| P13489 | *RNH1* | Ribonuclease inhibitor OS=Homo sapiens GN=RNH1 PE=1 SV=2 - [RINI_HUMAN] | 0.951 |
| P78536 | *ADAM17* | Disintegrin and metalloproteinase domain-containing protein 17 OS=Homo sapiens GN=ADAM17 PE=1 SV=1 - [ADA17_HUMAN] | 1.051 |
| O75821 | *EIF3G* | Eukaryotic translation initiation factor 3 subunit G OS=Homo sapiens GN=EIF3G PE=1 SV=2 - [EIF3G_HUMAN] | 1.032 |
| Q96B54 | *ZNF428* | Zinc finger protein 428 OS=Homo sapiens GN=ZNF428 PE=1 SV=2 - [ZN428_HUMAN] | 1.073 |
| Q9Y6D5 | *ARFGEF2* | Brefeldin A-inhibited guanine nucleotide-exchange protein 2 OS=Homo sapiens GN=ARFGEF2 PE=1 SV=3 - [BIG2_HUMAN] | 1.100 |
| P11586 | *MTHFD1* | C-1-tetrahydrofolate synthase, cytoplasmic OS=Homo sapiens GN=MTHFD1 PE=1 SV=3 - [C1TC_HUMAN] | 1.055 |
| Q7LBC6 | *KDM3B* | Isoform 2 of Lysine-specific demethylase 3B OS=Homo sapiens GN=KDM3B - [KDM3B_HUMAN] | 0.910 |
| Q15907 | *RAB11B* | Ras-related protein Rab-11B OS=Homo sapiens GN=RAB11B PE=1 SV=4 - [RB11B_HUMAN] | 1.054 |
| P78347 | *GTF2I* | Isoform 2 of General transcription factor II-I OS=Homo sapiens GN=GTF2I - [GTF2I_HUMAN] | 1.052 |
| P09601 | *HMOX1* | Heme oxygenase 1 OS=Homo sapiens GN=HMOX1 PE=1 SV=1 - [HMOX1_HUMAN] | 0.924 |
| Q9Y6G3 | *MRPL42* | 39S ribosomal protein L42, mitochondrial OS=Homo sapiens GN=MRPL42 PE=1 SV=1 - [RM42_HUMAN] | 0.969 |
| Q13084 | *MRPL28* | 39S ribosomal protein L28, mitochondrial OS=Homo sapiens GN=MRPL28 PE=1 SV=4 - [RM28_HUMAN] | 0.962 |
| Q70Z53 | *FRA10AC1* | Isoform 2 of Protein FRA10AC1 OS=Homo sapiens GN=FRA10AC1 - [F10C1_HUMAN] | 1.118 |
| Q9UEU0 | *VTI1B* | Vesicle transport through interaction with t-SNAREs homolog 1B OS=Homo sapiens GN=VTI1B PE=1 SV=3 - [VTI1B_HUMAN] | 1.049 |
| P52434 | *POLR2H* | DNA-directed RNA polymerases I, II, and III subunit RPABC3 OS=Homo sapiens GN=POLR2H PE=1 SV=4 - [RPAB3_HUMAN] | 1.041 |
| Q8IY95 | *TMEM192* | Isoform 2 of Transmembrane protein 192 OS=Homo sapiens GN=TMEM192 - [TM192_HUMAN] | 1.102 |
| O15123 | *ANGPT2* | Isoform 2 of Angiopoietin-2 OS=Homo sapiens GN=ANGPT2 - [ANGP2_HUMAN] | 0.851 |
| O14733 | *MAP2K7* | Dual specificity mitogen-activated protein kinase kinase 7 OS=Homo sapiens GN=MAP2K7 PE=1 SV=2 - [MP2K7_HUMAN] | 0.943 |
| Q93034 | *CUL5* | Cullin-5 OS=Homo sapiens GN=CUL5 PE=1 SV=4 - [CUL5_HUMAN] | 1.034 |
| P22570 | *FDXR* | NADPH:adrenodoxin oxidoreductase, mitochondrial OS=Homo sapiens GN=FDXR PE=1 SV=3 - [ADRO_HUMAN] | 0.876 |
| P49591 | *SARS* | Serine--tRNA ligase, cytoplasmic OS=Homo sapiens GN=SARS PE=1 SV=3 - [SYSC_HUMAN] | 1.050 |
| P12829 | *MYL4* | Myosin light chain 4 OS=Homo sapiens GN=MYL4 PE=1 SV=3 - [MYL4_HUMAN] | 1.444 |
| P49815 | *TSC2* | Isoform 6 of Tuberin OS=Homo sapiens GN=TSC2 - [TSC2_HUMAN] | 1.052 |
| Q86Y46 | *KRT73* | Keratin, type II cytoskeletal 73 OS=Homo sapiens GN=KRT73 PE=1 SV=1 - [K2C73_HUMAN] | 1.102 |
| Q9Y608 | *LRRFIP2* | Isoform 4 of Leucine-rich repeat flightless-interacting protein 2 OS=Homo sapiens GN=LRRFIP2 - [LRRF2_HUMAN] | 0.962 |
| Q4V9L6 | *TMEM119* | Transmembrane protein 119 OS=Homo sapiens GN=TMEM119 PE=2 SV=1 - [TM119_HUMAN] | 1.258 |
| Q12888 | *TP53BP1* | Tumor suppressor p53-binding protein 1 OS=Homo sapiens GN=TP53BP1 PE=1 SV=2 - [TP53B_HUMAN] | 0.936 |
| O75592 | *MYCBP2* | Isoform 2 of Probable E3 ubiquitin-protein ligase MYCBP2 OS=Homo sapiens GN=MYCBP2 - [MYCB2_HUMAN] | 1.051 |
| Q9BYD2 | *MRPL9* | 39S ribosomal protein L9, mitochondrial OS=Homo sapiens GN=MRPL9 PE=1 SV=2 - [RM09_HUMAN] | 0.936 |
| Q04724 | *TLE1* | Transducin-like enhancer protein 1 OS=Homo sapiens GN=TLE1 PE=1 SV=2 - [TLE1_HUMAN] | 1.129 |
| Q9Y237 | *PIN4* | Peptidyl-prolyl cis-trans isomerase NIMA-interacting 4 OS=Homo sapiens GN=PIN4 PE=1 SV=1 - [PIN4_HUMAN] | 1.049 |
| O60784 | *TOM1* | Target of Myb protein 1 OS=Homo sapiens GN=TOM1 PE=1 SV=2 - [TOM1_HUMAN] | 1.099 |
| P00734 | *F2* | Prothrombin OS=Homo sapiens GN=F2 PE=1 SV=2 - [THRB_HUMAN] | 0.890 |
| P41240 | *CSK* | Tyrosine-protein kinase CSK OS=Homo sapiens GN=CSK PE=1 SV=1 - [CSK_HUMAN] | 0.934 |
| Q9UKB3 | *DNAJC12* | DnaJ homolog subfamily C member 12 OS=Homo sapiens GN=DNAJC12 PE=1 SV=1 - [DJC12_HUMAN] | 0.876 |
| P24593 | *IGFBP5* | Insulin-like growth factor-binding protein 5 OS=Homo sapiens GN=IGFBP5 PE=1 SV=1 - [IBP5_HUMAN] | 1.483 |
| Q5TC12 | *ATPAF1* | ATP synthase mitochondrial F1 complex assembly factor 1 OS=Homo sapiens GN=ATPAF1 PE=1 SV=1 - [ATPF1_HUMAN] | 0.917 |
| Q9UHA4 | *LAMTOR3* | Ragulator complex protein LAMTOR3 OS=Homo sapiens GN=LAMTOR3 PE=1 SV=1 - [LTOR3_HUMAN] | 1.085 |
| Q02218 | *OGDH* | 2-oxoglutarate dehydrogenase, mitochondrial OS=Homo sapiens GN=OGDH PE=1 SV=3 - [ODO1_HUMAN] | 0.943 |
| Q9H267 | *VPS33B* | Vacuolar protein sorting-associated protein 33B OS=Homo sapiens GN=VPS33B PE=1 SV=2 - [VP33B_HUMAN] | 1.076 |
| Q12929 | *EPS8* | Epidermal growth factor receptor kinase substrate 8 OS=Homo sapiens GN=EPS8 PE=1 SV=1 - [EPS8_HUMAN] | 0.897 |
| Q96B36 | *AKT1S1* | Isoform 2 of Proline-rich AKT1 substrate 1 OS=Homo sapiens GN=AKT1S1 - [AKTS1_HUMAN] | 1.037 |
| Q9NTJ5 | *SACM1L* | Phosphatidylinositide phosphatase SAC1 OS=Homo sapiens GN=SACM1L PE=1 SV=2 - [SAC1_HUMAN] | 1.063 |
| P62491 | *RAB11A* | Ras-related protein Rab-11A OS=Homo sapiens GN=RAB11A PE=1 SV=3 - [RB11A_HUMAN] | 1.061 |
| O00471 | *EXOC5* | Exocyst complex component 5 OS=Homo sapiens GN=EXOC5 PE=1 SV=1 - [EXOC5_HUMAN] | 1.040 |
| Q9HB90 | *RRAGC* | Ras-related GTP-binding protein C OS=Homo sapiens GN=RRAGC PE=1 SV=1 - [RRAGC_HUMAN] | 1.053 |
| Q9Y295 | *DRG1* | Developmentally-regulated GTP-binding protein 1 OS=Homo sapiens GN=DRG1 PE=1 SV=1 - [DRG1_HUMAN] | 1.032 |
| Q9BRT3 | *MIEN1* | Migration and invasion enhancer 1 OS=Homo sapiens GN=MIEN1 PE=1 SV=1 - [MIEN1_HUMAN] | 1.077 |
| P19525 | *EIF2AK2* | Interferon-induced, double-stranded RNA-activated protein kinase OS=Homo sapiens GN=EIF2AK2 PE=1 SV=2 - [E2AK2_HUMAN] | 1.094 |
| P37198 | *NUP62* | Nuclear pore glycoprotein p62 OS=Homo sapiens GN=NUP62 PE=1 SV=3 - [NUP62_HUMAN] | 1.034 |
| Q15942 | *ZYX* | Zyxin OS=Homo sapiens GN=ZYX PE=1 SV=1 - [ZYX_HUMAN] | 1.092 |
| Q00765 | *REEP5* | Receptor expression-enhancing protein 5 OS=Homo sapiens GN=REEP5 PE=1 SV=3 - [REEP5_HUMAN] | 0.918 |
| P22466 | *GAL* | Galanin peptides OS=Homo sapiens GN=GAL PE=1 SV=3 - [GALA_HUMAN] | 0.735 |
| Q96ST3 | *SIN3A* | Paired amphipathic helix protein Sin3a OS=Homo sapiens GN=SIN3A PE=1 SV=2 - [SIN3A_HUMAN] | 1.033 |
| Q09161 | *NCBP1* | Nuclear cap-binding protein subunit 1 OS=Homo sapiens GN=NCBP1 PE=1 SV=1 - [NCBP1_HUMAN] | 1.040 |
| P49257 | *LMAN1* | Protein ERGIC-53 OS=Homo sapiens GN=LMAN1 PE=1 SV=2 - [LMAN1_HUMAN] | 0.937 |
| Q9NRX4 | *PHPT1* | Isoform 2 of 14 kDa phosphohistidine phosphatase OS=Homo sapiens GN=PHPT1 - [PHP14_HUMAN] | 0.961 |
| P04040 | *CAT* | Catalase OS=Homo sapiens GN=CAT PE=1 SV=3 - [CATA_HUMAN] | 0.841 |
| Q9BW30 | *TPPP3* | Tubulin polymerization-promoting protein family member 3 OS=Homo sapiens GN=TPPP3 PE=1 SV=1 - [TPPP3_HUMAN] | 1.305 |
| O00469 | *PLOD2* | Procollagen-lysine,2-oxoglutarate 5-dioxygenase 2 OS=Homo sapiens GN=PLOD2 PE=1 SV=2 - [PLOD2_HUMAN] | 1.164 |
| P63010 | *AP2B1* | AP-2 complex subunit beta OS=Homo sapiens GN=AP2B1 PE=1 SV=1 - [AP2B1_HUMAN] | 0.966 |
| Q9H6K5 | *#N/A* | Putative uncharacterized protein FLJ22184 OS=Homo sapiens PE=1 SV=1 - [YS027_HUMAN] | 0.933 |
| Q96E09 | *FAM122A* | Protein FAM122A OS=Homo sapiens GN=FAM122A PE=1 SV=1 - [F122A_HUMAN] | 1.069 |
| O94985 | *CLSTN1* | Isoform 2 of Calsyntenin-1 OS=Homo sapiens GN=CLSTN1 - [CSTN1_HUMAN] | 1.135 |
| P20810 | *CAST* | Isoform 3 of Calpastatin OS=Homo sapiens GN=CAST - [ICAL_HUMAN] | 1.104 |
| Q96G03 | *PGM2* | Phosphoglucomutase-2 OS=Homo sapiens GN=PGM2 PE=1 SV=4 - [PGM2_HUMAN] | 0.940 |
| Q8N9U0 | *TC2N* | Isoform 2 of Tandem C2 domains nuclear protein OS=Homo sapiens GN=TC2N - [TAC2N_HUMAN] | 0.832 |
| P22626 | *HNRNPA2B1* | Isoform A2 of Heterogeneous nuclear ribonucleoproteins A2/B1 OS=Homo sapiens GN=HNRNPA2B1 - [ROA2_HUMAN] | 0.938 |
| Q9H4A4 | *RNPEP* | Aminopeptidase B OS=Homo sapiens GN=RNPEP PE=1 SV=2 - [AMPB_HUMAN] | 0.955 |
| Q9Y4W6 | *AFG3L2* | AFG3-like protein 2 OS=Homo sapiens GN=AFG3L2 PE=1 SV=2 - [AFG32_HUMAN] | 1.085 |
| P19971 | *TYMP* | Thymidine phosphorylase OS=Homo sapiens GN=TYMP PE=1 SV=2 - [TYPH_HUMAN] | 1.160 |
| P84090 | *ERH* | Enhancer of rudimentary homolog OS=Homo sapiens GN=ERH PE=1 SV=1 - [ERH_HUMAN] | 0.970 |
| Q92973 | *TNPO1* | Isoform 2 of Transportin-1 OS=Homo sapiens GN=TNPO1 - [TNPO1_HUMAN] | 1.059 |
| Q8IYB3 | *SRRM1* | Isoform 2 of Serine/arginine repetitive matrix protein 1 OS=Homo sapiens GN=SRRM1 - [SRRM1_HUMAN] | 1.059 |
| Q12797 | *ASPH* | Aspartyl/asparaginyl beta-hydroxylase OS=Homo sapiens GN=ASPH PE=1 SV=3 - [ASPH_HUMAN] | 1.104 |
| P07437 | *TUBB* | Tubulin beta chain OS=Homo sapiens GN=TUBB PE=1 SV=2 - [TBB5_HUMAN] | 1.042 |
| Q8IWS0 | *PHF6* | Isoform 2 of PHD finger protein 6 OS=Homo sapiens GN=PHF6 - [PHF6_HUMAN] | 1.056 |
| P50570 | *DNM2* | Isoform 2 of Dynamin-2 OS=Homo sapiens GN=DNM2 - [DYN2_HUMAN] | 1.060 |
| Q13151 | *HNRNPA0* | Heterogeneous nuclear ribonucleoprotein A0 OS=Homo sapiens GN=HNRNPA0 PE=1 SV=1 - [ROA0_HUMAN] | 0.941 |
| Q9Y2I8 | *WDR37* | WD repeat-containing protein 37 OS=Homo sapiens GN=WDR37 PE=2 SV=2 - [WDR37_HUMAN] | 1.040 |
| Q9BS26 | *ERP44* | Endoplasmic reticulum resident protein 44 OS=Homo sapiens GN=ERP44 PE=1 SV=1 - [ERP44_HUMAN] | 1.051 |
| Q9P2G1 | *ANKIB1* | Ankyrin repeat and IBR domain-containing protein 1 OS=Homo sapiens GN=ANKIB1 PE=1 SV=3 - [AKIB1_HUMAN] | 0.943 |
| P60900 | *PSMA6* | Proteasome subunit alpha type-6 OS=Homo sapiens GN=PSMA6 PE=1 SV=1 - [PSA6_HUMAN] | 1.021 |
| Q12906 | *ILF3* | Isoform 5 of Interleukin enhancer-binding factor 3 OS=Homo sapiens GN=ILF3 - [ILF3_HUMAN] | 1.033 |
| Q8TEA7 | *TBCK* | Isoform 3 of TBC domain-containing protein kinase-like protein OS=Homo sapiens GN=TBCK - [TBCK_HUMAN] | 0.970 |
| Q16851 | *UGP2* | Isoform 2 of UTP--glucose-1-phosphate uridylyltransferase OS=Homo sapiens GN=UGP2 - [UGPA_HUMAN] | 0.953 |
| Q14195 | *DPYSL3* | Dihydropyrimidinase-related protein 3 OS=Homo sapiens GN=DPYSL3 PE=1 SV=1 - [DPYL3_HUMAN] | 0.920 |
| P10253 | *GAA* | Lysosomal alpha-glucosidase OS=Homo sapiens GN=GAA PE=1 SV=4 - [LYAG_HUMAN] | 1.122 |
| Q9UJX2 | *CDC23* | Isoform 3 of Cell division cycle protein 23 homolog OS=Homo sapiens GN=CDC23 - [CDC23_HUMAN] | 1.051 |
| Q14696 | *MESDC2* | LDLR chaperone MESD OS=Homo sapiens GN=MESDC2 PE=1 SV=2 - [MESD_HUMAN] | 0.938 |
| Q8IV08 | *PLD3* | Phospholipase D3 OS=Homo sapiens GN=PLD3 PE=1 SV=1 - [PLD3_HUMAN] | 0.882 |
| P55008 | *AIF1* | Allograft inflammatory factor 1 OS=Homo sapiens GN=AIF1 PE=1 SV=1 - [AIF1_HUMAN] | 0.902 |
| P16278 | *GLB1* | Isoform 2 of Beta-galactosidase OS=Homo sapiens GN=GLB1 - [BGAL_HUMAN] | 0.944 |
| Q9NX24 | *NHP2* | H/ACA ribonucleoprotein complex subunit 2 OS=Homo sapiens GN=NHP2 PE=1 SV=1 - [NHP2_HUMAN] | 0.936 |
| Q15818 | *NPTX1* | Neuronal pentraxin-1 OS=Homo sapiens GN=NPTX1 PE=2 SV=2 - [NPTX1_HUMAN] | 0.755 |
| Q9BPX6 | *MICU1* | Isoform 2 of Calcium uptake protein 1, mitochondrial OS=Homo sapiens GN=MICU1 - [MICU1_HUMAN] | 0.944 |
| Q8N4V1 | *MMGT1* | Membrane magnesium transporter 1 OS=Homo sapiens GN=MMGT1 PE=1 SV=1 - [MMGT1_HUMAN] | 0.950 |
| Q02790 | *FKBP4* | Peptidyl-prolyl cis-trans isomerase FKBP4 OS=Homo sapiens GN=FKBP4 PE=1 SV=3 - [FKBP4_HUMAN] | 1.063 |
| O14639 | *ABLIM1* | Isoform 2 of Actin-binding LIM protein 1 OS=Homo sapiens GN=ABLIM1 - [ABLM1_HUMAN] | 1.114 |
| O75152 | *ZC3H11A* | Zinc finger CCCH domain-containing protein 11A OS=Homo sapiens GN=ZC3H11A PE=1 SV=3 - [ZC11A_HUMAN] | 0.951 |
| P10314 | *HLA-A* | HLA class I histocompatibility antigen, A-32 alpha chain OS=Homo sapiens GN=HLA-A PE=2 SV=2 - [1A32_HUMAN] | 1.326 |
| Q2TAA2 | *IAH1* | Isoamyl acetate-hydrolyzing esterase 1 homolog OS=Homo sapiens GN=IAH1 PE=1 SV=1 - [IAH1_HUMAN] | 1.070 |
| P16083 | *NQO2* | Ribosyldihydronicotinamide dehydrogenase [quinone] OS=Homo sapiens GN=NQO2 PE=1 SV=5 - [NQO2_HUMAN] | 1.228 |
| O00487 | *PSMD14* | 26S proteasome non-ATPase regulatory subunit 14 OS=Homo sapiens GN=PSMD14 PE=1 SV=1 - [PSDE_HUMAN] | 0.933 |
| P15586 | *GNS* | N-acetylglucosamine-6-sulfatase OS=Homo sapiens GN=GNS PE=1 SV=3 - [GNS_HUMAN] | 1.130 |
| Q66K74 | *MAP1S* | Microtubule-associated protein 1S OS=Homo sapiens GN=MAP1S PE=1 SV=2 - [MAP1S_HUMAN] | 1.055 |
| P07919 | *UQCRH* | Cytochrome b-c1 complex subunit 6, mitochondrial OS=Homo sapiens GN=UQCRH PE=1 SV=2 - [QCR6_HUMAN] | 1.084 |
| Q13155 | *AIMP2* | Aminoacyl tRNA synthase complex-interacting multifunctional protein 2 OS=Homo sapiens GN=AIMP2 PE=1 SV=2 - [AIMP2_HUMAN] | 1.041 |
| P11177 | *PDHB* | Isoform 2 of Pyruvate dehydrogenase E1 component subunit beta, mitochondrial OS=Homo sapiens GN=PDHB - [ODPB_HUMAN] | 0.953 |
| Q03188 | *CENPC* | Centromere protein C 1 OS=Homo sapiens GN=CENPC1 PE=1 SV=2 - [CENPC_HUMAN] | 1.050 |
| O43390 | *HNRNPR* | Heterogeneous nuclear ribonucleoprotein R OS=Homo sapiens GN=HNRNPR PE=1 SV=1 - [HNRPR_HUMAN] | 1.040 |
| Q06210 | *GFPT1* | Isoform 2 of Glucosamine--fructose-6-phosphate aminotransferase [isomerizing] 1 OS=Homo sapiens GN=GFPT1 - [GFPT1_HUMAN] | 0.934 |
| P06681 | *C2* | Complement C2 OS=Homo sapiens GN=C2 PE=1 SV=2 - [CO2_HUMAN] | 0.915 |
| Q08378 | *GOLGA3* | Isoform 2 of Golgin subfamily A member 3 OS=Homo sapiens GN=GOLGA3 - [GOGA3_HUMAN] | 1.060 |
| Q8WXH0 | *SYNE2* | Nesprin-2 OS=Homo sapiens GN=SYNE2 PE=1 SV=3 - [SYNE2_HUMAN] | 1.076 |
| Q9BTT6 | *LRRC1* | Leucine-rich repeat-containing protein 1 OS=Homo sapiens GN=LRRC1 PE=1 SV=1 - [LRRC1_HUMAN] | 1.087 |
| Q92878 | *RAD50* | DNA repair protein RAD50 OS=Homo sapiens GN=RAD50 PE=1 SV=1 - [RAD50_HUMAN] | 1.043 |
| P11172 | *UMPS* | Isoform 2 of Uridine 5'-monophosphate synthase OS=Homo sapiens GN=UMPS - [UMPS_HUMAN] | 0.959 |
| Q53GQ0 | *HSD17B12* | Estradiol 17-beta-dehydrogenase 12 OS=Homo sapiens GN=HSD17B12 PE=1 SV=2 - [DHB12_HUMAN] | 0.939 |
| Q96EK5 | *KIAA1279* | KIF1-binding protein OS=Homo sapiens GN=KIAA1279 PE=1 SV=1 - [KBP_HUMAN] | 0.936 |
| P18615 | *NELFE* | Negative elongation factor E OS=Homo sapiens GN=RDBP PE=1 SV=3 - [NELFE_HUMAN] | 1.089 |
| P35249 | *RFC4* | Replication factor C subunit 4 OS=Homo sapiens GN=RFC4 PE=1 SV=2 - [RFC4_HUMAN] | 1.055 |
| Q6GYQ0 | *RALGAPA1* | Isoform 4 of Ral GTPase-activating protein subunit alpha-1 OS=Homo sapiens GN=RALGAPA1 - [RGPA1_HUMAN] | 1.062 |
| Q9NR28 | *DIABLO* | Isoform 2 of Diablo homolog, mitochondrial OS=Homo sapiens GN=DIABLO - [DBLOH_HUMAN] | 0.960 |
| Q8NFH4 | *NUP37* | Nucleoporin Nup37 OS=Homo sapiens GN=NUP37 PE=1 SV=1 - [NUP37_HUMAN] | 0.956 |
| Q9HCE5 | *METTL14* | Methyltransferase-like protein 14 OS=Homo sapiens GN=METTL14 PE=1 SV=2 - [MTL14_HUMAN] | 0.943 |
| Q9P2K3 | *RCOR3* | Isoform 2 of REST corepressor 3 OS=Homo sapiens GN=RCOR3 - [RCOR3_HUMAN] | 0.942 |
| Q0VF96 | *CGNL1* | Cingulin-like protein 1 OS=Homo sapiens GN=CGNL1 PE=1 SV=2 - [CGNL1_HUMAN] | 0.825 |
| Q96EC8 | *YIPF6* | Protein YIPF6 OS=Homo sapiens GN=YIPF6 PE=2 SV=2 - [YIPF6_HUMAN] | 1.080 |
| Q9BT22 | *ALG1* | Chitobiosyldiphosphodolichol beta-mannosyltransferase OS=Homo sapiens GN=ALG1 PE=1 SV=2 - [ALG1_HUMAN] | 1.075 |
| Q9BXN1 | *ASPN* | Asporin OS=Homo sapiens GN=ASPN PE=1 SV=2 - [ASPN_HUMAN] | 0.797 |
| Q14156 | *EFR3A* | Isoform 3 of Protein EFR3 homolog A OS=Homo sapiens GN=EFR3A - [EFR3A_HUMAN] | 0.929 |
| P55036 | *PSMD4* | Isoform Rpn10E of 26S proteasome non-ATPase regulatory subunit 4 OS=Homo sapiens GN=PSMD4 - [PSMD4_HUMAN] | 0.909 |
| P68366 | *TUBA4A* | Tubulin alpha-4A chain OS=Homo sapiens GN=TUBA4A PE=1 SV=1 - [TBA4A_HUMAN] | 1.158 |
| Q9P016 | *THYN1* | Isoform 2 of Thymocyte nuclear protein 1 OS=Homo sapiens GN=THYN1 - [THYN1_HUMAN] | 0.923 |
| P46940 | *IQGAP1* | Ras GTPase-activating-like protein IQGAP1 OS=Homo sapiens GN=IQGAP1 PE=1 SV=1 - [IQGA1_HUMAN] | 0.929 |
| P36578 | *RPL4* | 60S ribosomal protein L4 OS=Homo sapiens GN=RPL4 PE=1 SV=5 - [RL4_HUMAN] | 1.036 |
| Q15057 | *ACAP2* | Arf-GAP with coiled-coil, ANK repeat and PH domain-containing protein 2 OS=Homo sapiens GN=ACAP2 PE=1 SV=3 - [ACAP2_HUMAN] | 0.965 |
| Q5VTR2 | *RNF20* | E3 ubiquitin-protein ligase BRE1A OS=Homo sapiens GN=RNF20 PE=1 SV=2 - [BRE1A_HUMAN] | 1.038 |
| P08397 | *HMBS* | Isoform 2 of Porphobilinogen deaminase OS=Homo sapiens GN=HMBS - [HEM3_HUMAN] | 1.097 |
| O94919 | *ENDOD1* | Endonuclease domain-containing 1 protein OS=Homo sapiens GN=ENDOD1 PE=1 SV=2 - [ENDD1_HUMAN] | 0.916 |
| P29350 | *PTPN6* | Tyrosine-protein phosphatase non-receptor type 6 OS=Homo sapiens GN=PTPN6 PE=1 SV=1 - [PTN6_HUMAN] | 0.970 |
| Q96FZ7 | *CHMP6* | Charged multivesicular body protein 6 OS=Homo sapiens GN=CHMP6 PE=1 SV=3 - [CHMP6_HUMAN] | 1.038 |
| Q9NQ50 | *MRPL40* | 39S ribosomal protein L40, mitochondrial OS=Homo sapiens GN=MRPL40 PE=1 SV=1 - [RM40_HUMAN] | 0.952 |
| Q7KZI7 | *MARK2* | Isoform 10 of Serine/threonine-protein kinase MARK2 OS=Homo sapiens GN=MARK2 - [MARK2_HUMAN] | 0.955 |
| P41226 | *UBA7* | Ubiquitin-like modifier-activating enzyme 7 OS=Homo sapiens GN=UBA7 PE=1 SV=2 - [UBA7_HUMAN] | 1.073 |
| Q9NQX3 | *GPHN* | Gephyrin OS=Homo sapiens GN=GPHN PE=1 SV=1 - [GEPH_HUMAN] | 0.926 |
| Q9Y4P3 | *TBL2* | Transducin beta-like protein 2 OS=Homo sapiens GN=TBL2 PE=1 SV=1 - [TBL2_HUMAN] | 0.957 |
| O14497 | *ARID1A* | AT-rich interactive domain-containing protein 1A OS=Homo sapiens GN=ARID1A PE=1 SV=3 - [ARI1A_HUMAN] | 1.057 |
| P54725 | *RAD23A* | UV excision repair protein RAD23 homolog A OS=Homo sapiens GN=RAD23A PE=1 SV=1 - [RD23A_HUMAN] | 1.050 |
| O15226 | *NKRF* | NF-kappa-B-repressing factor OS=Homo sapiens GN=NKRF PE=1 SV=2 - [NKRF_HUMAN] | 0.962 |
| Q15165 | *PON2* | Isoform 3 of Serum paraoxonase/arylesterase 2 OS=Homo sapiens GN=PON2 - [PON2_HUMAN] | 1.105 |
| Q9Y678 | *COPG1* | Coatomer subunit gamma OS=Homo sapiens GN=COPG PE=1 SV=1 - [COPG_HUMAN] | 1.085 |
| O75150 | *RNF40* | Isoform 3 of E3 ubiquitin-protein ligase BRE1B OS=Homo sapiens GN=RNF40 - [BRE1B_HUMAN] | 1.031 |
| Q96HN2 | *AHCYL2* | Isoform 2 of Putative adenosylhomocysteinase 3 OS=Homo sapiens GN=AHCYL2 - [SAHH3_HUMAN] | 1.031 |
| Q96AJ9 | *VTI1A* | Isoform 1 of Vesicle transport through interaction with t-SNAREs homolog 1A OS=Homo sapiens GN=VTI1A - [VTI1A_HUMAN] | 0.958 |
| Q9UJW0 | *DCTN4* | Isoform 2 of Dynactin subunit 4 OS=Homo sapiens GN=DCTN4 - [DCTN4_HUMAN] | 0.904 |
| P51398 | *DAP3* | 28S ribosomal protein S29, mitochondrial OS=Homo sapiens GN=DAP3 PE=1 SV=1 - [RT29_HUMAN] | 0.962 |
| P09914 | *IFIT1* | Interferon-induced protein with tetratricopeptide repeats 1 OS=Homo sapiens GN=IFIT1 PE=1 SV=2 - [IFIT1_HUMAN] | 1.186 |
| P20336 | *RAB3A* | Ras-related protein Rab-3A OS=Homo sapiens GN=RAB3A PE=1 SV=1 - [RAB3A_HUMAN] | 0.911 |
| Q9NUQ3 | *TXLNG* | Gamma-taxilin OS=Homo sapiens GN=TXLNG PE=1 SV=2 - [TXLNG_HUMAN] | 0.948 |
| P52735 | *VAV2* | Isoform 3 of Guanine nucleotide exchange factor VAV2 OS=Homo sapiens GN=VAV2 - [VAV2_HUMAN] | 0.935 |
| P07992 | *ERCC1* | DNA excision repair protein ERCC-1 OS=Homo sapiens GN=ERCC1 PE=1 SV=1 - [ERCC1_HUMAN] | 1.097 |
| Q01433 | *AMPD2* | Isoform Ex1A-3 of AMP deaminase 2 OS=Homo sapiens GN=AMPD2 - [AMPD2_HUMAN] | 0.938 |
| Q96PQ0 | *SORCS2* | VPS10 domain-containing receptor SorCS2 OS=Homo sapiens GN=SORCS2 PE=1 SV=3 - [SORC2_HUMAN] | 1.164 |
| P98196 | *ATP11A* | Probable phospholipid-transporting ATPase IH OS=Homo sapiens GN=ATP11A PE=2 SV=3 - [AT11A_HUMAN] | 1.113 |
| Q96RY7 | *IFT140* | Intraflagellar transport protein 140 homolog OS=Homo sapiens GN=IFT140 PE=1 SV=1 - [IF140_HUMAN] | 0.956 |
| P62820 | *RAB1A* | Ras-related protein Rab-1A OS=Homo sapiens GN=RAB1A PE=1 SV=3 - [RAB1A_HUMAN] | 0.921 |
| Q15007 | *WTAP* | Isoform 2 of Pre-mRNA-splicing regulator WTAP OS=Homo sapiens GN=WTAP - [FL2D_HUMAN] | 1.017 |
| Q9Y4B6 | *VPRBP* | Isoform 3 of Protein VPRBP OS=Homo sapiens GN=VPRBP - [VPRBP_HUMAN] | 1.054 |
| Q53H47 | *SETMAR* | Histone-lysine N-methyltransferase SETMAR OS=Homo sapiens GN=SETMAR PE=1 SV=1 - [SETMR_HUMAN] | 0.960 |
| Q5T6J7 | *IDNK* | Probable gluconokinase OS=Homo sapiens GN=C9orf103 PE=2 SV=1 - [GNTK_HUMAN] | 0.914 |
| Q9UNK0 | *STX8* | Syntaxin-8 OS=Homo sapiens GN=STX8 PE=1 SV=2 - [STX8_HUMAN] | 1.056 |
| P52272 | *HNRNPM* | Isoform 2 of Heterogeneous nuclear ribonucleoprotein M OS=Homo sapiens GN=HNRNPM - [HNRPM_HUMAN] | 1.026 |
| Q15435 | *PPP1R7* | Protein phosphatase 1 regulatory subunit 7 OS=Homo sapiens GN=PPP1R7 PE=1 SV=1 - [PP1R7_HUMAN] | 1.030 |
| P50539 | *MXI1* | Isoform 2 of Max-interacting protein 1 OS=Homo sapiens GN=MXI1 - [MXI1_HUMAN] | 0.924 |
| Q8NHH9 | *ATL2* | Isoform 3 of Atlastin-2 OS=Homo sapiens GN=ATL2 - [ATLA2_HUMAN] | 0.953 |
| P52298 | *NCBP2* | Nuclear cap-binding protein subunit 2 OS=Homo sapiens GN=NCBP2 PE=1 SV=1 - [NCBP2_HUMAN] | 1.037 |
| P61326 | *MAGOH* | Protein mago nashi homolog OS=Homo sapiens GN=MAGOH PE=1 SV=1 - [MGN_HUMAN] | 0.969 |
| Q9Y5U2 | *TSSC4* | Isoform 2 of Protein TSSC4 OS=Homo sapiens GN=TSSC4 - [TSSC4_HUMAN] | 1.056 |
| Q9BY11 | *PACSIN1* | Protein kinase C and casein kinase substrate in neurons protein 1 OS=Homo sapiens GN=PACSIN1 PE=1 SV=1 - [PACN1_HUMAN] | 1.301 |
| O60885 | *BRD4* | Bromodomain-containing protein 4 OS=Homo sapiens GN=BRD4 PE=1 SV=2 - [BRD4_HUMAN] | 1.039 |
| Q9Y6W5 | *WASF2* | Wiskott-Aldrich syndrome protein family member 2 OS=Homo sapiens GN=WASF2 PE=1 SV=3 - [WASF2_HUMAN] | 0.959 |
| Q9Y2Z0 | *SUGT1* | Isoform 2 of Suppressor of G2 allele of SKP1 homolog OS=Homo sapiens GN=SUGT1 - [SUGT1_HUMAN] | 1.076 |
| Q9P253 | *VPS18* | Vacuolar protein sorting-associated protein 18 homolog OS=Homo sapiens GN=VPS18 PE=1 SV=2 - [VPS18_HUMAN] | 0.954 |
| P82930 | *MRPS34* | 28S ribosomal protein S34, mitochondrial OS=Homo sapiens GN=MRPS34 PE=1 SV=2 - [RT34_HUMAN] | 1.030 |
| Q9BV36 | *MLPH* | Isoform 2 of Melanophilin OS=Homo sapiens GN=MLPH - [MELPH_HUMAN] | 0.822 |
| Q9Y3I0 | *RTCB* | tRNA-splicing ligase RtcB homolog OS=Homo sapiens GN=C22orf28 PE=1 SV=1 - [RTCB_HUMAN] | 0.968 |
| Q9Y6W3 | *CAPN7* | Calpain-7 OS=Homo sapiens GN=CAPN7 PE=1 SV=1 - [CAN7_HUMAN] | 0.940 |
| Q5K4L6 | *SLC27A3* | Isoform 3 of Long-chain fatty acid transport protein 3 OS=Homo sapiens GN=SLC27A3 - [S27A3_HUMAN] | 0.845 |
| Q6P9B9 | *INTS5* | Integrator complex subunit 5 OS=Homo sapiens GN=INTS5 PE=1 SV=1 - [INT5_HUMAN] | 1.058 |
| O00299 | *CLIC1* | Chloride intracellular channel protein 1 OS=Homo sapiens GN=CLIC1 PE=1 SV=4 - [CLIC1_HUMAN] | 1.072 |
| Q9H3Z4 | *DNAJC5* | DnaJ homolog subfamily C member 5 OS=Homo sapiens GN=DNAJC5 PE=1 SV=1 - [DNJC5_HUMAN] | 0.938 |
| P06396 | *GSN* | Isoform 2 of Gelsolin OS=Homo sapiens GN=GSN - [GELS_HUMAN] | 0.891 |
| Q9UK45 | *LSM7* | U6 snRNA-associated Sm-like protein LSm7 OS=Homo sapiens GN=LSM7 PE=1 SV=1 - [LSM7_HUMAN] | 1.068 |
| O76038 | *SCGN* | Secretagogin OS=Homo sapiens GN=SCGN PE=1 SV=2 - [SEGN_HUMAN] | 0.915 |
| Q8NF91 | *SYNE1* | Isoform 4 of Nesprin-1 OS=Homo sapiens GN=SYNE1 - [SYNE1_HUMAN] | 0.955 |
| Q14258 | *TRIM25* | E3 ubiquitin/ISG15 ligase TRIM25 OS=Homo sapiens GN=TRIM25 PE=1 SV=2 - [TRI25_HUMAN] | 1.062 |
| Q9H6U6 | *BCAS3* | Isoform 1 of Breast carcinoma-amplified sequence 3 OS=Homo sapiens GN=BCAS3 - [BCAS3_HUMAN] | 0.956 |
| Q9NX63 | *CHCHD3* | Coiled-coil-helix-coiled-coil-helix domain-containing protein 3, mitochondrial OS=Homo sapiens GN=CHCHD3 PE=1 SV=1 - [CHCH3_HUMAN] | 0.947 |
| Q8IYJ3 | *SYTL1* | Synaptotagmin-like protein 1 OS=Homo sapiens GN=SYTL1 PE=1 SV=1 - [SYTL1_HUMAN] | 1.071 |
| P78368 | *CSNK1G2* | Casein kinase I isoform gamma-2 OS=Homo sapiens GN=CSNK1G2 PE=1 SV=1 - [KC1G2_HUMAN] | 0.971 |
| Q9H6F2 | *TMEM38A* | Trimeric intracellular cation channel type A OS=Homo sapiens GN=TMEM38A PE=1 SV=1 - [TM38A_HUMAN] | 1.174 |
| P30443 | *HLA-A* | HLA class I histocompatibility antigen, A-1 alpha chain OS=Homo sapiens GN=HLA-A PE=1 SV=1 - [1A01_HUMAN] | 0.669 |
| Q8IWB7 | *WDFY1* | WD repeat and FYVE domain-containing protein 1 OS=Homo sapiens GN=WDFY1 PE=1 SV=1 - [WDFY1_HUMAN] | 0.977 |
| O75083 | *WDR1* | Isoform 2 of WD repeat-containing protein 1 OS=Homo sapiens GN=WDR1 - [WDR1_HUMAN] | 1.054 |
| Q9BQ61 | *C19orf43* | Uncharacterized protein C19orf43 OS=Homo sapiens GN=C19orf43 PE=1 SV=1 - [CS043_HUMAN] | 1.051 |
| Q12965 | *MYO1E* | Myosin-Ie OS=Homo sapiens GN=MYO1E PE=1 SV=2 - [MYO1E_HUMAN] | 0.933 |
| Q14993 | *COL19A1* | Collagen alpha-1(XIX) chain OS=Homo sapiens GN=COL19A1 PE=1 SV=3 - [COJA1_HUMAN] | 1.401 |
| Q9NR12 | *PDLIM7* | Isoform 2 of PDZ and LIM domain protein 7 OS=Homo sapiens GN=PDLIM7 - [PDLI7_HUMAN] | 1.095 |
| Q8TCX1 | *DYNC2LI1* | Isoform 5 of Cytoplasmic dynein 2 light intermediate chain 1 OS=Homo sapiens GN=DYNC2LI1 - [DC2L1_HUMAN] | 0.945 |
| Q6UXH1 | *CRELD2* | Isoform 4 of Cysteine-rich with EGF-like domain protein 2 OS=Homo sapiens GN=CRELD2 - [CREL2_HUMAN] | 1.113 |
| P56556 | *NDUFA6* | NADH dehydrogenase [ubiquinone] 1 alpha subcomplex subunit 6 OS=Homo sapiens GN=NDUFA6 PE=1 SV=3 - [NDUA6_HUMAN] | 0.962 |
| P05090 | *APOD* | Apolipoprotein D OS=Homo sapiens GN=APOD PE=1 SV=1 - [APOD_HUMAN] | 1.390 |
| P62899 | *RPL31* | 60S ribosomal protein L31 OS=Homo sapiens GN=RPL31 PE=1 SV=1 - [RL31_HUMAN] | 1.027 |
| P33240 | *CSTF2* | Isoform 2 of Cleavage stimulation factor subunit 2 OS=Homo sapiens GN=CSTF2 - [CSTF2_HUMAN] | 1.031 |
| Q96EL2 | *MRPS24* | 28S ribosomal protein S24, mitochondrial OS=Homo sapiens GN=MRPS24 PE=1 SV=1 - [RT24_HUMAN] | 0.965 |
| P36405 | *ARL3* | ADP-ribosylation factor-like protein 3 OS=Homo sapiens GN=ARL3 PE=1 SV=2 - [ARL3_HUMAN] | 0.973 |
| Q15287 | *RNPS1* | Isoform 3 of RNA-binding protein with serine-rich domain 1 OS=Homo sapiens GN=RNPS1 - [RNPS1_HUMAN] | 1.030 |
| P00441 | *SOD1* | Superoxide dismutase [Cu-Zn] OS=Homo sapiens GN=SOD1 PE=1 SV=2 - [SODC_HUMAN] | 0.965 |
| P06748 | *NPM1* | Isoform 2 of Nucleophosmin OS=Homo sapiens GN=NPM1 - [NPM_HUMAN] | 1.046 |
| Q99570 | *PIK3R4* | Phosphoinositide 3-kinase regulatory subunit 4 OS=Homo sapiens GN=PIK3R4 PE=1 SV=3 - [PI3R4_HUMAN] | 0.957 |
| P46459 | *NSF* | Vesicle-fusing ATPase OS=Homo sapiens GN=NSF PE=1 SV=3 - [NSF_HUMAN] | 0.959 |
| Q8NFV4 | *ABHD11* | Isoform 4 of Abhydrolase domain-containing protein 11 OS=Homo sapiens GN=ABHD11 - [ABHDB_HUMAN] | 0.940 |
| Q15059 | *BRD3* | Isoform 2 of Bromodomain-containing protein 3 OS=Homo sapiens GN=BRD3 - [BRD3_HUMAN] | 0.959 |
| Q99720 | *SIGMAR1* | Isoform 4 of Sigma non-opioid intracellular receptor 1 OS=Homo sapiens GN=SIGMAR1 - [SGMR1_HUMAN] | 1.085 |
| Q04656 | *ATP7A* | Isoform 5 of Copper-transporting ATPase 1 OS=Homo sapiens GN=ATP7A - [ATP7A_HUMAN] | 0.934 |
| P05106 | *ITGB3* | Isoform Beta-3B of Integrin beta-3 OS=Homo sapiens GN=ITGB3 - [ITB3_HUMAN] | 0.895 |
| O75477 | *ERLIN1* | Erlin-1 OS=Homo sapiens GN=ERLIN1 PE=1 SV=1 - [ERLN1_HUMAN] | 1.054 |
| Q9UBU8 | *MORF4L1* | Isoform 2 of Mortality factor 4-like protein 1 OS=Homo sapiens GN=MORF4L1 - [MO4L1_HUMAN] | 1.050 |
| Q08AD1 | *CAMSAP2* | Isoform 2 of Calmodulin-regulated spectrin-associated protein 2 OS=Homo sapiens GN=CAMSAP2 - [CAMP2_HUMAN] | 1.060 |
| P15374 | *UCHL3* | Ubiquitin carboxyl-terminal hydrolase isozyme L3 OS=Homo sapiens GN=UCHL3 PE=1 SV=1 - [UCHL3_HUMAN] | 0.950 |
| Q8WVK2 | *SNRNP27* | U4/U6.U5 small nuclear ribonucleoprotein 27 kDa protein OS=Homo sapiens GN=SNRNP27 PE=1 SV=1 - [SNR27_HUMAN] | 0.951 |
| Q96EQ0 | *SGTB* | Small glutamine-rich tetratricopeptide repeat-containing protein beta OS=Homo sapiens GN=SGTB PE=1 SV=1 - [SGTB_HUMAN] | 1.076 |
| Q6ZSR9 | *#N/A* | Uncharacterized protein FLJ45252 OS=Homo sapiens PE=1 SV=2 - [YJ005_HUMAN] | 0.961 |
| Q06481 | *APLP2* | Isoform 5 of Amyloid-like protein 2 OS=Homo sapiens GN=APLP2 - [APLP2_HUMAN] | 1.068 |
| Q9BXP5 | *SRRT* | Isoform 5 of Serrate RNA effector molecule homolog OS=Homo sapiens GN=SRRT - [SRRT_HUMAN] | 1.032 |
| O75521 | *ECI2* | Isoform 2 of Enoyl-CoA delta isomerase 2, mitochondrial OS=Homo sapiens GN=ECI2 - [ECI2_HUMAN] | 0.888 |
| O95870 | *ABHD16A* | Abhydrolase domain-containing protein 16A OS=Homo sapiens GN=ABHD16A PE=1 SV=3 - [ABHGA_HUMAN] | 0.946 |
| Q96F10 | *SAT2* | Diamine acetyltransferase 2 OS=Homo sapiens GN=SAT2 PE=1 SV=1 - [SAT2_HUMAN] | 1.109 |
| Q8NBQ5 | *HSD17B11* | Estradiol 17-beta-dehydrogenase 11 OS=Homo sapiens GN=HSD17B11 PE=1 SV=3 - [DHB11_HUMAN] | 0.892 |
| P68871 | *HBB* | Hemoglobin subunit beta OS=Homo sapiens GN=HBB PE=1 SV=2 - [HBB_HUMAN] | 0.846 |
| O43813 | *LANCL1* | LanC-like protein 1 OS=Homo sapiens GN=LANCL1 PE=1 SV=1 - [LANC1_HUMAN] | 0.960 |
| Q8N465 | *D2HGDH* | D-2-hydroxyglutarate dehydrogenase, mitochondrial OS=Homo sapiens GN=D2HGDH PE=1 SV=3 - [D2HDH_HUMAN] | 1.074 |
| O60684 | *KPNA6* | Importin subunit alpha-7 OS=Homo sapiens GN=KPNA6 PE=1 SV=1 - [IMA7_HUMAN] | 1.040 |
| Q9Y2X3 | *NOP58* | Nucleolar protein 58 OS=Homo sapiens GN=NOP58 PE=1 SV=1 - [NOP58_HUMAN] | 1.036 |
| P40926 | *MDH2* | Malate dehydrogenase, mitochondrial OS=Homo sapiens GN=MDH2 PE=1 SV=3 - [MDHM_HUMAN] | 1.050 |
| Q92575 | *UBXN4* | UBX domain-containing protein 4 OS=Homo sapiens GN=UBXN4 PE=1 SV=2 - [UBXN4_HUMAN] | 1.065 |
| P28370 | *SMARCA1* | Isoform 2 of Probable global transcription activator SNF2L1 OS=Homo sapiens GN=SMARCA1 - [SMCA1_HUMAN] | 1.054 |
| Q6IN85 | *SMEK1* | Isoform 5 of Serine/threonine-protein phosphatase 4 regulatory subunit 3A OS=Homo sapiens GN=SMEK1 - [P4R3A_HUMAN] | 1.025 |
| O43818 | *RRP9* | U3 small nucleolar RNA-interacting protein 2 OS=Homo sapiens GN=RRP9 PE=1 SV=1 - [U3IP2_HUMAN] | 1.050 |
| P13639 | *EEF2* | Elongation factor 2 OS=Homo sapiens GN=EEF2 PE=1 SV=4 - [EF2_HUMAN] | 1.033 |
| Q12974 | *PTP4A2* | Isoform 2 of Protein tyrosine phosphatase type IVA 2 OS=Homo sapiens GN=PTP4A2 - [TP4A2_HUMAN] | 0.946 |
| Q9H6X2 | *ANTXR1* | Isoform 3 of Anthrax toxin receptor 1 OS=Homo sapiens GN=ANTXR1 - [ANTR1_HUMAN] | 1.146 |
| Q8N1F7 | *NUP93* | Nuclear pore complex protein Nup93 OS=Homo sapiens GN=NUP93 PE=1 SV=2 - [NUP93_HUMAN] | 1.034 |
| Q16270 | *IGFBP7* | Insulin-like growth factor-binding protein 7 OS=Homo sapiens GN=IGFBP7 PE=1 SV=1 - [IBP7_HUMAN] | 0.867 |
| P62937 | *PPIA* | Peptidyl-prolyl cis-trans isomerase A OS=Homo sapiens GN=PPIA PE=1 SV=2 - [PPIA_HUMAN] | 1.027 |
| Q9H0V9 | *LMAN2L* | VIP36-like protein OS=Homo sapiens GN=LMAN2L PE=1 SV=1 - [LMA2L_HUMAN] | 0.949 |
| Q13153 | *PAK1* | Serine/threonine-protein kinase PAK 1 OS=Homo sapiens GN=PAK1 PE=1 SV=2 - [PAK1_HUMAN] | 0.947 |
| P30041 | *PRDX6* | Peroxiredoxin-6 OS=Homo sapiens GN=PRDX6 PE=1 SV=3 - [PRDX6_HUMAN] | 1.050 |
| Q17R31 | *TATDN3* | Isoform 2 of Putative deoxyribonuclease TATDN3 OS=Homo sapiens GN=TATDN3 - [TATD3_HUMAN] | 1.130 |
| Q8NBR6 | *FAM63B* | Isoform 2 of Protein FAM63B OS=Homo sapiens GN=FAM63B - [FA63B_HUMAN] | 0.934 |
| O94885 | *SASH1* | SAM and SH3 domain-containing protein 1 OS=Homo sapiens GN=SASH1 PE=1 SV=3 - [SASH1_HUMAN] | 1.080 |
| Q14126 | *DSG2* | Desmoglein-2 OS=Homo sapiens GN=DSG2 PE=1 SV=2 - [DSG2_HUMAN] | 1.168 |
| P51161 | *FABP6* | Gastrotropin OS=Homo sapiens GN=FABP6 PE=1 SV=2 - [FABP6_HUMAN] | 0.938 |
| Q9NYB9 | *ABI2* | Isoform 2 of Abl interactor 2 OS=Homo sapiens GN=ABI2 - [ABI2_HUMAN] | 0.942 |
| Q14204 | *DYNC1H1* | Cytoplasmic dynein 1 heavy chain 1 OS=Homo sapiens GN=DYNC1H1 PE=1 SV=5 - [DYHC1_HUMAN] | 1.038 |
| Q8WXF7 | *ATL1* | Atlastin-1 OS=Homo sapiens GN=ATL1 PE=1 SV=1 - [ATLA1_HUMAN] | 0.921 |
| Q16537 | *PPP2R5E* | Serine/threonine-protein phosphatase 2A 56 kDa regulatory subunit epsilon isoform OS=Homo sapiens GN=PPP2R5E PE=1 SV=1 - [2A5E_HUMAN] | 1.029 |
| P26358 | *DNMT1* | Isoform 3 of DNA (cytosine-5)-methyltransferase 1 OS=Homo sapiens GN=DNMT1 - [DNMT1_HUMAN] | 0.977 |
| O14979 | *HNRNPDL* | Isoform 3 of Heterogeneous nuclear ribonucleoprotein D-like OS=Homo sapiens GN=HNRPDL - [HNRDL_HUMAN] | 1.036 |
| Q8N129 | *CNPY4* | Protein canopy homolog 4 OS=Homo sapiens GN=CNPY4 PE=2 SV=1 - [CNPY4_HUMAN] | 1.102 |
| Q5T160 | *RARS2* | Probable arginine--tRNA ligase, mitochondrial OS=Homo sapiens GN=RARS2 PE=1 SV=1 - [SYRM_HUMAN] | 0.941 |
| P51532 | *SMARCA4* | Transcription activator BRG1 OS=Homo sapiens GN=SMARCA4 PE=1 SV=2 - [SMCA4_HUMAN] | 1.079 |
| Q00341 | *HDLBP* | Vigilin OS=Homo sapiens GN=HDLBP PE=1 SV=2 - [VIGLN_HUMAN] | 1.024 |
| Q15637 | *SF1* | Isoform 6 of Splicing factor 1 OS=Homo sapiens GN=SF1 - [SF01_HUMAN] | 1.024 |
| P07305 | *H1F0* | Isoform 2 of Histone H1.0 OS=Homo sapiens GN=H1F0 - [H10_HUMAN] | 1.046 |
| O60264 | *SMARCA5* | SWI/SNF-related matrix-associated actin-dependent regulator of chromatin subfamily A member 5 OS=Homo sapiens GN=SMARCA5 PE=1 SV=1 - [SMCA5_HUMAN] | 1.044 |
| Q6P9A2 | *GALNT18* | Isoform 2 of Putative polypeptide N-acetylgalactosaminyltransferase-like protein 4 OS=Homo sapiens GN=GALNTL4 - [GLTL4_HUMAN] | 0.855 |
| Q96AC1 | *FERMT2* | Fermitin family homolog 2 OS=Homo sapiens GN=FERMT2 PE=1 SV=1 - [FERM2_HUMAN] | 0.926 |
| Q99707 | *MTR* | Methionine synthase OS=Homo sapiens GN=MTR PE=1 SV=2 - [METH_HUMAN] | 0.925 |
| Q9BV36 | *MLPH* | Isoform 3 of Melanophilin OS=Homo sapiens GN=MLPH - [MELPH_HUMAN] | 0.836 |
| P23786 | *CPT2* | Carnitine O-palmitoyltransferase 2, mitochondrial OS=Homo sapiens GN=CPT2 PE=1 SV=2 - [CPT2_HUMAN] | 0.932 |
| Q9Y608 | *LRRFIP2* | Isoform 2 of Leucine-rich repeat flightless-interacting protein 2 OS=Homo sapiens GN=LRRFIP2 - [LRRF2_HUMAN] | 0.957 |
| Q8IWT6 | *LRRC8A* | Leucine-rich repeat-containing protein 8A OS=Homo sapiens GN=LRRC8A PE=1 SV=1 - [LRC8A_HUMAN] | 0.901 |
| Q9BZ95 | *WHSC1L1* | Isoform 3 of Histone-lysine N-methyltransferase NSD3 OS=Homo sapiens GN=WHSC1L1 - [NSD3_HUMAN] | 1.039 |
| Q8TCS8 | *PNPT1* | Polyribonucleotide nucleotidyltransferase 1, mitochondrial OS=Homo sapiens GN=PNPT1 PE=1 SV=2 - [PNPT1_HUMAN] | 0.967 |
| Q8WUD1 | *RAB2B* | Ras-related protein Rab-2B OS=Homo sapiens GN=RAB2B PE=1 SV=1 - [RAB2B_HUMAN] | 1.041 |
| P15559 | *NQO1* | NAD(P)H dehydrogenase [quinone] 1 OS=Homo sapiens GN=NQO1 PE=1 SV=1 - [NQO1_HUMAN] | 0.857 |
| P84085 | *ARF5* | ADP-ribosylation factor 5 OS=Homo sapiens GN=ARF5 PE=1 SV=2 - [ARF5_HUMAN] | 1.051 |
| P23368 | *ME2* | NAD-dependent malic enzyme, mitochondrial OS=Homo sapiens GN=ME2 PE=1 SV=1 - [MAOM_HUMAN] | 1.045 |
| Q9Y6K9 | *IKBKG* | Isoform 3 of NF-kappa-B essential modulator OS=Homo sapiens GN=IKBKG - [NEMO_HUMAN] | 1.028 |
| O14972 | *DSCR3* | Down syndrome critical region protein 3 OS=Homo sapiens GN=DSCR3 PE=1 SV=1 - [DSCR3_HUMAN] | 0.958 |
| P50395 | *GDI2* | Rab GDP dissociation inhibitor beta OS=Homo sapiens GN=GDI2 PE=1 SV=2 - [GDIB_HUMAN] | 1.055 |
| Q13011 | *ECH1* | Delta(3,5)-Delta(2,4)-dienoyl-CoA isomerase, mitochondrial OS=Homo sapiens GN=ECH1 PE=1 SV=2 - [ECH1_HUMAN] | 0.965 |
| O75489 | *NDUFS3* | NADH dehydrogenase [ubiquinone] iron-sulfur protein 3, mitochondrial OS=Homo sapiens GN=NDUFS3 PE=1 SV=1 - [NDUS3_HUMAN] | 0.971 |
| Q8TB36 | *GDAP1* | Ganglioside-induced differentiation-associated protein 1 OS=Homo sapiens GN=GDAP1 PE=1 SV=3 - [GDAP1_HUMAN] | 0.935 |
| Q7Z3U7 | *MON2* | Isoform 2 of Protein MON2 homolog OS=Homo sapiens GN=MON2 - [MON2_HUMAN] | 0.971 |
| Q9BVK6 | *TMED9* | Transmembrane emp24 domain-containing protein 9 OS=Homo sapiens GN=TMED9 PE=1 SV=2 - [TMED9_HUMAN] | 1.070 |
| Q02818 | *NUCB1* | Nucleobindin-1 OS=Homo sapiens GN=NUCB1 PE=1 SV=4 - [NUCB1_HUMAN] | 1.083 |
| Q15366 | *PCBP2* | Poly(rC)-binding protein 2 OS=Homo sapiens GN=PCBP2 PE=1 SV=1 - [PCBP2_HUMAN] | 1.023 |
| Q96HW7 | *INTS4* | Integrator complex subunit 4 OS=Homo sapiens GN=INTS4 PE=1 SV=2 - [INT4_HUMAN] | 1.028 |
| P32119 | *PRDX2* | Peroxiredoxin-2 OS=Homo sapiens GN=PRDX2 PE=1 SV=5 - [PRDX2_HUMAN] | 1.076 |
| Q92614 | *MYO18A* | Isoform 5 of Myosin-XVIIIa OS=Homo sapiens GN=MYO18A - [MY18A_HUMAN] | 1.080 |
| Q9NVH1 | *DNAJC11* | Isoform 3 of DnaJ homolog subfamily C member 11 OS=Homo sapiens GN=DNAJC11 - [DJC11_HUMAN] | 1.039 |
| P50747 | *HLCS* | Biotin--protein ligase OS=Homo sapiens GN=HLCS PE=1 SV=1 - [BPL1_HUMAN] | 0.968 |
| Q96JJ7 | *TMX3* | Protein disulfide-isomerase TMX3 OS=Homo sapiens GN=TMX3 PE=1 SV=2 - [TMX3_HUMAN] | 1.065 |
| Q9UH62 | *ARMCX3* | Armadillo repeat-containing X-linked protein 3 OS=Homo sapiens GN=ARMCX3 PE=1 SV=1 - [ARMX3_HUMAN] | 1.083 |
| P40763 | *STAT3* | Signal transducer and activator of transcription 3 OS=Homo sapiens GN=STAT3 PE=1 SV=2 - [STAT3_HUMAN] | 1.035 |
| Q14289 | *PTK2B* | Isoform 2 of Protein-tyrosine kinase 2-beta OS=Homo sapiens GN=PTK2B - [FAK2_HUMAN] | 0.929 |
| Q8NHS0 | *DNAJB8* | DnaJ homolog subfamily B member 8 OS=Homo sapiens GN=DNAJB8 PE=1 SV=1 - [DNJB8_HUMAN] | 0.980 |
| Q6ZNB6 | *NFXL1* | NF-X1-type zinc finger protein NFXL1 OS=Homo sapiens GN=NFXL1 PE=1 SV=2 - [NFXL1_HUMAN] | 0.938 |
| O43776 | *NARS* | Asparagine--tRNA ligase, cytoplasmic OS=Homo sapiens GN=NARS PE=1 SV=1 - [SYNC_HUMAN] | 1.051 |
| Q14746 | *COG2* | Conserved oligomeric Golgi complex subunit 2 OS=Homo sapiens GN=COG2 PE=1 SV=1 - [COG2_HUMAN] | 1.044 |
| P15144 | *ANPEP* | Aminopeptidase N OS=Homo sapiens GN=ANPEP PE=1 SV=4 - [AMPN_HUMAN] | 0.852 |
| Q96LJ7 | *DHRS1* | Dehydrogenase/reductase SDR family member 1 OS=Homo sapiens GN=DHRS1 PE=1 SV=1 - [DHRS1_HUMAN] | 1.065 |
| Q16513 | *PKN2* | Isoform 2 of Serine/threonine-protein kinase N2 OS=Homo sapiens GN=PKN2 - [PKN2_HUMAN] | 1.064 |
| Q86UW7 | *CADPS2* | Isoform 3 of Calcium-dependent secretion activator 2 OS=Homo sapiens GN=CADPS2 - [CAPS2_HUMAN] | 1.095 |
| Q9UNM6 | *PSMD13* | 26S proteasome non-ATPase regulatory subunit 13 OS=Homo sapiens GN=PSMD13 PE=1 SV=2 - [PSD13_HUMAN] | 1.034 |
| P51151 | *RAB9A* | Ras-related protein Rab-9A OS=Homo sapiens GN=RAB9A PE=1 SV=1 - [RAB9A_HUMAN] | 0.943 |
| Q9Y2W2 | *WBP11* | WW domain-binding protein 11 OS=Homo sapiens GN=WBP11 PE=1 SV=1 - [WBP11_HUMAN] | 0.972 |
| Q2TAL8 | *QRICH1* | Glutamine-rich protein 1 OS=Homo sapiens GN=QRICH1 PE=1 SV=1 - [QRIC1_HUMAN] | 1.074 |
| P15735 | *PHKG2* | Isoform 2 of Phosphorylase b kinase gamma catalytic chain, testis/liver isoform OS=Homo sapiens GN=PHKG2 - [PHKG2_HUMAN] | 1.061 |
| Q5SWX8 | *C1orf27* | Isoform 3 of Protein odr-4 homolog OS=Homo sapiens GN=ODR4 - [ODR4_HUMAN] | 0.963 |
| Q02108 | *GUCY1A3* | Guanylate cyclase soluble subunit alpha-3 OS=Homo sapiens GN=GUCY1A3 PE=1 SV=2 - [GCYA3_HUMAN] | 1.121 |
| Q9BRA2 | *TXNDC17* | Thioredoxin domain-containing protein 17 OS=Homo sapiens GN=TXNDC17 PE=1 SV=1 - [TXD17_HUMAN] | 1.044 |
| Q7Z6B0 | *CCDC91* | Isoform 2 of Coiled-coil domain-containing protein 91 OS=Homo sapiens GN=CCDC91 - [CCD91_HUMAN] | 0.947 |
| P09234 | *SNRPC* | U1 small nuclear ribonucleoprotein C OS=Homo sapiens GN=SNRPC PE=1 SV=1 - [RU1C_HUMAN] | 0.902 |
| O00505 | *KPNA3* | Importin subunit alpha-3 OS=Homo sapiens GN=KPNA3 PE=1 SV=2 - [IMA3_HUMAN] | 0.974 |
| P05154 | *SERPINA5* | Plasma serine protease inhibitor OS=Homo sapiens GN=SERPINA5 PE=1 SV=3 - [IPSP_HUMAN] | 0.892 |
| P69905 | *HBA2* | Hemoglobin subunit alpha OS=Homo sapiens GN=HBA1 PE=1 SV=2 - [HBA_HUMAN] | 0.843 |
| O14497 | *ARID1A* | Isoform 3 of AT-rich interactive domain-containing protein 1A OS=Homo sapiens GN=ARID1A - [ARI1A_HUMAN] | 1.055 |
| Q9NQC7 | *CYLD* | Isoform 2 of Ubiquitin carboxyl-terminal hydrolase CYLD OS=Homo sapiens GN=CYLD - [CYLD_HUMAN] | 1.094 |
| O00182 | *LGALS9* | Isoform Short of Galectin-9 OS=Homo sapiens GN=LGALS9 - [LEG9_HUMAN] | 0.902 |
| Q13838 | *DDX39B* | Spliceosome RNA helicase DDX39B OS=Homo sapiens GN=DDX39B PE=1 SV=1 - [DX39B_HUMAN] | 1.028 |
| P48729 | *CSNK1A1* | Casein kinase I isoform alpha OS=Homo sapiens GN=CSNK1A1 PE=1 SV=2 - [KC1A_HUMAN] | 1.033 |
| P31321 | *PRKAR1B* | cAMP-dependent protein kinase type I-beta regulatory subunit OS=Homo sapiens GN=PRKAR1B PE=1 SV=4 - [KAP1_HUMAN] | 0.955 |
| O95786 | *DDX58* | Isoform 2 of Probable ATP-dependent RNA helicase DDX58 OS=Homo sapiens GN=DDX58 - [DDX58_HUMAN] | 1.119 |
| Q7RTV0 | *PHF5A* | PHD finger-like domain-containing protein 5A OS=Homo sapiens GN=PHF5A PE=1 SV=1 - [PHF5A_HUMAN] | 0.971 |
| P42025 | *ACTR1B* | Beta-centractin OS=Homo sapiens GN=ACTR1B PE=1 SV=1 - [ACTY_HUMAN] | 1.073 |
| Q9NXE4 | *SMPD4* | Isoform 3 of Sphingomyelin phosphodiesterase 4 OS=Homo sapiens GN=SMPD4 - [NSMA3_HUMAN] | 1.050 |
| P62750 | *RPL23A* | 60S ribosomal protein L23a OS=Homo sapiens GN=RPL23A PE=1 SV=1 - [RL23A_HUMAN] | 1.027 |
| Q13017 | *ARHGAP5* | Isoform 2 of Rho GTPase-activating protein 5 OS=Homo sapiens GN=ARHGAP5 - [RHG05_HUMAN] | 0.947 |
| Q2TV78 | *#N/A* | Isoform 2 of Putative macrophage-stimulating protein MSTP9 OS=Homo sapiens GN=MST1P9 - [MSTP9_HUMAN] | 1.119 |
| O94760 | *DDAH1* | N(G),N(G)-dimethylarginine dimethylaminohydrolase 1 OS=Homo sapiens GN=DDAH1 PE=1 SV=3 - [DDAH1_HUMAN] | 0.937 |
| Q8WVV9 | *HNRNPLL* | Isoform 4 of Heterogeneous nuclear ribonucleoprotein L-like OS=Homo sapiens GN=HNRPLL - [HNRLL_HUMAN] | 0.969 |
| Q15459 | *SF3A1* | Splicing factor 3A subunit 1 OS=Homo sapiens GN=SF3A1 PE=1 SV=1 - [SF3A1_HUMAN] | 1.025 |
| Q8WZA0 | *LZIC* | Protein LZIC OS=Homo sapiens GN=LZIC PE=1 SV=1 - [LZIC_HUMAN] | 1.044 |
| P58005 | *SESN3* | Isoform 2 of Sestrin-3 OS=Homo sapiens GN=SESN3 - [SESN3_HUMAN] | 0.933 |
| P98179 | *RBM3* | Putative RNA-binding protein 3 OS=Homo sapiens GN=RBM3 PE=1 SV=1 - [RBM3_HUMAN] | 1.068 |
| Q9Y3U8 | *RPL36* | 60S ribosomal protein L36 OS=Homo sapiens GN=RPL36 PE=1 SV=3 - [RL36_HUMAN] | 1.094 |
| O95168 | *NDUFB4* | NADH dehydrogenase [ubiquinone] 1 beta subcomplex subunit 4 OS=Homo sapiens GN=NDUFB4 PE=1 SV=3 - [NDUB4_HUMAN] | 0.968 |
| O43677 | *NDUFC1* | NADH dehydrogenase [ubiquinone] 1 subunit C1, mitochondrial OS=Homo sapiens GN=NDUFC1 PE=2 SV=1 - [NDUC1_HUMAN] | 0.932 |
| P30740 | *SERPINB1* | Leukocyte elastase inhibitor OS=Homo sapiens GN=SERPINB1 PE=1 SV=1 - [ILEU_HUMAN] | 0.954 |
| P23246 | *SFPQ* | Splicing factor, proline- and glutamine-rich OS=Homo sapiens GN=SFPQ PE=1 SV=2 - [SFPQ_HUMAN] | 0.957 |
| Q9NQT8 | *KIF13B* | Kinesin-like protein KIF13B OS=Homo sapiens GN=KIF13B PE=1 SV=1 - [KI13B_HUMAN] | 1.061 |
| P09211 | *GSTP1* | Glutathione S-transferase P OS=Homo sapiens GN=GSTP1 PE=1 SV=2 - [GSTP1_HUMAN] | 0.913 |
| P08574 | *CYC1* | Cytochrome c1, heme protein, mitochondrial OS=Homo sapiens GN=CYC1 PE=1 SV=3 - [CY1_HUMAN] | 0.953 |
| Q9BU61 | *NDUFAF3* | NADH dehydrogenase [ubiquinone] 1 alpha subcomplex assembly factor 3 OS=Homo sapiens GN=NDUFAF3 PE=1 SV=1 - [NDUF3_HUMAN] | 0.963 |
| Q16555 | *DPYSL2* | Dihydropyrimidinase-related protein 2 OS=Homo sapiens GN=DPYSL2 PE=1 SV=1 - [DPYL2_HUMAN] | 0.948 |
| Q8NBS9 | *TXNDC5* | Thioredoxin domain-containing protein 5 OS=Homo sapiens GN=TXNDC5 PE=1 SV=2 - [TXND5_HUMAN] | 1.098 |
| Q9ULD0 | *OGDHL* | Isoform 3 of 2-oxoglutarate dehydrogenase-like, mitochondrial OS=Homo sapiens GN=OGDHL - [OGDHL_HUMAN] | 0.902 |
| O15031 | *PLXNB2* | Plexin-B2 OS=Homo sapiens GN=PLXNB2 PE=1 SV=3 - [PLXB2_HUMAN] | 0.965 |
| Q15554 | *TERF2* | Isoform 2 of Telomeric repeat-binding factor 2 OS=Homo sapiens GN=TERF2 - [TERF2_HUMAN] | 1.039 |
| O75351 | *VPS4B* | Vacuolar protein sorting-associated protein 4B OS=Homo sapiens GN=VPS4B PE=1 SV=2 - [VPS4B_HUMAN] | 1.027 |
| Q7Z3J2 | *C16orf62* | Isoform 2 of UPF0505 protein C16orf62 OS=Homo sapiens GN=C16orf62 - [CP062_HUMAN] | 0.937 |
| P62736 | *ACTA2* | Actin, aortic smooth muscle OS=Homo sapiens GN=ACTA2 PE=1 SV=1 - [ACTA_HUMAN] | 0.909 |
| Q08117 | *AES* | Amino-terminal enhancer of split OS=Homo sapiens GN=AES PE=1 SV=4 - [AES_HUMAN] | 0.949 |
| Q9NR99 | *MXRA5* | Matrix-remodeling-associated protein 5 OS=Homo sapiens GN=MXRA5 PE=2 SV=3 - [MXRA5_HUMAN] | 1.125 |
| Q8WZ82 | *OVCA2* | Ovarian cancer-associated gene 2 protein OS=Homo sapiens GN=OVCA2 PE=1 SV=1 - [OVCA2_HUMAN] | 0.956 |
| Q9H300 | *PARL* | Isoform 2 of Presenilins-associated rhomboid-like protein, mitochondrial OS=Homo sapiens GN=PARL - [PARL_HUMAN] | 1.118 |
| Q92530 | *PSMF1* | Proteasome inhibitor PI31 subunit OS=Homo sapiens GN=PSMF1 PE=1 SV=2 - [PSMF1_HUMAN] | 0.958 |
| O14737 | *PDCD5* | Programmed cell death protein 5 OS=Homo sapiens GN=PDCD5 PE=1 SV=3 - [PDCD5_HUMAN] | 1.045 |
| Q8IXQ4 | *GPALPP1* | Isoform 3 of Uncharacterized protein KIAA1704 OS=Homo sapiens GN=KIAA1704 - [K1704_HUMAN] | 1.054 |
| Q9ULF5 | *SLC39A10* | Zinc transporter ZIP10 OS=Homo sapiens GN=SLC39A10 PE=1 SV=2 - [S39AA_HUMAN] | 1.040 |
| Q9NR34 | *MAN1C1* | Mannosyl-oligosaccharide 1,2-alpha-mannosidase IC OS=Homo sapiens GN=MAN1C1 PE=1 SV=1 - [MA1C1_HUMAN] | 1.069 |
| Q9NX62 | *IMPAD1* | Inositol monophosphatase 3 OS=Homo sapiens GN=IMPAD1 PE=1 SV=1 - [IMPA3_HUMAN] | 1.058 |
| P32455 | *GBP1* | Interferon-induced guanylate-binding protein 1 OS=Homo sapiens GN=GBP1 PE=1 SV=2 - [GBP1_HUMAN] | 1.129 |
| Q8N5G0 | *SMIM20* | Isoform 2 of Uncharacterized protein C4orf52 OS=Homo sapiens GN=C4orf52 - [CD052_HUMAN] | 0.941 |
| P61224 | *RAP1B* | Ras-related protein Rap-1b OS=Homo sapiens GN=RAP1B PE=1 SV=1 - [RAP1B_HUMAN] | 1.069 |
| Q13217 | *DNAJC3* | DnaJ homolog subfamily C member 3 OS=Homo sapiens GN=DNAJC3 PE=1 SV=1 - [DNJC3_HUMAN] | 0.922 |
| Q15233 | *NONO* | Non-POU domain-containing octamer-binding protein OS=Homo sapiens GN=NONO PE=1 SV=4 - [NONO_HUMAN] | 1.024 |
| Q86TN4 | *TRPT1* | Isoform 2 of tRNA 2'-phosphotransferase 1 OS=Homo sapiens GN=TRPT1 - [TRPT1_HUMAN] | 1.035 |
| O60610 | *DIAPH1* | Isoform 2 of Protein diaphanous homolog 1 OS=Homo sapiens GN=DIAPH1 - [DIAP1_HUMAN] | 0.963 |
| C4AMC7 | *#N/A* | Putative WAS protein family homolog 3 OS=Homo sapiens GN=WASH3P PE=2 SV=2 - [WASH3_HUMAN] | 1.026 |
| Q9P2B2 | *PTGFRN* | Prostaglandin F2 receptor negative regulator OS=Homo sapiens GN=PTGFRN PE=1 SV=2 - [FPRP_HUMAN] | 0.889 |
| Q14320 | *FAM50A* | Protein FAM50A OS=Homo sapiens GN=FAM50A PE=1 SV=2 - [FA50A_HUMAN] | 1.036 |
| Q5QJE6 | *DNTTIP2* | Deoxynucleotidyltransferase terminal-interacting protein 2 OS=Homo sapiens GN=DNTTIP2 PE=1 SV=2 - [TDIF2_HUMAN] | 0.976 |
| P22059 | *OSBP* | Oxysterol-binding protein 1 OS=Homo sapiens GN=OSBP PE=1 SV=1 - [OSBP1_HUMAN] | 1.037 |
| P78316 | *NOP14* | Isoform 2 of Nucleolar protein 14 OS=Homo sapiens GN=NOP14 - [NOP14_HUMAN] | 0.957 |
| O00422 | *SAP18* | Histone deacetylase complex subunit SAP18 OS=Homo sapiens GN=SAP18 PE=1 SV=1 - [SAP18_HUMAN] | 1.025 |
| Q12907 | *LMAN2* | Vesicular integral-membrane protein VIP36 OS=Homo sapiens GN=LMAN2 PE=1 SV=1 - [LMAN2_HUMAN] | 0.975 |
| P30044 | *PRDX5* | Isoform Cytoplasmic+peroxisomal of Peroxiredoxin-5, mitochondrial OS=Homo sapiens GN=PRDX5 - [PRDX5_HUMAN] | 0.899 |
| O95674 | *CDS2* | Phosphatidate cytidylyltransferase 2 OS=Homo sapiens GN=CDS2 PE=1 SV=1 - [CDS2_HUMAN] | 0.946 |
| Q9Y3E0 | *GOLT1B* | Vesicle transport protein GOT1B OS=Homo sapiens GN=GOLT1B PE=1 SV=1 - [GOT1B_HUMAN] | 1.040 |
| Q99873 | *PRMT1* | Isoform 3 of Protein arginine N-methyltransferase 1 OS=Homo sapiens GN=PRMT1 - [ANM1_HUMAN] | 1.033 |
| O15344 | *MID1* | Isoform 2 of Midline-1 OS=Homo sapiens GN=MID1 - [TRI18_HUMAN] | 1.089 |
| P11021 | *HSPA5* | 78 kDa glucose-regulated protein OS=Homo sapiens GN=HSPA5 PE=1 SV=2 - [GRP78_HUMAN] | 1.077 |
| P02144 | *MB* | Myoglobin OS=Homo sapiens GN=MB PE=1 SV=2 - [MYG_HUMAN] | 0.779 |
| Q86UK7 | *ZNF598* | Isoform 2 of Zinc finger protein 598 OS=Homo sapiens GN=ZNF598 - [ZN598_HUMAN] | 1.065 |
| Q9Y446 | *PKP3* | Plakophilin-3 OS=Homo sapiens GN=PKP3 PE=1 SV=1 - [PKP3_HUMAN] | 0.947 |
| O00194 | *RAB27B* | Ras-related protein Rab-27B OS=Homo sapiens GN=RAB27B PE=1 SV=4 - [RB27B_HUMAN] | 0.811 |
| Q6I9Y2 | *THOC7* | THO complex subunit 7 homolog OS=Homo sapiens GN=THOC7 PE=1 SV=3 - [THOC7_HUMAN] | 1.036 |
| P62487 | *POLR2G* | DNA-directed RNA polymerase II subunit RPB7 OS=Homo sapiens GN=POLR2G PE=1 SV=1 - [RPB7_HUMAN] | 0.970 |
| Q9BSD7 | *NTPCR* | Cancer-related nucleoside-triphosphatase OS=Homo sapiens GN=NTPCR PE=1 SV=1 - [NTPCR_HUMAN] | 1.050 |
| Q06323 | *PSME1* | Proteasome activator complex subunit 1 OS=Homo sapiens GN=PSME1 PE=1 SV=1 - [PSME1_HUMAN] | 1.046 |
| Q9C0B0 | *UNK* | RING finger protein unkempt homolog OS=Homo sapiens GN=UNK PE=1 SV=2 - [UNK_HUMAN] | 0.973 |
| P57105 | *SYNJ2BP* | Synaptojanin-2-binding protein OS=Homo sapiens GN=SYNJ2BP PE=1 SV=2 - [SYJ2B_HUMAN] | 1.050 |
| Q92922 | *SMARCC1* | SWI/SNF complex subunit SMARCC1 OS=Homo sapiens GN=SMARCC1 PE=1 SV=3 - [SMRC1_HUMAN] | 1.050 |
| O43143 | *DHX15* | Putative pre-mRNA-splicing factor ATP-dependent RNA helicase DHX15 OS=Homo sapiens GN=DHX15 PE=1 SV=2 - [DHX15_HUMAN] | 1.030 |
| O00635 | *TRIM38* | Tripartite motif-containing protein 38 OS=Homo sapiens GN=TRIM38 PE=2 SV=1 - [TRI38_HUMAN] | 1.045 |
| Q13596 | *SNX1* | Isoform 1A of Sorting nexin-1 OS=Homo sapiens GN=SNX1 - [SNX1_HUMAN] | 0.969 |
| Q9NQE9 | *HINT3* | Histidine triad nucleotide-binding protein 3 OS=Homo sapiens GN=HINT3 PE=1 SV=1 - [HINT3_HUMAN] | 1.083 |
| Q13459 | *MYO9B* | Isoform Short of Myosin-IXb OS=Homo sapiens GN=MYO9B - [MYO9B_HUMAN] | 1.040 |
| Q9NZR1 | *TMOD2* | Isoform 2 of Tropomodulin-2 OS=Homo sapiens GN=TMOD2 - [TMOD2_HUMAN] | 0.956 |
| Q9NTI5 | *PDS5B* | Isoform 2 of Sister chromatid cohesion protein PDS5 homolog B OS=Homo sapiens GN=PDS5B - [PDS5B_HUMAN] | 0.951 |
| Q9H9S4 | *CAB39L* | Calcium-binding protein 39-like OS=Homo sapiens GN=CAB39L PE=1 SV=3 - [CB39L_HUMAN] | 1.085 |
| Q15545 | *TAF7* | Transcription initiation factor TFIID subunit 7 OS=Homo sapiens GN=TAF7 PE=1 SV=1 - [TAF7_HUMAN] | 0.954 |
| P23511 | *NFYA* | Isoform Short of Nuclear transcription factor Y subunit alpha OS=Homo sapiens GN=NFYA - [NFYA_HUMAN] | 1.038 |
| Q13501 | *SQSTM1* | Isoform 2 of Sequestosome-1 OS=Homo sapiens GN=SQSTM1 - [SQSTM_HUMAN] | 0.910 |
| P31939 | *ATIC* | Bifunctional purine biosynthesis protein PURH OS=Homo sapiens GN=ATIC PE=1 SV=3 - [PUR9_HUMAN] | 1.043 |
| Q9H6S3 | *EPS8L2* | Epidermal growth factor receptor kinase substrate 8-like protein 2 OS=Homo sapiens GN=EPS8L2 PE=1 SV=2 - [ES8L2_HUMAN] | 1.145 |
| Q8TBX8 | *PIP4K2C* | Phosphatidylinositol-5-phosphate 4-kinase type-2 gamma OS=Homo sapiens GN=PIP4K2C PE=1 SV=3 - [PI42C_HUMAN] | 1.042 |
| Q15555 | *MAPRE2* | Microtubule-associated protein RP/EB family member 2 OS=Homo sapiens GN=MAPRE2 PE=1 SV=1 - [MARE2_HUMAN] | 1.026 |
| P62280 | *RPS11* | 40S ribosomal protein S11 OS=Homo sapiens GN=RPS11 PE=1 SV=3 - [RS11_HUMAN] | 0.965 |
| Q9BY67 | *CADM1* | Cell adhesion molecule 1 OS=Homo sapiens GN=CADM1 PE=1 SV=2 - [CADM1_HUMAN] | 0.929 |
[truncated: 106,278 more chars]
