# Supplementary material for: Genetic and epigenetic background and protein expression profiles in relation to telomerase activation in medullary thyroid carcinoma
Source: Oncotarget. 2016 Feb 8;7(16):21332–46. doi: 10.18632/oncotarget.7237 (PMC5008288; doi:10.18632/oncotarget.7237)
Supplement: Supplementary file 5 [file oncotarget-07-21332-s005.doc]

| **Supplementary Table S4. Differentially expressed proteins by HiRIEF-LC-MS/MS between telomerase positive (n = 8) and negative (n = 6) MTCs.** | | | | |
| --- | --- | --- | --- | --- |
| **Accession** |  |  | ***P-*** | **Fold Positive** |
| **no.** | **Symbol** | **Name** | **value** | **vs. Negative** |
| ***Over-expressed in telomerase activation positive vs. negative MTCs*** | | |  |  |
| P51692 | *RAB3B* | Ras-related protein Rab-3B | 0.014 | 4.04 |
| P55268 | *IGFBP2* | Insulin-like growth factor-binding protein 2 | 0.049 | 3.55 |
| P13987 | *SGPL1* | Sphingosine-1-phosphate lyase 1 | 0.005 | 2.60 |
| P48163 | *IDH2* | Isocitrate dehydrogenase [NADP], mitochondrial | 0.036 | 2.08 |
| P50579 | *PPP1R14B* | Protein phosphatase 1 regulatory subunit 14B | 0.037 | 2.03 |
| Q04837 | *GMDS* | GDP-mannose 4,6 dehydratase | 0.047 | 2.00 |
| Q9Y2S2 | *TMSL3* | Thymosin beta-4-like protein 3 | 0.026 | 1.95 |
| O95622 | *CLPTM1L* | Isoform 2 of Cleft lip and palate transmembrane protein 1-like protein | 0.005 | 1.90 |
| Q56VL3 | *PYCR1* | Pyrroline-5-carboxylate reductase 1, mitochondrial | 0.040 | 1.83 |
| P49908 | *CPD* | Carboxypeptidase D | 0.007 | 1.81 |
| P51178 | *APP* | Isoform L-APP733 of Amyloid beta A4 protein | 0.021 | 1.77 |
| Q9NRW1 | *SPARC* | SPARC | 0.028 | 1.74 |
| P53396 | *PLCB4* | Isoform 3 of 1-phosphatidylinositol-4,5-bisphosphate phosphodiesterase beta-4 | 0.026 | 1.73 |
| Q9NUJ1 | *TM7SF2* | Isoform 2 of Delta(14)-sterol reductase | 0.048 | 1.72 |
| Q96RL7 | *BRI3BP* | BRI3-binding protein | 0.035 | 1.67 |
| P00966 | *SMC6* | Structural maintenance of chromosomes protein 6 | 0.030 | 1.64 |
| P08571 | *GALNT10* | Isoform 3 of Polypeptide N-acetylgalactosaminyltransferase 10 | 0.035 | 1.64 |
| Q9H3G5 | *CIAPIN1* | Isoform 3 of Anamorsin | 0.046 | 1.64 |
| O00629 | *GDPD1* | Isoform 3 of Glycerophosphodiester phosphodiesterase domain-containing protein 1 | 0.003 | 1.63 |
| P46527 | *TMSB10* | Thymosin beta-10 | 0.006 | 1.60 |
| O15230 | *P3H1* | Prolyl 3-hydroxylase 1 | 0.020 | 1.60 |
| Q8N9F7 | *OCIAD2* | Isoform 2 of OCIA domain-containing protein 2 | 0.001 | 1.58 |
| O43264 | *DPP3* | Dipeptidyl peptidase 3 | 0.043 | 1.57 |
| P28289 | *PEX1* | Peroxisome biogenesis factor 1 | 0.029 | 1.56 |
| Q9BX67 | *BRMS1* | Breast cancer metastasis-suppressor 1 | 0.030 | 1.56 |
| P21926 | *TMEM63B* | Transmembrane protein 63B | 0.011 | 1.54 |
| Q9NRL3 | *KHDRBS3* | Isoform 2 of KH domain-containing, RNA-binding, signal transduction-associated protein 3 | 0.039 | 1.51 |
| P49748 | *CRTAP* | Cartilage-associated protein | 0.036 | 1.50 |
| Q6ZMZ3 | *UBE2J1* | Ubiquitin-conjugating enzyme E2 J1 | 0.030 | 1.47 |
| Q9H074 | *HSBP1* | Heat shock factor-binding protein 1 | 0.020 | 1.47 |
| P42574 | *TRIO* | Isoform 5 of Triple functional domain protein | 0.038 | 1.43 |
| Q6IPR1 | *NGDN* | Isoform 2 of Neuroguidin | 0.025 | 1.42 |
| Q96KA5 | *DST* | Isoform 2 of Dystonin | 0.049 | 1.41 |
| O95470 | *COPG2* | Coatomer subunit gamma-2 | 0.028 | 1.41 |
| Q9H223 | *PAIP1* | Isoform 2 of Polyadenylate-binding protein-interacting protein 1 | 0.004 | 1.41 |
| P10909 | *GPSM1* | Isoform 4 of G-protein-signaling modulator 1 | 0.028 | 1.40 |
| P30626 | *CHD9* | Isoform 2 of Chromodomain-helicase-DNA-binding protein 9 | 0.019 | 1.40 |
| P63313 | *MARCKS* | Myristoylated alanine-rich C-kinase substrate | 0.017 | 1.40 |
| Q5VYK3 | *STX18* | Syntaxin-18 | 0.029 | 1.39 |
| O75976 | *RALY* | RNA-binding protein Raly | 0.042 | 1.39 |
| Q9H845 | *CASP3* | Caspase-3 | 0.005 | 1.38 |
| O00186 | *SH3GLB2* | Isoform 2 of Endophilin-B2 | 0.029 | 1.38 |
| P51149 | *MORC2* | Isoform 2 of MORC family CW-type zinc finger protein 2 | 0.021 | 1.38 |
| Q15047 | *UCHL5* | Isoform 2 of Ubiquitin carboxyl-terminal hydrolase isozyme L5 | 0.017 | 1.38 |
| Q9NY15 | *DDX47* | Probable ATP-dependent RNA helicase DDX47 | 0.030 | 1.37 |
| P39019 | *SCFD2* | Isoform 2 of Sec1 family domain-containing protein 2 | 0.028 | 1.37 |
| Q6VY07 | *ZW10* | Centromere/kinetochore protein zw10 homolog | 0.003 | 1.36 |
| Q2NL82 | *APOL2* | Apolipoprotein L2 | 0.015 | 1.35 |
| Q16822 | *CFL1* | Cofilin-1 | 0.028 | 1.35 |
| Q9H3H9 | *ACBD5* | Isoform 4 of Acyl-CoA-binding domain-containing protein 5 | 0.048 | 1.35 |
| P15153 | *HTT* | Huntingtin | 0.045 | 1.35 |
| Q6ZN30 | *LYPLA2* | Acyl-protein thioesterase 2 | 0.042 | 1.34 |
| Q9UBS3 | *PARD3* | Isoform 9 of Partitioning defective 3 homolog | 0.016 | 1.34 |
| Q5T3F8 | *PACS1* | Phosphofurin acidic cluster sorting protein 1 | 0.008 | 1.34 |
| Q9UBW8 | *C19orf25* | UPF0449 protein C19orf25 | 0.016 | 1.33 |
| P22830 | *TIPRL* | TIP41-like protein | 0.046 | 1.33 |
| P41208 | *LMNB1* | Lamin-B1 OS | 0.043 | 1.33 |
| P10768 | *RINT1* | RAD50-interacting protein 1 | 0.039 | 1.32 |
| P23634 | *PPM1G* | Protein phosphatase 1G | 0.029 | 1.32 |
| P30084 | *CLNS1A* | Methylosome subunit pICln | 0.044 | 1.31 |
| P04899 | *ASH2L* | Isoform 2 of Set1/Ash2 histone methyltransferase complex subunit ASH2 | 0.021 | 1.30 |
| P09936 | *NAV1* | Isoform 5 of Neuron navigator 1 | 0.019 | 1.30 |
| Q02083 | *CTTN* | Src substrate cortactin | 0.047 | 1.30 |
| P26440 | *SETD3* | Histone-lysine N-methyltransferase setd3 | 0.035 | 1.30 |
| O00178 | *PPP5C* | Serine/threonine-protein phosphatase 5 | 0.028 | 1.29 |
| Q8NFW8 | *STRN4* | Striatin-4 | 0.004 | 1.29 |
| P20337 | *DUSP23* | Dual specificity protein phosphatase 23 | 0.031 | 1.29 |
| Q9UF11 | *DPM3* | Dolichol-phosphate mannosyltransferase subunit 3 | 0.022 | 1.28 |
| Q96RQ3 | *RNF214* | RING finger protein 214 | 0.032 | 1.28 |
| Q15819 | *DENR* | Density-regulated protein | 0.021 | 1.28 |
| Q9BQE5 | *METAP1* | Methionine aminopeptidase 1 | 0.020 | 1.27 |
| Q8NI22 | *IRF3* | Interferon regulatory factor 3 | 0.034 | 1.27 |
| Q9UFG5 | *RAB25* | Ras-related protein Rab-25 | 0.036 | 1.27 |
| Q8TEW0 | *MPI* | Isoform 2 of Mannose-6-phosphate isomerase | 0.018 | 1.27 |
| Q96F85 | *SETDB1* | Isoform 2 of Histone-lysine N-methyltransferase SETDB1 | 0.007 | 1.27 |
| Q86V97 | *ZC3H18* | Zinc finger CCCH domain-containing protein 18 | 0.020 | 1.27 |
| Q9Y496 | *KIF3A* | Kinesin-like protein KIF3A | 0.016 | 1.26 |
| Q9BVL2 | *GTPBP1* | GTP-binding protein 1 | 0.014 | 1.24 |
| O14974 | *KIAA0368* | Proteasome-associated protein ECM29 homolog | 0.006 | 1.22 |
| Q9Y5K5 | *CD2AP* | CD2-associated protein | 0.039 | 1.21 |
| P29401 | *SZRD1* | Isoform 2 of UPF0485 protein C1orf144 | 0.020 | 1.21 |
| P29966 | *GCC2* | GRIP and coiled-coil domain-containing protein 2 | 0.045 | 1.21 |
| P11047 | *EIF2S2* | Eukaryotic translation initiation factor 2 subunit 2 | 0.022 | 1.21 |
| Q9BUF7 | *FAM96B* | Mitotic spindle-associated MMXD complex subunit MIP18 | 0.033 | 1.21 |
| P34949 | *ATP2A2* | Isoform 2 of Sarcoplasmic/endoplasmic reticulum calcium ATPase 2 | 0.046 | 1.21 |
| P20674 | *NUDCD1* | Isoform 3 of NudC domain-containing protein 1 | 0.024 | 1.20 |
| Q8N475 | *UBE2V2* | Ubiquitin-conjugating enzyme E2 variant 2 | 0.015 | 1.20 |
| Q3L8U1 | *NUPL1* | Isoform 2 of Nucleoporin p58/p45 | 0.017 | 1.19 |
| Q8NEY1 | *TSR1* | Pre-rRNA-processing protein TSR1 homolog | 0.009 | 1.19 |
| Q86VM9 | *GCC1* | GRIP and coiled-coil domain-containing protein 1 | 0.043 | 1.19 |
| O75506 | *AIMP1* | Aminoacyl tRNA synthase complex-interacting multifunctional protein 1 | 0.037 | 1.19 |
| Q7Z422 | *RPS19* | 40S ribosomal protein S19 | 0.007 | 1.18 |
| Q32P28 | *CNOT1* | Isoform 2 of CCR4-NOT transcription complex subunit 1 | 0.030 | 1.17 |
| P53582 | *VCP* | Transitional endoplasmic reticulum ATPase | 0.040 | 1.17 |
| A0AV96 | *GART* | Trifunctional purine biosynthetic protein adenosine-3 | 0.044 | 1.16 |
| Q9Y6X9 | *MED22* | Isoform Surf5A of Mediator of RNA polymerase II transcription subunit 22 | 0.045 | 1.16 |
| Q9UBL3 | *RAB6A* | Ras-related protein Rab-6A | 0.045 | 1.14 |
| P05067 | *STAMBP* | STAM-binding protein | 0.022 | 1.13 |
| O43583 | *PDS5A* | Sister chromatid cohesion protein PDS5 homolog A | 0.050 | 1.13 |
| Q9UL12 | *LARP7* | La-related protein 7 | 0.031 | 1.12 |
| P20042 | *IARS* | Isoleucine--tRNA ligase, cytoplasmic | 0.034 | 1.11 |
| ***Under-expressed in telomerase activation positive vs. negative MTCs*** | | |  |  |
| Q8WWX9 | *SPOCK2* | Testican-2 | 0.044 | 0.18 |
| Q9H147 | *SORD* | Sorbitol dehydrogenase | 0.045 | 0.27 |
| Q29RF7 | *DNAJB9* | DnaJ homolog subfamily B member 9 | 0.011 | 0.27 |
| P26038 | *CLU* | Isoform 4 of Clusterin | 0.006 | 0.28 |
| O60518 | *SEPP1* | Selenoprotein P | 0.001 | 0.30 |
| Q9UBR2 | *ASS1* | Argininosuccinate synthase | 0.002 | 0.32 |
| P18065 | *LAMB2* | Laminin subunit beta-2 | 0.000 | 0.33 |
| Q03001 | *LAMA5* | Laminin subunit alpha-5 | 0.003 | 0.33 |
| Q5T8D3 | *FHL1* | Isoform 3 of Four and a half LIM domains protein 1 | 0.031 | 0.33 |
| O76062 | *CLIC2* | Chloride intracellular channel protein 2 | 0.032 | 0.34 |
| Q6GMV2 | *TCEAL5* | Transcription elongation factor A protein-like 5 | 0.030 | 0.35 |
| Q9UI12 | *TCEAL2* | Transcription elongation factor A protein-like 2 | 0.010 | 0.36 |
| O60547 | *CLU* | Isoform 3 of Clusterin | 0.047 | 0.37 |
| P10909 | *CD59* | CD59 glycoprotein | 0.001 | 0.39 |
| Q14247 | *BNC2* | Isoform 2 of Zinc finger protein basonuclin-2 | 0.011 | 0.39 |
| O14880 | *AASS* | Alpha-aminoadipic semialdehyde synthase, mitochondrial | 0.025 | 0.40 |
| P21397 | *FSTL5* | Follistatin-related protein 5 | 0.019 | 0.42 |
| Q5W0U4 | *CD9* | CD9 antigen | 0.004 | 0.43 |
| O75663 | *TAGLN3* | Transgelin-3 | 0.036 | 0.44 |
| P16615 | *CNRIP1* | Isoform 2 of CB1 cannabinoid receptor-interacting protein 1 | 0.016 | 0.44 |
| O75369 | *ME1* | NADP-dependent malic enzyme | 0.001 | 0.48 |
| Q6FI81 | *LAMC1* | Laminin subunit gamma-1 | 0.017 | 0.49 |
| Q96CN7 | *IGFALS* | Insulin-like growth factor-binding protein complex acid labile subunit | 0.027 | 0.50 |
| P20340 | *NID1* | Isoform 2 of Nidogen-1 | 0.028 | 0.50 |
| P42858 | *STAB1* | Stabilin-1 | 0.007 | 0.50 |
| Q00796 | *B4GAT1* | N-acetyllactosaminide beta-1,3-N-acetylglucosaminyltransferase | 0.044 | 0.51 |
| Q8IWJ2 | *JAM3* | Junctional adhesion molecule C | 0.004 | 0.52 |
| P43652 | *PROS1* | Vitamin K-dependent protein S | 0.033 | 0.52 |
| Q9BUK0 | *SYN1* | Isoform IB of Synapsin-1 | 0.029 | 0.52 |
| Q15528 | *ADCY5* | Adenylate cyclase type 5 | 0.001 | 0.52 |
| P54105 | *RAB6B* | Ras-related protein Rab-6B | 0.002 | 0.53 |
| Q9BZ67 | *UCHL1* | Ubiquitin carboxyl-terminal hydrolase isozyme L1 | 0.013 | 0.53 |
| Q9Y375 | *HEBP1* | Heme-binding protein 1 | 0.027 | 0.53 |
| Q92563 | *CRB3* | Crumbs protein homolog 3 | 0.018 | 0.54 |
| Q96RP9 | *BCL2* | Isoform Beta of Apoptosis regulator Bcl-2 | 0.040 | 0.54 |
| Q14012 | *MAOA* | Amine oxidase [flavin-containing] A | 0.046 | 0.55 |
| O60243 | *SARDH* | Sarcosine dehydrogenase, mitochondrial | 0.021 | 0.55 |
| O43505 | *NID1* | Nidogen-1 | 0.026 | 0.56 |
| Q9UJ68 | *VWDE* | von Willebrand factor D and EGF domain-containing protein | 0.038 | 0.56 |
| P22102 | *SYNE3* | Isoform 2 of Nesprin-3 | 0.004 | 0.56 |
| Q9Y3A5 | *ACLY* | Isoform 2 of ATP-citrate synthase | 0.002 | 0.56 |
| Q96CN9 | *PLEKHB1* | Isoform 4 of Pleckstrin homology domain-containing family B member 1 | 0.014 | 0.56 |
| Q9NY33 | *CYSTM1* | UPF0467 protein C5orf32 | 0.036 | 0.56 |
| P20700 | *CRYL1* | Isoform 2 of Lambda-crystallin homolog | 0.001 | 0.56 |
| P02794 | *PLCD1* | 1-phosphatidylinositol-4,5-bisphosphate phosphodiesterase delta-1 | 0.001 | 0.56 |
| Q9UKM9 | *CPVL* | Probable serine carboxypeptidase CPVL | 0.002 | 0.57 |
| O95372 | *MSRA* | Isoform 2 of Mitochondrial peptide methionine sulfoxide reductase | 0.044 | 0.57 |
| Q96E11 | *CHCHD7* | Coiled-coil-helix-coiled-coil-helix domain-containing protein 7 | 0.045 | 0.59 |
| Q96DC8 | *KBTBD6* | Kelch repeat and BTB domain-containing protein 6 | 0.016 | 0.59 |
| Q96DB2 | *CTSZ* | Cathepsin Z | 0.049 | 0.60 |
| P32322 | *METTL7A* | Methyltransferase-like protein 7A | 0.024 | 0.60 |
| P10415 | *ALDH2* | Aldehyde dehydrogenase, mitochondrial | 0.039 | 0.62 |
| P55072 | *NAP1L5* | Nucleosome assembly protein 1-like 5 | 0.022 | 0.62 |
| P05091 | *HS6ST1* | Isoform 2 of Heparan-sulfate 6-O-sulfotransferase 1 | 0.044 | 0.62 |
| O75525 | *LYRM5* | LYR motif-containing protein 5 | 0.005 | 0.62 |
| Q9Y5K6 | *PSAP* | Proactivator polypeptide | 0.031 | 0.63 |
| Q6NUQ1 | *STEAP3* | Isoform 4 of Metalloreductase STEAP3 | 0.036 | 0.63 |
| O43633 | *AFM* | Afamin | 0.045 | 0.63 |
| O75962 | *ECHDC3* | Enoyl-CoA hydratase domain-containing protein 3, mitochondrial | 0.041 | 0.63 |
| Q9NRX5 | *CD14* | Monocyte differentiation antigen CD14 | 0.002 | 0.63 |
| Q04760 | *PPT1* | Isoform 2 of Palmitoyl-protein thioesterase 1 | 0.032 | 0.64 |
| Q8N2E2 | *HDAC11* | Histone deacetylase 11 | 0.041 | 0.64 |
| Q96C90 | *#N/A* | Selenoprotein M | 0.050 | 0.64 |
| Q12904 | *MCCC1* | Methylcrotonoyl-CoA carboxylase subunit alpha, mitochondrial | 0.014 | 0.64 |
| Q02750 | *TMOD1* | Tropomodulin-1 | 0.004 | 0.65 |
| O00159 | *ACADVL* | Isoform 2 of Very long-chain specific acyl-CoA dehydrogenase, mitochondrial | 0.004 | 0.65 |
| Q9Y4E6 | *FECH* | Ferrochelatase, mitochondrial O | 0.012 | 0.65 |
| P49585 | *ABHD10* | Abhydrolase domain-containing protein 10, mitochondrial | 0.002 | 0.65 |
| Q658P3 | *CMAS* | N-acylneuraminate cytidylyltransferase | 0.014 | 0.66 |
| Q9H1C7 | *FLNB* | Isoform 5 of Filamin-B | 0.046 | 0.66 |
| P48735 | *ACAD9* | Acyl-CoA dehydrogenase family member 9, mitochondrial | 0.007 | 0.66 |
| Q9UI15 | *HTATSF1* | HIV Tat-specific factor 1 | 0.022 | 0.66 |
| P57735 | *RANBP6* | Ran-binding protein 6 | 0.049 | 0.67 |
| O75718 | *EHD4* | EH domain-containing protein 4 | 0.006 | 0.68 |
| Q86SR1 | *MYO1C* | Isoform 2 of Myosin-Ic | 0.037 | 0.68 |
| Q86TU7 | *MGST3* | Microsomal glutathione S-transferase 3 | 0.046 | 0.68 |
| Q8WY22 | *FTH1* | Ferritin heavy chain | 0.043 | 0.68 |
| Q9Y2G0 | *CFL2* | Cofilin-2 | 0.022 | 0.68 |
| P21757 | *ACAA2* | 3-ketoacyl-CoA thiolase, mitochondrial | 0.023 | 0.68 |
| Q14653 | *STAT5B* | Signal transducer and activator of transcription 5B | 0.000 | 0.69 |
| P41252 | *NAAA* | Isoform 2 of N-acylethanolamine-hydrolyzing acid amidase | 0.013 | 0.70 |
| Q9Y3D0 | *ISOC1* | Isochorismatase domain-containing protein 1 | 0.046 | 0.70 |
| P07225 | *IVD* | Isovaleryl-CoA dehydrogenase, mitochondrial | 0.013 | 0.70 |
| Q8ND24 | *DNTTIP1* | Deoxynucleotidyltransferase terminal-interacting protein 1 | 0.050 | 0.70 |
| P56277 | *NBEA* | Neurobeachin | 0.024 | 0.71 |
| P50897 | *SERINC1* | Serine incorporator 1 | 0.038 | 0.71 |
| O15247 | *SRI* | Sorcin | 0.006 | 0.71 |
| Q4G0J3 | *XPC* | DNA repair protein complementing XP-C cells | 0.029 | 0.71 |
| Q9BVJ7 | *TKT* | Transketolase | 0.017 | 0.71 |
| P07602 | *ECHS1* | Enoyl-CoA hydratase, mitochondrial | 0.013 | 0.71 |
| Q13642 | *EFR3B* | Isoform 3 of Protein EFR3 homolog B | 0.035 | 0.72 |
| Q9HCU9 | *GNAI2* | Isoform 2 of Guanine nucleotide-binding protein G(i) subunit alpha-2 | 0.013 | 0.72 |
| Q9H0S4 | *MSR1* | Isoform II of Macrophage scavenger receptor types I and II | 0.035 | 0.73 |
| A5YKK6 | *GNG12* | Guanine nucleotide-binding protein G(I)/G(S)/G(O) subunit gamma-12 | 0.027 | 0.73 |
| Q9UQ26 | *ATP2B4* | Isoform ZB of Plasma membrane calcium-transporting ATPase 4 | 0.013 | 0.73 |
| Q5H9L2 | *GLO1* | Isoform 2 of Lactoylglutathione lyase | 0.038 | 0.73 |
| Q9Y385 | *CDKN1B* | Cyclin-dependent kinase inhibitor 1B | 0.003 | 0.73 |
| Q96SB8 | *LTA4H* | Leukotriene A-4 hydrolase | 0.026 | 0.74 |
| P16333 | *SPRYD4* | SPRY domain-containing protein 4 | 0.022 | 0.74 |
| Q01831 | *CAMK1* | Calcium/calmodulin-dependent protein kinase type 1 | 0.044 | 0.75 |
| O43933 | *SSBP1* | Single-stranded DNA-binding protein, mitochondrial | 0.001 | 0.75 |
| Q9NR46 | *MSN* | Moesin | 0.050 | 0.75 |
| Q9P2W9 | *GNA13* | Guanine nucleotide-binding protein subunit alpha-13 | 0.026 | 0.75 |
| O15355 | *ESD* | S-formylglutathione hydrolase | 0.012 | 0.76 |
| P17600 | *NCK1* | Cytoplasmic protein NCK1 | 0.029 | 0.76 |
| P53041 | *STXBP3* | Syntaxin-binding protein 3 | 0.007 | 0.76 |
| Q86YR5 | *BSPRY* | Isoform 2 of B box and SPRY domain-containing protein | 0.046 | 0.76 |
| P09486 | *RIMS2* | Isoform 4 of Regulating synaptic membrane exocytosis protein 2 | 0.030 | 0.76 |
| P14543 | *ACAT1* | Acetyl-CoA acetyltransferase, mitochondrial | 0.022 | 0.76 |
| P23528 | *KRI1* | Isoform 2 of Protein KRI1 homolog | 0.024 | 0.77 |
| Q8WU76 | *VPS13A* | Isoform 4 of Vacuolar protein sorting-associated protein 13A | 0.002 | 0.77 |
| Q9UBF2 | *RBM47* | Isoform 2 of RNA-binding protein 47 | 0.020 | 0.77 |
| P35858 | *GFM1* | Elongation factor G, mitochondrial | 0.044 | 0.77 |
| Q9NQR4 | *MCFD2* | Multiple coagulation factor deficiency protein 2 | 0.015 | 0.77 |
| P78356 | *PCYT1A* | Choline-phosphate cytidylyltransferase A | 0.036 | 0.77 |
| Q9UBI6 | *SBDS* | Ribosome maturation protein SBDS | 0.043 | 0.77 |
| Q96EY8 | *PCK2* | Phosphoenolpyruvate carboxykinase [GTP], mitochondrial | 0.009 | 0.78 |
| Q9NRV9 | *COPS7A* | COP9 signalosome complex subunit 7a | 0.011 | 0.78 |
| A6NIH7 | *NDUFAF1* | Complex I intermediate-associated protein 30, mitochondrial | 0.044 | 0.79 |
| Q14344 | *MAP2K1* | Isoform 2 of Dual specificity mitogen-activated protein kinase kinase 1 | 0.037 | 0.79 |
| A8MW06 | *CETN2* | Centrin-2 | 0.012 | 0.79 |
| P14543 | *NIT2* | Omega-amidase NIT2 | 0.027 | 0.79 |
| Q15147 | *RAB7A* | Ras-related protein Rab-7a | 0.007 | 0.80 |
| P09960 | *METAP2* | Methionine aminopeptidase 2 | 0.001 | 0.81 |
| Q8NEJ9 | *MRRF* | Isoform 8 of Ribosome-recycling factor, mitochondrial | 0.042 | 0.81 |
| Q9UDR5 | *PIP4K2B* | Phosphatidylinositol-5-phosphate 4-kinase type-2 beta | 0.027 | 0.81 |
| Q8NFP9 | *KPNA4* | Importin subunit alpha-4 | 0.003 | 0.81 |
| Q8N9T8 | *GNPDA1* | Glucosamine-6-phosphate isomerase 1 | 0.022 | 0.81 |
| Q9H8H3 | *UNC119B* | Protein unc-119 homolog B | 0.026 | 0.81 |
| Q96RS6 | *COX5A* | Cytochrome c oxidase subunit 5A, mitochondrial | 0.019 | 0.81 |
| P42765 | *PPP1R12A* | Isoform 4 of Protein phosphatase 1 regulatory subunit 12A | 0.017 | 0.82 |
| O43719 | *WDR7* | Isoform 2 of WD repeat-containing protein 7 | 0.036 | 0.83 |
| Q8WW59 | *MMAB* | Cob(I)yrinic acid a,c-diamide adenosyltransferase, mitochondrial | 0.027 | 0.83 |
| P24752 | *CMC4* | Mature T-cell proliferation 1 neighbor protein | 0.032 | 0.85 |
| Q96NT1 | *ATP6V1H* | Isoform 2 of V-type proton ATPase subunit H | 0.047 | 0.85 |
| Q9P2X0 | *CHMP2A* | Charged multivesicular body protein 2a | 0.039 | 0.87 |
| Q9Y281 | *RAC2* | Ras-related C3 botulinum toxin substrate 2 | 0.010 | 0.87 |
| P46926 | *SMYD5* | SET and MYND domain-containing protein 5 | 0.047 | 0.88 |
| O95630 | *FRMD8* | Isoform 2 of FERM domain-containing protein 8 | 0.044 | 0.88 |
|  |  |  |  |  |
