# Supplementary material for: Genetic and epigenetic background and protein expression profiles in relation to telomerase activation in medullary thyroid carcinoma
Source: Oncotarget. 2016 Feb 8;7(16):21332–46. doi: 10.18632/oncotarget.7237 (PMC5008288; doi:10.18632/oncotarget.7237)
Supplement: Supplementary file 6 [file oncotarget-07-21332-s006.doc]

| **Supplementary Table S5. The 93 proteins identified in the OPLS model for telomerase positive MTCs (n =8) and used for pathway analysis.** | | |
| --- | --- | --- |
|  | | |
| **Accession no.** | **Symbol** | **Name** |
| Q8WY22 | BRI3BP | BRI3-binding protein |
| Q9P035 | PTPLAD1 | 3-hydroxyacyl-CoA dehydratase 3 |
| P25787 | PSMA2 | Proteasome subunit alpha type-2 |
| Q8TDN6 | BRIX1 | Ribosome biogenesis protein BRX1 homolog |
| O14818 | PSMA7 | Proteasome subunit alpha type-7 |
| Q99832 | CCT7 | T-complex protein 1 subunit eta |
| P0CAP2 | POLR2M | Isoform 3 of DNA-directed RNA polymerase II subunit GRINL1A |
| O00401 | WASL | Neural Wiskott-Aldrich syndrome protein |
| O75391 | SPAG7 | Sperm-associated antigen 7 |
| Q9C0H9 | SRCIN1 | Isoform 2 of SRC kinase signaling inhibitor 1 |
| P28066 | PSMA5 | Proteasome subunit alpha type-5 |
| Q9UBF2 | COPG2 | Coatomer subunit gamma-2 |
| O75569 | PRKRA | Interferon-inducible double stranded RNA-dependent protein kinase activator A |
| Q04206 | RELA | Isoform 2 of Transcription factor p65 |
| Q12788 | TBL3 | Transducin beta-like protein 3 |
| P28340 | POLD1 | DNA polymerase delta catalytic subunit |
| P25786 | PSMA1 | Proteasome subunit alpha type-1 |
| P60900 | PSMA6 | Proteasome subunit alpha type-6 |
| O14531 | DPYSL4 | Dihydropyrimidinase-related protein 4 |
| P23528 | CFL1 | Cofilin-1 |
| P52209 | PGD | 6-phosphogluconate dehydrogenase, decarboxylating |
| Q9UKF7 | PITPNC1 | Isoform 2 of Cytoplasmic phosphatidylinositol transfer protein 1 |
| P05186 | ALPL | Alkaline phosphatase, tissue-nonspecific isozyme |
| P40227 | CCT6A | T-complex protein 1 subunit zeta |
| Q9H267 | VPS33B | Vacuolar protein sorting-associated protein 33B |
| Q86VP1 | TAX1BP1 | Isoform 3 of Tax1-binding protein 1 |
| Q8N568 | DCLK2 | Isoform 2 of Serine/threonine-protein kinase DCLK2 |
| Q9NUJ1 | ABHD10 | Abhydrolase domain-containing protein 10, mitochondrial |
| P02753 | RBP4 | Retinol-binding protein 4 |
| Q99797 | MIPEP | Mitochondrial intermediate peptidase |
| P50990 | CCT8 | T-complex protein 1 subunit theta |
| Q92878 | RAD50 | DNA repair protein RAD50 |
| P16949 | STMN1 | Stathmin |
| Q9BVC6 | TMEM109 | Transmembrane protein 109 |
| Q16643 | DBN1 | Drebrin |
| Q9NZ72 | STMN3 | Stathmin-3 |
| O60243 | HS6ST1 | Isoform 2 of Heparan-sulfate 6-O-sulfotransferase 1 |
| P05455 | SSB | Lupus La protein |
| O00139 | KIF2A | Isoform 2 of Kinesin-like protein KIF2A |
| Q9NRG9 | AAAS | Aladin |
| P48643 | CCT5 | T-complex protein 1 subunit epsilon |
| P42898 | MTHFR | Methylenetetrahydrofolate reductase |
| P52701 | MSH6 | Isoform GTBP-alt of DNA mismatch repair protein Msh6 |
| Q01105 | SET | Isoform 2 of Protein SET |
| P10515 | DLAT | Dihydrolipoyllysine-residue acetyltransferase component of pyruvate dehydrogenase complex, mitochondrial |
| Q8IV48 | ERI1 | 3'-5' exoribonuclease 1 |
| P13010 | XRCC5 | X-ray repair cross-complementing protein 5 |
| Q96GM5 | SMARCD1 | Isoform 2 of SWI/SNF-related matrix-associated actin-dependent regulator of chromatin subfamily D member 1 |
| P78371 | CCT2 | T-complex protein 1 subunit beta |
| P23434 | GCSH | Glycine cleavage system H protein, mitochondrial |
| Q13283 | G3BP1 | Ras GTPase-activating protein-binding protein 1 |
| Q9H910 | HN1L | Isoform 2 of Hematological and neurological expressed 1-like protein |
| Q13509 | TUBB3 | Tubulin beta-3 chain |
| Q9Y3A3 | MOB4 | Isoform 2 of MOB-like protein phocein |
| Q9H3G5 | CPVL | Probable serine carboxypeptidase CPVL |
| Q8N8S7 | ENAH | Isoform 2 of Protein enabled homolog |
| Q9BU89 | DOHH | Deoxyhypusine hydroxylase |
| P50416 | CPT1A | Isoform 2 of Carnitine O-palmitoyltransferase 1, liver isoform |
| Q9BUL5 | PHF23 | Isoform 2 of PHD finger protein 23 |
| Q13263 | TRIM28 | Transcription intermediary factor 1-beta |
| Q9NRL3 | STRN4 | Striatin-4 |
| Q96I59 | NARS2 | Probable asparagine--tRNA ligase, mitochondrial |
| Q9BX67 | JAM3 | Junctional adhesion molecule C |
| P19634 | SLC9A1 | Sodium/hydrogen exchanger 1 |
| Q9NWZ8 | GEMIN8 | Gem-associated protein 8 |
| Q8TCX1 | DYNC2LI1 | Isoform 5 of Cytoplasmic dynein 2 light intermediate chain 1 |
| Q9BRX9 | WDR83 | WD repeat domain-containing protein 83 |
| Q969G3 | SMARCE1 | Isoform 2 of SWI/SNF-related matrix-associated actin-dependent regulator of chromatin subfamily E member 1 |
| P53582 | METAP1 | Methionine aminopeptidase 1 |
| Q15036 | SNX17 | Sorting nexin-17 |
| P26038 | MSN | Moesin |
| O95573 | ACSL3 | Long-chain-fatty-acid--CoA ligase 3 |
| P55010 | EIF5 | Eukaryotic translation initiation factor 5 |
| P00568 | AK1 | Adenylate kinase isoenzyme 1 |
| Q92541 | RTF1 | RNA polymerase-associated protein RTF1 homolog |
| P41732 | TSPAN7 | Tetraspanin-7 |
| P12956 | XRCC6 | X-ray repair cross-complementing protein 6 |
| O75683 | SURF6 | Surfeit locus protein 6 |
| P09211 | GSTP1 | Glutathione S-transferase P |
| Q9Y530 | OARD1 | O-acetyl-ADP-ribose deacetylase C6orf130 |
| Q8TF05 | PPP4R1 | Isoform 2 of Serine/threonine-protein phosphatase 4 regulatory subunit 1 |
| P43246 | MSH2 | DNA mismatch repair protein Msh2 |
| P08758 | ANXA5 | Annexin A5 O |
| P25789 | PSMA4 | Proteasome subunit alpha type-4 |
| P78356 | PIP4K2B | Phosphatidylinositol-5-phosphate 4-kinase type-2 beta |
| P54289 | CACNA2D1 | Isoform 4 of Voltage-dependent calcium channel subunit alpha-2/delta-1 |
| Q96N66 | MBOAT7 | Isoform 3 of Lysophospholipid acyltransferase 7 |
| P49006 | MARCKSL1 | MARCKS-related protein |
| P17812 | CTPS1 | CTP synthase 1 |
| Q9Y512 | SAMM50 | Sorting and assembly machinery component 50 homolog |
| P50991 | CCT4 | T-complex protein 1 subunit delta |
| Q9BVJ7 | DUSP23 | Dual specificity protein phosphatase 23 |
| Q96GQ7 | DDX27 | Probable ATP-dependent RNA helicase DDX27 |
| OPLS = orthogonal projections to latent structures | | |
|  |  |  |
